# Failed induction of human labour is associated with an altered myometrial phosphoproteome

**DOI:** 10.1038/s41598-025-27605-6

**Published:** 2025-12-01

**Authors:** Katherine Alice Birchenall, Claire Hudson, Philip A. Lewis, Rachna Bahl, Sarah Quinn, Kate Heesom, Gavin Iain Welsh, Andrés López Bernal

**Affiliations:** 1 Department of Obstetrics and Gynaecology, St Michael’s Hospital, Southwell Street, Bristol, BS2 8EG UK; 2 Translational Health Sciences, University of Bristol, Dorothy Hodgkin Building, Whitson Street, Bristol, BS1 3NY UK; 3 School of Medicine and Public Health, The University of Newcastle, Callaghan, New South Wales Australia; 4 Mothers and Babies Research Program, Hunter Medical Research Institute, New Lambton Heights, New South Wales Australia; 5 University of Bristol Proteomics Facility, Biomedical Sciences Building, University Walk, Bristol, BS8 1TD UK

**Keywords:** Proteomics, Reproductive biology

## Abstract

Induction of labour (IOL) is increasingly recommended for a variety of maternal and fetal indications. Approximately 30% of labours that are induced result in an emergency caesarean section (failed induction of labour). We have previously reported that the feto-placental metabolome of spontaneous labour is not reproduced by current methods of IOL. Here, we have investigated the myometrial phosphoproteome and found an altered phenotype associated with failed IOL. We conducted global phosphoproteomics analysis of human myometrium obtained at the time of term elective caesarean section from women who had previously had a vaginal birth and compared with myometrium obtained at the time of emergency caesarean from women with a failed IOL. The myometrium was cut into strips and placed in an organ bath under different experimental conditions: pre-contracting; spontaneously contracting at peak contraction and peak relaxation; and oxytocin-induced contracting state at peak contraction and peak relaxation. Human myometrial proteins and phosphorylation events were differentially expressed in myometrium from women with failed IOL compared with myometrium from women with previous vaginal birth. This included changes within key pathways of cytoskeletal remodelling, extracellular matrix dysregulation, and defective metabolic adaptation; such differences may reflect an altered myometrial phenotype in failed IOL and open new areas of research to improve the clinical induction of labour.

**Supplementary Information:**

The online version contains supplementary material available at 10.1038/s41598-025-27605-6.

## Introduction

 Our understanding of the mechanisms of human parturition remains incomplete due to the complex and dynamic hormonal and functional changes that occur within the utero-feto-placental unit towards the end of gestation and the difficulties in designing non-invasive experiments during late pregnancy and labour. Globally, increasing numbers of women have their labours induced^1,2^, including approximately a third of all pregnancies in the UK, and it is estimated that 15–30% of induced labours result in an emergency caesarean section (emCS), often for failure to progress in labour^3,4^. Clinically, failure to progress in labour (also known as labour dystocia) may be secondary to cephalopelvic disproportion, where the fetus is deemed too large for the maternal pelvis, or because of fetal malposition^5^. However, for some women there may be an underlying functional problem of the myometrium which impacts its capacity to generate strong regular contractions over several hours of labour.

Human myometrium undergoes phasic contraction and relaxation cycles because of spontaneous cell membrane depolarisation and changes in intracellular calcium (Ca^2+^). Phasic contractions are actioned by the reversible and opposing actions of the enzymes myosin light chain kinase (MYLK) and myosin phosphatase (MYLP), coordinated via Ca^2+^ entry and extrusion mechanisms^6–8^. MYLK is a Ca^2+^-calmodulin (Ca-CaM)-dependent enzyme that reversibly phosphorylates myosin light chains (MLC) and is driven by Ca^2+^ concentration ([Ca^2+^]); by contrast, MYLP is regulated by phosphorylation which is Ca^2+^-independent^8^. Oxytocin (OXT) is commonly used to induce uterine contractility in labour and acts through binding to G protein-coupled receptors to increase Ca^2+^ influx into myocytes via the Gq/Phospholipase C pathway^8,9^. We have previously undertaken investigation of the phosphoproteome of the human myometrium during spontaneous and oxytocin-induced contractions in vitro, and shown that the reversible phosphorylation of a regulatory subunit of MYLP (at Threonine 853) can occur during phasic myometrial contraction, and that this plays a role in the [Ca^2+^]-sensitivity induced by OXT^8^.

Genomic analysis of human myometrium has highlighted genes altered during emCS from pathways related to hypoxia, inflammation, apoptosis, stress, muscle contraction and chemokine signalling^5^, and previous studies suggest a differential transcriptome for arrest of labour descent^10,11^. These studies are useful to determine which genes are transcribed within the myometrium during labour, however genomic methods do not always reflect the proteins produced^12,13^, nor the post-translational phosphorylation of proteins, which is an important regulatory mechanism to enhance or inhibit protein function in the tissue^14^.

We have previously reported that the feto-placental metabolome of spontaneous labour is not replicated by IOL^15^, and suggested the activation of alternative pathways. We now propose that there is a phenotype of myometrium that is more likely to result in failed IOL and have conducted global phosphoproteomics analyses to enable investigation of the total proteome of myometrial tissue alongside protein-phosphorylation events. This analytical method allows direct comparison of the phosphoproteome of myometrium sampled from women who birthed via elective caesarean section (elCS) at term (not in labour) with myometrium sampled from women who birthed via emCS following failed IOL at term. The differences identified between these two groups presented here reflect a myometrial phenotype associated with failure to progress following IOL. We suggest it is likely that a proportion of these proteins are required for effective coordination of myometrial contractions to enable timely vaginal birth, and that identification of these protein changes will contribute to improved understanding of the mechanism of spontaneous labour in women and provide targets for more efficient IOL.

## Results

This study compares the global analysis of myometrial phosphoproteome changes during spontaneous and oxytocin-induced contractions in vitro of myometrium sampled from women who birthed via elCS and who had a history (previous pregnancy) of reaching full cervical dilatation, with that of myometrium sampled from women with a failed IOL resulting in an emCS. Samples from six women were included in the study: three who had an elCS having laboured to full dilatation in a previous pregnancy, and three who had an emCS following IOL for post-term in their first pregnancy.

Supplementary Information (SI) 1 shows the participant demographics. All those in the elCS group had previously had one pregnancy where they had laboured and experienced effective uterine contractions, enabling full cervical dilatation to allow vaginal birth. The indications for elCS in their current pregnancies were placenta praevia, maternal request, and previous third-degree perineal tear. These women were selected for inclusion in this group as their myometrium had previously been proven to be effective in enabling vaginal birth. In contrast, the women in the IOL group were in their first pregnancy and were induced for being post-term (“overdue”), and were selected as they had not yet laboured despite being post-term. None of the participants had any other maternal or fetal complications. The IOL process included vaginal prostaglandin administration followed by artificial rupture of membranes and intravenous infusion of oxytocin, as per local protocol. All three in the failed IOL group had an emCS in labour with failure to progress: one labour failed to progress beyond 3 cm dilatation and there was fetal distress; one failed to progress beyond 6 cm; and the other failed to progress beyond 8 cm dilatation. None of the women developed an infection in labour. The gestational age at birth was different between the elCS and emCS groups, expected as elCS are routinely conducted between 39 and 40 completed weeks’ gestation, whereas all the failed IOL group were induced for post-term, which was routinely offered from 40 weeks and 12 days gestation at St Michael’s Hospital at the time of the study.

### Global comparison of myometrial proteome between term not-in-labour myometrium (with previous successful vaginal birth) and term in-labour myometrium from women with failed IOL

**Fig. 1:**
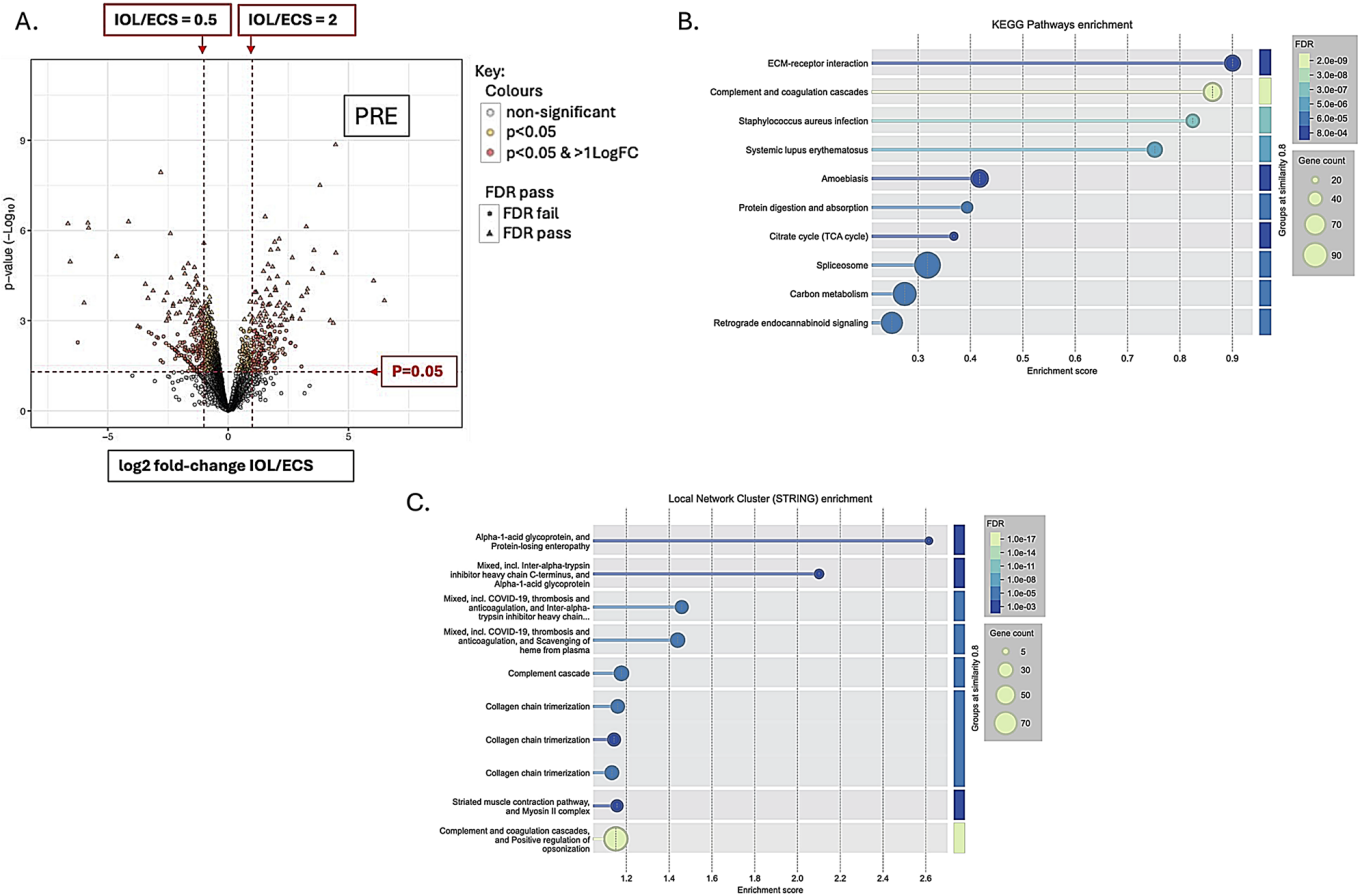
Total protein analysis for comparison of failed IOL group and elCS group under the pre-contracting condition. (**A**) Volcano plot illustrating the mean log2-fold change in total protein between the failed IOL group (n=3) and the elCS group (n=3) (x-axis) plotted against the -log10(p-value) (y-axis) in the pre-contracting condition (PRE). Horizontal dashed red line indicates p=0.05, above which differences between the failed IOL group and elCS group are significant; two vertical red dashed lines indicate halving and doubling between the failed IOL and elCS groups. (**B**) Bubble plot illustrating significantly enriched KEGG pathways identified through STRING’s functional enrichment analysis. Proteins ranked by log-fold change, and enrichment calculated using Kolmogorov-Smirnov (KS) test. Significance displayed as -log10(False Discovery Rate (FDR)); lighter colours indicate stronger enrichment; bubble size represents the number of proteins (gene count) associated with each KEGG pathway. Only pathways meeting an FDR threshold of ≤0.05 are shown. (**C**) Bubble plot displaying enriched local network clusters based on STRING’s protein-protein interaction network. Each cluster represents a densely connected subnetwork of proteins with potential shared biological function. Enrichment assessed using the KS test. Significance is indicated by -log10(FDR), with lighter colours reflecting stronger enrichment. Bubble size corresponds to the number of proteins (gene count) within each enriched cluster. Only clusters with FDR ≤ 0.05 are shown.

**Fig. 2:**
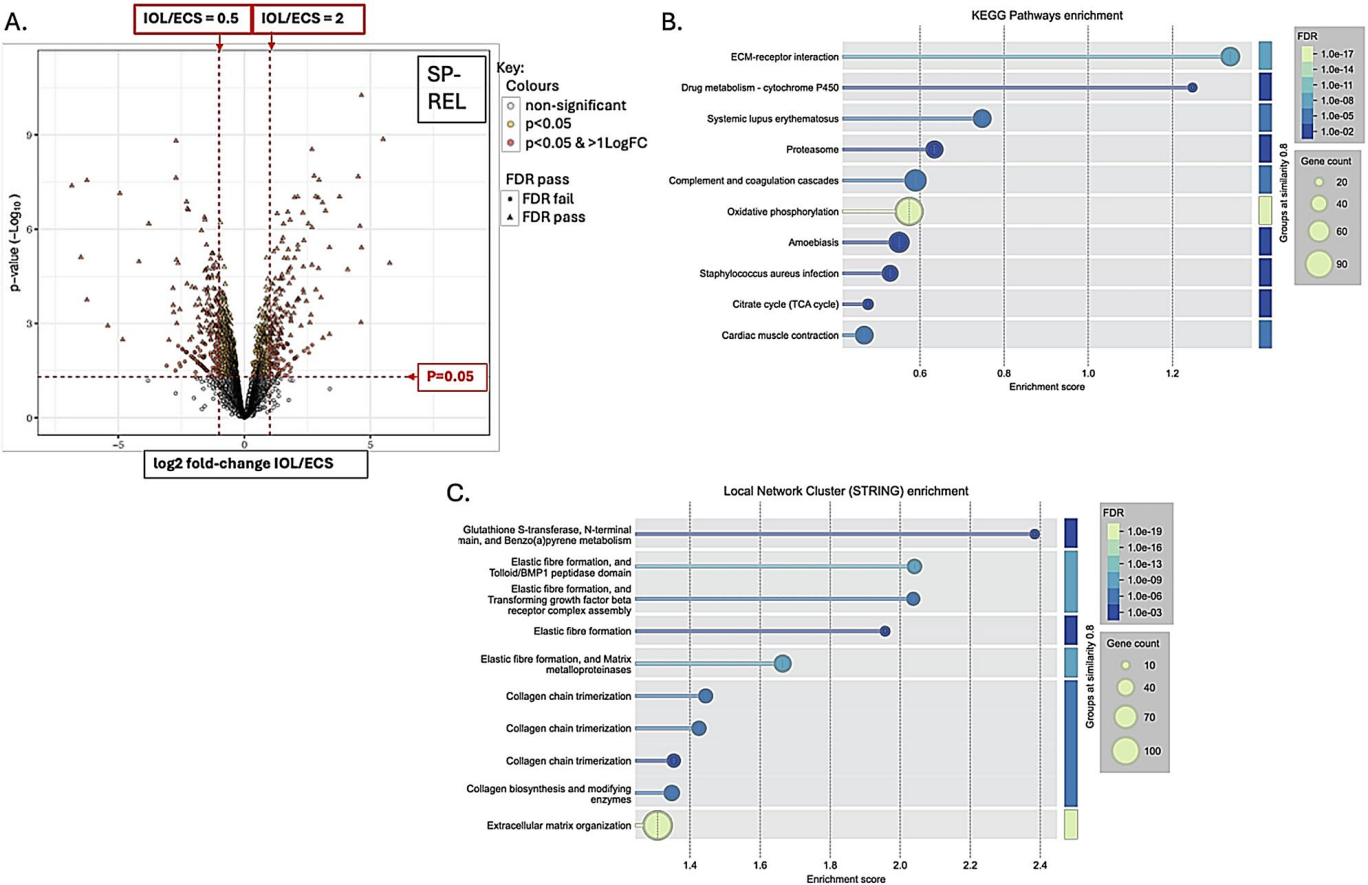
Total protein analysis for comparison of failed IOL group and elCS group under the relaxed-phase during spontaneous contractions condition. (**A**) Volcano plot illustrating the mean log2-fold change in total protein between the failed IOL group (n=3) and the elCS group (n=3) (x-axis) plotted against the -log10(p-value) (y-axis) under the spontaneously contracting at peak relaxation (SP-REL) condition. Horizontal dashed red line indicates p=0.05, above which differences between the failed IOL group and elCS group are significant; two vertical red dashed lines indicate halving and doubling between the failed IOL and elCS groups. (**B**) Bubble plot illustrating significantly enriched KEGG pathways identified through STRING’s functional enrichment analysis. Proteins ranked by log-fold change, and enrichment calculated using Kolmogorov-Smirnov (KS) test. Significance displayed as -log10(False Discovery Rate (FDR)); lighter colours indicate stronger enrichment; bubble size represents the number of proteins (gene count) associated with each KEGG pathway. Only pathways meeting an FDR threshold of ≤0.05 are shown. (**C**) Bubble plot displaying enriched local network clusters based on STRING’s protein-protein interaction network. Each cluster represents a densely connected subnetwork of proteins with potential shared biological function. Enrichment assessed using the KS test. Significance is indicated by -log10(FDR), with lighter colours reflecting stronger enrichment. Bubble size corresponds to the number of proteins (gene count) within each enriched cluster. Only clusters with FDR ≤ 0.05 are shown.

**Fig. 3:**
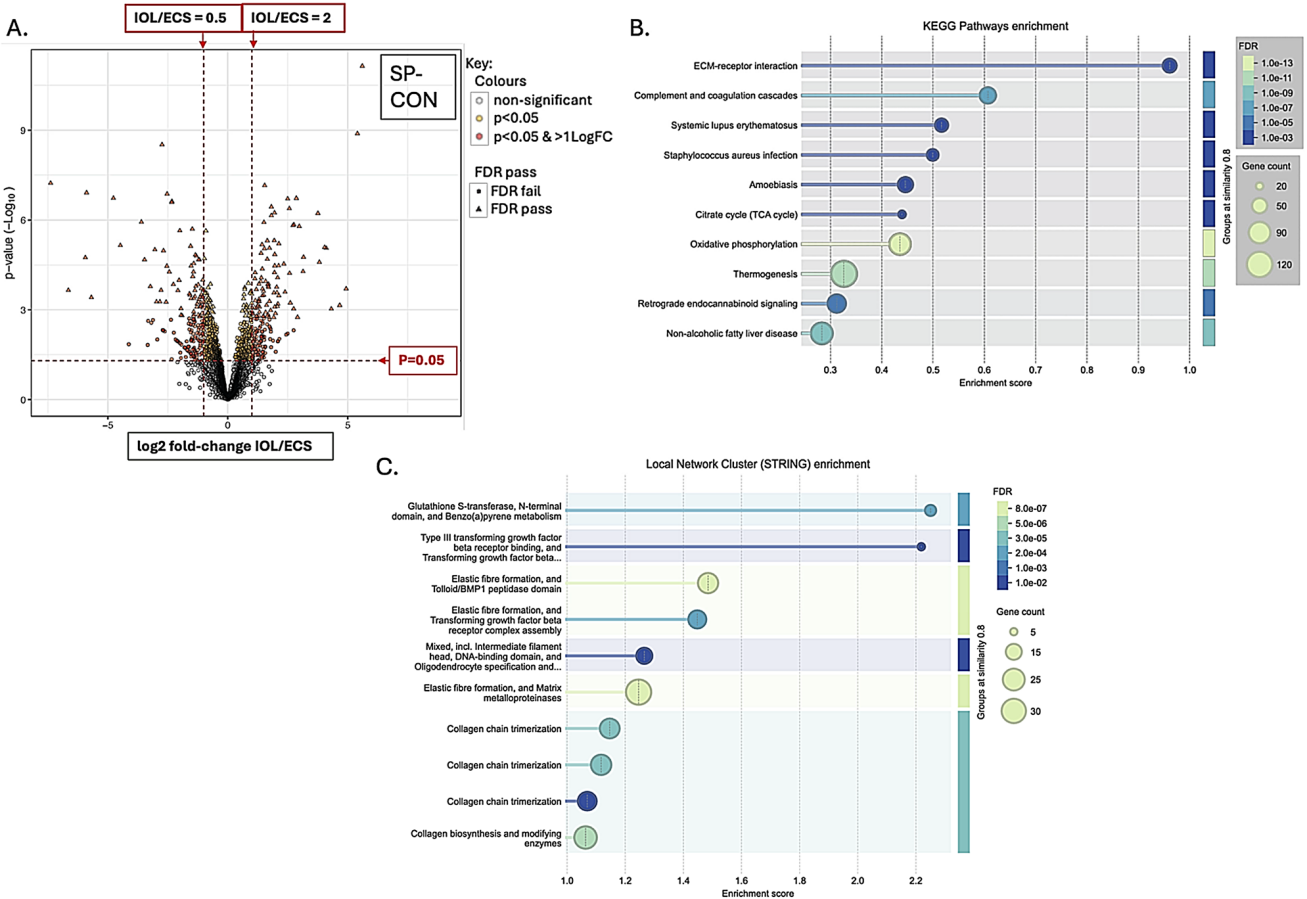
Total protein analysis for comparison of failed IOL group and elCS group under the contracted-phase during spontaneous contractions condition. (**A**) Volcano plot illustrating the mean log2-fold change in total protein between the failed IOL group (n=3) and the elCS group (n=3) (x-axis) plotted against the -log10(p-value) (y-axis) under the spontaneously contracting at peak contraction (SP-CON) condition. Horizontal dashed red line indicates p=0.05, above which differences between the failed IOL group and elCS group are significant; two vertical red dashed lines indicate halving and doubling between the failed IOL and elCS groups. (**B**) Bubble plot illustrating significantly enriched KEGG pathways identified through STRING’s functional enrichment analysis. Proteins ranked by log-fold change, and enrichment calculated using Kolmogorov-Smirnov (KS) test. Significance displayed as -log10(False Discovery Rate (FDR)); lighter colours indicate stronger enrichment; bubble size represents the number of proteins (gene count) associated with each KEGG pathway. Only pathways meeting an FDR threshold of ≤0.05 are shown. (**C**) Bubble plot displaying enriched local network clusters based on STRING’s protein-protein interaction network. Each cluster represents a densely connected subnetwork of proteins with potential shared biological function. Enrichment assessed using the KS test. Significance is indicated by -log10(FDR), with lighter colours reflecting stronger enrichment. Bubble size corresponds to the number of proteins (gene count) within each enriched cluster. Only clusters with FDR ≤ 0.05 are shown.

**Fig. 4:**
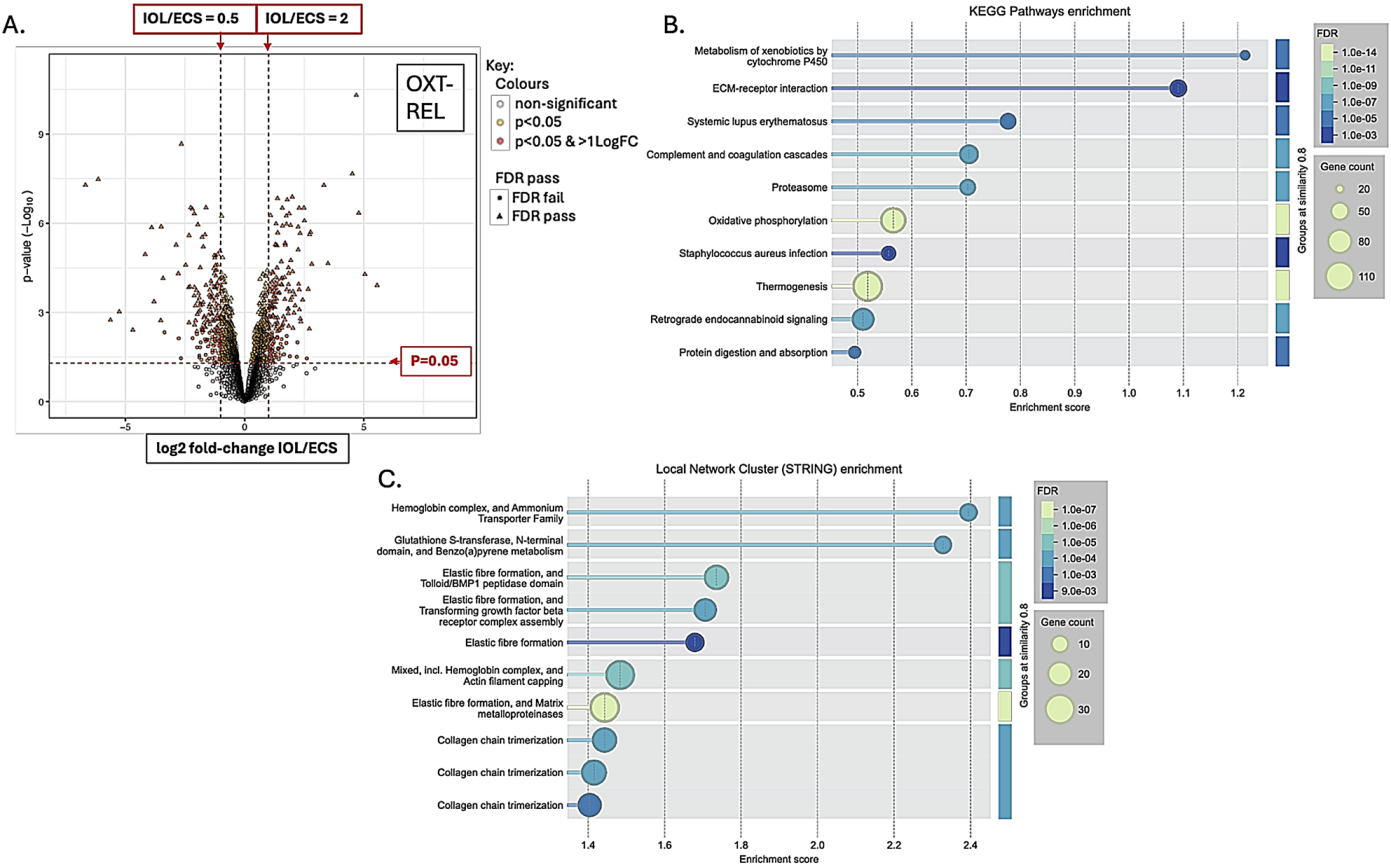
Total protein analysis for comparison of failed IOL group and elCS group under the relaxed-phase during oxytocin-induced contractions condition. (**A**) Volcano plot illustrating the mean log2-fold change in total protein between the failed IOL group (n=3) and the elCS group (n=3) (x-axis) plotted against the -log10(p-value) (y-axis) under the oxytocin-induced contractions at peak relaxation (OXT-REL) condition. Horizontal dashed red line indicates p=0.05, above which differences between the failed IOL group and elCS group are significant; two vertical red dashed lines indicate halving and doubling between the failed IOL and elCS groups. (**B**) Bubble plot illustrating significantly enriched KEGG pathways identified through STRING’s functional enrichment analysis. Proteins ranked by log-fold change, and enrichment calculated using Kolmogorov-Smirnov (KS) test. Significance displayed as -log10(False Discovery Rate (FDR)); lighter colours indicate stronger enrichment; bubble size represents the number of proteins (gene count) associated with each KEGG pathway. Only pathways meeting an FDR threshold of ≤0.05 are shown. (**C**) Bubble plot displaying enriched local network clusters based on STRING’s protein-protein interaction network. Each cluster represents a densely connected subnetwork of proteins with potential shared biological function. Enrichment assessed using the KS test. Significance is indicated by -log10(FDR), with lighter colours reflecting stronger enrichment. Bubble size corresponds to the number of proteins (gene count) within each enriched cluster. Only clusters with FDR ≤ 0.05 are shown.

There were 7755 proteins identified and 2355 phosphopeptides. Figures 1, 2, 3 and 4 A are volcano plots highlighting those proteins which passed the False Discovery Rate (FDR; significant if ≤ 0.05) and those which either significantly halved or doubled in the failed IOL samples compared to the elCS samples under each of the experimental groups: pre-contracting, relaxed-phase during spontaneous contractions, contracted-phase during spontaneous contractions, relaxed-phase during OXT-induced contractions, and contracted-phase during OXT-induced contractions. Table 1 illustrates where the most differences in protein concentration between the two groups under the different experimental conditions occurred. Tables showing those proteins which passed FDR and those proteins which significantly at least doubled or halved in the failed IOL group compared to the elCS group under the different experimental conditions are presented in SI2 – SI11. The most significant increases in total protein in the failed IOL group were seen in the relaxed state, with or without stimulation with oxytocin. SI12 shows the total proteins for which the mean concentration passed FDR (q ≤ 0.05) and at least significantly (*p* ≤ 0.05) doubled in the failed IOL group under one or more of the experimental conditions. This includes 23 proteins for which this was true under all five conditions, including: Annexin A3 (*ANXA3*), cartilage oligomeric matric protein (*COMP*), Collagen alpha-1 (XII) chain (*COL12A1*), Desmuslin, isoform CRA_a (*SYNM*), Matrilin-2 (*MATN2*), Matrix Gla protein (*MGP*) and Lysl oxidase homolog 2 (*LOXL2*), Transforming growth factor-beta-induced protein ig-h3 (*TGFBI*), TNC variant protein, Prostacyclin synthase (*PGIS*), Polypeptide N-acetyl-galactosaminyl-transferase (*GALNT18*), and Vitamin D binding protein (*A0A1B1CYC5*). SI13 lists the total proteins for which the mean concentration at least significantly (*p* ≤ 0.05) halved and passed the false discovery rate (q ≤ 0.05) in the failed IOL group under one or more experimental conditions. This includes 33 proteins for which this was true under all five conditions, including: MHC Class I antigen HLA-A, HLA-B, and HLA-C, A-kinase anchor protein 12 (AKAP12), membrane metallo-endopeptidase/Neprilysin (*MME*), activating signal co-integrator 1 complex subunit 2 (*ASCC2*), endosialin (*CD248*), peptidyl-prolyl cis-trans isomerase-like 3 (*PPIL3*), and tubulin polymerization-promoting protein family member 3 (*TPPP3*).

**Fig. 5:**
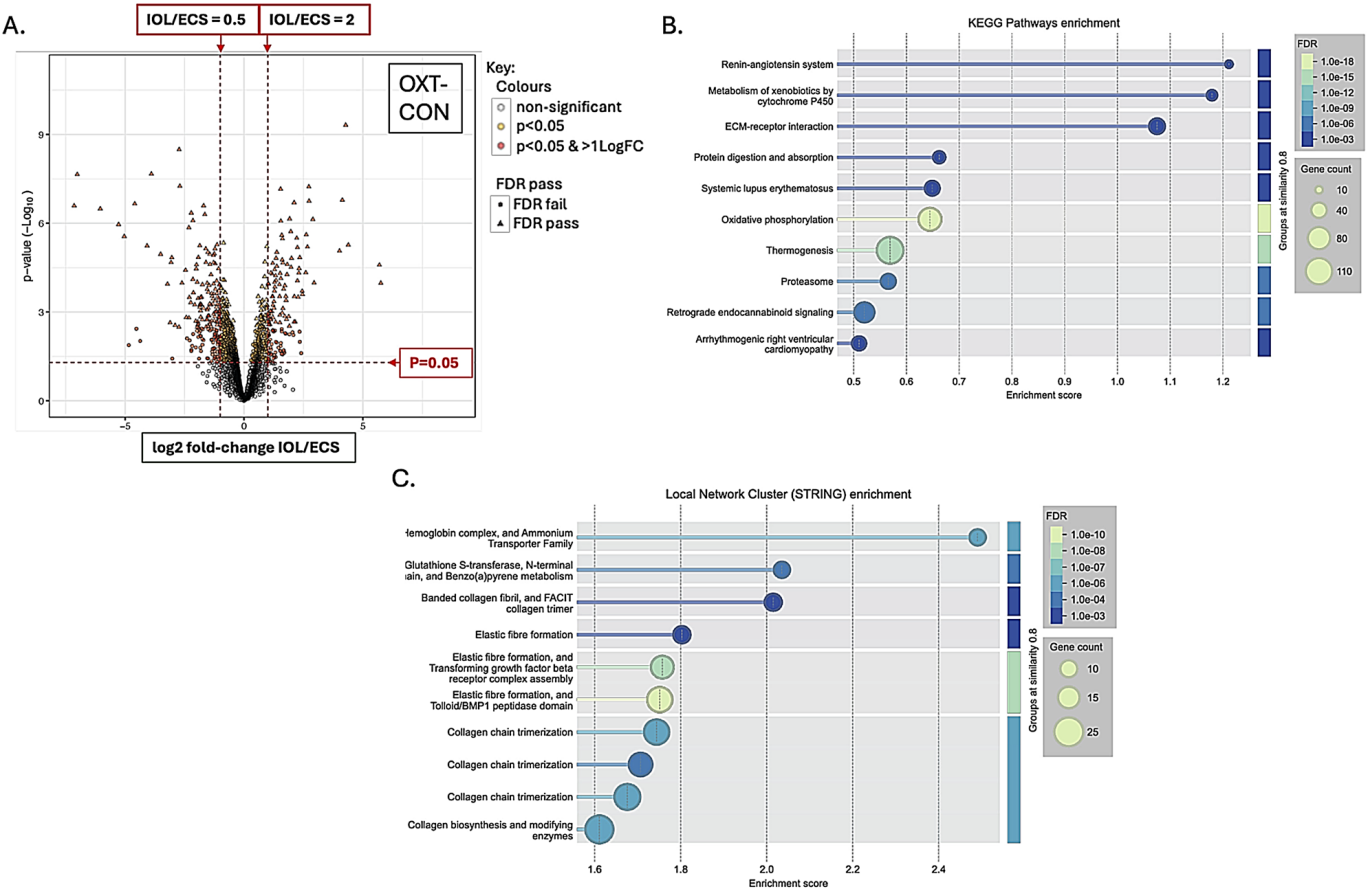
Total protein analysis for comparison of failed IOL group and elCS group under the contracted-phase during oxytocin-induced contractions condition. (**A**) Volcano plot illustrating the mean log2-fold change in total protein between the failed IOL group (n=3) and the elCS group (n=3) (x-axis) plotted against the -log10(p-value) (y-axis) under the oxytocin-induced contractions at peak contraction (OXT-CON) condition. Horizontal dashed red line indicates p=0.05, above which differences between the failed IOL group and elCS group are significant; two vertical red dashed lines indicate halving and doubling between the failed IOL and elCS groups. (**B**) Bubble plot illustrating significantly enriched KEGG pathways identified through STRING’s functional enrichment analysis. Proteins ranked by log-fold change, and enrichment calculated using Kolmogorov-Smirnov (KS) test. Significance displayed as -log10(False Discovery Rate (FDR)); lighter colours indicate stronger enrichment; bubble size represents the number of proteins (gene count) associated with each KEGG pathway. Only pathways meeting an FDR threshold of ≤0.05 are shown. (**C**) Bubble plot displaying enriched local network clusters based on STRING’s protein-protein interaction network. Each cluster represents a densely connected subnetwork of proteins with potential shared biological function. Enrichment assessed using the KS test. Significance is indicated by -log10(FDR), with lighter colours reflecting stronger enrichment. Bubble size corresponds to the number of proteins (gene count) within each enriched cluster. Only clusters with FDR ≤ 0.05 are shown.

Functional protein enrichment analysis was performed using the online STRING programme, performing both KEGG pathway enrichment analysis and local network cluster enrichment on all proteins for which there was a log-fold change available between the IOL and elCS groups under the five different experimental conditions. Figures 1B-C, 2, 3, 4 and 5B-C are bubble plots showing those KEGG pathways and network clusters which passed FDR. SI 14–23 show the full results of these analyses; SI24 illustrates those KEGG pathways which showed functional enrichment where the FDR was significant in at least two of the conditions and SI25 shows the same for the network cluster analysis. KEGG pathways which were enriched under all conditions included: ECM-receptor interaction, oxidative phosphorylation, retrograde endocannabinoid signalling, and protein digestion and absorption (SI24). Local network clusters which were enriched among all five conditions included citrate cycle (TCA cycle), and Pyruvate metabolism, elastic fibre formation and matrix metalloproteinases, extracellular matrix organisation, NADH dehydrogenase (ubiquinone) activity, respiratory chain complex, and respiratory electron transport clusters (SI25).

### Global comparison of phosphoproteome for term not-in-labour myometrium (with previous successful vaginal birth) with term in-labour myometrium from women with failed IOL under different experimental conditions

Overall, fifteen phosphorylation events were differentially expressed among the different conditions and passed FDR (Table 2 and SI26 to SI34): ten phosphopeptides at least halved and five at least doubled. Phosphorylation of Nexilin (F-actin-binding protein (NEXN)) at Serine 179 in the failed IOL group was less than a third that of the elCS group under all five experimental conditions (log2fcs of pre-contracting phase − 3.76; spontaneous relaxed-phase − 3.62; spontaneous contracted-phase − 3.62; OXT relaxed-phase − 4.32; OXT contracted-phase − 3.70). In addition, under the pre-contracting condition, protein prune homolog 2 (PRUNE2) phosphorylation at Serine 699 was reduced − 3.57 log2-fold in the failed IOL group; and under the relaxed-phase during spontaneous contractions condition, Filamin-A (FLN-A) phosphorylation at Serine 968 and at Serine 2180 more than doubled in the failed IOL group. Under the contracted-phase during spontaneous contractions condition, phosphorylation of monocarboxylate transporter 1 (SLC16A1) at Serine 461 and Serine 467 more than doubled in the failed IOL group, and phosphorylation of Nuclear mitotic apparatus protein 1 (NUMA1) at Serine 1763, beta-2-syntrophin (59 kDa dystrophin-associated protein A1 basic component 2) (SNTB2) at Serine 110, and Serine/threonine-protein kinase 10 (EC 2.7.11.1) (STK10) at Serine 438, all at least halved in the failed IOL group. Under the relaxed-phase during OXT-induced contractions condition, synaptopodin 2 (SYNP02) phosphorylation at Y735 was 2.01 log2-fold greater in the failed IOL group, and phosphorylation of chondroitin sulfate proteoglycan 2 (Versican), isoform CRA_c (CSPG2) at Serine 2955 was − 4.33 log2-fold less; and under the contracted-phase during OXT-induced contractions condition, protein transport protein Sec31A (Sect. 31-like protein 1) (SEC31A) phosphorylation at Serine 799 was 1.40 log2-fold greater in the failed IOL group.

**Table 1:** Total number of proteins which either at least doubled or at least halved in the failed IOL group compared with the elCS group under the different experimental conditions. PRE = pre-contracting; SP-REL = relaxed-phase during spontaneous contractions; OXT-REL = relaxed-phase during oxytocin-induced contractions; SP-CON = contracted-phase during spontaneous contractions; OXT-CON = contracted-phase during oxytocin-induced contractions.

Experimental condition	At least doubled in failed IOL group			At least halved in failed IOL group		
	Doubled	Significantly doubled (*p* < 0.05)	Significantly doubled (*p* < 0.05) and passed FDR (q < 0.05)	Halved	Significantly halved (*p* < 0.05)	Significantly halved (*p* < 0.05) and passed FDR (q < 0.05)
PRE	281	218	73	359	282	187
SP-REL	263	228	144	309	261	165
OXT-REL	226	198	136	257	234	172
SP-CON	255	206	94	302	244	107
OXT-CON	179	157	95	291	262	160

**Table 2:** Total number of phosphopeptides which either at least doubled or at least halved in the failed IOL group compared with the elCS group under the different experimental conditions and passed FDR, where darker red indicates higher number; darker blue indicates lower number. PRE = pre-contracting; SP-REL = relaxed-phase during spontaneous contractions; OXT-REL = relaxed-phase during oxytocin-induced contractions; SP-CON = contracted-phase during spontaneous contractions; OXT-CON = contracted-phase during oxytocin-induced contractions.

Experimental condition	At least doubled in failed IOL group			At least halved in failed IOL group		
	Doubled	Significantly doubled (*p* < 0.05)	Significantly doubled (*p* < 0.05) and passed FDR (q < 0.05)	Halved	Significantly halved (*p* < 0.05)	Significantly halved (*p* < 0.05) and passed FDR (q < 0.05)
PRE	60	38	0	82	55	2
SP-REL	49	40	2	61	42	1
OXT-REL	36	27	1	55	38	2
SP-CON	42	28	1	65	39	4
OXT-CON	31	16	1	59	42	1

### Quiescent to contracting-phase in vitro term not-in-labour myometrium

To investigate whether the observed differences in total protein, phosphorylation, and functional enrichment analysis between the myometrium sampled from women with failed IOL and myometrium sampled from women with previously proven labour to full cervical dilatation could simply be due to changes in the condition of the myometrium between a contracting and non-contracting state, the same analyses were performed to compare the phosphoproteomic profile of the myometrium of the elCS group (term not-in-labour) under the pre-contracting condition with the relaxed and contracted phases during spontaneous contractions. Tables showing those proteins which at least significantly doubled or halved and passed FDR between these comparisons are shown in SI35-37. There were ratio data for the comparison of the proteome and phosphoproteome of myometrium in the spontaneously contracting phase, snap-frozen at peak relaxation (relaxed-phase during spontaneous contractions condition), and the pre-contracting phase (relaxed-phase during spontaneous contractions/pre-contracting), for 7755 proteins, and for 2355 phosphopeptides. Fewer proteins were differentially expressed between the spontaneously contracting phase and pre-contracting phase than the number of proteins differentially expressed when the same analysis was performed to compare the failed IOL group with the elCS group under these conditions (Tables 1 and 3). For example, under the pre-contracting condition, 260 proteins were significantly differentially expressed and passed FDR between the failed IOL and elCS group, compared with only 15 proteins significantly differentially expressed between the relaxed-phase during spontaneous contractions and the pre-contracting conditions, and only one protein between the contracted-phase during spontaneous contractions and the pre-contracting conditions. Differential phosphorylation events were similarly lower (Tables 2 and 4).

**Table 3:** Total number of proteins for which the comparison between the contracted and relaxed phases during spontaneous contractions and the pre-contracting condition (contracted-phase during spontaneous contractions /pre-contracting and relaxed-phase during spontaneous contractions / pre-contracting, respectively) showed at least a two-fold increase or decrease, including whether these changes were statistically significant (p<0.05) and whether they also passed FDR (p<0.05 and q<0.05). SP-CON/PRE = contracted-phase during spontaneous contractions /pre-contracting; SP-REL/PRE = relaxed-phase during spontaneous contractions / pre-contracting.

Experimental condition	Ratio at least doubled			Ratio at least halved		
	Doubled	Significantly doubled (*p* < 0.05)	Significantly doubled (*p* < 0.05) and passed FDR (q < 0.05)	Halved	Significantly halved (*p* < 0.05)	Significantly halved (*p* < 0.05) and passed FDR (q < 0.05)
SP-CON/PRE	100	71	0	153	109	1
SP-REL/PRE	83	61	4	179	157	11

**Table 4:** Total number of phosphopeptides for which the comparison between the contracted and relaxed phases during spontaneous contractions and the pre-contracting condition (contracted-phase during spontaneous contractions /pre-contracting and relaxed-phase during spontaneous contractions / pre-contracting, respectively) showed at least a two-fold increase or decrease, including whether these changes were statistically significant (p<0.05) and whether they also passed FDR (p<0.05 and q<0.05). SP-CON/PRE = contracted-phase during spontaneous contractions /pre-contracting; SP-REL/PRE = relaxed-phase during spontaneous contractions / pre-contracting.

Experimental condition	Ratio at least doubled			Ratio at least halved		
	Doubled	Significantly doubled (*p* < 0.05)	Significantly doubled (*p* < 0.05) and passed FDR (q < 0.05)	Halved	Significantly halved (*p* < 0.05)	Significantly halved (*p* < 0.05) and passed FDR (q < 0.05)
SP-CON/PRE	71	53	0	125	92	2
SP-REL/PRE	54	42	0	65	50	1

No proteins significantly doubled and passed FDR between the contracted-phase during spontaneous contractions condition and the pre-contracting condition, and one protein significantly halved and passed FDR (uncharacterised protein DKFZp686N08224 was 2.97-fold less in the contracted-phase during spontaneous contractions condition phase than the pre-contracting condition). Four proteins were significantly higher in the relaxed-phase during spontaneous contractions condition than in the pre-contracting condition in the elCS group and passed FDR, and 11 proteins were significantly lower and passed FDR. The proteins which were significantly greater were Parvalbumin (PVALB), Heat shock 70 kDa protein B’) (Heat shock protein family A member 6) (HSPA6), DNA repair protein XRCC1 (X-ray repair cross-complementing protein 1) (XRCC1), and GTP binding protein overexpressed in skeletal muscle, isoform CRA_a (GEM). The proteins which were significantly lower were: P2Y purinoceptor 14 (UDP-glucose receptor) (P2RY14), sodium-dependent phosphate transporter 2 (SLC20A2), Transforming growth factor beta-2 proprotein (TGFB2), fibroblast growth factor 1 (FGF1), solute carrier family 16, member 3, isoform CRA_a (SLC16A3), large ribosomal subunit protein eL14 (RPL14), EGF-containing fibulin-like extracellular matrix protein 1 (LTBP1), complement factor H (CFH), and histidine-rich glycoprotein. Two phosphorylation events were differentially expressed between the pre-contracting condition and the contracted-phase during spontaneous contractions condition in the elCS group and passed FDR: phosphorylation of Protein prune homolog 2 (BNIP2 motif-containing molecule at the C-terminal region 1) at Serine 699 and phosphorylation of Monocarboxylate transporter 1 (MCT 1) (Solute carrier family 16 member 1) at Serine 261 and Serine 467 (SI38). One phosphorylation event was differentially expressed between the pre-contracting condition and the relaxed-phase during spontaneous contractions condition and passed FDR, and this matched one of those seen between the pre-contracting condition and the contracted-phase during spontaneous contractions condition comparison: phosphorylation of Protein prune homolog 2 (BNIP2 motif-containing molecule at the C-terminal region 1) at Serine 699 (SI39).

Functional protein enrichment analysis was again performed using the online STRING programme, performing both KEGG pathway enrichment analysis and local network cluster enrichment on all proteins for which there was a log-fold change available for the comparison between the contracted-phase during spontaneous contractions and the pre-contracting conditions and the comparison between the relaxed-phase during spontaneous contractions and the pre-contracting conditions of the elCS group. SI40 and SI41 present the results of this analysis for the former comparison, and SI42-SI43 for the latter. For the comparison between the contracted-phase during spontaneous contractions and pre-contracting conditions, six KEGG pathways passed FDR and 43 network clusters; for the comparison between the relaxed-phase during spontaneous contractions and pre-contracting conditions, four KEGG pathways passed FDR and 45 network clusters. Pathways involving the complement cascades, haemostasis and dissolution of fibrin clot, and thrombosis and anticoagulation pathways were differentially enriched for both comparisons.

### Validation sample

As our sample size is small, we conducted a similar comparison between the relaxed-phase during spontaneous contractions and pre-contracting conditions analysis on myometrial samples collected from three further women at term elective CS for validation of our results. Of note, the phosphoproteomics analysis of these samples was conducted at a different time-point and with less sophisticated analysis than that presented above. There was ratio data for the comparison of the proteome and phosphoproteome of myometrium in the spontaneously contracting phase, snap-frozen at peak relaxation (relaxed-phase during spontaneous contractions), and the pre-contracting condition (REL/PRE), for 9174 proteins, and REL/PRE ratio information for 4600 phosphorylation events. Overall, 178 proteins at least halved between the pre-contracting phase and the relaxed-phase during spontaneous contractions conditions (SI44), and five at least doubled (SI45). These proteins were entered into bioinformatic software programmes including Reactome and String^16^. SI46 shows the 25 pathways identified by Reactome^16^ as being the most overrepresented in proteins which at least halved between the relaxed-phase during spontaneous contractions and the pre-contracting conditions, and SI47 those which at least doubled, indicating the potential pathways which may be occurring in vivo during contractions. These include changes in proteins involved in the complement and coagulation cascades, with 11 of 74 such proteins halving in the relaxed-phase during spontaneous contractions group compared with the pre-contracting group (SI46). The functional enrichment analysis indicated that the most overrepresented pathway was interleukin-4 and interleukin-13 signalling, followed by cytokine signalling in the immune system pathway.

## Discussion

Current hypotheses for the trigger of human parturition include a change in balance from a progesterone-dominant environment to one dominated by oestrogen, as well as activation of inflammatory pathways. Progesterone and oestrogen appear to have antagonistic effects on the myometrium, and previous studies have shown that oestrogen promotes transcription of genes such as *COX-2* and the OXT receptor (*OTr*), which stimulate myometrial activity, while progesterone stimulates those which result in decreased myometrial activity^17^. One suggested mechanism for functional progesterone withdrawal is a change in expression of progesterone and oestrogen receptors on uterine myocytes at the time of labour^18–20^. Moreover, the influence of fetal signals on uterine activation at the onset of labour is poorly understood. Here, we present novel data on protein distribution and phosphorylation in fresh human myometrium. Specifically, our findings indicate that myometrial proteins and phosphorylation events are differentially expressed in women with failed IOL compared with women having an elCS who had previously laboured to full dilatation. This could reflect an altered myometrial phenotype in failed IOL, or failed labour progression; however, these findings will need to be confirmed in comparison to spontaneous labour to gain further insight into the mechanism of human parturition. We also show that there are differences in the phosphoproteome between the pre-contracting and contracting state of human myometrial tissue.

The significant differences observed in the proteome and phosphoproteome between myometrium taken from women with failed IOL and emCS and women with history of vaginal birth at elCS support the hypothesis that failed IOL and failure to progress may be due to a dysfunctional myometrial phenotype, providing clues as to the proteins required for effective labour to full cervical dilatation and vaginal birth. This supports previous findings of a differential transcriptome associated with labour dystocia^10,21^.

The proteins most differentially expressed between the failed IOL group and the elCS group under all five experimental conditions suggest a potential disturbance in pathways required for transition from myometrial quiescence to active contractility. Desmuslin (synemin), a type IV intermediate filament involved in cell-cell junction organisation in cardiomyocytes and cytoskeletal organisation^22^, was found to have increased expression in the failed IOL group when compared with the elCS group. Our group has previously reported that synemin phosphorylation reduced during spontaneous contraction in vitro^6^, and a previous transcriptomic study which compared labouring and non-labouring myometrium (stored in a biobank) (with most (11/19) in the labouring group undergoing emCS for failure to progress), and found significantly lower synemin gene expression in the labouring group^23^. Synemin is known to be highly expressed in adult skeletal and cardiac muscle^22^, however its potential role in myometrial smooth muscle is not yet defined, and these findings suggest that its persistence may contribute to a non-labouring cytoskeletal state. Matrix Gla Protein was also increased in the failed IOL group under all experimental conditions; Matrix Gla Protein regulates vitamin K calcification in vascular smooth muscle and bone^24^. Although there is limited knowledge of the action of Matrix Gla Protein in the myometrium, reduced gene expression has been reported in both term and preterm labour. Further, immunohistochemistry indicates a co-localisation of Matrix Gla Protein with smooth-muscle actin monofilaments^25^, with suggested involvement in Ca ^2+^ transport within intracellular vesicles that may contribute to the switch from myometrial quiescence to contractility in labour^26^.

Further protein differences between the failed IOL and elCS groups point toward a potential role for altered extracellular matrix and signalling pathways in failed IOL. For example, Lysyl oxidase homolog 2 (LOXL2) more than doubled in the failed IOL group under all experimental groups. LOXL2 is a member of the lysine tyrosylquinone (LTQ)-dependent lysyl oxidase (LOX) family, which are copper-dependent amine oxidases^27^. As with other members of the LOX family, LOXL2 is thought to promote stiffening of the extracellular matrix (ECM) through its catalysing of cross-linking of the ECM proteins, including elastin and collagen^27^. LOXL2 is found in many human tissues, with greatest expression in the reproductive organs, including uterus, placenta and prostate. The expression of LOXL2 has also been associated with the upregulation of matrix metalloproteinase-9 and tissue inhibitor of metalloproteinase-1 (TIMP-1), thereby promoting ECM remodelling^27^. Given its known induction by Transforming Growth Factor (TGF)-β in many tissues, it is interesting that we found Transforming Growth Factor-Beta-induced Protein ig-h3 (TGFBI or βig-H3) was also significantly higher in the failed IOL group under all experimental conditions. βig-H3 is an extracellular matrix protein that is induced by TGF-β^28^, and secreted by smooth muscle cells, fibroblasts, and chondrocytes^28,29^. The observed increase in both βig-H3 and LOXL2 in the failed IOL group could therefore be a result of increased TGF-β. In addition, the scaffolding protein A-kinase anchor protein 12 was downregulated in the failed IOL group. These protein changes may have functional implications in labour, due to increased stiffness of the myometrium rendering the smooth muscle less able to coordinate contractions for effective labour. Further, the downregulation of the MHC class I antigens (HLA-A, -B, -C) observed in the failed IOL group may reflect an altered immune-myometrial interaction, diminishing the pro-inflammatory signalling normally required for effective labour onset. MHC class I human leukocyte antigens (HLAs)-A, -B, and -C have been suggested to be important at the maternal-fetal interface, potentially with educating uterine natural killer cells, thought to be important for improved pregnancy outcomes including pre-eclampsia^30^. A previous study investigating genes differentially expressed in labour dystocia also found down-regulation of key regulators of the host immune response, including MHC class I^11^. Likewise, reduction in glutathione S-transferase Mu 1 may represent a deficit in antioxidant defences in the failed IOL group, consistent with a role for oxidative stress in dysfunctional labour^31^.

Administration of an intravenous oxytocin infusion is typically used for inducing contractions during IOL, and therefore it is interesting to observe that the greatest number of proteins that significantly halved and passed FDR in the failed IOL group when compared with the elCS group were under the relaxed- and contracted-phases during OXT-induced contractions. Under both of these conditions, the greatest differences were seen among glutathione S-transferase Mu 1 and MHC class I antigen HLA-A, -B, and -C. These proteins were also differentially expressed under the pre-contracting condition. It is therefore possible that the attenuated immune and antioxidant responses that we have observed may reflect a broader defect in myometrial responsiveness to oxytocin in the failed IOL group, encompassing not only contractile signalling but also the associated immune and oxidative pathways that support effective labour in response to oxytocin^32^.

The phosphoproteomic analysis indicates broad disturbances in cytoskeletal and signalling proteins in the failed IOL group. Most strikingly, there was decreased phosphorylation at S179 of Nexilin (F-actin-binding protein) (Nelin) in the failed IOL group under all five experimental conditions. Nexilin is an actin filament-binding protein which, in cardiomyocytes, localises at Z-discs and help maintain Z-disc stability and protection from mechanical trauma^33,34^. Nexilin has been shown to be phosphorylated by CDC-like kinase 4 (CLK4) in cardiac muscle. Loss of CLK4 reduces Nexilin phosphorylation and results in disrupted cytoskeletal anchoring, sarcomeric instability, and cardiac hypertrophy; restoration of the Nexilin phosphorylation has been shown to rescue the phenotype^35^. Although to our knowledge there are no previously published reports of Nexilin in myometrium, our findings could support a functionally critical role of Nexilin phosphorylation for maintenance of actin-cytoskeletal organisation. Therefore, the reduced phosphorylation observed in the failed IOL group under all five experimental conditions may reflect impaired integrity and contractile dysfunction of a failed IOL myometrium phenotype.

Further phosphorylation differences suggest altered regulation of other actin-associated proteins, including Filamin-A, an actin crosslinker and mechanosensor, which showed increased phosphorylation at two sites under the pre-contracting condition. This is consistent with previous reports that phosphorylation modulates the actin-binding capacity and cytoskeletal organisation of Filamin-A^36^. In addition, phosphorylation of synaptopodin 2, also involved in actin remodelling^37^, was increased in the failed IOL group under the relaxed-phase during OXT-induced contractions. Moreover, the reduction in phosphorylation of both chondroitin sulfate proteoglycan 2 (versican), isoform CRA_c observed in the failed IOL group under the relaxed-phase during OXT-induced contractions, alongside beta-2-syntrophin under the contracted-phase during spontaneous contractions, could indicate dysregulation in extracellular matrix-cytoskeletal coupling, which has been shown to be important for human labour ^38–40^.

The enrichment analyses performed reinforce the phosphoproteomic findings, highlighting differences in both extracellular and metabolic pathways in the failed IOL group, including over-representation of ECM-receptor interaction, which is consistent with the altered extracellular matrix proteins such as versican and SNTB2 described above. In addition, local network clusters identified disruption of the citrate cycle (TCA cycle) and pyruvate metabolism. Collectively, these analyses support a model in which failed IOL is defined by abnormal cytoskeletal remodelling, extracellular matrix dysregulation, and defective metabolic adaptation.

Comparison of the spontaneous contracting phase with the pre-contraction phase in the elCS group was conducted to investigate any differences in the myometrial phosphoproteome between the non-contracting and contracting state, and to determine if all or any of the changes observed in the failed IOL/elCS comparisons above could have been due to changes in contractility states. The data from the quiescent, spontaneous relaxed and spontaneous contracting comparisons showed fewer significant differences than those identified between the failed IOL and elCS groups, which would support interpretation of a specific myometrial phosphoproteome phenotype associated with failed IOL, rather than a reflection of changes simply from the myometrium changing from quiescence to contracting. Enrichment analyses of the proteins differentially expressed identified KEGG pathways and local network clusters involving complement and coagulation cascades, which is consistent with previous studies regarding changing myometrial state from quiescent to contracting phase ^20,23,41^. For example, one study showed an increase in expression of genes coding for inflammatory markers with active labour along with a decrease in genes involved with muscle-specific pathways^23^, and another showed increased transcriptome activity for interleukins and cytokines, as well as those related to apoptosis and cell proliferation^20^. Our results and these genomic studies and proteomic data support the view that complement and inflammatory pathways are important for myometrial contractions. We also identified enrichment of pathways for haemostasis and dissolution of fibrin clot, and thrombosis and anticoagulation pathways, for both the spontaneous relaxed and spontaneous contracting comparisons. This is interesting as previous studies have shown that bleeding activates the clotting cascade which in turn activates thrombin^42^, and bleeding during pregnancy can be associated with preterm labour and an “irritable” uterus^43,44^. Thrombin stimulates myometrial contractions via activation of the phosphatidyl-inositol signalling pathway and subsequent influx of calcium ions into the cytosol both in vitro^45^ and in vivo^45,46^; thrombin also stimulates matrix metalloproteinase-1 production and may be involved in premature rupture of membranes in women^47^.

We acknowledge that our numbers are small and therefore performed a validation test of the comparison between the relaxed-phase during spontaneous contractions and the pre-contracting condition on three samples of term not-in-labour myometrium. Results of the enrichment analysis of these samples were similar to those of the comparison between the relaxed-phase during spontaneous contractions and pre-contracting conditions of the elCS sample, including increased enrichment of scavenging of heme from plasma, regulation of complement cascade, and complement cascade pathways. The results of the enrichment analysis of these samples were therefore similar to those of the elCS analysis of the main study, supporting its validity.

Further limitations to this study include differences in mean gestational age and labour status between the groups. The difference in gestational age between groups was expected as elective CSs were performed before 40 weeks’ gestation, whereas inductions for post-term pregnancy were routinely commenced at 40 + 12 days at St Michael’s Hospital and this difference, although small, could have accounted for some of our observed differences. There is limited evidence comparing myometrial contractility between early- and late-term myometrium, existing studies suggest a gradual increase in myometrial excitability, oxytocin receptor density, and connexin-43 expression towards term^48,49^. As stated above, a clearer understanding would be gained by comparing the phosphoproteome of myometrium obtained during emergency CS following spontaneous labour with emCS after IOL; however, obtaining such samples is ethically and practically challenging, as consent would need to be obtained in advance of labour. It is also possible that failure to progress in labour could reflect defective cervical softening and dilatation rather than solely myometrial dysfunction^50–52^. Although the cervix was not sampled here, its role in successful labour is of major importance and warrants further investigation. The proteomic changes we have identified here involve pathways regulating myometrial excitability and contraction, supporting a myometrial contribution to the phenotype of failure to progress in labour. While phosphorylation events occur rapidly, some changes in total protein abundance may represent sustained activation or degradation processes secondary to contraction signalling. Despite its clinical limitations, this study provides the first functional analysis of the total proteome and phosphoproteome of human myometrium in late pregnancy and during spontaneous and oxytocin-induced contractions and validates the proteomics methodology for human samples.

## Conclusions

This study provides a wealth of novel proteomic and phosphoproteomics data, identifying proteins and phosphorylation events in fresh human myometrium under carefully controlled conditions. Overall, our data support the hypothesis that there is a myometrial phenotype associated with failed IOL due to failure to progress in labour. The main differences occurred in proteins from pathways involving cytoskeletal remodelling, extracellular matrix dysregulation, and defective metabolic adaptation. These data should encourage further research into the role of these proteins and the alterations associated with dysfunctional myometrium. At present it is difficult to conclusively determine whether the apparent changes in myometrial phenotype are due to intrinsic physiopathological changes in the myometrium prior to IOL, or a consequence of tissue stress from difficult and prolonged IOL, and further research is necessary to compare IOL with spontaneous labour in a larger cohort of women. This research would be challenging to conduct without new methods to measure uterine function at a molecular level in a non-invasive manner. Further studies are warranted combining contractility measurements with functional molecular studies focusing on the pathways and proteins we have highlighted. These results show that myometrial phosphoproteomic approaches are a powerful resource to understand the complex process of human labour.

## Methods

### Clinical groups

These experiments were conducted on fresh strips of myometrium obtained from women undergoing caesarean section. We measured contractility and compared the phosphoproteome of the tissue during contraction and relaxation.

### Tissue collection

For each woman, a myometrial sample was obtained at the time of caesarean section, following birth of the baby and placenta, as previously described^6,8^. Samples were transported to the laboratory for immediate processing. With minimal handling of the myometrium, and while keeping the tissue submerged in isotonic saline, the samples were cut into five strips of approximately 5 mm by 20 mm dimensions with a scalpel^8^.

### Measurement of myometrial contractility

Contractility measurements were conducted using a four chamber Myobath-II system (World Precision Instruments (WPI), Stevenage, UK), as previously described^6,8^. Four of the cut myometrial strips were mounted using s-shaped hooks, and one was individually fixed under tension in each of the four 10 ml chambers of the Myobath-II system (SI48A). A fifth strip was connected to the upper s-hook of the first bath to provide a pre-contracting control. The strips were equilibrated in oxygenated Krebs solution at 37 degrees Celsius for 45 min. The s-hooks attached at either end of the four myometrial strips were connected to force transducers and data collected using Lab-Trax data acquisition system (WPI, Stevenage, UK)^8^. We have previously reported on the differences in tension induced under the different experimental conditions (described below), which illustrated that oxytocin-induced contractions generated more force than spontaneous contractions^6^.

### Experimental conditions

In each case, the four myometrial strips under tension were either allowed to continue to contract spontaneously or treated with 10 nM oxytocin (OXT) (Merck, Darmstadt, Germany). Approximately 40 min after OXT was or was not added, the strips from all four baths were snap frozen at either maximal relaxation or maximal contraction, alongside the control strip not under tension, as previously described^8^. The following five conditions were assessed (SI48):

1. No tension applied; considered pre-contraction (PRE).
2. Spontaneous contraction (no OXT) snap frozen 20 s following the end of a phasic contraction (SP-REL).
3. Spontaneous contraction (no OXT) snap frozen at peak phasic contraction (SP-CON).
4. Oxytocin-stimulated phasic contraction snap frozen 20 s following the end of a phasic contraction (OXT-REL).
5. Oxytocin-stimulated contraction (with OXT) snap frozen at peak phasic contraction (OXT-CON).

Samples were then stored at -80 degrees Celsius until protein extraction was performed.

### Tissue homogenisation

Myometrial strips were homogenised in radioimmunoprecipitation assay (RIPA) buffer at approximately 100 mg of wet tissue per millilitre of buffer, using a Polytron homogeniser, keeping everything on ice. The RIPA buffer contained 1% NP-40, 0.1% sodium dodecyl sulphate (SDS), 0.5% sodium deoxycholate, and phosphatase and protease inhibitors (PhoSTOP, cOmplete) (Roche Diagnostics Limited, Burgess Hill, UK). The lysates were cleared through centrifugation for ten minutes at 16000 g at 4 degrees Celsius. The BCA assay kit (Perbio Science UK, Cramlington, UK) was used to adjust the protein concentrations to 2 mg per millilitre; 100 µg of protein was then used for the phosphoproteomics analysis^6^.

### Tandem mass tag labelling, high pH reversed-phase chromatography and phosphopeptide enrichment

Aliquots of 100 µg of each sample were digested with trypsin (2.5 µg trypsin; 37 °C, overnight), and labelled with Tandem Mass Tag (TMT) ten plex reagents, according to manufacturer’s protocol (Thermo Fisher Scientific, Loughborough, UK), and the labelled samples pooled. For Total proteome analysis, an aliquot of 50ug of the pooled sample was desalted using a SepPak cartridge, per manufacturer’s instructions (Waters, Milford, Massachusetts, USA), and the eluate evaporated to dryness and resuspended in buffer A (20 mM ammonium hydroxide, pH 10) prior to fractionation by high pH reversed-phase chromatography using an Ultimate 3000 liquid chromatography system (Thermo Fisher Scientific). In brief, the sample was loaded onto an XBridge BEH C18 Column (130Å, 3.5 μm, 2.1 mm X 150 mm, Waters, UK) in buffer A and peptides eluted with an increasing gradient of buffer B (20 mM Ammonium Hydroxide in acetonitrile, pH 10) from 0 to 95% over 60 min. The resulting fractions (concatenated into 15 in total) were evaporated to dryness and resuspended in 1% formic acid prior to analysis by nano-LC MSMS, using an Orbitrap Fusion Tribrid mass spectrometer (Thermo Scientific).

For Phosphoproteome analysis, the remainder of the TMT-labelled pooled sample was also desalted using a SepPak cartridge (Waters, Milford, Massachusetts, USA), and the eluate evaporated to dryness and subjected to TiO2-based phosphopeptide enrichment, according to the manufacturer’s instructions (Pierce). The phospho-enriched sample was again evaporated to dryness and resuspended in 1% formic acid prior to analysis by nano-LC MSMS, using an Orbitrap Fusion Tribrid mass spectrometer (Thermo Scientific).

### Nano-LC mass spectrometry

High pH RP fractions (Total proteome analysis) or the phospho-enriched peptides (Phosphoproteome analysis) were further fractionated using an Ultimate 3000 nano-LC system in line with an Orbitrap Fusion Tribrid mass spectrometer (Thermo Scientific). In brief, peptides in 1% (vol/vol) formic acid were injected onto an Acclaim PepMap C18 nano-trap column (Thermo Scientific). After washing with 0.5% (vol/vol) acetonitrile 0.1% (vol/vol) formic acid peptides were resolved on a 250 mm × 75 μm Acclaim PepMap C18 reverse phase analytical column (Thermo Scientific) over a 150 min organic gradient, using seven gradient segments (1–6% solvent B over 1 min., 6–15% B over 58 min., 15–32%B over 58 min., 32–40%B over 5 min., 40–90%B over 1 min., held at 90%B for 6 min and then reduced to 1%B over 1 min.) with a flow rate of 300 nl min^− 1^. Solvent A was 0.1% formic acid and Solvent B was aqueous 80% acetonitrile in 0.1% formic acid. Peptides were ionized by nano-electrospray ionization at 2.0 kV using a stainless-steel emitter with an internal diameter of 30 μm (Thermo Scientific) and a capillary temperature of 275 °C. All spectra were acquired using an Orbitrap Fusion Tribrid mass spectrometer controlled by Xcalibur 2.1 software (Thermo Scientific) and operated in data-dependent acquisition mode using an SPS-MS3 workflow. FTMS1 spectra were collected at a resolution of 120 000, with an automatic gain control (AGC) target of 200 000 and a max injection time of 50ms. Precursors were filtered with an intensity threshold of 5000, according to charge state (to include charge states 2–7) and with monoisotopic peak determination set to peptide. Previously interrogated precursors were excluded using a dynamic window (60s +/-10ppm). The MS2 precursors were isolated with a quadrupole isolation window of 1.2 m/z. ITMS2 spectra were collected with an AGC target of 10 000, max injection time of 70ms and CID collision energy of 35%. For FTMS3 analysis, the Orbitrap was operated at 50 000 resolution with an AGC target of 50 000 and a max injection time of 105ms. Precursors were fragmented by high energy collision dissociation (HCD) at a normalised collision energy of 60% to ensure maximal TMT reporter ion yield. Synchronous Precursor Selection (SPS) was enabled to include up to 5 MS2 fragment ions in the FTMS3 scan.

### Analysis

The flow diagram in SI49 presents an overview of the proteomic and phosphoproteomic analysis performed for the failed IOL vs. elCS comparisons, and SI50 for the validation study (comparison between the relaxed-phase during spontaneous contractions and pre-contracting conditions only). Briefly, Proteome Discoverer software v2.1 (Thermo Scientific) was used to process and quantify the raw data files. A SEQUEST algorithm search was performed against the UniProt Human database (retrieved 01-2018, https://www.uniprot.org/) as well as a Common Contaminants database^53,54^. Any peptides that were found exclusively in the contaminants database were excluded. For global total proteomics analysis, results were filtered using a 5% false discovery rate (FDR) cut-off and normalised on total peptide for each sample. TMT tags allowed the abundance of individual peptides in each sample to be reported. The identified list of phosphopeptides was matched with the corresponding proteins in the total dataset, for ease of comparison. As both phosphopeptide and total protein samples came from the same pool of TMT labelled peptides, the phosphopeptide abundances were normalised using the total protein normalisation factors then adjusted to changes in total protein abundances, facilitating the identification of changes in the relative phosphorylation of a protein.

The following comparisons were calculated:

1. To investigate potential differences in the phosphoproteome of the myometrium from women who had a failed IOL and those with previously proven labour to full dilatation, comparison between the mean of the failed IOL group compared with the mean of the elCS group (failed IOL/elCS) for each of the different experimental conditions (pre-contracting, relaxed-phase during spontaneous contractions, the contracted-phase during spontaneous contractions condition, the relaxed-phase during OXT-induced contractions condition, the contracted-phase during OXT-induced contractions condition);
2. To identify potential proteins and phosphorylations which changed between the non-contracting and the spontaneously contracting state of myometrium, comparisons were made between the contracted-phase during spontaneous contractions and pre-contracting conditions, and between the relaxed-phase during spontaneous contractions and pre-contracting conditions. These comparisons were investigated elucidate which protein changes may be the effects of active contraction, as the pre-contracting myometrium is in a relaxed state, to gain more insight into the differences between the pre-contracting state and spontaneously contracting state both within (the contracted-phase during spontaneous contractions) and outside (relaxed-phase during spontaneous contractions ) of an active contraction.

Differential abundance analysis was performed using the limma package^55^, which implements linear modelling for each identified protein combined with empirical Bayes variance moderation in order to stabilise variance estimates and increase statistical power in smaller-sample, high-dimensional datasets, such as this one. Within each experimental condition, we applied limma to perform moderated t-tests between the IOL and elCS groups. The choice of limma is supported by recent benchmarking demonstrating its robust and reproducible performance across diverse proteomics workflows^56^.

To identify biologically relevant pathways and protein networks among the differentially expressed proteins, functional protein enrichment analysis was performed using the STRING database (https://string-db.org). Protein accession codes and corresponding logFC values were uploaded to STRING, with accession codes used for protein mapping and logFC values included to visualise the direction and magnitude of expression changes. This was undertaken for data generated under each of the five conditions (pre-contracting, relaxed-phase during spontaneous contractions, contracted-phase during spontaneous contractions, relaxed-phase during OXT-induced contractions, and the contracted-phase during OXT-induced contractions) for the comparisons of failed IOL with elCS, and also for the REL/PRE analyses. Two key outputs were generated: (1) KEGG pathway enrichment, which identifies statistically overrepresented pathways using a hypergeometric test. Enrichment scores are reported as -log10(FDR), with higher values indicating greater statistical significance; and (2) Local network cluster enrichment, which detects densely connected regions within the STRING protein-protein interaction network, representing potential functional modules. An FDR threshold of ≤ 0.05 was applied to determine significance. These analyses provide insights into the biological processes and molecular mechanisms potentially underlying the observed protein expression patterns.

## Supplementary Information

Below is the link to the electronic supplementary material.

Supplementary Material 1

## Data Availability

All data generated or analysed during this study are included in this published article and its Supplementary Information files.
