# Supplementary material for: Failed induction of human labour is associated with an altered myometrial phosphoproteome
Source: Sci Rep. 2025 Dec 1;15:44081. doi: 10.1038/s41598-025-27605-6 (PMC12715255; doi:10.1038/s41598-025-27605-6)

Supplementary Information 1: Maternal demographics for failed induction of labour vs elective caesarean section study

| **Age at Recruitment (years)** | **30^a^**  (26-34) | **36.7 ^a^**  (34-40) | **NS** |
| --- | --- | --- | --- |
| **Body Mass Index** | **24.6 ^a^** | **26.2^a^** | **NS** |
|  | (20-32.7) | (20-29.2) |  |
| **Gravida** | **1^b^** | **3 ^b^** | **NS** |
|  | (1-3) | (2-4) |  |
| **Parity** | **0 ^b^** | **1 ^b^** |  |
|  | (0) | (1) |  |
| **Alcohol during pregnancy** | **NIL** | **NIL** | **NA** |
| **Ethnicity** | **ALL WE** | **ALL WE** | **NA** |
| **Smoking during pregnancy** | **NIL** | **2 NIL** | **NS** |
|  |  | **1 3/day** |  |
| **Indication for induction** | **Post-term** | **NA** | **NA** |
| **Indication for elective CS** | **NA** | **1 PP**  **1 MR**  **1 PTDT** | **NA** |
| **Uterotonic given at delivery** | **3 Carbetocin** | **3 Carbetocin** | **NA** |
| **Gestational age at birth** | **293.3 ^b^**  (292-295) | **268.7 ^b^**  (258-272) | **0.006** |
| **Maternal complications** | **NIL** | **NIL** | **NA** |
| **Birthweight** | **3.7 ^a^** | **3.4 ^a^** | **NS** |
|  | (2.44-4.18) | (3.3-3.6) |  |
| **Baby sex** | **3 M** | **1 F**  **2 M** | **NA** |

^a^Mean ; ^b^Median; CS=Caesarean Section; WE=White European; PP = Placenta Praevia; MR=Maternal Request; PTDT=Previous Third-Degree Tear; F=Female; M=Male

**Supplementary Information 2:** Total proteins which significantly (p≤0.05) at least doubled and passed FDR (q≤0.05) in the failed IOL group compared with the elCS group under the pre-contracting condition, ordered by log-fold change.

| **Protein name** | **Accession** | **Gene name** | **Log Fold-change** | **p-value** | **q-value** | **Phosphorylation identified** |
| --- | --- | --- | --- | --- | --- | --- |
| **Parvalbumin** | H0Y3U0 | PVALB | 6.50 | <0.001 | 0.020 | NO |
| **Desmuslin, isoform CRA_a (Synemin)** | A0A075B7B1 | SYNM | 6.05 | <0.001 | 0.008 | NO |
| **Calpain-2 catalytic subunit (EC 3.4.22.53) (Calcium-activated neutral proteinase 2) (Calpain M-type) (Calpain-2 large subunit) (Millimolar-calpain)** | B2RCM3 | NA | 4.48 | <0.001 | 0.002 | NO |
| **maleylacetoacetate isomerase (EC 5.2.1.2)** | G3V4T6 | GSTZ1 | 4.47 | <0.001 | <0.001 | NO |
| **Choline transporter-like protein** | A0A088QCU6 | SLC44A2 | 4.37 | 0.001 | 0.043 | NO |
| **Ankyrin-3 (ANK-3) (Ankyrin-G)** | Q12955 | ANK3 | 4.25 | <0.001 | 0.038 | NO |
| **Zinc finger, UBR1 type 1, isoform CRA_c** | A0A024RAC9 | ZUBR1 | 3.93 | <0.001 | 0.006 | NO |
| **Large ribosomal subunit protein eL14 (60S ribosomal protein L14)** | Q6IPH7 | RPL14 | 3.82 | <0.001 | <0.001 | NO |
| **Heat shock 70 kDa protein 6 (Heat shock 70 kDa protein B') (Heat shock protein family A member 6)** | P17066 | HSPA6 | 3.57 | <0.001 | 0.002 | NO |
| **Carbonic anhydrase III, muscle specific, isoform CRA_a** | A0A024R825 | CA3 | 3.51 | <0.001 | 0.005 | NO |
| **DNA repair protein XRCC1 (X-ray repair cross-complementing protein 1)** | P18887 | XRCC1 | 3.30 | <0.001 | 0.012 | NO |
| **Immunoglobulin heavy constant gamma 4 (Ig gamma-4 chain C region)** | P01861 | IGHG4 | 3.27 | <0.001 | 0.013 | NO |
| **Matrix Gla protein (MGP) (Cell growth-inhibiting gene 36 protein)** | P08493 | MGP | 3.25 | <0.001 | <0.001 | NO |
| **Cartilage oligomeric matrix protein (COMP) (Thrombospondin-5) (TSP5)** | P49747 | COMP | 3.11 | <0.001 | 0.028 | NO |
| **Matrilin 2, isoform CRA_b** | A0A024R9B9 | MATN2 | 3.06 | <0.001 | 0.017 | NO |
| **Thrombospondin-1 (Glycoprotein G)** | P07996 | THBS1 | 2.99 | <0.001 | 0.037 | NO |
| **Annexin A3 (35-alpha calcimedin) (Annexin III) (Annexin-3) (Inositol 1,2-cyclic phosphate 2-phosphohydrolase) (Lipocortin III) (Placental anticoagulant protein III) (PAP-III)** | P12429 | ANXA3 | 2.67 | <0.001 | 0.003 | NO |
| **Low affinity immunoglobulin gamma Fc region receptor III-B (Fc-gamma RIII-beta) (CD16-I) (Fc-gamma RIII) (Fc-gamma RIIIb) (FcRIII) (FcRIIIb) (FcR-10) (IgG Fc receptor III-1) (CD antigen CD16b)** | O75015 | FCGR3B | 2.66 | <0.001 | 0.034 | NO |
| **Keratin, type II cytoskeletal 5 (Cytokeratin-5) (CK-5) (Keratin-5) (K5) (Type-II keratin Kb5)** | Q5XQN5 | KRT5 | 2.65 | <0.001 | 0.037 | NO |
| **Apolipoprotein(a) (Apo(a)) (Lp(a)) (EC 3.4.21.-)** | P08519 | LPA | 2.54 | <0.001 | 0.022 | NO |
| **N90-VRC38.08 heavy chain variable region** | A0A1W6IYI5 | NA | 2.48 | <0.001 | 0.009 | NO |
| **Melanoma-associated antigen C3 (Cancer/testis antigen 7.2) (CT7.2) (Hepatocellular carcinoma-associated antigen 2) (MAGE-C3 antigen)** | Q8TD91 | MAGEC3 | 2.45 | <0.001 | 0.038 | NO |
| **IGH@ protein** | Q6P089 | IGH@ | 2.43 | <0.001 | 0.021 | NO |
| **Thrombospondin-4** | P35443 | THBS4 | 2.38 | <0.001 | 0.025 | NO |
| **Methylthioribulose-1-phosphate dehydratase (MTRu-1-P dehydratase) (EC 4.2.1.109) (APAF1-interacting protein) (hAPIP)** | Q96GX9 | APIP | 2.35 | <0.001 | 0.038 | NO |
| **MHC class II antigen** | A0A1Y6MRR4 | HLA-DPB1 | 2.30 | <0.001 | 0.024 | NO |
| **Keratin, type I cytoskeletal 9 (Cytokeratin-9) (CK-9) (Keratin-9) (K9)** | P35527 | KRT9 | 2.28 | <0.001 | 0.030 | NO |
| **Alpha-1-acid glycoprotein 2 (AGP 2) (Orosomucoid-2) (OMD 2)** | P19652 | ORM2 | 2.23 | 0.001 | 0.048 | NO |
| **cDNA FLJ54808, highly similar to TBC1 domain family member 13** | B4DHM6 | NA | 2.17 | <0.001 | 0.034 | NO |
| **Keratin, type II cytoskeletal 1 (Cytokeratin-1) (Keratin-1) (Type-II keratin Kb1)** | H6VRG2 | KRT1 | 2.15 | 0.001 | 0.048 | NO |
| **Thrombospondin 1, isoform CRA_a** | A0A024R9Q1 | THBS1 | 2.14 | <0.001 | 0.020 | NO |
| **CCN family member 4 (WNT1-inducible-signaling pathway protein 1) (WISP-1) (Wnt-1-induced secreted protein)** | O95388 | CCN4 | 2.12 | <0.001 | 0.001 | NO |
| **Prostaglandin G/H synthase 1 (EC 1.14.99.1) (Cyclooxygenase-1) (COX-1) (Prostaglandin H2 synthase 1) (PGH synthase 1) (PGHS-1) (PHS 1) (Prostaglandin-endoperoxide synthase 1)** | P23219 | PTGS1 | 2.07 | <0.001 | 0.002 | NO |
| **FXYD domain containing ion transport regulator 6, isoform CRA_a** | A0A024R3J8 | FXYD6 | 2.05 | <0.001 | 0.007 | NO |
| **Myosin-reactive immunoglobulin heavy chain variable region** | Q9UL90 | NA | 2.04 | <0.001 | 0.022 | NO |
| **Serpin peptidase inhibitor, clade E (Nexin, plasminogen activator inhibitor type 1), member 1, isoform CRA_b** | A0A024QYT5 | SERPINE1 | 1.99 | <0.001 | 0.013 | NO |
| **Haptoglobin (Zonulin) [Cleaved into: Haptoglobin alpha chain; Haptoglobin beta chain]** | P00738 | HP | 1.96 | <0.001 | 0.013 | NO |
| **Protein bicaudal D homolog 1 (Bic-D 1)** | Q96G01 | BICD1 | 1.96 | <0.001 | 0.012 | NO |
| **Lectin, galactoside-binding, soluble, 1** | A0A0B6XK00 | LGALS1 | 1.95 | <0.001 | 0.031 | NO |
| **Neural cell adhesion molecule 1** | A0A087WTF6 | NCAM1 | 1.94 | <0.001 | 0.001 | NO |
| **Unconventional myosin-If (Myosin-Ie)** | O00160 | MYO1F | 1.91 | 0.001 | 0.048 | NO |
| **Alkaline phosphatase, tissue-nonspecific isozyme (AP-TNAP) (TNS-ALP) (TNSALP) (EC 3.1.3.1) (Alkaline phosphatase liver/bone/kidney isozyme) (Phosphoamidase) (Phosphocreatine phosphatase) (EC 3.9.1.1)** | P05186 | ALPL | 1.85 | <0.001 | 0.004 | NO |
| **Lysyl oxidase homolog 2 (EC 1.4.3.13) (Lysyl oxidase-like protein 2) (Lysyl oxidase-related protein 2) (Lysyl oxidase-related protein WS9-14)** | Q9Y4K0 | LOXL2 | 1.77 | <0.001 | 0.002 | NO |
| **TNC variant protein** | Q4LE33 | TNC variant protein | 1.76 | <0.001 | 0.016 | NO |
| **Uncharacterized protein DKFZp686K18196** | Q6N092 | DKFZp686K18196 | 1.76 | 0.001 | 0.046 | NO |
| **Diphosphoinositol polyphosphate phosphohydrolase 3-alpha (DIPP-3-alpha) (DIPP3-alpha) (hDIPP3alpha) (EC 3.6.1.52) (Diadenosine 5',5'''-P1,P6-hexaphosphate hydrolase 3-alpha) (Diadenosine hexaphosphate hydrolase (AMP-forming)) (EC 3.6.1.60) (Nucleoside diphosphate-linked moiety X motif 10) (Nudix motif 10) (hAps2)** | Q8NFP7 | NUDT10 | 1.74 | <0.001 | 0.021 | NO |
| **Collagen alpha-1(XII) chain** | Q99715 | COL12A1 | 1.74 | 0.001 | 0.038 | NO |
| **Vitamin D binding protein** | A0A1B1CYC5 | Gc | 1.71 | <0.001 | 0.007 | NO |
| **Probable non-functional immunoglobulin heavy variable 3-35** | A0A0C4DH35 | IGHV3-35 | 1.69 | 0.002 | 0.049 | NO |
| **cDNA FLJ51896, highly similar to Glia-derived nexin** | B4DMR3 | NA | 1.68 | <0.001 | 0.022 | NO |
| **SAA2-SAA4 readthrough** | A0A096LPE2 | SAA2-SAA4 | 1.66 | <0.001 | 0.004 | NO |
| **Matrilin-2** | O00339 | MATN2 | 1.65 | <0.001 | 0.022 | NO |
| **Secreted frizzled-related protein 1 (FRP-1) (sFRP-1) (Secreted apoptosis-related protein 2) (SARP-2)** | Q8N474 | SFRP1 | 1.60 | <0.001 | 0.012 | NO |
| **Plasma kallikrein (EC 3.4.21.34) (Fletcher factor) (Kininogenin) (Plasma prekallikrein) (PKK) [Cleaved into: Plasma kallikrein heavy chain; Plasma kallikrein light chain]** | P03952 | KLKB1 | 1.60 | <0.001 | 0.031 | NO |
| **Lipopolysaccharide-binding protein (LBP)** | P18428 | LBP | 1.57 | <0.001 | 0.034 | NO |
| **C14orf159 variant protein** | Q4LE40 | C14orf159 variant protein | 1.54 | <0.001 | <0.001 | NO |
| **Plasma protease C1 inhibitor (C1 Inh) (C1Inh) (C1 esterase inhibitor) (C1-inhibiting factor) (Serpin G1)** | P05155 | SERPING1 | 1.52 | <0.001 | 0.028 | NO |
| **Polypeptide N-acetylgalactosaminyltransferase 18 (EC 2.4.1.41) (Polypeptide GalNAc transferase 18) (GalNAc-T18) (Polypeptide GalNAc transferase-like protein 4) (GalNAc-T-like protein 4) (pp-GaNTase-like protein 4) (Polypeptide N-acetylgalactosaminyltransferase-like protein 4) (Protein-UDP acetylgalactosaminyltransferase-like protein 4) (UDP-GalNAc:polypeptide N-acetylgalactosaminyltransferase-like protein 4)** | Q6P9A2 | GALNT18 | 1.48 | 0.001 | 0.039 | NO |
| **SH3 domain-binding glutamic acid-rich-like protein 2 (Fovea-associated SH3 domain-binding protein)** | Q9UJC5 | SH3BGRL2 | 1.47 | <0.001 | 0.005 | NO |
| **Conserved oligomeric Golgi complex subunit 2 (COG complex subunit 2) (Component of oligomeric Golgi complex 2) (Low density lipoprotein receptor defect C-complementing protein)** | Q14746 | COG2 | 1.45 | <0.001 | 0.021 | NO |
| **Solute carrier family 2, facilitated glucose transporter member 4 (Glucose transporter type 4, insulin-responsive) (GLUT-4)** | P14672 | SLC2A4 | 1.44 | <0.001 | 0.023 | NO |
| **Transforming growth factor-beta-induced protein ig-h3 (Beta ig-h3) (Kerato-epithelin) (RGD-containing collagen-associated protein) (RGD-CAP)** | Q15582 | TGFBI | 1.37 | <0.001 | 0.034 | NO |
| **Dihydropyrimidinase-related protein 1 (DRP-1) (Collapsin response mediator protein 1) (CRMP-1) (Inactive dihydropyrimidinase) (Unc-33-like phosphoprotein 3) (ULIP-3)** | Q14194 | CRMP1 | 1.35 | 0.001 | 0.038 | NO |
| **Pregnancy zone protein (C3 and PZP-like alpha-2-macroglobulin domain-containing protein 6)** | P20742 | PZP | 1.35 | 0.001 | 0.048 | NO |
| **Tenascin-X (TN-X) (Hexabrachion-like protein)** | P22105 | TNXB | 1.34 | <0.001 | 0.018 | NO |
| **Alpha-2-macroglobulin (Alpha-2-M) (C3 and PZP-like alpha-2-macroglobulin domain-containing protein 5)** | P01023 | A2M | 1.34 | <0.001 | 0.030 | NO |
| **Sacsin (DnaJ homolog subfamily C member 29)** | Q9NZJ4 | SACS | 1.25 | <0.001 | 0.034 | NO |
| **Uncharacterized protein DKFZp686O1553** | Q5HYM1 | DKFZp686O1553 | 1.17 | <0.001 | 0.013 | NO |
| **Procollagen-lysine,2-oxoglutarate 5-dioxygenase 2 (EC 1.14.11.4) (Lysyl hydroxylase 2) (LH2)** | O00469 | PLOD2 | 1.15 | 0.001 | 0.042 | NO |
| **Myosin-1 (Myosin heavy chain 1) (Myosin heavy chain 2x) (MyHC-2x) (Myosin heavy chain IIx/d) (MyHC-IIx/d) (Myosin heavy chain, skeletal muscle, adult 1)** | P12882 | MYH1 | 1.15 | <0.001 | 0.013 | NO |
| **Heparin cofactor 2 (Heparin cofactor II) (HC-II) (Protease inhibitor leuserpin-2) (HLS2) (Serpin D1)** | P05546 | SERPIND1 | 1.15 | <0.001 | 0.020 | NO |
| **cDNA FLJ52761, highly similar to Actin, aortic smooth muscle** | B4DUI8 | NA | 1.10 | <0.001 | 0.013 | NO |
| **Prostacyclin synthase (EC 5.3.99.4) (Hydroperoxy icosatetraenoate dehydratase) (EC 4.2.1.152) (Prostaglandin I2 synthase)** | Q16647 | PTGIS | 1.04 | <0.001 | 0.037 | NO |

**Supplementary Information 3:** Total proteins which significantly (p≤0.05) at least halved and passed FDR (q≤0.05) in the failed IOL group compared with the elCS group under the pre-contracting condition, ordered by log-fold change.

|  |  |  |  |  |  |  |
| --- | --- | --- | --- | --- | --- | --- |
| **Protein name** | **Accession** | **Gene name** | **Log Fold-change** | **p-value** | **q-value** | **Phosphorylation identified** |
| **Glutathione S-transferase Mu 1 (EC 2.5.1.18) (GST HB subunit 4) (GST class-mu 1) (GSTM1-1) (GSTM1a-1a) (GSTM1b-1b) (GTH4)** | P09488 | GSTM1 | -6.66 | <0.001 | <0.001 | NO |
| **Angiotensin-converting enzyme 2 (EC 3.4.17.23) (Angiotensin-converting enzyme homolog) (ACEH) (Angiotensin-converting enzyme-related carboxypeptidase) (ACE-related carboxypeptidase) (EC 3.4.17.-) (Metalloprotease MPROT15) [Cleaved into: Processed angiotensin-converting enzyme 2]** | Q9BYF1 | ACE2 | -6.57 | <0.001 | 0.003 | NO |
| **cDNA FLJ39696 fis, clone SMINT2011033, highly similar to Sorting and assembly machinery component 50 homolog** | B3KUE6 |  | -5.99 | <0.001 | 0.020 | NO |
| **MHC class I antigen** | A0A1X9I3T8 | HLA-A | -5.83 | <0.001 | <0.001 | NO |
| **MHC class I antigen** | A0A1X9I444 | HLA-B | -5.80 | <0.001 | <0.001 | NO |
| **Endoplasmic reticulum DnaJ/PDI fusion protein 3** | Q6YPB0 | NA | -4.63 | <0.001 | 0.003 | NO |
| **RRBP1 protein** | A1A5C4 | RRBP1 | -4.14 | <0.001 | <0.001 | NO |
| **Mitogen-activated protein kinase 5 (EC 2.7.11.25) (Apoptosis signal-regulating kinase 1) (ASK-1) (MAPK/ERK kinase kinase 5) (MEK kinase 5) (MEKK 5)** | Q99683 | MAP3K5 | -3.75 | 0.002 | 0.050 | NO |
| **Ig-like domain-containing protein** | B7ZW57 | NA | -3.44 | <0.001 | 0.010 | NO |
| **Elongator complex protein 5 (Dermal papilla-derived protein 6) (S-phase 2 protein)** | Q8TE02 | ELP5 | -3.33 | <0.001 | 0.018 | NO |
| **MHC class I antigen** | A0A0S4T3R3 | HLA-C | -3.12 | <0.001 | 0.013 | YES |
| **Endoplasmic reticulum aminopeptidase 2 (EC 3.4.11.-) (Leukocyte-derived arginine aminopeptidase) (L-RAP)** | Q6P179 | ERAP2 | -2.80 | <0.001 | <0.001 | NO |
| **C-X-C motif chemokine** | C9J4T6 | CXCL8 | -2.67 | <0.001 | 0.020 | NO |
| **LIM domain binding 3 isoform 1 (LIM domain binding 3, isoform CRA_h)** | A0A0S2Z530 | LDB3 | -2.56 | 0.001 | 0.038 | NO |
| **GTP-binding protein RAD (RAD1) (Ras associated with diabetes)** | P55042 | RRAD | -2.54 | <0.001 | 0.013 | YES |
| **Cytochrome b** | Q6VHB3 | CYTB | -2.48 | <0.001 | 0.034 | NO |
| **Allograft inflammatory factor 1-like (Ionized calcium-binding adapter molecule 2)** | Q9BQI0 | AIF1L | -2.47 | <0.001 | 0.030 | NO |
| **Aminopeptidase N (EC 3.4.11.2)** | B4DPH5 | NA | -2.45 | <0.001 | 0.037 | NO |
| **Leukocyte immunoglobulin-like receptor subfamily B member 5 (CD85 antigen-like family member C) (Leukocyte immunoglobulin-like receptor 8) (LIR-8) (CD antigen CD85c)** | O75023 | LILRB5 | -2.40 | <0.001 | <0.001 | NO |
| **Glutathione S-transferase theta-2B (EC 2.5.1.18) (Glutathione S-transferase theta-2) (GST class-theta-2)** | P0CG30 | GSTT2B | -2.37 | <0.001 | 0.007 | NO |
| **Tubulin polymerization-promoting protein family member 3 (TPPP/p20)** | Q9BW30 | TPPP3 | -2.29 | <0.001 | 0.022 | NO |
| **NA** | P01889 | NA | -2.28 | <0.001 | 0.021 | NO |
| **Glutathione S-transferase Mu 4 (EC 2.5.1.18) (GST class-mu 4) (GST-Mu2) (GSTM4-4) (Leukotriene C4 synthase GSTM4) (EC 4.4.1.20)** | Q03013 | GSTM4 | -2.15 | <0.001 | 0.030 | NO |
| **11-beta-hydroxysteroid dehydrogenase 1 (11-DH) (11-beta-HSD1) (EC 1.1.1.146) (7-oxosteroid reductase) (EC 1.1.1.201) (Corticosteroid 11-beta-dehydrogenase isozyme 1) (Short chain dehydrogenase/reductase family 26C member 1)** | P28845 | HSD11B1 | -2.02 | <0.001 | 0.030 | NO |
| **Aminopeptidase N (AP-N) (hAPN) (EC 3.4.11.2) (Alanyl aminopeptidase) (Aminopeptidase M) (AP-M) (Microsomal aminopeptidase) (Myeloid plasma membrane glycoprotein CD13) (gp150) (CD antigen CD13)** | P15144 | ANPEP | -2.01 | <0.001 | 0.020 | NO |
| **Neprilysin (EC 3.4.24.11) (Atriopeptidase) (Common acute lymphocytic leukemia antigen) (CALLA) (Enkephalinase) (Neutral endopeptidase 24.11) (NEP) (Neutral endopeptidase) (Skin fibroblast elastase) (SFE) (CD antigen CD10)** | P08473 | MME | -1.85 | <0.001 | 0.004 | NO |
| **Uncharacterized protein DKFZp686N08224** | Q6MZY5 | DKFZp686N08224 | -1.80 | <0.001 | 0.019 | NO |
| **cDNA FLJ55560, highly similar to Retinal dehydrogenase 2** | B4DH89 | NA | -1.80 | <0.001 | 0.006 | NO |
| **Neurofilament medium polypeptide (160 kDa neurofilament protein) (Neurofilament 3) (Neurofilament triplet M protein)** | A5YM63 | NEFM | -1.77 | 0.001 | 0.041 | YES |
| **MHC class I antigen** | A0A024F8H3 | HLA-B | -1.69 | <0.001 | 0.010 | NO |
| **E3 ubiquitin-protein ligase PPP1R11 (EC 2.3.2.27) (Hemochromatosis candidate gene V protein) (HCG V) (Protein phosphatase 1 regulatory subunit 11) (Protein phosphatase inhibitor 3)** | O60927 | PPP1R11 | -1.67 | <0.001 | 0.019 | NO |
| **Alpha-N-acetylglucosaminidase (EC 3.2.1.50) (N-acetyl-alpha-glucosaminidase) (NAG) [Cleaved into: Alpha-N-acetylglucosaminidase 82 kDa form; Alpha-N-acetylglucosaminidase 77 kDa form]** | P54802 | NAGLU | -1.66 | <0.001 | 0.004 | NO |
| **Signal peptidase complex catalytic subunit SEC11C (EC 3.4.21.89) (Microsomal signal peptidase 21 kDa subunit) (SPase 21 kDa subunit) (SEC11 homolog C) (SEC11-like protein 3) (SPC21)** | Q9BY50 | SEC11C | -1.64 | <0.001 | 0.030 | NO |
| **Angiotensin-converting enzyme (ACE) (EC 3.4.15.1) (Dipeptidyl carboxypeptidase I) (Kininase II) (CD antigen CD143) [Cleaved into: Angiotensin-converting enzyme, soluble form]** | P12821 | ACE | -1.60 | <0.001 | 0.011 | NO |
| **Glycogen phosphorylase, brain form (EC 2.4.1.1)** | P11216 | PYGB | -1.58 | <0.001 | 0.037 | NO |
| **Constitutive coactivator of peroxisome proliferator-activated receptor gamma (Constitutive coactivator of PPAR-gamma) (Constitutive coactivator of PPARG) (PPARG constitutive coactivator 1) (PGCC1) (Protein FAM120B)** | Q96EK7 | FAM120B | -1.52 | <0.001 | 0.024 | NO |
| **Ephrin-B1 (EFL-3) (ELK ligand) (ELK-L) (EPH-related receptor tyrosine kinase ligand 2) (LERK-2) [Cleaved into: Ephrin-B1 C-terminal fragment (Ephrin-B1 CTF); Ephrin-B1 intracellular domain (Ephrin-B1 ICD)]** | P98172 | EFNB1 | -1.52 | <0.001 | 0.018 | NO |
| **Glutamine synthetase (GS) (EC 6.3.1.2) (Glutamate--ammonia ligase) (Palmitoyltransferase GLUL) (EC 2.3.1.225)** | P15104 | GLUL | -1.51 | <0.001 | 0.031 | NO |
| **Melanotransferrin (Melanoma-associated antigen p97) (CD antigen CD228)** | P08582 | MELTF | -1.50 | <0.001 | 0.020 | NO |
| **Tubulin polymerization-promoting protein (TPPP) (EC 3.6.5.-) (25 kDa brain-specific protein) (TPPP/p25) (p24) (p25-alpha)** | O94811 | TPPP | -1.49 | <0.001 | 0.022 | NO |
| **Protein S100-A4 (Calvasculin) (Metastasin) (Placental calcium-binding protein) (Protein Mts1) (S100 calcium-binding protein A4)** | P26447 | S100A4 | -1.49 | <0.001 | 0.013 | NO |
| **Galactose mutarotase (EC 5.1.3.3) (Aldose 1-epimerase)** | Q96C23 | GALM | -1.40 | <0.001 | 0.012 | NO |
| **Leukocyte elastase inhibitor (LEI) (Monocyte/neutrophil elastase inhibitor) (EI) (M/NEI) (Peptidase inhibitor 2) (PI-2) (Serpin B1)** | P30740 | SERPINB1 | -1.40 | 0.001 | 0.045 | NO |
| **Aminopeptidase B (AP-B) (EC 3.4.11.6) (Arginine aminopeptidase) (Arginyl aminopeptidase)** | Q9H4A4 | RNPEP | -1.40 | <0.001 | 0.004 | NO |
| **Inositol 1,3,4-triphosphate 5/6 kinase, isoform CRA_a** | A0A024R6H3 | ITPK1 | -1.39 | <0.001 | 0.022 | NO |
| **Sphingosine 1-phosphate receptor 3 (S1P receptor 3) (S1P3) (Endothelial differentiation G-protein coupled receptor 3) (Sphingosine 1-phosphate receptor Edg-3) (S1P receptor Edg-3)** | Q99500 | S1PR3 | -1.37 | <0.001 | 0.020 | NO |
| **Oxidoreductase HTATIP2 (EC 1.1.1.-) (30 kDa HIV-1 TAT-interacting protein) (HIV-1 TAT-interactive protein 2)** | Q9BUP3 | HTATIP2 | -1.35 | <0.001 | 0.008 | NO |
| **Forkhead box protein O1 (Forkhead box protein O1A) (Forkhead in rhabdomyosarcoma)** | Q12778 | FOXO1 | -1.32 | <0.001 | 0.007 | YES |
| **Multidrug and toxin extrusion protein 1 (MATE-1) (hMATE-1) (Solute carrier family 47 member 1)** | Q96FL8 | SLC47A1 | -1.31 | <0.001 | 0.020 | NO |
| **Atrial natriuretic peptide-converting enzyme (EC 3.4.21.-) (Corin) (Heart-specific serine proteinase ATC2) (Pro-ANP-converting enzyme) (Transmembrane protease serine 10) [Cleaved into: Atrial natriuretic peptide-converting enzyme, N-terminal propeptide; Atrial natriuretic peptide-converting enzyme, activated protease fragment; Atrial natriuretic peptide-converting enzyme, 180 kDa soluble fragment; Atrial natriuretic peptide-converting enzyme, 160 kDa soluble fragment; Atrial natriuretic peptide-converting enzyme, 100 kDa soluble fragment]** | Q9Y5Q5 | CORIN | -1.29 | <0.001 | 0.034 | NO |
| **RNA binding motif (RNP1, RRM) protein 3, isoform CRA_c** | A0A024QYX3 | RBM3 | -1.26 | <0.001 | 0.010 | NO |
| **Endosialin (Tumor endothelial marker 1) (CD antigen CD248)** | Q9HCU0 | CD248 | -1.25 | <0.001 | 0.009 | YES |
| **Chitinase domain-containing protein 1** | B4DN31 | NA | -1.24 | <0.001 | 0.020 | NO |
| **Inactive C-alpha-formylglycine-generating enzyme 2 (Paralog of formylglycine-generating enzyme) (pFGE) (Sulfatase-modifying factor 2)** | Q8NBJ7 | SUMF2 | -1.24 | <0.001 | 0.031 | NO |
| **Protein-glutamine gamma-glutamyltransferase 2 (EC 2.3.2.13) (Erythrocyte transglutaminase) (Heart G alpha(h)) (hhG alpha(h)) (Isopeptidase TGM2) (EC 3.4.-.-) (Protein G alpha(h)) (G(h)) (Protein-glutamine deamidase TGM2) (EC 3.5.1.44) (Protein-glutamine dopaminyltransferase TGM2) (EC 2.3.1.-) (Protein-glutamine histaminyltransferase TGM2) (EC 2.3.1.-) (Protein-glutamine noradrenalinyltransferase TGM2) (EC 2.3.1.-) (Protein-glutamine serotonyltransferase TGM2) (EC 2.3.1.-) (Tissue transglutaminase) (tTG) (tTgase) (Transglutaminase C) (TG(C)) (TGC) (TGase C) (Transglutaminase H) (TGase H) (Transglutaminase II) (TGase II) (Transglutaminase-2) (TG2) (TGase-2) (hTG2)** | P21980 | TGM2 | -1.23 | 0.001 | 0.041 | NO |
| **Ubiquitin carboxyl-terminal hydrolase 16 (EC 3.4.19.12) (Deubiquitinating enzyme 16) (Ubiquitin thioesterase 16) (Ubiquitin-processing protease UBP-M) (Ubiquitin-specific-processing protease 16)** | Q9Y5T5 | USP16 | -1.19 | <0.001 | 0.038 | YES |
| **NA** | P58107 | NA | -1.17 | <0.001 | 0.031 | NO |
| **Peptidyl-prolyl cis-trans isomerase-like 3 (PPIase) (EC 5.2.1.8) (Cyclophilin J) (CyPJ) (Cyclophilin-like protein PPIL3) (Rotamase PPIL3)** | Q9H2H8 | PPIL3 | -1.16 | 0.002 | 0.049 | NO |
| **non-specific serine/threonine protein kinase (EC 2.7.11.1)** | D7RF68 | NA | -1.16 | <0.001 | 0.038 | NO |
| **Activating signal cointegrator 1 complex subunit 2 (ASC-1 complex subunit p100) (Trip4 complex subunit p100)** | Q9H1I8 | ASCC2 | -1.15 | <0.001 | 0.028 | NO |
| **Ankyrin repeat domain-containing protein SOWAHC (Ankyrin repeat domain-containing protein 57) (Protein sosondowah homolog C)** | Q53LP3 | SOWAHC | -1.15 | <0.001 | 0.024 | NO |
| **LIM and SH3 protein 1, isoform CRA_b** | A0A024R1S8 | LASP1 | -1.13 | <0.001 | 0.030 | YES |
| **Thioredoxin-like protein 4A (DIM1 protein homolog) (Spliceosomal U5 snRNP-specific 15 kDa protein) (Thioredoxin-like U5 snRNP protein U5-15kD)** | P83876 | TXNL4A | -1.13 | <0.001 | 0.028 | NO |
| **UBX domain-containing protein 6 (UBX domain-containing protein 1)** | Q9BZV1 | UBXN6 | -1.11 | <0.001 | 0.034 | NO |
| **Legumain (EC 3.4.22.34) (Asparaginyl endopeptidase) (AEP) (Protease, cysteine 1)** | Q99538 | LGMN | -1.10 | 0.001 | 0.048 | NO |
| **Biogenesis of lysosome-related organelles complex 1 subunit 3 (BLOC-1 subunit 3)** | Q6QNY0 | BLOC1S3 | -1.10 | <0.001 | 0.038 | YES |
| **Splicing factor 3B subunit 4 (Pre-mRNA-splicing factor SF3b 49 kDa subunit) (Spliceosome-associated protein 49) (SAP 49)** | Q15427 | SF3B4 | -1.09 | <0.001 | 0.030 | NO |
| **Zinc finger FYVE domain-containing protein 21 (ZF21)** | Q9BQ24 | ZFYVE21 | -1.06 | <0.001 | 0.038 | NO |
| **Protein N-terminal asparagine amidohydrolase (EC 3.5.1.121) (Protein NH2-terminal asparagine amidohydrolase) (PNAA) (Protein NH2-terminal asparagine deamidase) (PNAD) (Protein N-terminal Asn amidase) (Protein N-terminal asparagine amidase) (Protein NTN-amidase)** | Q96AB6 | NTAN1 | -1.05 | <0.001 | 0.030 | NO |
| **SAM and SH3 domain-containing protein 1 (Proline-glutamate repeat-containing protein)** | O94885 | SASH1 | -1.04 | <0.001 | 0.030 | YES |
| **A-kinase anchor protein 12 (AKAP-12) (A-kinase anchor protein 250 kDa) (AKAP 250) (Gravin) (Myasthenia gravis autoantigen)** | Q02952 | AKAP12 | -1.02 | <0.001 | 0.022 | YES |
| **Nucleotide sugar transporter SLC35D1 (Solute carrier family 35 member D1) (UDP-galactose transporter-related protein 7) (UGTrel7) (UDP-glucuronic acid/UDP-N-acetylgalactosamine transporter) (UDP-GlcA/UDP-GalNAc transporter)** | Q9NTN3 | SLC35D1 | -1.01 | <0.001 | 0.037 | NO |
| **6-phosphofructo-2-kinase/fructose-2,6-bisphosphatase 2 (6PF-2-K/Fru-2,6-P2ase 2) (PFK/FBPase 2) (6PF-2-K/Fru-2,6-P2ase heart-type isozyme) [Includes: 6-phosphofructo-2-kinase (EC 2.7.1.105); Fructose-2,6-bisphosphatase (EC 3.1.3.46)]** | O60825 | PFKFB2 | -1.01 | <0.001 | 0.024 | NO |
| **Inverted formin-2 (HBEBP2-binding protein C)** | Q27J81 | INF2 | -1.01 | <0.001 | 0.001 | NO |

**Supplementary Information 4:** Total proteins which significantly (p≤0.05) at least doubled and passed FDR (q≤0.05) in the failed IOL group compared with the elCS group under the relaxed-phase during spontaneous contractions condition, ordered by log-fold change.

| **Protein name** | **Accession** | **Gene name** | **Log Fold-change** | **p-value** | **q-value** | **Phosphorylation identified** |
| --- | --- | --- | --- | --- | --- | --- |
| **Galectin** | Q6NVH9 | LGALS3 | 8.93 | <0.001 | 0.004 | NO |
| **Desmuslin, isoform CRA_a (Synemin)** | A0A075B7B1 | SYNM | 5.77 | <0.001 | 0.001 | NO |
| **Large ribosomal subunit protein eL14 (60S ribosomal protein L14)** | Q6IPH7 | RPL14 | 5.51 | <0.001 | <0.001 | NO |
| **maleylacetoacetate isomerase (EC 5.2.1.2)** | G3V4T6 | GSTZ1 | 4.65 | <0.001 | <0.001 | NO |
| **Calpain-2 catalytic subunit (EC 3.4.22.53) (Calcium-activated neutral proteinase 2) (Calpain M-type) (Calpain-2 large subunit) (Millimolar-calpain)** | B2RCM3 | NA | 4.65 | <0.001 | <0.001 | NO |
| **Choline transporter-like protein** | A0A088QCU6 | SLC44A2 | 4.63 | <0.001 | 0.021 | NO |
| **Cartilage oligomeric matrix protein (COMP) (Thrombospondin-5) (TSP5)** | P49747 | COMP | 4.59 | <0.001 | <0.001 | NO |
| **Thrombospondin-4** | P35443 | THBS4 | 4.52 | <0.001 | <0.001 | NO |
| **Zinc finger, UBR1 type 1, isoform CRA_c** | A0A024RAC9 | ZUBR1 | 4.10 | <0.001 | 0.002 | NO |
| **Transforming growth factor beta-2 proprotein (Cetermin) (Glioblastoma-derived T-cell suppressor factor) (G-TSF) [Cleaved into: Latency-associated peptide (LAP); Transforming growth factor beta-2 (TGF-beta-2)]** | P61812 | TGFB2 | 3.78 | <0.001 | <0.001 | NO |
| **Carbonic anhydrase III, muscle specific, isoform CRA_a** | A0A024R825 | CA3 | 3.38 | <0.001 | <0.001 | NO |
| **Serpin B5 (Maspin) (Peptidase inhibitor 5) (PI-5)** | P36952 | SERPINB5 | 3.38 | 0.002 | 0.034 | NO |
| **Thrombospondin 1, isoform CRA_a** | A0A024R9Q1 | THBS1 | 3.22 | <0.001 | <0.001 | NO |
| **Ankyrin-3 (ANK-3) (Ankyrin-G)** | Q12955 | ANK3 | 3.12 | 0.003 | 0.040 | NO |
| **Matrix Gla protein (MGP) (Cell growth-inhibiting gene 36 protein)** | P08493 | MGP | 3.10 | <0.001 | <0.001 | NO |
| **Thrombospondin-1 (Glycoprotein G)** | P07996 | THBS1 | 3.04 | <0.001 | 0.007 | NO |
| **TNC variant protein** | Q4LE33 | TNC variant protein | 2.96 | <0.001 | <0.001 | YES |
| **Thrombospondin-2** | P35442 | THBS2 | 2.94 | <0.001 | 0.002 | NO |
| **Serpin peptidase inhibitor, clade E (Nexin, plasminogen activator inhibitor type 1), member 1, isoform CRA_b** | A0A024QYT5 | SERPINE1 | 2.93 | <0.001 | <0.001 | NO |
| **Immunoglobulin heavy constant gamma 4 (Ig gamma-4 chain C region)** | P01861 | IGHG4 | 2.84 | <0.001 | 0.005 | NO |
| **CCN family member 4 (WNT1-inducible-signaling pathway protein 1) (WISP-1) (Wnt-1-induced secreted protein)** | O95388 | CCN4 | 2.83 | <0.001 | <0.001 | NO |
| **Transforming growth factor-beta-induced protein ig-h3 (Beta ig-h3) (Kerato-epithelin) (RGD-containing collagen-associated protein) (RGD-CAP)** | Q15582 | TGFBI | 2.77 | <0.001 | <0.001 | NO |
| **Solute carrier family 16 (Monocarboxylic acid transporters), member 3, isoform CRA_a** | A0A024R8U1 | SLC16A3 | 2.68 | <0.001 | 0.001 | NO |
| **Cadherin 2, type 1, N-cadherin (Neuronal), isoform CRA_b** | A0A024RC42 | CDH2 | 2.68 | <0.001 | <0.001 | NO |
| **Annexin A3 (35-alpha calcimedin) (Annexin III) (Annexin-3) (Inositol 1,2-cyclic phosphate 2-phosphohydrolase) (Lipocortin III) (Placental anticoagulant protein III) (PAP-III)** | P12429 | ANXA3 | 2.68 | <0.001 | <0.001 | NO |
| **Matrilin 2, isoform CRA_b** | A0A024R9B9 | MATN2 | 2.64 | <0.001 | 0.015 | NO |
| **Midkine (MK) (Amphiregulin-associated protein) (ARAP) (Midgestation and kidney protein) (Neurite outgrowth-promoting factor 2) (Neurite outgrowth-promoting protein)** | P21741 | MDK | 2.60 | <0.001 | 0.021 | NO |
| **Alkaline phosphatase, tissue-nonspecific isozyme (AP-TNAP) (TNS-ALP) (TNSALP) (EC 3.1.3.1) (Alkaline phosphatase liver/bone/kidney isozyme) (Phosphoamidase) (Phosphocreatine phosphatase) (EC 3.9.1.1)** | P05186 | ALPL | 2.54 | <0.001 | <0.001 | NO |
| **Unconventional myosin-If (Myosin-Ie)** | O00160 | MYO1F | 2.46 | <0.001 | 0.008 | NO |
| **Secreted frizzled-related protein 1 (FRP-1) (sFRP-1) (Secreted apoptosis-related protein 2) (SARP-2)** | Q8N474 | SFRP1 | 2.36 | <0.001 | <0.001 | NO |
| **Periostin, osteoblast specific factor, isoform CRA_a** | A0A024RDT5 | POSTN | 2.35 | <0.001 | 0.006 | NO |
| **N90-VRC38.08 heavy chain variable region** | A0A1W6IYI5 | NA | 2.34 | <0.001 | 0.002 | NO |
| **Serine racemase (EC 5.1.1.18) (D-serine ammonia-lyase) (D-serine dehydratase) (EC 4.3.1.18) (L-serine ammonia-lyase) (L-serine dehydratase) (EC 4.3.1.17)** | Q9GZT4 | SRR | 2.33 | <0.001 | 0.005 | NO |
| **Sodium-dependent phosphate transporter 2 (Gibbon ape leukemia virus receptor 2) (GLVR-2) (Phosphate transporter 2) (PiT-2) (Pit2) (hPit2) (Solute carrier family 20 member 2)** | Q08357 | SLC20A2 | 2.32 | <0.001 | <0.001 | NO |
| **Tenascin C** | A0A0U1RR80 | TNC | 2.25 | 0.001 | 0.024 | NO |
| **NA** | Q15063 | NA | 2.16 | <0.001 | 0.008 | NO |
| **cDNA FLJ51896, highly similar to Glia-derived nexin** | B4DMR3 | NA | 2.12 | <0.001 | <0.001 | NO |
| **Collagen alpha-1(XV) chain [Cleaved into: Restin (Endostatin-XV) (Related to endostatin) (Restin-I); Restin-2 (Restin-II); Restin-3 (Restin-III); Restin-4 (Restin-IV)]** | P39059 | COL15A1 | 2.12 | <0.001 | <0.001 | NO |
| **MHC class II antigen** | A0A1Y6MRR4 | HLA-DPB1 | 2.12 | <0.001 | 0.008 | NO |
| **Tenascin-X (TN-X) (Hexabrachion-like protein)** | P22105 | TNXB | 2.11 | <0.001 | <0.001 | YES |
| **Cysteine-rich motor neuron 1 protein (CRIM-1) (Cysteine-rich repeat-containing protein S52) [Cleaved into: Processed cysteine-rich motor neuron 1 protein]** | Q9NZV1 | CRIM1 | 2.08 | <0.001 | 0.008 | NO |
| **Lysyl oxidase homolog 2 (EC 1.4.3.13) (Lysyl oxidase-like protein 2) (Lysyl oxidase-related protein 2) (Lysyl oxidase-related protein WS9-14)** | Q9Y4K0 | LOXL2 | 1.99 | <0.001 | <0.001 | NO |
| **Polypeptide N-acetylgalactosaminyltransferase 18 (EC 2.4.1.41) (Polypeptide GalNAc transferase 18) (GalNAc-T18) (Polypeptide GalNAc transferase-like protein 4) (GalNAc-T-like protein 4) (pp-GaNTase-like protein 4) (Polypeptide N-acetylgalactosaminyltransferase-like protein 4) (Protein-UDP acetylgalactosaminyltransferase-like protein 4) (UDP-GalNAc:polypeptide N-acetylgalactosaminyltransferase-like protein 4)** | Q6P9A2 | GALNT18 | 1.99 | <0.001 | <0.001 | NO |
| **L-amino-acid oxidase (LAAO) (LAO) (EC 1.4.3.2) (EC 1.4.3.25) (Interleukin-4-induced protein 1) (IL4-induced protein 1) (hIL4I1) (Protein Fig-1) (hFIG1)** | Q96RQ9 | IL4I1 | 1.96 | <0.001 | 0.014 | NO |
| **PG-M** | Q6MZK8 | DKFZp686K06110 | 1.95 | <0.001 | 0.011 | YES |
| **Zinc finger FYVE domain-containing protein 26 (FYVE domain-containing centrosomal protein) (FYVE-CENT) (Spastizin)** | Q68DK2 | ZFYVE26 | 1.93 | <0.001 | 0.003 | NO |
| **Extracellular sulfatase Sulf-1 (hSulf-1) (Arylsulfatase) (EC 3.1.6.1) (N-acetylglucosamine-6-sulfatase) (EC 3.1.6.14) [Cleaved into: Extracellular sulfatase Sulf-2 secreted form]** | Q8IWU6 | SULF1 | 1.85 | <0.001 | 0.021 | NO |
| **IGH@ protein** | Q6P089 | IGH@ | 1.84 | <0.001 | 0.018 | NO |
| **Cytoplasmic dynein 2 heavy chain 1 (Cytoplasmic dynein 2 heavy chain) (Dynein cytoplasmic heavy chain 2) (Dynein heavy chain 11) (hDHC11) (Dynein heavy chain isotype 1B)** | Q8NCM8 | DYNC2H1 | 1.84 | <0.001 | 0.016 | NO |
| **Calmodulin regulator protein PCP4 (Brain-specific polypeptide PEP-19) (Purkinje cell protein 4)** | P48539 | PCP4 | 1.83 | 0.002 | 0.036 | NO |
| **Matrilin-2** | O00339 | MATN2 | 1.83 | <0.001 | 0.002 | NO |
| **SAA2-SAA4 readthrough** | A0A096LPE2 | SAA2-SAA4 | 1.81 | <0.001 | <0.001 | NO |
| **Biglycan (Bone/cartilage proteoglycan I) (PG-S1)** | P21810 | BGN | 1.78 | <0.001 | 0.004 | NO |
| **Protein bicaudal D homolog 1 (Bic-D 1)** | Q96G01 | BICD1 | 1.77 | <0.001 | 0.002 | NO |
| **Myosin-reactive immunoglobulin heavy chain variable region** | Q9UL90 | NA | 1.74 | <0.001 | 0.010 | NO |
| **D-3-phosphoglycerate dehydrogenase (3-PGDH) (EC 1.1.1.95) (2-oxoglutarate reductase) (EC 1.1.1.399) (Malate dehydrogenase) (EC 1.1.1.37)** | O43175 | PHGDH | 1.74 | <0.001 | 0.014 | NO |
| **Prostaglandin G/H synthase 1 (EC 1.14.99.1) (Cyclooxygenase-1) (COX-1) (Prostaglandin H2 synthase 1) (PGH synthase 1) (PGHS-1) (PHS 1) (Prostaglandin-endoperoxide synthase 1)** | P23219 | PTGS1 | 1.74 | <0.001 | <0.001 | NO |
| **[Pyruvate dehydrogenase (acetyl-transferring)] kinase isozyme 1, mitochondrial (EC 2.7.11.2) (Pyruvate dehydrogenase kinase isoform 1) (PDH kinase 1)** | Q15118 | PDK1 | 1.72 | <0.001 | 0.002 | NO |
| **Inter-alpha-trypsin inhibitor heavy chain H1 (ITI heavy chain H1) (ITI-HC1) (Inter-alpha-inhibitor heavy chain 1) (Inter-alpha-trypsin inhibitor complex component III) (Serum-derived hyaluronan-associated protein) (SHAP)** | P19827 | ITIH1 | 1.72 | <0.001 | <0.001 | NO |
| **Collagen alpha-1(XII) chain** | Q99715 | COL12A1 | 1.71 | <0.001 | 0.010 | NO |
| **Neural cell adhesion molecule 1** | A0A087WTF6 | NCAM1 | 1.71 | <0.001 | <0.001 | NO |
| **Fibrillin-2 [Cleaved into: Placensin]** | P35556 | FBN2 | 1.62 | <0.001 | 0.015 | NO |
| **Tumor-associated calcium signal transducer 2 (Cell surface glycoprotein Trop-2) (Membrane component chromosome 1 surface marker 1) (Pancreatic carcinoma marker protein GA733-1)** | P09758 | TACSTD2 | 1.60 | <0.001 | 0.014 | NO |
| **Matrix metalloproteinase-19 (MMP-19) (EC 3.4.24.-) (Matrix metalloproteinase RASI) (Matrix metalloproteinase-18) (MMP-18)** | Q99542 | MMP19 | 1.58 | <0.001 | <0.001 | NO |
| **OX-2 membrane glycoprotein (CD antigen CD200)** | P41217 | CD200 | 1.55 | <0.001 | <0.001 | NO |
| **Prostacyclin synthase (EC 5.3.99.4) (Hydroperoxy icosatetraenoate dehydratase) (EC 4.2.1.152) (Prostaglandin I2 synthase)** | Q16647 | PTGIS | 1.53 | <0.001 | <0.001 | NO |
| **CCN family member 2 (Cellular communication network factor 2) (Connective tissue growth factor)** | Q5M8T4 | CTGF | 1.53 | <0.001 | 0.005 | NO |
| **FXYD domain containing ion transport regulator 6, isoform CRA_a** | A0A024R3J8 | FXYD6 | 1.53 | <0.001 | 0.003 | NO |
| **Cerebral cavernous malformations 2 protein (Malcavernin)** | Q9BSQ5 | CCM2 | 1.49 | 0.001 | 0.026 | NO |
| **Vitamin D binding protein** | A0A1B1CYC5 | Gc | 1.49 | <0.001 | 0.002 | NO |
| **SH3 domain-binding glutamic acid-rich-like protein 2 (Fovea-associated SH3 domain-binding protein)** | Q9UJC5 | SH3BGRL2 | 1.49 | <0.001 | <0.001 | NO |
| **NATenascin-X** | A0A087WWA5 | TNXB | 1.48 | <0.001 | 0.016 | NO |
| **Apolipoprotein(a) (Apo(a)) (Lp(a)) (EC 3.4.21.-)** | P08519 | LPA | 1.46 | 0.002 | 0.031 | NO |
| **Fibulin-1 (FIBL-1)** | P23142 | FBLN1 | 1.45 | <0.001 | 0.017 | YES |
| **Metalloproteinase inhibitor 1 (Erythroid-potentiating activity) (EPA) (Fibroblast collagenase inhibitor) (Collagenase inhibitor) (Tissue inhibitor of metalloproteinases 1) (TIMP-1)** | P01033 | TIMP1 | 1.45 | <0.001 | 0.003 | NO |
| **Protein S100-P (Migration-inducing gene 9 protein) (MIG9) (Protein S100-E) (S100 calcium-binding protein P)** | P25815 | S100P | 1.45 | 0.002 | 0.034 | NO |
| **Asporin (Periodontal ligament-associated protein 1) (PLAP-1)** | Q9BXN1 | ASPN | 1.45 | <0.001 | 0.013 | NO |
| **Nephronectin (Preosteoblast EGF-like repeat protein with MAM domain) (Protein EGFL6-like)** | Q6UXI9 | NPNT | 1.44 | 0.003 | 0.043 | NO |
| **Fibronectin 1, isoform CRA_n** | A0A024R462 | FN1 | 1.43 | 0.002 | 0.033 | NO |
| **Tubulointerstitial nephritis antigen-like (Glucocorticoid-inducible protein 5) (Oxidized LDL-responsive gene 2 protein) (OLRG-2) (Tubulointerstitial nephritis antigen-related protein) (TIN Ag-related protein) (TIN-Ag-RP)** | Q9GZM7 | TINAGL1 | 1.43 | <0.001 | 0.021 | NO |
| **Chondroitin sulfate proteoglycan 2 (Versican), isoform CRA_c** | A0A024RAL1 | CSPG2 | 1.43 | <0.001 | 0.006 | YES |
| **Insulin-like growth factor-binding protein 7 (IBP-7) (IGF-binding protein 7) (IGFBP-7) (IGFBP-rP1) (MAC25 protein) (PGI2-stimulating factor) (Prostacyclin-stimulating factor) (Tumor-derived adhesion factor) (TAF)** | Q16270 | IGFBP7 | 1.42 | <0.001 | 0.005 | NO |
| **Latent-transforming growth factor beta-binding protein 2 (LTBP-2)** | Q14767 | LTBP2 | 1.42 | <0.001 | 0.003 | NO |
| **Mitochondrial adenyl nucleotide antiporter SLC25A23 (Mitochondrial ATP-Mg/Pi carrier protein 2) (Short calcium-binding mitochondrial carrier protein 3) (SCaMC-3) (Solute carrier family 25 member 23)** | Q9BV35 | SLC25A23 | 1.42 | <0.001 | 0.012 | NO |
| **Lipopolysaccharide-binding protein (LBP)** | P18428 | LBP | 1.41 | 0.002 | 0.030 | NO |
| **Synaptophysin-like protein 2** | Q5VXT5 | SYPL2 | 1.37 | <0.001 | 0.005 | NO |
| **Microfibril-associated glycoprotein 4** | P55083 | MFAP4 | 1.37 | 0.003 | 0.040 | NO |
| **Gamma-interferon-inducible lysosomal thiol reductase (EC 1.8.-.-) (Gamma-interferon-inducible protein IP-30)** | A8K686 | NA | 1.36 | 0.003 | 0.043 | NO |
| **Fibulin-1** | B4DUV1 | NA | 1.33 | 0.002 | 0.034 | NO |
| **Core histone macro-H2A.1 (Histone macroH2A1) (mH2A1) (Histone H2A.y) (H2A/y) (Medulloblastoma antigen MU-MB-50.205)** | O75367 | MACROH2A1 | 1.32 | 0.004 | 0.049 | NO |
| **CCN family member 1 (Cellular communication network factor 1) (Cysteine-rich angiogenic inducer 61) (Insulin-like growth factor-binding protein 10) (IBP-10) (IGF-binding protein 10) (IGFBP-10) (Protein CYR61) (Protein GIG1)** | O00622 | CCN1 | 1.32 | <0.001 | 0.012 | NO |
| **Matrix-remodeling-associated protein 5 (Adhesion protein with leucine-rich repeats and immunoglobulin domains related to perlecan) (Adlican)** | Q9NR99 | MXRA5 | 1.32 | 0.001 | 0.024 | NO |
| **Solute carrier family 2, facilitated glucose transporter member 4 (Glucose transporter type 4, insulin-responsive) (GLUT-4)** | P14672 | SLC2A4 | 1.31 | <0.001 | 0.018 | NO |
| **C14orf159 variant protein** | Q4LE40 | C14orf159 variant protein | 1.30 | <0.001 | <0.001 | NO |
| **Septin-6** | Q14141 | SEPTIN6 | 1.29 | <0.001 | <0.001 | NO |
| **Ribonuclease T2 (EC 4.6.1.19) (Ribonuclease 6)** | O00584 | RNASET2 | 1.28 | <0.001 | 0.008 | NO |
| **Something about silencing protein 10 (Charged amino acid-rich leucine zipper 1) (CRL1) (Disrupter of silencing SAS10) (UTP3 homolog)** | Q9NQZ2 | UTP3 | 1.26 | <0.001 | 0.021 | NO |
| **Cytochrome c oxidase subunit 1 (EC 7.1.1.9) (Cytochrome c oxidase polypeptide I)** | P00395 | MT-CO1 | 1.25 | 0.001 | 0.022 | NO |
| **Alpha-1B-glycoprotein (Alpha-1-B glycoprotein)** | P04217 | A1BG | 1.25 | <0.001 | 0.005 | NO |
| **von Willebrand factor A domain-containing protein 1** | Q6PCB0 | VWA1 | 1.24 | <0.001 | 0.009 | NO |
| **Growth arrest-specific protein 6 (GAS-6) (AXL receptor tyrosine kinase ligand)** | Q14393 | GAS6 | 1.24 | 0.003 | 0.044 | NO |
| **ADP/ATP translocase 1 (ADP,ATP carrier protein 1) (ADP,ATP carrier protein, heart/skeletal muscle isoform T1) (Adenine nucleotide translocator 1) (ANT 1) (Solute carrier family 25 member 4)** | P12235 | SLC25A4 | 1.23 | <0.001 | 0.003 | NO |
| **Haptoglobin (Zonulin) [Cleaved into: Haptoglobin alpha chain; Haptoglobin beta chain]** | P00738 | HP | 1.21 | 0.002 | 0.030 | NO |
| **Laminin subunit alpha-2 (Laminin M chain) (Laminin-12 subunit alpha) (Laminin-2 subunit alpha) (Laminin-4 subunit alpha) (Merosin heavy chain)** | P24043 | LAMA2 | 1.21 | <0.001 | 0.008 | NO |
| **Latent-transforming growth factor beta-binding protein 1 (LTBP-1) (Transforming growth factor beta-1-binding protein 1) (TGF-beta1-BP-1)** | Q14766 | LTBP1 | 1.20 | <0.001 | 0.015 | NO |
| **Adipocyte enhancer-binding protein 1 (AE-binding protein 1) (Aortic carboxypeptidase-like protein)** | Q8IUX7 | AEBP1 | 1.18 | <0.001 | 0.017 | NO |
| **Dual adapter for phosphotyrosine and 3-phosphotyrosine and 3-phosphoinositide (hDAPP1) (B lymphocyte adapter protein Bam32) (B-cell adapter molecule of 32 kDa)** | Q9UN19 | DAPP1 | 1.18 | <0.001 | 0.021 | NO |
| **Protein AMBP (Protein HC) [Cleaved into: Alpha-1-microglobulin (EC 1.6.2.-) (Alpha-1 microglycoprotein) (Complex-forming glycoprotein heterogeneous in charge); Inter-alpha-trypsin inhibitor light chain (ITI-LC) (Bikunin) (EDC1) (HI-30) (Uronic-acid-rich protein); Trypstatin]** | P02760 | AMBP | 1.18 | <0.001 | 0.016 | NO |
| **Complement C5 (C3 and PZP-like alpha-2-macroglobulin domain-containing protein 4) [Cleaved into: Complement C5 beta chain; Complement C5 alpha chain; C5a anaphylatoxin; Complement C5 alpha' chain]** | P01031 | C5 | 1.17 | <0.001 | 0.021 | YES |
| **Zinc transporter 7 (ZnT-7) (Solute carrier family 30 member 7) (Znt-like transporter 2)** | Q8NEW0 | SLC30A7 | 1.16 | <0.001 | 0.010 | NO |
| **Inter-alpha-trypsin inhibitor heavy chain H2 (ITI heavy chain H2) (ITI-HC2) (Inter-alpha-inhibitor heavy chain 2) (Inter-alpha-trypsin inhibitor complex component II) (Serum-derived hyaluronan-associated protein) (SHAP)** | P19823 | ITIH2 | 1.16 | <0.001 | <0.001 | NO |
| **Pentraxin-related protein PTX3 (Pentaxin-related protein PTX3) (Tumor necrosis factor alpha-induced protein 5) (TNF alpha-induced protein 5) (Tumor necrosis factor-inducible gene 14 protein) (TSG-14)** | P26022 | PTX3 | 1.16 | 0.002 | 0.028 | NO |
| **Dynein axonemal heavy chain 5 (Axonemal beta dynein heavy chain 5) (Ciliary dynein heavy chain 5)** | Q8TE73 | DNAH5 | 1.13 | 0.001 | 0.027 | NO |
| **Filamin A-interacting protein 1-like (130 kDa GPBP-interacting protein) (90 kDa GPBP-interacting protein) (Protein down-regulated in ovarian cancer 1) (DOC-1)** | Q4L180 | FILIP1L | 1.13 | <0.001 | 0.004 | YES |
| **Sushi repeat-containing protein SRPX** | P78539 | SRPX | 1.13 | 0.002 | 0.035 | NO |
| **cDNA, FLJ92657, highly similar to Homo sapiens heterogeneous nuclear ribonucleoprotein C (C1/C2) (HNRPC), transcript variant 2, mRNA** | B2R5W2 | NA | 1.12 | 0.002 | 0.034 | NO |
| **Fibroleukin (Fibrinogen-like protein 2) (pT49)** | Q14314 | FGL2 | 1.11 | 0.002 | 0.033 | NO |
| **Lactadherin (Breast epithelial antigen BA46) (HMFG) (MFGM) (Milk fat globule-EGF factor 8) (MFG-E8) (SED1) [Cleaved into: Lactadherin short form; Medin]** | Q08431 | MFGE8 | 1.10 | 0.001 | 0.025 | NO |
| **Complement factor H (H factor 1)** | P08603 | CFH | 1.07 | <0.001 | 0.005 | NO |
| **Chromosome 1 open reading frame 24, isoform CRA_a** | A0A024R978 | C1orf24 | 1.07 | 0.001 | 0.024 | NO |
| **Uncharacterized protein DKFZp686O1553** | Q5HYM1 | DKFZp686O1553 | 1.06 | <0.001 | 0.004 | NO |
| **Disheveled-associated activator of morphogenesis 2** | Q86T65 | DAAM2 | 1.05 | 0.002 | 0.030 | NO |
| **Latent-transforming growth factor beta-binding protein 4 (LTBP-4)** | Q8N2S1 | LTBP4 | 1.05 | 0.001 | 0.025 | NO |
| **N-acetylgalactosaminyltransferase 7 (EC 2.4.1.41) (Polypeptide GalNAc transferase 7) (GalNAc-T7) (pp-GaNTase 7) (Protein-UDP acetylgalactosaminyltransferase 7) (UDP-GalNAc:polypeptide N-acetylgalactosaminyltransferase 7)** | Q86SF2 | GALNT7 | 1.05 | 0.004 | 0.046 | NO |
| **Procollagen-lysine,2-oxoglutarate 5-dioxygenase 2 (EC 1.14.11.4) (Lysyl hydroxylase 2) (LH2)** | O00469 | PLOD2 | 1.05 | <0.001 | 0.016 | NO |
| **Apolipoprotein C-III (Apo-CIII) (ApoC-III) (Apolipoprotein C3)** | P02656 | APOC3 | 1.05 | 0.003 | 0.044 | NO |
| **glutaminase (EC 3.5.1.2)** | B3KM58 | NA | 1.05 | <0.001 | 0.013 | NO |
| **Pregnancy zone protein (C3 and PZP-like alpha-2-macroglobulin domain-containing protein 6)** | P20742 | PZP | 1.03 | 0.003 | 0.041 | NO |
| **cDNA, FLJ93255, highly similar to Homo sapiens solute carrier family 7 (cationic amino acidtransporter, y+ system), member 1 (SLC7A1), mRNA** | B2R728 | NA | 1.03 | 0.001 | 0.025 | NO |
| **Twisted gastrulation protein homolog 1** | Q9GZX9 | TWSG1 | 1.02 | 0.002 | 0.028 | NO |
| **Alpha-catulin (Alpha-catenin-related protein) (ACRP) (Catenin alpha-like protein 1)** | Q9UBT7 | CTNNAL1 | 1.02 | 0.002 | 0.032 | NO |
| **Galectin-8 (Gal-8) (Po66 carbohydrate-binding protein) (Po66-CBP) (Prostate carcinoma tumor antigen 1) (PCTA-1)** | O00214 | LGALS8 | 1.02 | 0.003 | 0.044 | NO |
| **Corrinoid adenosyltransferase MMAB (EC 2.5.1.-) (ATP:co(I)rrinoid adenosyltransferase MMAB) (Methylmalonic aciduria type B protein)** | Q96EY8 | MMAB | 1.02 | 0.002 | 0.034 | NO |
| **Aldehyde dehydrogenase X, mitochondrial (EC 1.2.1.3) (Aldehyde dehydrogenase 5) (Aldehyde dehydrogenase family 1 member B1)** | P30837 | ALDH1B1 | 1.02 | <0.001 | 0.020 | NO |
| **Versican core protein (Chondroitin sulfate proteoglycan core protein 2) (Large fibroblast proteoglycan) (PG-M)** | Q59FG9 | NA | 1.01 | <0.001 | 0.020 | NO |
| **Monocarboxylate transporter 2 (MCT 2) (Solute carrier family 16 member 7)** | O60669 | SLC16A7 | 1.00 | <0.001 | 0.018 | YES |
| **Glutathione peroxidase 3 (GPx-3) (GSHPx-3) (EC 1.11.1.9) (Extracellular glutathione peroxidase) (Plasma glutathione peroxidase) (GPx-P) (GSHPx-P)** | P22352 | GPX3 | 1.00 | 0.003 | 0.038 | NO |
| **Phospholipid scramblase 1 (PL scramblase 1) (Ca(2+)-dependent phospholipid scramblase 1) (Erythrocyte phospholipid scramblase) (Mg(2+)-dependent nuclease) (EC 3.1.-.-) (MmTRA1b)** | O15162 | PLSCR1 | 1.00 | 0.003 | 0.038 | NO |

**Supplementary Information 5:** Total proteins which significantly (p≤0.05) at least halved and passed FDR (q≤0.05) in the failed IOL group compared with the elCS group under the relaxed-phase during spontaneous contractions condition, ordered by log-fold change.

|  |  |  |  |  |  |  |
| --- | --- | --- | --- | --- | --- | --- |
| **Protein name** | **Accession** | **Gene name** | **Log Fold-change** | **p-value** | **q-value** | **Phosphorylation identified** |
| **Glutathione S-transferase Mu 1 (EC 2.5.1.18) (GST HB subunit 4) (GST class-mu 1) (GSTM1-1) (GSTM1a-1a) (GSTM1b-1b) (GTH4)** | P09488 | GSTM1 | -6.85 | <0.001 | <0.001 | NO |
| **Angiotensin-converting enzyme 2 (EC 3.4.17.23) (Angiotensin-converting enzyme homolog) (ACEH) (Angiotensin-converting enzyme-related carboxypeptidase) (ACE-related carboxypeptidase) (EC 3.4.17.-) (Metalloprotease MPROT15) [Cleaved into: Processed angiotensin-converting enzyme 2]** | Q9BYF1 | ACE2 | -6.49 | <0.001 | <0.001 | NO |
| **MHC class I antigen** | A0A1X9I3T8 | HLA-A | -6.25 | <0.001 | <0.001 | NO |
| **cDNA FLJ39696 fis, clone SMINT2011033, highly similar to Sorting and assembly machinery component 50 homolog** | B3KUE6 | NA | -6.25 | <0.001 | 0.008 | NO |
| **MHC class I antigen (MHC class I protein)** | K7WT83 | HLA-A | -5.42 | 0.001 | 0.024 | NO |
| **Alpha-S1-casein (allergen Bos d 8) [Cleaved into: Antioxidant peptide]** | P02662 | CSN1S1 | -5.37 | <0.001 | 0.009 | YES |
| **MHC class I antigen** | A0A1X9I444 | HLA-B | -4.94 | <0.001 | <0.001 | NO |
| **HLA DR-beta-III** | Q30131 | NA | -4.83 | 0.003 | 0.043 | NO |
| **Endoplasmic reticulum DnaJ/PDI fusion protein 3** | Q6YPB0 | NA | -4.18 | <0.001 | 0.001 | NO |
| **RRBP1 protein** | A1A5C4 | RRBP1 | -3.79 | <0.001 | <0.001 | NO |
| **Mitogen-activated protein kinase kinase kinase 5 (EC 2.7.11.25) (Apoptosis signal-regulating kinase 1) (ASK-1) (MAPK/ERK kinase kinase 5) (MEK kinase 5) (MEKK 5)** | Q99683 | MAP3K5 | -2.99 | 0.003 | 0.044 | NO |
| **Aminopeptidase N (EC 3.4.11.2)** | B4DPH5 | NA | -2.83 | <0.001 | 0.005 | NO |
| **Cytochrome b** | Q6VHB3 | CYTB | -2.82 | <0.001 | 0.010 | NO |
| **MHC class I antigen** | A0A0S4T3R3 | HLA-C | -2.80 | <0.001 | 0.014 | YES |
| **Leukocyte immunoglobulin-like receptor subfamily B member 5 (CD85 antigen-like family member C) (Leukocyte immunoglobulin-like receptor 8) (LIR-8) (CD antigen CD85c)** | O75023 | LILRB5 | -2.71 | <0.001 | <0.001 | NO |
| **Endoplasmic reticulum aminopeptidase 2 (EC 3.4.11.-) (Leukocyte-derived arginine aminopeptidase) (L-RAP)** | Q6P179 | ERAP2 | -2.71 | <0.001 | <0.001 | NO |
| **Glutathione S-transferase Mu 4 (EC 2.5.1.18) (GST class-mu 4) (GST-Mu2) (GSTM4-4) (Leukotriene C4 synthase GSTM4) (EC 4.4.1.20)** | Q03013 | GSTM4 | -2.69 | <0.001 | 0.001 | NO |
| **HEAT repeat-containing protein 3 (Symportin Syo1) (hsSyo1)** | Q7Z4Q2 | HEATR3 | -2.68 | 0.001 | 0.022 | NO |
| **Tubulin polymerization-promoting protein family member 3 (TPPP/p20)** | Q9BW30 | TPPP3 | -2.63 | <0.001 | 0.001 | NO |
| **Ig-like domain-containing protein** | B7ZW57 | NA | -2.56 | <0.001 | 0.012 | NO |
| **Neprilysin (EC 3.4.24.11) (Atriopeptidase) (Common acute lymphocytic leukemia antigen) (CALLA) (Enkephalinase) (Neutral endopeptidase 24.11) (NEP) (Neutral endopeptidase) (Skin fibroblast elastase) (SFE) (CD antigen CD10)** | P08473 | MME | -2.29 | <0.001 | <0.001 | NO |
| **Fatty acid-binding protein 5 (Epidermal-type fatty acid-binding protein) (E-FABP) (Fatty acid-binding protein, epidermal) (Psoriasis-associated fatty acid-binding protein homolog) (PA-FABP)** | Q01469 | FABP5 | -2.27 | <0.001 | 0.004 | NO |
| **MHC class I antigen** | A0A024F8H3 | HLA-B | -2.26 | <0.001 | <0.001 | NO |
| **Ephrin-B1 (EFL-3) (ELK ligand) (ELK-L) (EPH-related receptor tyrosine kinase ligand 2) (LERK-2) [Cleaved into: Ephrin-B1 C-terminal fragment (Ephrin-B1 CTF); Ephrin-B1 intracellular domain (Ephrin-B1 ICD)]** | P98172 | EFNB1 | -2.22 | <0.001 | <0.001 | NO |
| **MHC class II antigen** | K4RIC6 | HLA-DRB1 | -2.16 | 0.003 | 0.044 | NO |
| **Elongator complex protein 5 (Dermal papilla-derived protein 6) (S-phase 2 protein)** | Q8TE02 | ELP5 | -2.08 | 0.003 | 0.039 | NO |
| **Glutathione S-transferase theta-2B (EC 2.5.1.18) (Glutathione S-transferase theta-2) (GST class-theta-2)** | P0CG30 | GSTT2B | -2.07 | <0.001 | 0.004 | NO |
| **Aldehyde oxidase (EC 1.2.3.1) (Aldehyde oxidase 1) (Azaheterocycle hydroxylase) (EC 1.17.3.-)** | Q06278 | AOX1 | -1.94 | <0.001 | 0.005 | NO |
| **NA** | P01889 | NA | -1.93 | <0.001 | 0.010 | NO |
| **Platelet glycoprotein Ib beta chain (GP-Ib beta) (GPIb-beta) (GPIbB) (Antigen CD42b-beta) (CD antigen CD42c)** | P13224 | GP1BB | -1.83 | <0.001 | 0.017 | NO |
| **Aspartoacylase (EC 3.5.1.15) (Aminoacylase-2) (ACY-2)** | P45381 | ASPA | -1.80 | <0.001 | 0.008 | NO |
| **MHC class I antigen** | Q2LE77 | HLA-Cw | -1.78 | 0.001 | 0.024 | NO |
| **cDNA FLJ55458, highly similar to Programmed cell death 6-interacting protein** | B4DHD2 | NA | -1.77 | 0.002 | 0.030 | NO |
| **Neurofilament medium polypeptide (160 kDa neurofilament protein) (Neurofilament 3) (Neurofilament triplet M protein)** | A5YM63 | NEFM | -1.75 | <0.001 | 0.010 | YES |
| **Zinc finger RNA-binding protein (hZFR) (M-phase phosphoprotein homolog)** | Q96KR1 | ZFR | -1.73 | 0.001 | 0.025 | YES |
| **Pseudouridylate synthase TRUB1 (EC 5.4.99.-) (TruB pseudouridine synthase homolog 1) (tRNA pseudouridine 55 synthase TRUB1) (Psi55 synthase TRUB1) (EC 5.4.99.25)** | Q8WWH5 | TRUB1 | -1.72 | 0.002 | 0.032 | NO |
| **Alpha-N-acetylglucosaminidase (EC 3.2.1.50) (N-acetyl-alpha-glucosaminidase) (NAG) [Cleaved into: Alpha-N-acetylglucosaminidase 82 kDa form; Alpha-N-acetylglucosaminidase 77 kDa form]** | P54802 | NAGLU | -1.70 | <0.001 | <0.001 | NO |
| **Cysteine-rich protein 1 (CRP-1) (Cysteine-rich heart protein) (CRHP) (hCRHP) (Cysteine-rich intestinal protein) (CRIP)** | P50238 | CRIP1 | -1.66 | 0.004 | 0.048 | NO |
| **Glutathione S-transferase Mu 5 (EC 2.5.1.18) (GST class-mu 5) (GSTM5-5)** | P46439 | GSTM5 | -1.63 | <0.001 | 0.013 | NO |
| **Scavenger receptor class A member 5 (Scavenger receptor hlg)** | Q6ZMJ2 | SCARA5 | -1.62 | <0.001 | 0.013 | NO |
| **Fatty acid-binding protein, heart (Fatty acid-binding protein 3) (Heart-type fatty acid-binding protein) (H-FABP) (Mammary-derived growth inhibitor) (MDGI) (Muscle fatty acid-binding protein) (M-FABP)** | P05413 | FABP3 | -1.58 | 0.001 | 0.026 | NO |
| **Aminopeptidase B (AP-B) (EC 3.4.11.6) (Arginine aminopeptidase) (Arginyl aminopeptidase)** | Q9H4A4 | RNPEP | -1.58 | <0.001 | <0.001 | NO |
| **Leukocyte elastase inhibitor (LEI) (Monocyte/neutrophil elastase inhibitor) (EI) (M/NEI) (Peptidase inhibitor 2) (PI-2) (Serpin B1)** | P30740 | SERPINB1 | -1.57 | <0.001 | 0.005 | NO |
| **Endosialin (Tumor endothelial marker 1) (CD antigen CD248)** | Q9HCU0 | CD248 | -1.56 | <0.001 | <0.001 | YES |
| **cDNA FLJ55560, highly similar to Retinal dehydrogenase 2** | B4DH89 | NA | -1.56 | <0.001 | 0.002 | NO |
| **Angiotensin-converting enzyme (ACE) (EC 3.4.15.1) (Dipeptidyl carboxypeptidase I) (Kininase II) (CD antigen CD143) [Cleaved into: Angiotensin-converting enzyme, soluble form]** | P12821 | ACE | -1.53 | <0.001 | 0.002 | NO |
| **Cofilin 1** | E9PQB7 | CFL1 | -1.50 | <0.001 | 0.020 | NO |
| **Ribose-phosphate pyrophosphokinase 2 (EC 2.7.6.1) (PPRibP) (Phosphoribosyl pyrophosphate synthase II) (PRS-II)** | P11908 | PRPS2 | -1.50 | <0.001 | <0.001 | NO |
| **cGMP-dependent 3',5'-cyclic phosphodiesterase (EC 3.1.4.17) (Cyclic GMP-stimulated phosphodiesterase) (CGS-PDE) (cGSPDE)** | O00408 | PDE2A | -1.49 | <0.001 | 0.012 | NO |
| **Nondiscriminating glutamyl-tRNA synthetase EARS2, mitochondrial (EC 6.1.1.24) (Glutamate--tRNA(Gln) ligase EARS2, mitochondrial) (EC 6.1.1.17) (Glutamyl-tRNA synthetase) (GluRS) (Mitochondrial glutamyl-tRNA synthetase) (mtGluRS)** | Q5JPH6 | EARS2 | -1.49 | <0.001 | 0.005 | NO |
| **Actin remodeling regulator NHS (Congenital cataracts and dental anomalies protein) (Nance-Horan syndrome protein)** | Q6T4R5 | NHS | -1.49 | <0.001 | 0.014 | YES |
| **MHC Class I antigen** | G9I2K8 | HLA-B | -1.48 | <0.001 | 0.014 | NO |
| **Multidrug and toxin extrusion protein 1 (MATE-1) (hMATE-1) (Solute carrier family 47 member 1)** | Q96FL8 | SLC47A1 | -1.48 | <0.001 | 0.005 | NO |
| **Actin filament-associated protein 1-like 2 (AFAP1-like protein 2)** | Q8N4X5 | AFAP1L2 | -1.48 | <0.001 | 0.009 | NO |
| **Peptidyl-prolyl cis-trans isomerase-like 3 (PPIase) (EC 5.2.1.8) (Cyclophilin J) (CyPJ) (Cyclophilin-like protein PPIL3) (Rotamase PPIL3)** | Q9H2H8 | PPIL3 | -1.45 | <0.001 | 0.003 | NO |
| **Sulfotransferase** | A0A024QZB4 | hCG_1993905 | -1.45 | 0.002 | 0.030 | NO |
| **Insulin-like growth factor-binding protein 5 (IBP-5) (IGF-binding protein 5) (IGFBP-5)** | P24593 | IGFBP5 | -1.45 | <0.001 | 0.006 | YES |
| **Inactive phospholipase C-like protein 1 (PLC-L1) (Phospholipase C-deleted in lung carcinoma) (Phospholipase C-related but catalytically inactive protein) (PRIP)** | Q15111 | PLCL1 | -1.45 | <0.001 | 0.002 | YES |
| **Tubulin polymerization-promoting protein (TPPP) (EC 3.6.5.-) (25 kDa brain-specific protein) (TPPP/p25) (p24) (p25-alpha)** | O94811 | TPPP | -1.44 | <0.001 | 0.005 | NO |
| **Lymphatic vessel endothelial hyaluronic acid receptor 1 (LYVE-1) (Cell surface retention sequence-binding protein 1) (CRSBP-1) (Extracellular link domain-containing protein 1) (Hyaluronic acid receptor)** | Q9Y5Y7 | LYVE1 | -1.44 | <0.001 | 0.003 | NO |
| **NAD(P)H dehydrogenase [quinone] 1 (EC 1.6.5.2) (Azoreductase) (DT-diaphorase) (DTD) (Menadione reductase) (NAD(P)H:quinone oxidoreductase 1) (Phylloquinone reductase) (Quinone reductase 1) (QR1)** | P15559 | NQO1 | -1.43 | <0.001 | 0.014 | NO |
| **Protein S100-A4 (Calvasculin) (Metastasin) (Placental calcium-binding protein) (Protein Mts1) (S100 calcium-binding protein A4)** | P26447 | S100A4 | -1.41 | <0.001 | 0.003 | NO |
| **A-kinase anchor protein 7 isoform gamma (AKAP-7 isoform gamma) (A-kinase anchor protein 18 kDa) (AKAP 18) (Protein kinase A-anchoring protein 7 isoform gamma) (PRKA7 isoform gamma)** | Q9P0M2 | AKAP7 | -1.40 | 0.001 | 0.026 | NO |
| **Glycogen phosphorylase, brain form (EC 2.4.1.1)** | P11216 | PYGB | -1.40 | <0.001 | 0.015 | NO |
| **Methanethiol oxidase (MTO) (EC 1.8.3.4) (56 kDa selenium-binding protein) (SBP56) (SP56) (Selenium-binding protein 1)** | Q13228 | SELENBP1 | -1.40 | <0.001 | 0.008 | NO |
| **Transforming growth factor beta receptor type 3 (TGF-beta receptor type 3) (TGFR-3) (Betaglycan) (Transforming growth factor beta receptor III) (TGF-beta receptor type III)** | Q03167 | TGFBR3 | -1.39 | 0.002 | 0.035 | NO |
| **Calcitonin gene-related peptide type 1 receptor (CGRP type 1 receptor) (Calcitonin receptor-like receptor) (CRLR)** | Q16602 | CALCRL | -1.37 | 0.001 | 0.024 | NO |
| **Carbonic anhydrase 12 (EC 4.2.1.1) (Carbonate dehydratase XII) (Carbonic anhydrase XII) (CA-XII) (Tumor antigen HOM-RCC-3.1.3)** | O43570 | CA12 | -1.36 | <0.001 | 0.014 | NO |
| **E3 ubiquitin-protein ligase TRIM32 (EC 2.3.2.27) (72 kDa Tat-interacting protein) (RING-type E3 ubiquitin transferase TRIM32) (Tripartite motif-containing protein 32) (Zinc finger protein HT2A)** | Q13049 | TRIM32 | -1.36 | 0.002 | 0.031 | NO |
| **Solute carrier organic anion transporter family member 2A1 (SLCO2A1) (OATP2A1) (PHOAR2) (Prostaglandin transporter) (PGT) (Solute carrier family 21 member 2) (SLC21A2)** | Q92959 | SLCO2A1 | -1.33 | <0.001 | <0.001 | NO |
| **Proteasome subunit beta type-8 (EC 3.4.25.1) (Low molecular mass protein 7) (Macropain subunit C13) (Multicatalytic endopeptidase complex subunit C13) (Proteasome component C13) (Proteasome subunit beta-5i)** | P28062 | PSMB8 | -1.33 | 0.003 | 0.042 | NO |
| **Nuclear factor 1 C-type (NF1-C) (Nuclear factor 1/C) (CCAAT-box-binding transcription factor) (CTF) (Nuclear factor I/C) (NF-I/C) (NFI-C) (TGGCA-binding protein)** | P08651 | NFIC | -1.33 | <0.001 | 0.010 | YES |
| **Poly(rC)-binding protein 3 (Alpha-CP3) (PCBP3-overlapping transcript) (PCBP3-overlapping transcript 1)** | P57721 | PCBP3 | -1.32 | 0.003 | 0.044 | NO |
| **Ribosyldihydronicotinamide dehydrogenase [quinone] (EC 1.10.5.1) (NRH dehydrogenase [quinone] 2) (NRH:quinone oxidoreductase 2) (Quinone reductase 2) (QR2)** | P16083 | NQO2 | -1.32 | <0.001 | 0.011 | NO |
| **Aminopeptidase N (AP-N) (hAPN) (EC 3.4.11.2) (Alanyl aminopeptidase) (Aminopeptidase M) (AP-M) (Microsomal aminopeptidase) (Myeloid plasma membrane glycoprotein CD13) (gp150) (CD antigen CD13)** | P15144 | ANPEP | -1.32 | 0.002 | 0.034 | NO |
| **Forkhead box protein O1 (Forkhead box protein O1A) (Forkhead in rhabdomyosarcoma)** | Q12778 | FOXO1 | -1.29 | <0.001 | <0.001 | YES |
| **Galactose mutarotase (EC 5.1.3.3) (Aldose 1-epimerase)** | Q96C23 | GALM | -1.28 | <0.001 | 0.003 | NO |
| **Putative monooxygenase p33MONOX (EC 1.-.-.-) (Brain-derived rescue factor p60MONOX) (Flavin monooxygenase motif-containing protein of 33 kDa)** | Q96A73 | KIAA1191 | -1.28 | <0.001 | 0.002 | NO |
| **Aldehyde dehydrogenase family 3 member B1 (EC 1.2.1.28) (EC 1.2.1.5) (EC 1.2.1.7) (Aldehyde dehydrogenase 7) (Long-chain fatty aldehyde dehydrogenase) (EC 1.2.1.48) (Medium-chain fatty aldehyde dehydrogenase)** | P43353 | ALDH3B1 | -1.27 | 0.002 | 0.029 | NO |
| **Erythrocyte membrane protein band 4.1 like 3 (cDNA FLJ77757)** | A8K968 | EPB41L3 | -1.27 | <0.001 | 0.016 | NO |
| **Sodium/hydrogen exchanger 9 (Na(+)/H(+) exchanger 9) (NHE-9) (Solute carrier family 9 member 9)** | Q8IVB4 | SLC9A9 | -1.26 | <0.001 | 0.011 | NO |
| **Parathymosin** | P20962 | PTMS | -1.26 | <0.001 | 0.002 | NO |
| **COUP transcription factor 2 (COUP-TF2) (Apolipoprotein A-I regulatory protein 1) (ARP-1) (COUP transcription factor II) (COUP-TF II) (Nuclear receptor subfamily 2 group F member 2)** | P24468 | NR2F2 | -1.26 | <0.001 | 0.021 | NO |
| **Dipeptidyl peptidase 4 (EC 3.4.14.5) (ADABP) (Adenosine deaminase complexing protein 2) (ADCP-2) (Dipeptidyl peptidase IV) (DPP IV) (T-cell activation antigen CD26) (TP103) (CD antigen CD26) [Cleaved into: Dipeptidyl peptidase 4 membrane form (Dipeptidyl peptidase IV membrane form); Dipeptidyl peptidase 4 soluble form (Dipeptidyl peptidase IV soluble form)]** | P27487 | DPP4 | -1.25 | 0.001 | 0.022 | NO |
| **Oxidoreductase HTATIP2 (EC 1.1.1.-) (30 kDa HIV-1 TAT-interacting protein) (HIV-1 TAT-interactive protein 2)** | Q9BUP3 | HTATIP2 | -1.25 | <0.001 | 0.001 | NO |
| **A-kinase anchor protein 12 (AKAP-12) (A-kinase anchor protein 250 kDa) (AKAP 250) (Gravin) (Myasthenia gravis autoantigen)** | Q02952 | AKAP12 | -1.24 | <0.001 | <0.001 | YES |
| **Phosphatase and actin regulator 2** | O75167 | PHACTR2 | -1.23 | <0.001 | 0.005 | NO |
| **Receptor-type tyrosine-protein phosphatase gamma (Protein-tyrosine phosphatase gamma) (R-PTP-gamma) (EC 3.1.3.48)** | P23470 | PTPRG | -1.22 | 0.002 | 0.031 | NO |
| **Prostaglandin reductase 2 (PRG-2) (EC 1.3.1.48) (15-oxoprostaglandin 13-reductase) (Zinc-binding alcohol dehydrogenase domain-containing protein 1)** | Q8N8N7 | PTGR2 | -1.17 | <0.001 | 0.015 | NO |
| **Adipose-secreted signaling protein** | Q9GZN8 | ADISSP | -1.16 | 0.001 | 0.024 | NO |
| **Phosphatidylcholine transfer protein (PC-TP) (START domain-containing protein 2) (StARD2) (StAR-related lipid transfer protein 2)** | Q9UKL6 | PCTP | -1.16 | 0.001 | 0.025 | NO |
| **Butyrophilin subfamily 3 member A3** | O00478 | BTN3A3 | -1.16 | <0.001 | 0.002 | NO |
| **Proteasome subunit beta type-10 (EC 3.4.25.1) (Low molecular mass protein 10) (Macropain subunit MECl-1) (Multicatalytic endopeptidase complex subunit MECl-1) (Proteasome MECl-1) (Proteasome subunit beta-2i)** | P40306 | PSMB10 | -1.16 | 0.002 | 0.032 | NO |
| **Acid ceramidase (EC 3.5.1.23)** | A8K0B6 | NA | -1.15 | <0.001 | 0.005 | NO |
| **Mitochondrial enolase superfamily member 1 (EC 4.2.1.68) (Antisense RNA to thymidylate synthase) (rTS) (L-fuconate dehydratase)** | Q7L5Y1 | ENOSF1 | -1.15 | <0.001 | 0.008 | NO |
| **MHC class I antigen** | Q546C9 | HLA-B | -1.15 | 0.001 | 0.025 | YES |
| **Tryptase beta-2 (Tryptase-2) (EC 3.4.21.59) (Tryptase II)** | P20231 | TPSB2 | -1.15 | 0.001 | 0.025 | NO |
| **von Willebrand factor (vWF) [Cleaved into: von Willebrand antigen 2 (von Willebrand antigen II)]** | P04275 | VWF | -1.13 | <0.001 | 0.001 | NO |
| **NA** | P58107 | NA | -1.12 | <0.001 | 0.008 | NO |
| **Sphingosine 1-phosphate receptor 3 (S1P receptor 3) (S1P3) (Endothelial differentiation G-protein coupled receptor 3) (Sphingosine 1-phosphate receptor Edg-3) (S1P receptor Edg-3)** | Q99500 | S1PR3 | -1.12 | 0.001 | 0.024 | NO |
| **IgG receptor FcRn large subunit p51 (FcRn) (IgG Fc fragment receptor transporter alpha chain) (Neonatal Fc receptor)** | P55899 | FCGRT | -1.11 | <0.001 | 0.015 | NO |
| **Beta-2-microglobulin [Cleaved into: Beta-2-microglobulin form pI 5.3]** | P61769 | B2M | -1.10 | 0.003 | 0.041 | NO |
| **Peroxisomal 2,4-dienoyl-CoA reductase [(3E)-enoyl-CoA-producing] (pDCR) (EC 1.3.1.124) (2,4-dienoyl-CoA reductase 2) (Short chain dehydrogenase/reductase family 17C member 1)** | Q9NUI1 | DECR2 | -1.10 | 0.003 | 0.041 | NO |
| **AP-1 complex subunit gamma-like 2 (Gamma2-adaptin) (G2ad)** | O75843 | AP1G2 | -1.10 | <0.001 | 0.008 | NO |
| **Bifunctional epoxide hydrolase 2 [Includes: Cytosolic epoxide hydrolase 2 (CEH) (EC 3.3.2.10) (Epoxide hydratase) (Soluble epoxide hydrolase) (SEH); Lipid-phosphate phosphatase (EC 3.1.3.76)]** | P34913 | EPHX2 | -1.08 | <0.001 | 0.008 | NO |
| **D-ribitol-5-phosphate cytidylyltransferase (EC 2.7.7.40) (2-C-methyl-D-erythritol 4-phosphate cytidylyltransferase-like protein) (Isoprenoid synthase domain-containing protein) (hISPD)** | A4D126 | CRPPA | -1.07 | 0.002 | 0.034 | NO |
| **Pro-cathepsin H [Cleaved into: Cathepsin H mini chain; Cathepsin H (EC 3.4.22.16); Cathepsin H heavy chain; Cathepsin H light chain]** | P09668 | CTSH | -1.07 | 0.002 | 0.033 | NO |
| **Homeobox protein Meis1** | O00470 | MEIS1 | -1.07 | <0.001 | 0.009 | YES |
| **Activating signal cointegrator 1 complex subunit 2 (ASC-1 complex subunit p100) (Trip4 complex subunit p100)** | Q9H1I8 | ASCC2 | -1.07 | <0.001 | 0.008 | NO |
| **Transmembrane glycoprotein NMB (Hematopoietic growth factor inducible neurokinin-1 type)** | Q14956 | GPNMB | -1.07 | 0.002 | 0.036 | NO |
| **Phosphopentomutase (EC 5.4.2.7) (Glucose phosphomutase 2) (Phosphodeoxyribomutase) (Phosphoglucomutase-2) (EC 5.4.2.2)** | Q96G03 | PGM2 | -1.06 | <0.001 | 0.002 | NO |
| **Tumor necrosis factor alpha-induced protein 2 (TNF alpha-induced protein 2) (Primary response gene B94 protein)** | Q03169 | TNFAIP2 | -1.05 | <0.001 | 0.018 | YES |
| **MHC class I antigen** | F6IQY8 | HLA-A | -1.05 | 0.003 | 0.044 | NO |
| **Cadherin 11, type 2, OB-cadherin (Osteoblast)** | Q96CZ9 | CDH11 | -1.04 | <0.001 | 0.003 | NO |
| **Phospholysine phosphohistidine inorganic pyrophosphate phosphatase (hLHPP) (EC 3.1.3.-) (EC 3.6.1.1)** | Q9H008 | LHPP | -1.04 | 0.001 | 0.022 | NO |
| **Uncharacterized protein DKFZp686A1195** | Q6MZU1 | DKFZp686A1195 | -1.04 | <0.001 | 0.002 | NO |
| **Calpastatin** | D6RBR1 | CAST | -1.04 | <0.001 | 0.014 | NO |
| **Protein N-terminal asparagine amidohydrolase (EC 3.5.1.121) (Protein NH2-terminal asparagine amidohydrolase) (PNAA) (Protein NH2-terminal asparagine deamidase) (PNAD) (Protein N-terminal Asn amidase) (Protein N-terminal asparagine amidase) (Protein NTN-amidase)** | Q96AB6 | NTAN1 | -1.03 | <0.001 | 0.006 | NO |
| **SAM and SH3 domain-containing protein 1 (Proline-glutamate repeat-containing protein)** | O94885 | SASH1 | -1.03 | <0.001 | 0.006 | YES |
| **Polymerase (RNA) II (DNA directed) polypeptide K, 7.0kDa, isoform CRA_a** | A0A024R9G0 | POLR2K | -1.03 | 0.001 | 0.025 | NO |
| **EPM2A-interacting protein 1 (Laforin-interacting protein)** | Q7L775 | EPM2AIP1 | -1.01 | 0.003 | 0.039 | NO |
| **PSME3-interacting protein (NEFA-interacting nuclear protein NIP30) (PA28G-interacting protein)** | Q9GZU8 | PSME3IP1 | -1.01 | <0.001 | 0.010 | NO |
| **Thioredoxin-like protein 4A (DIM1 protein homolog) (Spliceosomal U5 snRNP-specific 15 kDa protein) (Thioredoxin-like U5 snRNP protein U5-15kD)** | P83876 | TXNL4A | -1.01 | 0.001 | 0.024 | NO |
| **PDZ and LIM domain protein 1 (C-terminal LIM domain protein 1) (Elfin) (LIM domain protein CLP-36)** | O00151 | PDLIM1 | -1.01 | 0.001 | 0.025 | NO |
| **Pre-B-cell leukemia transcription factor 1 (Homeobox protein PBX1) (Homeobox protein PRL)** | P40424 | PBX1 | -1.00 | <0.001 | 0.014 | NO |
| **Inactive C-alpha-formylglycine-generating enzyme 2 (Paralog of formylglycine-generating enzyme) (pFGE) (Sulfatase-modifying factor 2)** | Q8NBJ7 | SUMF2 | -1.00 | <0.001 | 0.021 | NO |
| **Protein-glutamine gamma-glutamyltransferase 2 (EC 2.3.2.13) (Erythrocyte transglutaminase) (Heart G alpha(h)) (hhG alpha(h)) (Isopeptidase TGM2) (EC 3.4.-.-) (Protein G alpha(h)) (G(h)) (Protein-glutamine deamidase TGM2) (EC 3.5.1.44) (Protein-glutamine dopaminyltransferase TGM2) (EC 2.3.1.-) (Protein-glutamine histaminyltransferase TGM2) (EC 2.3.1.-) (Protein-glutamine noradrenalinyltransferase TGM2) (EC 2.3.1.-) (Protein-glutamine serotonyltransferase TGM2) (EC 2.3.1.-) (Tissue transglutaminase) (tTG) (tTgase) (Transglutaminase C) (TG(C)) (TGC) (TGase C) (Transglutaminase H) (TGase H) (Transglutaminase II) (TGase II) (Transglutaminase-2) (TG2) (TGase-2) (hTG2)** | P21980 | TGM2 | -1.00 | 0.002 | 0.028 | NO |
| **Clustered mitochondria protein homolog** | I3L2B0 | CLUH | -1.00 | <0.001 | <0.001 | NO |
| **Protein phosphatase inhibitor 2 (IPP-2)** | P41236 | PPP1R2 | -1.00 | 0.001 | 0.023 | YES |

**Supplementary Information 6:** Total proteins which significantly (p≤0.05) at least doubled and passed FDR (q≤0.05) in the failed IOL group compared with the elCS group under the contracted-phase during spontaneous contractions condition, ordered by log-fold change.

|  |  |  |  |  |  |  |
| --- | --- | --- | --- | --- | --- | --- |
| **Protein name** | **Accession** | **Gene name** | **Log Fold-change** | **p-value** | **q-value** | **Phosphorylation identified** |
| **maleylacetoacetate isomerase (EC 5.2.1.2)** | G3V4T6 | GSTZ1 | 5.61 | <0.001 | <0.001 | NO |
| **Large ribosomal subunit protein eL14 (60S ribosomal protein L14)** | Q6IPH7 | RPL14 | 5.41 | <0.001 | <0.001 | NO |
| **Desmuslin, isoform CRA_a (Synemin)** | A0A075B7B1 | SYNM | 4.94 | <0.001 | 0.014 | NO |
| **Parvalbumin** | H0Y3U0 | PVALB | 4.67 | <0.001 | 0.028 | NO |
| **Ankyrin-3 (ANK-3) (Ankyrin-G)** | Q12955 | ANK3 | 4.32 | <0.001 | 0.032 | NO |
| **Zinc finger, UBR1 type 1, isoform CRA_c** | A0A024RAC9 | ZUBR1 | 4.11 | <0.001 | 0.002 | NO |
| **Cartilage oligomeric matrix protein (COMP) (Thrombospondin-5) (TSP5)** | P49747 | COMP | 4.04 | <0.001 | 0.002 | NO |
| **Calpain-2 catalytic subunit (EC 3.4.22.53) (Calcium-activated neutral proteinase 2) (Calpain M-type) (Calpain-2 large subunit) (Millimolar-calpain)** | B2RCM3 | NA | 3.82 | <0.001 | 0.003 | NO |
| **Thrombospondin-4** | P35443 | THBS4 | 3.76 | <0.001 | <0.001 | NO |
| **Carbonic anhydrase III, muscle specific, isoform CRA_a** | A0A024R825 | CA3 | 3.16 | <0.001 | 0.003 | NO |
| **Immunoglobulin heavy constant gamma 4 (Ig gamma-4 chain C region)** | P01861 | IGHG4 | 3.14 | <0.001 | 0.006 | NO |
| **N90-VRC38.08 heavy chain variable region** | A0A1W6IYI5 | NA | 2.98 | <0.001 | <0.001 | NO |
| **Myosin light chain 1/3, skeletal muscle isoform (MLC1/MLC3) (MLC1F/MLC3F) (Myosin light chain alkali 1/2) (Myosin light chain A1/A2)** | P05976 | MYL1 | 2.91 | 0.002 | 0.048 | NO |
| **Alkaline phosphatase, tissue-nonspecific isozyme (AP-TNAP) (TNS-ALP) (TNSALP) (EC 3.1.3.1) (Alkaline phosphatase liver/bone/kidney isozyme) (Phosphoamidase) (Phosphocreatine phosphatase) (EC 3.9.1.1)** | P05186 | ALPL | 2.86 | <0.001 | <0.001 | NO |
| **Matrix Gla protein (MGP) (Cell growth-inhibiting gene 36 protein)** | P08493 | MGP | 2.76 | <0.001 | <0.001 | NO |
| **Midkine (MK) (Amphiregulin-associated protein) (ARAP) (Midgestation and kidney protein) (Neurite outgrowth-promoting factor 2) (Neurite outgrowth-promoting protein)** | P21741 | MDK | 2.75 | <0.001 | 0.032 | NO |
| **Annexin A3 (35-alpha calcimedin) (Annexin III) (Annexin-3) (Inositol 1,2-cyclic phosphate 2-phosphohydrolase) (Lipocortin III) (Placental anticoagulant protein III) (PAP-III)** | P12429 | ANXA3 | 2.72 | <0.001 | <0.001 | NO |
| **Matrilin 2, isoform CRA_b** | A0A024R9B9 | MATN2 | 2.61 | <0.001 | 0.025 | NO |
| **Thrombospondin-2** | P35442 | THBS2 | 2.58 | <0.001 | 0.006 | NO |
| **TNC variant protein** | Q4LE33 | TNC variant protein | 2.57 | <0.001 | <0.001 | YES |
| **Transforming growth factor-beta-induced protein ig-h3 (Beta ig-h3) (Kerato-epithelin) (RGD-containing collagen-associated protein) (RGD-CAP)** | Q15582 | TGFBI | 2.50 | <0.001 | <0.001 | NO |
| **C-type lectin domain family 11 member A (C-type lectin superfamily member 3) (Lymphocyte secreted C-type lectin) (Osteolectin) (Stem cell growth factor) (p47)** | Q9Y240 | CLEC11A | 2.39 | <0.001 | 0.016 | NO |
| **Thrombospondin 1, isoform CRA_a** | A0A024R9Q1 | THBS1 | 2.34 | <0.001 | 0.004 | NO |
| **Secreted frizzled-related protein 1 (FRP-1) (sFRP-1) (Secreted apoptosis-related protein 2) (SARP-2)** | Q8N474 | SFRP1 | 2.13 | <0.001 | 0.001 | NO |
| **IGH@ protein** | Q6P089 | IGH@ | 2.13 | <0.001 | 0.018 | NO |
| **Transforming growth factor beta-2 proprotein (Cetermin) (Glioblastoma-derived T-cell suppressor factor) (G-TSF) [Cleaved into: Latency-associated peptide (LAP); Transforming growth factor beta-2 (TGF-beta-2)]** | P61812 | TGFB2 | 2.12 | <0.001 | 0.004 | NO |
| **PG-M** | Q6MZK8 | DKFZp686K06110 | 2.09 | <0.001 | 0.027 | YES |
| **Myosin-reactive immunoglobulin heavy chain variable region** | Q9UL90 | NA | 2.08 | <0.001 | 0.008 | NO |
| **Unconventional myosin-If (Myosin-Ie)** | O00160 | MYO1F | 2.07 | <0.001 | 0.030 | NO |
| **Serine racemase (EC 5.1.1.18) (D-serine ammonia-lyase) (D-serine dehydratase) (EC 4.3.1.18) (L-serine ammonia-lyase) (L-serine dehydratase) (EC 4.3.1.17)** | Q9GZT4 | SRR | 2.05 | <0.001 | 0.016 | NO |
| **D-3-phosphoglycerate dehydrogenase (3-PGDH) (EC 1.1.1.95) (2-oxoglutarate reductase) (EC 1.1.1.399) (Malate dehydrogenase) (EC 1.1.1.37)** | O43175 | PHGDH | 2.04 | <0.001 | 0.013 | NO |
| **Biglycan** | C9JKG1 | BGN | 1.99 | 0.001 | 0.040 | NO |
| **Peptidase inhibitor 16 (PI-16) (Cysteine-rich secretory protein 9) (CRISP-9) (PSP94-binding protein) (CD antigen CD364)** | Q6UXB8 | PI16 | 1.96 | <0.001 | 0.030 | NO |
| **Matrilin-2** | O00339 | MATN2 | 1.96 | <0.001 | 0.002 | NO |
| **Serpin peptidase inhibitor, clade E (Nexin, plasminogen activator inhibitor type 1), member 1, isoform CRA_b** | A0A024QYT5 | SERPINE1 | 1.96 | <0.001 | 0.004 | NO |
| **Cadherin 2, type 1, N-cadherin (Neuronal), isoform CRA_b** | A0A024RC42 | CDH2 | 1.95 | <0.001 | <0.001 | NO |
| **Dual adapter for phosphotyrosine and 3-phosphotyrosine and 3-phosphoinositide (hDAPP1) (B lymphocyte adapter protein Bam32) (B-cell adapter molecule of 32 kDa)** | Q9UN19 | DAPP1 | 1.95 | <0.001 | 0.006 | NO |
| **CCN family member 4 (WNT1-inducible-signaling pathway protein 1) (WISP-1) (Wnt-1-induced secreted protein)** | O95388 | CCN4 | 1.94 | <0.001 | 0.001 | NO |
| **Solute carrier family 16 (Monocarboxylic acid transporters), member 3, isoform CRA_a** | A0A024R8U1 | SLC16A3 | 1.93 | <0.001 | 0.016 | NO |
| **peptidylprolyl isomerase (EC 5.2.1.8)** | Q6ZR21 | NA | 1.92 | 0.002 | 0.048 | NO |
| **Lysyl oxidase homolog 2 (EC 1.4.3.13) (Lysyl oxidase-like protein 2) (Lysyl oxidase-related protein 2) (Lysyl oxidase-related protein WS9-14)** | Q9Y4K0 | LOXL2 | 1.82 | <0.001 | <0.001 | NO |
| **SH3 domain-binding glutamic acid-rich-like protein 2 (Fovea-associated SH3 domain-binding protein)** | Q9UJC5 | SH3BGRL2 | 1.82 | <0.001 | <0.001 | NO |
| **Apolipoprotein(a) (Apo(a)) (Lp(a)) (EC 3.4.21.-)** | P08519 | LPA | 1.79 | 0.001 | 0.035 | NO |
| **Uncharacterized protein DKFZp686K18196** | Q6N092 | DKFZp686K18196 | 1.74 | <0.001 | 0.024 | NO |
| **Prostaglandin G/H synthase 1 (EC 1.14.99.1) (Cyclooxygenase-1) (COX-1) (Prostaglandin H2 synthase 1) (PGH synthase 1) (PGHS-1) (PHS 1) (Prostaglandin-endoperoxide synthase 1)** | P23219 | PTGS1 | 1.74 | <0.001 | 0.002 | NO |
| **Cytoplasmic dynein 2 heavy chain 1 (Cytoplasmic dynein 2 heavy chain) (Dynein cytoplasmic heavy chain 2) (Dynein heavy chain 11) (hDHC11) (Dynein heavy chain isotype 1B)** | Q8NCM8 | DYNC2H1 | 1.72 | 0.002 | 0.048 | NO |
| **Transmembrane protein 14C** | Q53F27 | NA | 1.70 | <0.001 | 0.013 | NO |
| **Haptoglobin (Zonulin) [Cleaved into: Haptoglobin alpha chain; Haptoglobin beta chain]** | P00738 | HP | 1.70 | <0.001 | 0.011 | NO |
| **Biglycan (Bone/cartilage proteoglycan I) (PG-S1)** | P21810 | BGN | 1.69 | <0.001 | 0.013 | NO |
| **Immunoglobulin heavy variable 1-69 (Ig heavy chain V-I region EU) (Ig heavy chain V-I region SIE) (Ig heavy chain V-I region WOL)** | P01742 | IGHV1-69 | 1.68 | 0.001 | 0.043 | NO |
| **Collagen alpha-1(XV) chain [Cleaved into: Restin (Endostatin-XV) (Related to endostatin) (Restin-I); Restin-2 (Restin-II); Restin-3 (Restin-III); Restin-4 (Restin-IV)]** | P39059 | COL15A1 | 1.66 | <0.001 | 0.011 | NO |
| **Tenascin-X (TN-X) (Hexabrachion-like protein)** | P22105 | TNXB | 1.65 | <0.001 | 0.001 | YES |
| **Ribonuclease T2 (EC 4.6.1.19) (Ribonuclease 6)** | O00584 | RNASET2 | 1.65 | <0.001 | 0.003 | NO |
| **Uncharacterized protein DKFZp686M0562** | Q6MZL2 | DKFZp686M0562 | 1.65 | 0.002 | 0.046 | NO |
| **Collagen alpha-1(XII) chain** | Q99715 | COL12A1 | 1.62 | <0.001 | 0.028 | NO |
| **[Pyruvate dehydrogenase (acetyl-transferring)] kinase isozyme 1, mitochondrial (EC 2.7.11.2) (Pyruvate dehydrogenase kinase isoform 1) (PDH kinase 1)** | Q15118 | PDK1 | 1.58 | <0.001 | 0.010 | NO |
| **MHC class II antigen** | A0A1Y6MRR4 | HLA-DPB1 | 1.58 | 0.001 | 0.038 | NO |
| **C14orf159 variant protein** | Q4LE40 | C14orf159 variant protein | 1.54 | <0.001 | <0.001 | NO |
| **SAA2-SAA4 readthrough** | A0A096LPE2 | SAA2-SAA4 | 1.53 | <0.001 | 0.002 | NO |
| **Septin-6** | Q14141 | SEPTIN6 | 1.51 | <0.001 | <0.001 | NO |
| **Fibrillin-2 [Cleaved into: Placensin]** | P35556 | FBN2 | 1.50 | 0.002 | 0.046 | NO |
| **Polypeptide N-acetylgalactosaminyltransferase 18 (EC 2.4.1.41) (Polypeptide GalNAc transferase 18) (GalNAc-T18) (Polypeptide GalNAc transferase-like protein 4) (GalNAc-T-like protein 4) (pp-GaNTase-like protein 4) (Polypeptide N-acetylgalactosaminyltransferase-like protein 4) (Protein-UDP acetylgalactosaminyltransferase-like protein 4) (UDP-GalNAc:polypeptide N-acetylgalactosaminyltransferase-like protein 4)** | Q6P9A2 | GALNT18 | 1.50 | <0.001 | 0.018 | NO |
| **Diphosphoinositol polyphosphate phosphohydrolase 3-alpha (DIPP-3-alpha) (DIPP3-alpha) (hDIPP3alpha) (EC 3.6.1.52) (Diadenosine 5',5'''-P1,P6-hexaphosphate hydrolase 3-alpha) (Diadenosine hexaphosphate hydrolase (AMP-forming)) (EC 3.6.1.60) (Nucleoside diphosphate-linked moiety X motif 10) (Nudix motif 10) (hAps2)** | Q8NFP7 | NUDT10 | 1.49 | <0.001 | 0.015 | NO |
| **Myosin-reactive immunoglobulin light chain variable region** | Q9UL82 | NA | 1.47 | 0.002 | 0.046 | NO |
| **NADH:ubiquinone oxidoreductase MLRQ subunit homolog, isoform CRA_a** | A0A024RB39 | LOC56901 | 1.45 | <0.001 | 0.014 | NO |
| **Solute carrier family 2, facilitated glucose transporter member 4 (Glucose transporter type 4, insulin-responsive) (GLUT-4)** | P14672 | SLC2A4 | 1.44 | <0.001 | 0.018 | NO |
| **Neural cell adhesion molecule 1** | A0A087WTF6 | NCAM1 | 1.44 | <0.001 | 0.005 | NO |
| **Inter-alpha-trypsin inhibitor heavy chain H1 (ITI heavy chain H1) (ITI-HC1) (Inter-alpha-inhibitor heavy chain 1) (Inter-alpha-trypsin inhibitor complex component III) (Serum-derived hyaluronan-associated protein) (SHAP)** | P19827 | ITIH1 | 1.43 | <0.001 | 0.001 | NO |
| **Latent-transforming growth factor beta-binding protein 2 (LTBP-2)** | Q14767 | LTBP2 | 1.40 | <0.001 | 0.008 | NO |
| **Prostacyclin synthase (EC 5.3.99.4) (Hydroperoxy icosatetraenoate dehydratase) (EC 4.2.1.152) (Prostaglandin I2 synthase)** | Q16647 | PTGIS | 1.39 | <0.001 | 0.002 | NO |
| **Vitamin D binding protein** | A0A1B1CYC5 | Gc | 1.38 | <0.001 | 0.010 | NO |
| **Zinc finger FYVE domain-containing protein 26 (FYVE domain-containing centrosomal protein) (FYVE-CENT) (Spastizin)** | Q68DK2 | ZFYVE26 | 1.32 | 0.001 | 0.038 | NO |
| **Fibroleukin (Fibrinogen-like protein 2) (pT49)** | Q14314 | FGL2 | 1.32 | <0.001 | 0.030 | NO |
| **Serine/threonine-protein kinase 10 (EC 2.7.11.1) (Lymphocyte-oriented kinase)** | O94804 | STK10 | 1.31 | <0.001 | 0.024 | YES |
| **Adipocyte enhancer-binding protein 1 (AE-binding protein 1) (Aortic carboxypeptidase-like protein)** | Q8IUX7 | AEBP1 | 1.30 | <0.001 | 0.022 | NO |
| **Matrix metalloproteinase-19 (MMP-19) (EC 3.4.24.-) (Matrix metalloproteinase RASI) (Matrix metalloproteinase-18) (MMP-18)** | Q99542 | MMP19 | 1.30 | <0.001 | 0.011 | NO |
| **Asporin (Periodontal ligament-associated protein 1) (PLAP-1)** | Q9BXN1 | ASPN | 1.30 | 0.002 | 0.045 | NO |
| **Protein AMBP (Protein HC) [Cleaved into: Alpha-1-microglobulin (EC 1.6.2.-) (Alpha-1 microglycoprotein) (Complex-forming glycoprotein heterogeneous in charge); Inter-alpha-trypsin inhibitor light chain (ITI-LC) (Bikunin) (EDC1) (HI-30) (Uronic-acid-rich protein); Trypstatin]** | P02760 | AMBP | 1.27 | <0.001 | 0.024 | NO |
| **Dihydropyrimidinase-related protein 1 (DRP-1) (Collapsin response mediator protein 1) (CRMP-1) (Inactive dihydropyrimidinase) (Unc-33-like phosphoprotein 3) (ULIP-3)** | Q14194 | CRMP1 | 1.26 | <0.001 | 0.028 | NO |
| **IgG L chain** | S6B2A1 | NA | 1.24 | <0.001 | 0.030 | NO |
| **cDNA FLJ51896, highly similar to Glia-derived nexin** | B4DMR3 | NA | 1.23 | 0.002 | 0.046 | NO |
| **Corrinoid adenosyltransferase MMAB (EC 2.5.1.-) (ATP:co(I)rrinoid adenosyltransferase MMAB) (Methylmalonic aciduria type B protein)** | Q96EY8 | MMAB | 1.20 | <0.001 | 0.031 | NO |
| **Frizzled-2 (Fz-2) (hFz2) (FzE2)** | Q14332 | FZD2 | 1.19 | <0.001 | 0.014 | NO |
| **CCN family member 2 (Cellular communication network factor 2) (Connective tissue growth factor)** | Q5M8T4 | CTGF | 1.19 | 0.002 | 0.046 | NO |
| **Zinc transporter 7 (ZnT-7) (Solute carrier family 30 member 7) (Znt-like transporter 2)** | Q8NEW0 | SLC30A7 | 1.15 | <0.001 | 0.023 | NO |
| **OX-2 membrane glycoprotein (CD antigen CD200)** | P41217 | CD200 | 1.14 | <0.001 | 0.006 | NO |
| **Bifunctional methylenetetrahydrofolate dehydrogenase/cyclohydrolase, mitochondrial [Includes: NAD-dependent methylenetetrahydrofolate dehydrogenase (EC 1.5.1.15); Methenyltetrahydrofolate cyclohydrolase (EC 3.5.4.9)]** | P13995 | MTHFD2 | 1.13 | <0.001 | 0.008 | NO |
| **Metalloproteinase inhibitor 1 (Erythroid-potentiating activity) (EPA) (Fibroblast collagenase inhibitor) (Collagenase inhibitor) (Tissue inhibitor of metalloproteinases 1) (TIMP-1)** | P01033 | TIMP1 | 1.12 | <0.001 | 0.030 | NO |
| **Alpha-2-macroglobulin (Alpha-2-M) (C3 and PZP-like alpha-2-macroglobulin domain-containing protein 5)** | P01023 | A2M | 1.07 | 0.001 | 0.043 | NO |
| **glutaminase (EC 3.5.1.2)** | B3KM58 | NA | 1.01 | <0.001 | 0.030 | NO |
| **Procollagen-lysine,2-oxoglutarate 5-dioxygenase 2 (EC 1.14.11.4) (Lysyl hydroxylase 2) (LH2)** | O00469 | PLOD2 | 1.01 | 0.001 | 0.040 | NO |
| **Monocarboxylate transporter 2 (MCT 2) (Solute carrier family 16 member 7)** | O60669 | SLC16A7 | 1.01 | 0.001 | 0.036 | YES |
| **Frizzled-1 (Fz-1) (hFz1) (FzE1)** | Q9UP38 | FZD1 | 1.00 | <0.001 | 0.025 | NO |
| **Complement factor H (H factor 1)** | P08603 | CFH | 1.00 | <0.001 | 0.017 | NO |
|  |  |  |  |  |  |  |

**Supplementary Information 7:** Total proteins which significantly (p≤0.05) at least halved and passed FDR (q≤0.05) in the failed IOL group under the contracted-phase during spontaneous contractions condition, ordered by log-fold change.

|  |  |  |  |  |  |  |
| --- | --- | --- | --- | --- | --- | --- |
| **Protein name** | **Accession** | **Gene name** | **Log Fold-change** | **p-value** | **q-value** | **Phosphorylation identified** |
| **Glutathione S-transferase Mu 1 (EC 2.5.1.18) (GST HB subunit 4) (GST class-mu 1) (GSTM1-1) (GSTM1a-1a) (GSTM1b-1b) (GTH4)** | P09488 | GSTM1 | -7.39 | <0.001 | <0.001 | NO |
| **MHC class I antigen (MHC class I protein)** | K7WT83 | HLA-A | -6.65 | <0.001 | 0.014 | NO |
| **Angiotensin-converting enzyme 2 (EC 3.4.17.23) (Angiotensin-converting enzyme homolog) (ACEH) (Angiotensin-converting enzyme-related carboxypeptidase) (ACE-related carboxypeptidase) (EC 3.4.17.-) (Metalloprotease MPROT15) [Cleaved into: Processed angiotensin-converting enzyme 2]** | Q9BYF1 | ACE2 | -5.94 | <0.001 | 0.003 | NO |
| **MHC class I antigen** | A0A1X9I3T8 | HLA-A | -5.89 | <0.001 | <0.001 | NO |
| **cDNA FLJ39696 fis, clone SMINT2011033, highly similar to Sorting and assembly machinery component 50 homolog** | B3KUE6 | NA | -5.69 | <0.001 | 0.020 | NO |
| **MHC class I antigen** | A0A1X9I444 | HLA-B | -4.77 | <0.001 | <0.001 | NO |
| **Endoplasmic reticulum DnaJ/PDI fusion protein 3** | Q6YPB0 | NA | -4.48 | <0.001 | 0.001 | NO |
| **RRBP1 protein** | A1A5C4 | RRBP1 | -3.60 | <0.001 | <0.001 | NO |
| **MHC class I antigen** | Q2LE77 | HLA-Cw | -3.47 | <0.001 | 0.003 | NO |
| **Elongator complex protein 5 (Dermal papilla-derived protein 6) (S-phase 2 protein)** | Q8TE02 | ELP5 | -3.23 | <0.001 | 0.013 | NO |
| **Transforming growth factor beta receptor type 3 (TGF-beta receptor type 3) (TGFR-3) (Betaglycan) (Transforming growth factor beta receptor III) (TGF-beta receptor type III)** | Q03167 | TGFBR3 | -3.04 | <0.001 | 0.002 | NO |
| **Aminopeptidase N (EC 3.4.11.2)** | B4DPH5 | NA | -2.97 | <0.001 | 0.024 | NO |
| **MHC class I antigen** | A0A0S4T3R3 | HLA-C | -2.83 | <0.001 | 0.024 | YES |
| **Ig-like domain-containing protein** | B7ZW57 | NA | -2.78 | <0.001 | 0.014 | NO |
| **Endoplasmic reticulum aminopeptidase 2 (EC 3.4.11.-) (Leukocyte-derived arginine aminopeptidase) (L-RAP)** | Q6P179 | ERAP2 | -2.74 | <0.001 | <0.001 | NO |
| **Glutathione S-transferase theta-2B (EC 2.5.1.18) (Glutathione S-transferase theta-2) (GST class-theta-2)** | P0CG30 | GSTT2B | -2.68 | <0.001 | 0.002 | NO |
| **Cytochrome b** | Q6VHB3 | CYTB | -2.57 | <0.001 | 0.024 | NO |
| **Glutathione S-transferase Mu 4 (EC 2.5.1.18) (GST class-mu 4) (GST-Mu2) (GSTM4-4) (Leukotriene C4 synthase GSTM4) (EC 4.4.1.20)** | Q03013 | GSTM4 | -2.54 | <0.001 | 0.004 | NO |
| **Leukocyte immunoglobulin-like receptor subfamily B member 5 (CD85 antigen-like family member C) (Leukocyte immunoglobulin-like receptor 8) (LIR-8) (CD antigen CD85c)** | O75023 | LILRB5 | -2.53 | <0.001 | <0.001 | NO |
| **Tubulin polymerization-promoting protein family member 3 (TPPP/p20)** | Q9BW30 | TPPP3 | -2.44 | <0.001 | 0.006 | NO |
| **Ephrin-B1 (EFL-3) (ELK ligand) (ELK-L) (EPH-related receptor tyrosine kinase ligand 2) (LERK-2) [Cleaved into: Ephrin-B1 C-terminal fragment (Ephrin-B1 CTF); Ephrin-B1 intracellular domain (Ephrin-B1 ICD)]** | P98172 | EFNB1 | -2.34 | <0.001 | <0.001 | NO |
| **Neprilysin (EC 3.4.24.11) (Atriopeptidase) (Common acute lymphocytic leukemia antigen) (CALLA) (Enkephalinase) (Neutral endopeptidase 24.11) (NEP) (Neutral endopeptidase) (Skin fibroblast elastase) (SFE) (CD antigen CD10)** | P08473 | MME | -2.33 | <0.001 | <0.001 | NO |
| **Proteasome assembly chaperone 2** | Q9P1R6 | NA | -2.27 | <0.001 | 0.011 | NO |
| **NA** | P01889 | NA | -2.08 | <0.001 | 0.015 | NO |
| **Neurofilament medium polypeptide (160 kDa neurofilament protein) (Neurofilament 3) (Neurofilament triplet M protein)** | A5YM63 | NEFM | -2.03 | <0.001 | 0.009 | YES |
| **MHC class I antigen** | A0A024F8H3 | HLA-B | -2.00 | <0.001 | <0.001 | NO |
| **Epiplakin (450 kDa epidermal antigen)** | P58107 | EPPK1 | -1.95 | 0.002 | 0.045 | YES |
| **Platelet glycoprotein Ib beta chain (GP-Ib beta) (GPIb-beta) (GPIbB) (Antigen CD42b-beta) (CD antigen CD42c)** | P13224 | GP1BB | -1.95 | <0.001 | 0.031 | NO |
| **cDNA FLJ55458, highly similar to Programmed cell death 6-interacting protein** | B4DHD2 | NA | -1.90 | 0.002 | 0.045 | NO |
| **NAD(P)H dehydrogenase [quinone] 1 (EC 1.6.5.2) (Azoreductase) (DT-diaphorase) (DTD) (Menadione reductase) (NAD(P)H:quinone oxidoreductase 1) (Phylloquinone reductase) (Quinone reductase 1) (QR1)** | P15559 | NQO1 | -1.60 | <0.001 | 0.017 | NO |
| **Glycogen phosphorylase, brain form (EC 2.4.1.1)** | P11216 | PYGB | -1.57 | <0.001 | 0.018 | NO |
| **Alpha-N-acetylglucosaminidase (EC 3.2.1.50) (N-acetyl-alpha-glucosaminidase) (NAG) [Cleaved into: Alpha-N-acetylglucosaminidase 82 kDa form; Alpha-N-acetylglucosaminidase 77 kDa form]** | P54802 | NAGLU | -1.55 | <0.001 | 0.001 | NO |
| **Peptidyl-prolyl cis-trans isomerase-like 3 (PPIase) (EC 5.2.1.8) (Cyclophilin J) (CyPJ) (Cyclophilin-like protein PPIL3) (Rotamase PPIL3)** | Q9H2H8 | PPIL3 | -1.53 | <0.001 | 0.004 | NO |
| **Carbonic anhydrase 12 (EC 4.2.1.1) (Carbonate dehydratase XII) (Carbonic anhydrase XII) (CA-XII) (Tumor antigen HOM-RCC-3.1.3)** | O43570 | CA12 | -1.53 | <0.001 | 0.021 | NO |
| **Aminopeptidase B (AP-B) (EC 3.4.11.6) (Arginine aminopeptidase) (Arginyl aminopeptidase)** | Q9H4A4 | RNPEP | -1.48 | <0.001 | <0.001 | NO |
| **cDNA FLJ55560, highly similar to Retinal dehydrogenase 2** | B4DH89 | NA | -1.46 | <0.001 | 0.007 | NO |
| **Katanin p80 WD40 repeat-containing subunit B1 (Katanin p80 subunit B1) (p80 katanin)** | Q9BVA0 | KATNB1 | -1.43 | 0.002 | 0.046 | NO |
| **Protein S100-A4 (Calvasculin) (Metastasin) (Placental calcium-binding protein) (Protein Mts1) (S100 calcium-binding protein A4)** | P26447 | S100A4 | -1.43 | <0.001 | 0.006 | NO |
| **MHC Class I antigen** | G9I2K8 | HLA-B | -1.42 | 0.001 | 0.037 | NO |
| **NA** | P58107 | NA | -1.42 | <0.001 | 0.003 | NO |
| **Leukocyte elastase inhibitor (LEI) (Monocyte/neutrophil elastase inhibitor) (EI) (M/NEI) (Peptidase inhibitor 2) (PI-2) (Serpin B1)** | P30740 | SERPINB1 | -1.42 | <0.001 | 0.021 | NO |
| **Calcitonin gene-related peptide type 1 receptor (CGRP type 1 receptor) (Calcitonin receptor-like receptor) (CRLR)** | Q16602 | CALCRL | -1.41 | 0.001 | 0.043 | NO |
| **Tubulin polymerization-promoting protein (TPPP) (EC 3.6.5.-) (25 kDa brain-specific protein) (TPPP/p25) (p24) (p25-alpha)** | O94811 | TPPP | -1.37 | <0.001 | 0.014 | NO |
| **Methanethiol oxidase (MTO) (EC 1.8.3.4) (56 kDa selenium-binding protein) (SBP56) (SP56) (Selenium-binding protein 1)** | Q13228 | SELENBP1 | -1.37 | <0.001 | 0.019 | NO |
| **Solute carrier organic anion transporter family member 2A1 (SLCO2A1) (OATP2A1) (PHOAR2) (Prostaglandin transporter) (PGT) (Solute carrier family 21 member 2) (SLC21A2)** | Q92959 | SLCO2A1 | -1.35 | <0.001 | 0.002 | NO |
| **Angiotensin-converting enzyme (ACE) (EC 3.4.15.1) (Dipeptidyl carboxypeptidase I) (Kininase II) (CD antigen CD143) [Cleaved into: Angiotensin-converting enzyme, soluble form]** | P12821 | ACE | -1.33 | <0.001 | 0.013 | NO |
| **Oxidoreductase HTATIP2 (EC 1.1.1.-) (30 kDa HIV-1 TAT-interacting protein) (HIV-1 TAT-interactive protein 2)** | Q9BUP3 | HTATIP2 | -1.31 | <0.001 | 0.002 | NO |
| **Ribosyldihydronicotinamide dehydrogenase [quinone] (EC 1.10.5.1) (NRH dehydrogenase [quinone] 2) (NRH:quinone oxidoreductase 2) (Quinone reductase 2) (QR2)** | P16083 | NQO2 | -1.29 | <0.001 | 0.030 | NO |
| **Insulin-like growth factor-binding protein 5 (IBP-5) (IGF-binding protein 5) (IGFBP-5)** | P24593 | IGFBP5 | -1.28 | <0.001 | 0.027 | YES |
| **Mitochondrial enolase superfamily member 1 (EC 4.2.1.68) (Antisense RNA to thymidylate synthase) (rTS) (L-fuconate dehydratase)** | Q7L5Y1 | ENOSF1 | -1.23 | <0.001 | 0.011 | NO |
| **Erythrocyte membrane protein band 4.1 like 3 (cDNA FLJ77757)** | A8K968 | EPB41L3 | -1.23 | 0.001 | 0.044 | NO |
| **Endosialin (Tumor endothelial marker 1) (CD antigen CD248)** | Q9HCU0 | CD248 | -1.22 | <0.001 | 0.003 | YES |
| **Constitutive coactivator of peroxisome proliferator-activated receptor gamma (Constitutive coactivator of PPAR-gamma) (Constitutive coactivator of PPARG) (PPARG constitutive coactivator 1) (PGCC1) (Protein FAM120B)** | Q96EK7 | FAM120B | -1.22 | <0.001 | 0.024 | NO |
| **Multidrug and toxin extrusion protein 1 (MATE-1) (hMATE-1) (Solute carrier family 47 member 1)** | Q96FL8 | SLC47A1 | -1.21 | <0.001 | 0.022 | NO |
| **A-kinase anchor protein 12 (AKAP-12) (A-kinase anchor protein 250 kDa) (AKAP 250) (Gravin) (Myasthenia gravis autoantigen)** | Q02952 | AKAP12 | -1.18 | <0.001 | 0.003 | YES |
| **Phosphatidylcholine transfer protein (PC-TP) (START domain-containing protein 2) (StARD2) (StAR-related lipid transfer protein 2)** | Q9UKL6 | PCTP | -1.18 | 0.002 | 0.047 | NO |
| **Protein-glutamine gamma-glutamyltransferase 2 (EC 2.3.2.13) (Erythrocyte transglutaminase) (Heart G alpha(h)) (hhG alpha(h)) (Isopeptidase TGM2) (EC 3.4.-.-) (Protein G alpha(h)) (G(h)) (Protein-glutamine deamidase TGM2) (EC 3.5.1.44) (Protein-glutamine dopaminyltransferase TGM2) (EC 2.3.1.-) (Protein-glutamine histaminyltransferase TGM2) (EC 2.3.1.-) (Protein-glutamine noradrenalinyltransferase TGM2) (EC 2.3.1.-) (Protein-glutamine serotonyltransferase TGM2) (EC 2.3.1.-) (Tissue transglutaminase) (tTG) (tTgase) (Transglutaminase C) (TG(C)) (TGC) (TGase C) (Transglutaminase H) (TGase H) (Transglutaminase II) (TGase II) (Transglutaminase-2) (TG2) (TGase-2) (hTG2)** | P21980 | TGM2 | -1.15 | <0.001 | 0.030 | NO |
| **Inactive phospholipase C-like protein 1 (PLC-L1) (Phospholipase C-deleted in lung carcinoma) (Phospholipase C-related but catalytically inactive protein) (PRIP)** | Q15111 | PLCL1 | -1.15 | <0.001 | 0.019 | YES |
| **NA** | Q6DN90 | NA | -1.15 | 0.002 | 0.045 | NO |
| **Lymphatic vessel endothelial hyaluronic acid receptor 1 (LYVE-1) (Cell surface retention sequence-binding protein 1) (CRSBP-1) (Extracellular link domain-containing protein 1) (Hyaluronic acid receptor)** | Q9Y5Y7 | LYVE1 | -1.13 | <0.001 | 0.029 | NO |
| **Inactive C-alpha-formylglycine-generating enzyme 2 (Paralog of formylglycine-generating enzyme) (pFGE) (Sulfatase-modifying factor 2)** | Q8NBJ7 | SUMF2 | -1.12 | <0.001 | 0.025 | NO |
| **Tumor necrosis factor alpha-induced protein 2 (TNF alpha-induced protein 2) (Primary response gene B94 protein)** | Q03169 | TNFAIP2 | -1.12 | <0.001 | 0.029 | YES |
| **Galactose mutarotase (EC 5.1.3.3) (Aldose 1-epimerase)** | Q96C23 | GALM | -1.10 | <0.001 | 0.017 | NO |
| **Ribose-phosphate pyrophosphokinase 2 (EC 2.7.6.1) (PPRibP) (Phosphoribosyl pyrophosphate synthase II) (PRS-II)** | P11908 | PRPS2 | -1.10 | <0.001 | 0.014 | NO |
| **PDZ and LIM domain protein 1 (C-terminal LIM domain protein 1) (Elfin) (LIM domain protein CLP-36)** | O00151 | PDLIM1 | -1.07 | 0.001 | 0.040 | NO |
| **Activating signal cointegrator 1 complex subunit 2 (ASC-1 complex subunit p100) (Trip4 complex subunit p100)** | Q9H1I8 | ASCC2 | -1.05 | <0.001 | 0.020 | NO |
| **Acid ceramidase (EC 3.5.1.23)** | A8K0B6 | NA | -1.05 | <0.001 | 0.019 | NO |
| **NA** | P13747 | NA | -1.05 | <0.001 | 0.018 | NO |
| **Thioredoxin-like protein 4A (DIM1 protein homolog) (Spliceosomal U5 snRNP-specific 15 kDa protein) (Thioredoxin-like U5 snRNP protein U5-15kD)** | P83876 | TXNL4A | -1.04 | <0.001 | 0.030 | NO |
| **Actin-binding LIM protein 3 (abLIM-3) (Actin-binding LIM protein family member 3)** | O94929 | ABLIM3 | -1.04 | <0.001 | 0.009 | YES |
| **SAM and SH3 domain-containing protein 1 (Proline-glutamate repeat-containing protein)** | O94885 | SASH1 | -1.03 | <0.001 | 0.014 | YES |
| **IgG receptor FcRn large subunit p51 (FcRn) (IgG Fc fragment receptor transporter alpha chain) (Neonatal Fc receptor)** | P55899 | FCGRT | -1.03 | 0.002 | 0.046 | NO |
| **Putative monooxygenase p33MONOX (EC 1.-.-.-) (Brain-derived rescue factor p60MONOX) (Flavin monooxygenase motif-containing protein of 33 kDa)** | Q96A73 | KIAA1191 | -1.02 | <0.001 | 0.024 | NO |
|  |  |  |  |  |  |  |

**Supplementary Information 8:** Total proteins which significantly (p≤0.05) at least doubled and passed FDR (q≤0.05) in the failed IOL group under the relaxed-phase during oxytocin-induced contractions condition, ordered by log-fold change.

|  |  |  |  |  |  |  |
| --- | --- | --- | --- | --- | --- | --- |
| **Protein name** | **Gene name** | **Accession** | **Log Fold-change** | **p-value** | **q-value** | **Phosphorylation identified** |
| **Choline transporter-like protein** | SLC44A2 | A0A088QCU6 | 5.56 | <0.001 | 0.006 | NO |
| **Desmuslin, isoform CRA_a (Synemin)** | SYNM | A0A075B7B1 | 5.05 | <0.001 | 0.004 | NO |
| **Cartilage oligomeric matrix protein (COMP) (Thrombospondin-5) (TSP5)** | COMP | P49747 | 4.79 | <0.001 | <0.001 | NO |
| **maleylacetoacetate isomerase (EC 5.2.1.2)** | GSTZ1 | G3V4T6 | 4.69 | <0.001 | <0.001 | NO |
| **Thrombospondin-4** | THBS4 | P35443 | 4.52 | <0.001 | <0.001 | NO |
| **Zinc finger, UBR1 type 1, isoform CRA_c** | ZUBR1 | A0A024RAC9 | 3.50 | <0.001 | 0.002 | NO |
| **Matrix Gla protein (MGP) (Cell growth-inhibiting gene 36 protein)** | MGP | P08493 | 3.33 | <0.001 | <0.001 | NO |
| **Biglycan** | BGN | C9JKG1 | 2.82 | <0.001 | 0.002 | NO |
| **N90-VRC38.08 heavy chain variable region** | NA | A0A1W6IYI5 | 2.77 | <0.001 | <0.001 | NO |
| **Thrombospondin 1, isoform CRA_a** | THBS1 | A0A024R9Q1 | 2.73 | <0.001 | <0.001 | NO |
| **Serine/threonine-protein kinase SMG1 (SMG-1) (hSMG-1) (EC 2.7.11.1) (Lambda/iota protein kinase C-interacting protein) (Lambda-interacting protein) (Nonsense mediated mRNA decay-associated PI3K-related kinase SMG1)** | SMG1 | Q96Q15 | 2.72 | 0.004 | 0.044 | NO |
| **Keratin, type II cytoskeletal 5 (Cytokeratin-5) (CK-5) (Keratin-5) (K5) (Type-II keratin Kb5)** | KRT5 | Q5XQN5 | 2.67 | <0.001 | 0.007 | NO |
| **Serpin peptidase inhibitor, clade E (Nexin, plasminogen activator inhibitor type 1), member 1, isoform CRA_b** | SERPINE1 | A0A024QYT5 | 2.51 | <0.001 | <0.001 | NO |
| **TNC variant protein** | TNC variant protein | Q4LE33 | 2.49 | <0.001 | <0.001 | YES |
| **Immunoglobulin heavy constant gamma 4 (Ig gamma-4 chain C region)** | IGHG4 | P01861 | 2.44 | <0.001 | 0.011 | NO |
| **Midkine (MK) (Amphiregulin-associated protein) (ARAP) (Midgestation and kidney protein) (Neurite outgrowth-promoting factor 2) (Neurite outgrowth-promoting protein)** | MDK | P21741 | 2.36 | 0.002 | 0.031 | NO |
| **Thrombospondin-1 (Glycoprotein G)** | THBS1 | P07996 | 2.34 | 0.002 | 0.028 | NO |
| **Biglycan (Bone/cartilage proteoglycan I) (PG-S1)** | BGN | P21810 | 2.33 | <0.001 | <0.001 | NO |
| **Keratin, type I cytoskeletal 10 (Cytokeratin-10) (CK-10) (Keratin-10) (K10)** | KRT10 | P13645 | 2.30 | <0.001 | 0.017 | NO |
| **Keratin, type II cytoskeletal 1 (Cytokeratin-1) (Keratin-1) (Type-II keratin Kb1)** | KRT1 | H6VRG2 | 2.29 | <0.001 | 0.007 | NO |
| **Transforming growth factor-beta-induced protein ig-h3 (Beta ig-h3) (Kerato-epithelin) (RGD-containing collagen-associated protein) (RGD-CAP)** | TGFBI | Q15582 | 2.29 | <0.001 | <0.001 | NO |
| **Keratin, type I cytoskeletal 9 (Cytokeratin-9) (CK-9) (Keratin-9) (K9)** | KRT9 | P35527 | 2.27 | <0.001 | 0.006 | NO |
| **Annexin A3 (35-alpha calcimedin) (Annexin III) (Annexin-3) (Inositol 1,2-cyclic phosphate 2-phosphohydrolase) (Lipocortin III) (Placental anticoagulant protein III) (PAP-III)** | ANXA3 | P12429 | 2.25 | <0.001 | <0.001 | NO |
| **Matrilin-2** | MATN2 | O00339 | 2.25 | <0.001 | <0.001 | NO |
| **Carbonic anhydrase III, muscle specific, isoform CRA_a** | CA3 | A0A024R825 | 2.12 | <0.001 | 0.015 | NO |
| **PG-M** | DKFZp686K06110 | Q6MZK8 | 2.09 | <0.001 | 0.009 | YES |
| **Dermcidin (EC 3.4.-.-) (Preproteolysin) [Cleaved into: Survival-promoting peptide; DCD-1]** | DCD | P81605 | 2.07 | 0.001 | 0.021 | NO |
| **Apolipoprotein(a) (Apo(a)) (Lp(a)) (EC 3.4.21.-)** | LPA | P08519 | 2.03 | <0.001 | 0.007 | NO |
| **Keratin, type II cytoskeletal 2 epidermal (Cytokeratin-2e) (CK-2e) (Epithelial keratin-2e) (Keratin-2 epidermis) (Keratin-2e) (K2e) (Type-II keratin Kb2)** | KRT2 | P35908 | 2.03 | 0.003 | 0.043 | NO |
| **Mimecan (Osteoglycin) (Osteoinductive factor) (OIF)** | OGN | P20774 | 2.01 | <0.001 | 0.003 | NO |
| **SAA2-SAA4 readthrough** | SAA2-SAA4 | A0A096LPE2 | 2.00 | <0.001 | <0.001 | NO |
| **Protein AAR2 homolog (AAR2 splicing factor homolog)** | AAR2 | A2A2Q9 | 1.98 | <0.001 | 0.010 | NO |
| **Asporin (Periodontal ligament-associated protein 1) (PLAP-1)** | ASPN | Q9BXN1 | 1.94 | <0.001 | 0.002 | NO |
| **Vitamin D binding protein** | Gc | A0A1B1CYC5 | 1.93 | <0.001 | <0.001 | NO |
| **Cytoplasmic dynein 2 heavy chain 1 (Cytoplasmic dynein 2 heavy chain) (Dynein cytoplasmic heavy chain 2) (Dynein heavy chain 11) (hDHC11) (Dynein heavy chain isotype 1B)** | DYNC2H1 | Q8NCM8 | 1.93 | <0.001 | 0.011 | NO |
| **D-3-phosphoglycerate dehydrogenase (3-PGDH) (EC 1.1.1.95) (2-oxoglutarate reductase) (EC 1.1.1.399) (Malate dehydrogenase) (EC 1.1.1.37)** | PHGDH | O43175 | 1.90 | <0.001 | 0.007 | NO |
| **Cadherin 2, type 1, N-cadherin (Neuronal), isoform CRA_b** | CDH2 | A0A024RC42 | 1.87 | <0.001 | <0.001 | NO |
| **Zinc finger FYVE domain-containing protein 26 (FYVE domain-containing centrosomal protein) (FYVE-CENT) (Spastizin)** | ZFYVE26 | Q68DK2 | 1.85 | <0.001 | 0.004 | NO |
| **Glycerol-3-phosphate acyltransferase 4 (EC 2.3.1.15) (1-acylglycerol-3-phosphate O-acyltransferase 6) (1-AGP acyltransferase 6) (1-AGPAT 6) (Acyl-CoA:glycerol-3-phosphate acyltransferase 4) (Lysophosphatidic acid acyltransferase zeta) (LPAAT-zeta) (Testis spermatogenesis apoptosis-related protein 7) (TSARG7)** | GPAT4 | Q86UL3 | 1.85 | 0.003 | 0.039 | NO |
| **Collagen alpha-1(XII) chain** | COL12A1 | Q99715 | 1.84 | <0.001 | 0.006 | NO |
| **Haptoglobin (Zonulin) [Cleaved into: Haptoglobin alpha chain; Haptoglobin beta chain]** | HP | P00738 | 1.84 | <0.001 | 0.003 | NO |
| **IGH@ protein** | IGH@ | Q6P089 | 1.84 | <0.001 | 0.017 | NO |
| **Periostin, osteoblast specific factor, isoform CRA_a** | POSTN | A0A024RDT5 | 1.83 | 0.001 | 0.022 | NO |
| **Methylthioribulose-1-phosphate dehydratase (MTRu-1-P dehydratase) (EC 4.2.1.109) (APAF1-interacting protein) (hAPIP)** | APIP | Q96GX9 | 1.81 | <0.001 | 0.017 | NO |
| **Inter-alpha-trypsin inhibitor heavy chain H1 (ITI heavy chain H1) (ITI-HC1) (Inter-alpha-inhibitor heavy chain 1) (Inter-alpha-trypsin inhibitor complex component III) (Serum-derived hyaluronan-associated protein) (SHAP)** | ITIH1 | P19827 | 1.79 | <0.001 | <0.001 | NO |
| **Tenascin-X** | TNXB | A0A087WWA5 | 1.77 | <0.001 | 0.007 | NO |
| **Tenascin-X (TN-X) (Hexabrachion-like protein)** | TNXB | P22105 | 1.76 | <0.001 | <0.001 | YES |
| **Lysyl oxidase homolog 2 (EC 1.4.3.13) (Lysyl oxidase-like protein 2) (Lysyl oxidase-related protein 2) (Lysyl oxidase-related protein WS9-14)** | LOXL2 | Q9Y4K0 | 1.75 | <0.001 | <0.001 | NO |
| **Prostaglandin G/H synthase 1 (EC 1.14.99.1) (Cyclooxygenase-1) (COX-1) (Prostaglandin H2 synthase 1) (PGH synthase 1) (PGHS-1) (PHS 1) (Prostaglandin-endoperoxide synthase 1)** | PTGS1 | P23219 | 1.71 | <0.001 | <0.001 | NO |
| **Latent-transforming growth factor beta-binding protein 2 (LTBP-2)** | LTBP2 | Q14767 | 1.70 | <0.001 | <0.001 | NO |
| **Myosin-reactive immunoglobulin heavy chain variable region** | NA | Q9UL90 | 1.69 | <0.001 | 0.011 | NO |
| **Filamin A** | FLNA | F8WE98 | 1.69 | 0.002 | 0.029 | NO |
| **Collagen alpha-1(XV) chain [Cleaved into: Restin (Endostatin-XV) (Related to endostatin) (Restin-I); Restin-2 (Restin-II); Restin-3 (Restin-III); Restin-4 (Restin-IV)]** | COL15A1 | P39059 | 1.68 | <0.001 | 0.004 | NO |
| **SPARC-related modular calcium-binding protein 1 (Secreted modular calcium-binding protein 1) (SMOC-1)** | SMOC1 | Q9H4F8 | 1.63 | 0.001 | 0.025 | NO |
| **Ig-like domain-containing protein** | DKFZp686K03196 | Q6N095 | 1.61 | 0.002 | 0.035 | NO |
| **Prostacyclin synthase (EC 5.3.99.4) (Hydroperoxy icosatetraenoate dehydratase) (EC 4.2.1.152) (Prostaglandin I2 synthase)** | PTGIS | Q16647 | 1.59 | <0.001 | <0.001 | NO |
| **Septin-6** | SEPTIN6 | Q14141 | 1.58 | <0.001 | <0.001 | NO |
| **Diphosphoinositol polyphosphate phosphohydrolase 3-alpha (DIPP-3-alpha) (DIPP3-alpha) (hDIPP3alpha) (EC 3.6.1.52) (Diadenosine 5',5'''-P1,P6-hexaphosphate hydrolase 3-alpha) (Diadenosine hexaphosphate hydrolase (AMP-forming)) (EC 3.6.1.60) (Nucleoside diphosphate-linked moiety X motif 10) (Nudix motif 10) (hAps2)** | NUDT10 | Q8NFP7 | 1.58 | <0.001 | 0.004 | NO |
| **NA** | NA | Q15063 | 1.57 | 0.003 | 0.038 | NO |
| **Polypeptide N-acetylgalactosaminyltransferase 18 (EC 2.4.1.41) (Polypeptide GalNAc transferase 18) (GalNAc-T18) (Polypeptide GalNAc transferase-like protein 4) (GalNAc-T-like protein 4) (pp-GaNTase-like protein 4) (Polypeptide N-acetylgalactosaminyltransferase-like protein 4) (Protein-UDP acetylgalactosaminyltransferase-like protein 4) (UDP-GalNAc:polypeptide N-acetylgalactosaminyltransferase-like protein 4)** | GALNT18 | Q6P9A2 | 1.57 | <0.001 | 0.006 | NO |
| **Extracellular sulfatase Sulf-1 (hSulf-1) (Arylsulfatase) (EC 3.1.6.1) (N-acetylglucosamine-6-sulfatase) (EC 3.1.6.14) [Cleaved into: Extracellular sulfatase Sulf-2 secreted form]** | SULF1 | Q8IWU6 | 1.56 | 0.003 | 0.042 | NO |
| **Metalloproteinase inhibitor 1 (Erythroid-potentiating activity) (EPA) (Fibroblast collagenase inhibitor) (Collagenase inhibitor) (Tissue inhibitor of metalloproteinases 1) (TIMP-1)** | TIMP1 | P01033 | 1.54 | <0.001 | 0.002 | NO |
| **Apolipoprotein C-III (Apo-CIII) (ApoC-III) (Apolipoprotein C3)** | APOC3 | P02656 | 1.54 | <0.001 | 0.006 | NO |
| **CCN family member 1 (Cellular communication network factor 1) (Cysteine-rich angiogenic inducer 61) (Insulin-like growth factor-binding protein 10) (IBP-10) (IGF-binding protein 10) (IGFBP-10) (Protein CYR61) (Protein GIG1)** | CCN1 | O00622 | 1.53 | <0.001 | 0.004 | NO |
| **Cysteine-rich motor neuron 1 protein (CRIM-1) (Cysteine-rich repeat-containing protein S52) [Cleaved into: Processed cysteine-rich motor neuron 1 protein]** | CRIM1 | Q9NZV1 | 1.48 | 0.004 | 0.044 | NO |
| **cDNA FLJ51896, highly similar to Glia-derived nexin** | NA | B4DMR3 | 1.46 | <0.001 | 0.008 | NO |
| **Transmembrane protein 106B** | TMEM106B | Q9NUM4 | 1.45 | 0.004 | 0.047 | NO |
| **Tuftelin** | TUFT1 | Q9NNX1 | 1.42 | <0.001 | 0.008 | NO |
| **Lumican (Corneal keratan sulfate proteoglycan 37B core protein) (Keratan sulfate proteoglycan) (KSPG)** | LUM | Q05443 | 1.41 | 0.002 | 0.029 | NO |
| **Corrinoid adenosyltransferase MMAB (EC 2.5.1.-) (ATP:co(I)rrinoid adenosyltransferase MMAB) (Methylmalonic aciduria type B protein)** | MMAB | Q96EY8 | 1.40 | <0.001 | 0.006 | NO |
| **Uncharacterized protein DKFZp686O1553** | DKFZp686O1553 | Q5HYM1 | 1.39 | <0.001 | <0.001 | NO |
| **MHC class II antigen** | HLA-DPB1 | A0A1Y6MRR4 | 1.38 | 0.002 | 0.026 | NO |
| **DNA repair protein XRCC1 (X-ray repair cross-complementing protein 1)** | XRCC1 | P18887 | 1.38 | 0.004 | 0.048 | NO |
| **C14orf159 variant protein** | C14orf159 variant protein | Q4LE40 | 1.38 | <0.001 | <0.001 | NO |
| **Uncharacterized protein DKFZp686M0562** | DKFZp686M0562 | Q6MZL2 | 1.38 | 0.002 | 0.035 | NO |
| **Fibrillin-2 [Cleaved into: Placensin]** | FBN2 | P35556 | 1.37 | 0.002 | 0.032 | NO |
| **Ribonuclease T2 (EC 4.6.1.19) (Ribonuclease 6)** | RNASET2 | O00584 | 1.37 | <0.001 | 0.005 | NO |
| **Monocarboxylate transporter 2 (MCT 2) (Solute carrier family 16 member 7)** | SLC16A7 | O60669 | 1.36 | <0.001 | 0.003 | YES |
| **Microfibril-associated glycoprotein 4** | MFAP4 | P55083 | 1.36 | 0.003 | 0.039 | NO |
| **Bifunctional methylenetetrahydrofolate dehydrogenase/cyclohydrolase, mitochondrial [Includes: NAD-dependent methylenetetrahydrofolate dehydrogenase (EC 1.5.1.15); Methenyltetrahydrofolate cyclohydrolase (EC 3.5.4.9)]** | MTHFD2 | P13995 | 1.36 | <0.001 | <0.001 | NO |
| **CCN family member 2 (Cellular communication network factor 2) (Connective tissue growth factor)** | CTGF | Q5M8T4 | 1.34 | <0.001 | 0.011 | NO |
| **Adipocyte enhancer-binding protein 1 (AE-binding protein 1) (Aortic carboxypeptidase-like protein)** | AEBP1 | Q8IUX7 | 1.34 | <0.001 | 0.007 | NO |
| **Synaptophysin-like protein 2** | SYPL2 | Q5VXT5 | 1.33 | <0.001 | 0.006 | NO |
| **Zinc transporter 7 (ZnT-7) (Solute carrier family 30 member 7) (Znt-like transporter 2)** | SLC30A7 | Q8NEW0 | 1.33 | <0.001 | 0.004 | NO |
| **Twisted gastrulation protein homolog 1** | TWSG1 | Q9GZX9 | 1.32 | <0.001 | 0.006 | NO |
| **OX-2 membrane glycoprotein (CD antigen CD200)** | CD200 | P41217 | 1.32 | <0.001 | <0.001 | NO |
| **Inter-alpha-trypsin inhibitor heavy chain H2 (ITI heavy chain H2) (ITI-HC2) (Inter-alpha-inhibitor heavy chain 2) (Inter-alpha-trypsin inhibitor complex component II) (Serum-derived hyaluronan-associated protein) (SHAP)** | ITIH2 | P19823 | 1.32 | <0.001 | <0.001 | NO |
| **Uncharacterized protein DKFZp686K18196** | DKFZp686K18196 | Q6N092 | 1.31 | 0.004 | 0.044 | NO |
| **Sodium/potassium-transporting ATPase subunit alpha** | NA | B3KW93 | 1.30 | 0.002 | 0.026 | NO |
| **[Pyruvate dehydrogenase (acetyl-transferring)] kinase isozyme 1, mitochondrial (EC 2.7.11.2) (Pyruvate dehydrogenase kinase isoform 1) (PDH kinase 1)** | PDK1 | Q15118 | 1.30 | <0.001 | 0.012 | NO |
| **Chromosome 1 open reading frame 24, isoform CRA_a** | C1orf24 | A0A024R978 | 1.29 | <0.001 | 0.008 | NO |
| **Glypican-4 (K-glypican) [Cleaved into: Secreted glypican-4]** | GPC4 | O75487 | 1.29 | <0.001 | <0.001 | YES |
| **Fibroleukin (Fibrinogen-like protein 2) (pT49)** | FGL2 | Q14314 | 1.27 | <0.001 | 0.016 | NO |
| **Tubulointerstitial nephritis antigen-like (Glucocorticoid-inducible protein 5) (Oxidized LDL-responsive gene 2 protein) (OLRG-2) (Tubulointerstitial nephritis antigen-related protein) (TIN Ag-related protein) (TIN-Ag-RP)** | TINAGL1 | Q9GZM7 | 1.27 | 0.002 | 0.035 | NO |
| **FXYD domain containing ion transport regulator 6, isoform CRA_a** | FXYD6 | A0A024R3J8 | 1.26 | <0.001 | 0.007 | NO |
| **Protein AMBP (Protein HC) [Cleaved into: Alpha-1-microglobulin (EC 1.6.2.-) (Alpha-1 microglycoprotein) (Complex-forming glycoprotein heterogeneous in charge); Inter-alpha-trypsin inhibitor light chain (ITI-LC) (Bikunin) (EDC1) (HI-30) (Uronic-acid-rich protein); Trypstatin]** | AMBP | P02760 | 1.26 | <0.001 | 0.010 | NO |
| **Matrix metalloproteinase-19 (MMP-19) (EC 3.4.24.-) (Matrix metalloproteinase RASI) (Matrix metalloproteinase-18) (MMP-18)** | MMP19 | Q99542 | 1.24 | <0.001 | 0.006 | NO |
| **Neurofilament light polypeptide isoform 2** | NEFL | A0A0S2Z4B1 | 1.24 | <0.001 | 0.009 | NO |
| **Probable phospholipid-transporting ATPase IIA (EC 7.6.2.1) (ATPase class II type 9A)** | ATP9A | O75110 | 1.23 | <0.001 | 0.012 | NO |
| **Retinoic acid receptor responder protein 2 (Chemerin) (RAR-responsive protein TIG2) (Tazarotene-induced gene 2 protein)** | RARRES2 | Q99969 | 1.22 | <0.001 | 0.009 | NO |
| **glutaminase (EC 3.5.1.2)** | NA | B3KM58 | 1.21 | <0.001 | 0.005 | NO |
| **Nucleolar RNA helicase 2 (EC 3.6.4.13) (DEAD box protein 21) (Gu-alpha) (Nucleolar RNA helicase Gu) (Nucleolar RNA helicase II) (RH II/Gu)** | DDX21 | Q9NR30 | 1.20 | 0.001 | 0.024 | NO |
| **Chondroitin sulfate proteoglycan 2 (Versican), isoform CRA_c** | CSPG2 | A0A024RAL1 | 1.20 | <0.001 | 0.015 | YES |
| **Tetraspanin-15 (Tspan-15) (Tetraspan NET-7) (Transmembrane 4 superfamily member 15)** | TSPAN15 | O95858 | 1.20 | <0.001 | 0.004 | NO |
| **Pentraxin-related protein PTX3 (Pentaxin-related protein PTX3) (Tumor necrosis factor alpha-induced protein 5) (TNF alpha-induced protein 5) (Tumor necrosis factor-inducible gene 14 protein) (TSG-14)** | PTX3 | P26022 | 1.19 | 0.001 | 0.023 | NO |
| **Lumican (Keratan sulfate proteoglycan lumican) (KSPG lumican)** | LUM | P51884 | 1.18 | <0.001 | 0.007 | NO |
| **Liver carboxylesterase 1 (Acyl-coenzyme A:cholesterol acyltransferase) (ACAT) (Brain carboxylesterase hBr1) (Carboxylesterase 1) (CE-1) (hCE-1) (EC 3.1.1.1) (Cholesteryl ester hydrolase) (CEH) (EC 3.1.1.13) (Cocaine carboxylesterase) (Egasyn) (HMSE) (Methylumbelliferyl-acetate deacetylase 1) (EC 3.1.1.56) (Monocyte/macrophage serine esterase) (Retinyl ester hydrolase) (REH) (Serine esterase 1) (Triacylglycerol hydrolase) (TGH)** | CES1 | P23141 | 1.17 | <0.001 | 0.007 | NO |
| **Glutaminase kidney isoform, mitochondrial (GLS) (EC 3.5.1.2) (K-glutaminase) (L-glutamine amidohydrolase) [Cleaved into: Glutaminase kidney isoform, mitochondrial 68 kDa chain; Glutaminase kidney isoform, mitochondrial 65 kDa chain]** | GLS | O94925 | 1.17 | <0.001 | 0.006 | NO |
| **SH3 domain-binding glutamic acid-rich-like protein 2 (Fovea-associated SH3 domain-binding protein)** | SH3BGRL2 | Q9UJC5 | 1.17 | <0.001 | 0.003 | NO |
| **Microsomal glutathione S-transferase 2 (Microsomal GST-2) (EC 2.5.1.18) (Glutathione peroxidase MGST2) (EC 1.11.1.-) (Leukotriene C4 synthase MGST2) (EC 4.4.1.20) (Microsomal glutathione S-transferase II) (Microsomal GST-II)** | MGST2 | Q99735 | 1.16 | <0.001 | 0.012 | NO |
| **Latent-transforming growth factor beta-binding protein 4 (LTBP-4)** | LTBP4 | Q8N2S1 | 1.14 | <0.001 | 0.015 | NO |
| **Prolargin (Proline-arginine-rich end leucine-rich repeat protein)** | PRELP | P51888 | 1.14 | <0.001 | 0.005 | NO |
| **Metallothionein-1E (MT-1E) (Metallothionein-IE) (MT-IE)** | MT1E | P04732 | 1.13 | <0.001 | 0.016 | NO |
| **Prostaglandin F2 receptor negative regulator (CD9 partner 1) (CD9P-1) (Glu-Trp-Ile EWI motif-containing protein F) (EWI-F) (Prostaglandin F2-alpha receptor regulatory protein) (Prostaglandin F2-alpha receptor-associated protein) (CD antigen CD315)** | PTGFRN | Q9P2B2 | 1.13 | <0.001 | 0.015 | NO |
| **Integrin alpha-7 [Cleaved into: Integrin alpha-7 heavy chain; Integrin alpha-7 light chain; Integrin alpha-7 70 kDa form]** | ITGA7 | Q13683 | 1.13 | <0.001 | 0.017 | NO |
| **NADH:ubiquinone oxidoreductase MLRQ subunit homolog, isoform CRA_a** | LOC56901 | A0A024RB39 | 1.10 | 0.001 | 0.025 | NO |
| **Uncharacterized protein KIAA1671** | KIAA1671 | Q9BY89 | 1.10 | 0.003 | 0.038 | YES |
| **Guanine nucleotide-binding protein G(i) subunit alpha-1 (EC 3.6.5.-) (Adenylate cyclase-inhibiting G alpha protein)** | GNAI1 | P63096 | 1.09 | <0.001 | <0.001 | NO |
| **Procollagen-lysine,2-oxoglutarate 5-dioxygenase 2 (EC 1.14.11.4) (Lysyl hydroxylase 2) (LH2)** | PLOD2 | O00469 | 1.09 | <0.001 | 0.012 | NO |
| **Laminin subunit alpha-2 (Laminin M chain) (Laminin-12 subunit alpha) (Laminin-2 subunit alpha) (Laminin-4 subunit alpha) (Merosin heavy chain)** | LAMA2 | P24043 | 1.08 | <0.001 | 0.013 | NO |
| **ADP/ATP translocase 1 (ADP,ATP carrier protein 1) (ADP,ATP carrier protein, heart/skeletal muscle isoform T1) (Adenine nucleotide translocator 1) (ANT 1) (Solute carrier family 25 member 4)** | SLC25A4 | P12235 | 1.07 | <0.001 | 0.007 | NO |
| **Alpha-catulin (Alpha-catenin-related protein) (ACRP) (Catenin alpha-like protein 1)** | CTNNAL1 | Q9UBT7 | 1.06 | 0.001 | 0.025 | NO |
| **Cytochrome b-c1 complex subunit 9 (Complex III subunit 9) (Complex III subunit X) (Cytochrome c1 non-heme 7 kDa protein) (Ubiquinol-cytochrome c reductase complex 7.2 kDa protein)** | UQCR10 | Q9UDW1 | 1.05 | <0.001 | 0.006 | NO |
| **Thrombospondin-3** | THBS3 | P49746 | 1.05 | <0.001 | 0.007 | NO |
| **Junctional adhesion molecule B (JAM-B) (Junctional adhesion molecule 2) (JAM-2) (Vascular endothelial junction-associated molecule) (VE-JAM) (CD antigen CD322)** | JAM2 | P57087 | 1.05 | <0.001 | 0.004 | NO |
| **Coiled-coil domain-containing protein 80 (Down-regulated by oncogenes protein 1) (Up-regulated in BRS-3 deficient mouse homolog)** | CCDC80 | Q76M96 | 1.05 | <0.001 | 0.015 | NO |
| **Alpha-1B-glycoprotein (Alpha-1-B glycoprotein)** | A1BG | P04217 | 1.03 | <0.001 | 0.015 | NO |
| **NA** | NA | Q8IUG5 | 1.02 | 0.003 | 0.043 | NO |
| **Acrosomal protein KIAA1210** | KIAA1210 | Q9ULL0 | 1.01 | 0.001 | 0.026 | NO |
| **Pregnancy zone protein (C3 and PZP-like alpha-2-macroglobulin domain-containing protein 6)** | PZP | P20742 | 1.01 | 0.004 | 0.045 | NO |
| **DNA helicase MCM8 (EC 3.6.4.12) (Minichromosome maintenance 8)** | MCM8 | Q9UJA3 | 1.00 | <0.001 | 0.018 | NO |
| **Insulin-like growth factor-binding protein 7 (IBP-7) (IGF-binding protein 7) (IGFBP-7) (IGFBP-rP1) (MAC25 protein) (PGI2-stimulating factor) (Prostacyclin-stimulating factor) (Tumor-derived adhesion factor) (TAF)** | IGFBP7 | Q16270 | 1.00 | 0.002 | 0.032 | NO |
|  |  |  |  |  |  |  |

**Supplementary Information 9:** Total proteins which significantly (p≤0.05) at least halved and passed FDR (q≤0.05) in the failed IOL group under the relaxed-phase during oxytocin-induced contractions condition, ordered by log-fold change.

|  |  |  |  |  |  |  |
| --- | --- | --- | --- | --- | --- | --- |
| **Protein name** | **Gene name** | **Accession** | **Log Fold-change** | **p-value** | **q-value** | **Phosphorylation identified** |
| **Glutathione S-transferase Mu 1 (EC 2.5.1.18) (GST HB subunit 4) (GST class-mu 1) (GSTM1-1) (GSTM1a-1a) (GSTM1b-1b) (GTH4)** | P09488 | GSTM1 | -6.68 | <0.001 | <0.001 | NO |
| **MHC class I antigen** | A0A1X9I3T8 | HLA-A | -6.14 | <0.001 | <0.001 | NO |
| **MHC class I antigen (MHC class I protein)** | K7WT83 | HLA-A | -5.63 | 0.002 | 0.029 | NO |
| **Granulysin (Lymphokine LAG-2) (Protein NKG5) (T-cell activation protein 519)** | P22749 | GNLY | -5.26 | <0.001 | 0.019 | NO |
| **HLA DR-beta-III** | Q30131 | NA | -4.69 | 0.004 | 0.046 | NO |
| **Endoplasmic reticulum DnaJ/PDI fusion protein 3** | Q6YPB0 | NA | -4.17 | <0.001 | 0.001 | NO |
| **MHC class I antigen** | A0A1X9I444 | HLA-B | -3.90 | <0.001 | <0.001 | NO |
| **Angiotensin-converting enzyme 2 (EC 3.4.17.23) (Angiotensin-converting enzyme homolog) (ACEH) (Angiotensin-converting enzyme-related carboxypeptidase) (ACE-related carboxypeptidase) (EC 3.4.17.-) (Metalloprotease MPROT15) [Cleaved into: Processed angiotensin-converting enzyme 2]** | Q9BYF1 | ACE2 | -3.80 | <0.001 | 0.012 | NO |
| **X-linked retinitis pigmentosa GTPase regulator** | Q92834 | RPGR | -3.52 | 0.002 | 0.030 | NO |
| **RRBP1 protein** | A1A5C4 | RRBP1 | -3.50 | <0.001 | <0.001 | NO |
| **MHC class I antigen** | A0A0S4T3R3 | HLA-C | -3.42 | <0.001 | 0.004 | YES |
| **Glutathione S-transferase theta-2B (EC 2.5.1.18) (Glutathione S-transferase theta-2) (GST class-theta-2)** | P0CG30 | GSTT2B | -2.87 | <0.001 | <0.001 | NO |
| **MHC class I antigen** | Q2LE77 | HLA-Cw | -2.78 | <0.001 | 0.004 | NO |
| **Endoplasmic reticulum aminopeptidase 2 (EC 3.4.11.-) (Leukocyte-derived arginine aminopeptidase) (L-RAP)** | Q6P179 | ERAP2 | -2.65 | <0.001 | <0.001 | NO |
| **Glutathione S-transferase Mu 4 (EC 2.5.1.18) (GST class-mu 4) (GST-Mu2) (GSTM4-4) (Leukotriene C4 synthase GSTM4) (EC 4.4.1.20)** | Q03013 | GSTM4 | -2.47 | <0.001 | 0.002 | NO |
| **AP complex subunit beta** | B2RBF6 | NA | -2.35 | <0.001 | 0.006 | NO |
| **Hemoglobin subunit delta** | E9PEW8 | HBD | -2.33 | 0.002 | 0.033 | NO |
| **cDNA, FLJ95164, highly similar to Homo sapiens SEC14-like 2 (S. cerevisiae) (SEC14L2), mRNA** | B2RAW8 | NA | -2.33 | <0.001 | <0.001 | NO |
| **MHC class I antigen** | A0A1G4HPS6 | HLA-A | -2.29 | <0.001 | 0.006 | NO |
| **Leukocyte immunoglobulin-like receptor subfamily B member 5 (CD85 antigen-like family member C) (Leukocyte immunoglobulin-like receptor 8) (LIR-8) (CD antigen CD85c)** | O75023 | LILRB5 | -2.26 | <0.001 | <0.001 | NO |
| **MHC class I antigen** | A0A024F8H3 | HLA-B | -2.19 | <0.001 | <0.001 | NO |
| **Ephrin-B1 (EFL-3) (ELK ligand) (ELK-L) (EPH-related receptor tyrosine kinase ligand 2) (LERK-2) [Cleaved into: Ephrin-B1 C-terminal fragment (Ephrin-B1 CTF); Ephrin-B1 intracellular domain (Ephrin-B1 ICD)]** | P98172 | EFNB1 | -2.11 | <0.001 | <0.001 | NO |
| **Keratin, type I cytoskeletal 19 (Cytokeratin-19) (CK-19) (Keratin-19) (K19)** | P08727 | KRT19 | -2.11 | <0.001 | 0.009 | NO |
| **Tubulin polymerization-promoting protein family member 3 (TPPP/p20)** | Q9BW30 | TPPP3 | -2.10 | <0.001 | 0.006 | NO |
| **Aminopeptidase N (EC 3.4.11.2)** | B4DPH5 | NA | -2.10 | 0.003 | 0.036 | NO |
| **Rho GTPase-activating protein 4 (Rho-GAP hematopoietic protein C1) (Rho-type GTPase-activating protein 4) (p115)** | P98171 | ARHGAP4 | -2.06 | 0.002 | 0.030 | NO |
| **Epiplakin (450 kDa epidermal antigen)** | P58107 | EPPK1 | -2.06 | <0.001 | 0.015 | YES |
| **Fatty acid-binding protein 5 (Epidermal-type fatty acid-binding protein) (E-FABP) (Fatty acid-binding protein, epidermal) (Psoriasis-associated fatty acid-binding protein homolog) (PA-FABP)** | Q01469 | FABP5 | -2.02 | <0.001 | 0.007 | NO |
| **Hemoglobin delta-beta fusion protein** | Q5XTR9 | HBD/HBB | -2.00 | 0.004 | 0.048 | NO |
| **Carbonic anhydrase 2 (EC 4.2.1.1) (Carbonate dehydratase II) (Carbonic anhydrase C) (CAC) (Carbonic anhydrase II) (CA-II) (Cyanamide hydratase CA2) (EC 4.2.1.69)** | P00918 | CA2 | -1.98 | 0.001 | 0.022 | NO |
| **Neprilysin (EC 3.4.24.11) (Atriopeptidase) (Common acute lymphocytic leukemia antigen) (CALLA) (Enkephalinase) (Neutral endopeptidase 24.11) (NEP) (Neutral endopeptidase) (Skin fibroblast elastase) (SFE) (CD antigen CD10)** | P08473 | MME | -1.96 | <0.001 | <0.001 | NO |
| **Protein LSM14 homolog B (RNA-associated protein 55B) (hRAP55B)** | Q9BX40 | LSM14B | -1.95 | <0.001 | 0.012 | NO |
| **Fatty acid-binding protein, heart (Fatty acid-binding protein 3) (Heart-type fatty acid-binding protein) (H-FABP) (Mammary-derived growth inhibitor) (MDGI) (Muscle fatty acid-binding protein) (M-FABP)** | P05413 | FABP3 | -1.93 | <0.001 | 0.008 | NO |
| **Beta-globin protein** | Q9UM85 | beta-globin | -1.93 | <0.001 | 0.020 | NO |
| **Keratin, type II cytoskeletal 8 (Cytokeratin-8) (CK-8) (Keratin-8) (K8) (Type-II keratin Kb8)** | P05787 | KRT8 | -1.90 | <0.001 | 0.010 | NO |
| **Alpha-hemoglobin-stabilizing protein (Erythroid differentiation-related factor) (Erythroid-associated factor)** | Q9NZD4 | AHSP | -1.87 | <0.001 | 0.018 | NO |
| **Proteasome assembly chaperone 2** | Q9P1R6 | NA | -1.86 | <0.001 | 0.013 | NO |
| **Platelet glycoprotein Ib beta chain (GP-Ib beta) (GPIb-beta) (GPIbB) (Antigen CD42b-beta) (CD antigen CD42c)** | P13224 | GP1BB | -1.86 | <0.001 | 0.015 | NO |
| **Glycogen phosphorylase, brain form (EC 2.4.1.1)** | P11216 | PYGB | -1.86 | <0.001 | 0.003 | NO |
| **Carbonic anhydrase 1 (EC 4.2.1.1) (Carbonate dehydratase I) (Carbonic anhydrase B) (CAB) (Carbonic anhydrase I) (CA-I) (Cyanamide hydratase CA1) (EC 4.2.1.69)** | P00915 | CA1 | -1.79 | 0.003 | 0.042 | NO |
| **Hemoglobin subunit delta (Delta-globin) (Hemoglobin delta chain)** | P02042 | HBD | -1.78 | 0.004 | 0.046 | NO |
| **Peptidyl-prolyl cis-trans isomerase-like 3 (PPIase) (EC 5.2.1.8) (Cyclophilin J) (CyPJ) (Cyclophilin-like protein PPIL3) (Rotamase PPIL3)** | Q9H2H8 | PPIL3 | -1.78 | <0.001 | <0.001 | NO |
| **Poly(rC)-binding protein 3 (Alpha-CP3) (PCBP3-overlapping transcript) (PCBP3-overlapping transcript 1)** | P57721 | PCBP3 | -1.77 | <0.001 | 0.014 | NO |
| **Hemoglobin beta subunit variant** | Q9UK54 | HBB | -1.77 | 0.004 | 0.046 | NO |
| **Ig-like domain-containing protein** | B7ZW57 | NA | -1.76 | 0.004 | 0.046 | NO |
| **Zinc finger protein 648** | Q5T619 | ZNF648 | -1.74 | 0.004 | 0.048 | NO |
| **11-beta-hydroxysteroid dehydrogenase 1 (11-DH) (11-beta-HSD1) (EC 1.1.1.146) (7-oxosteroid reductase) (EC 1.1.1.201) (Corticosteroid 11-beta-dehydrogenase isozyme 1) (Short chain dehydrogenase/reductase family 26C member 1)** | P28845 | HSD11B1 | -1.74 | <0.001 | 0.012 | NO |
| **cDNA FLJ55560, highly similar to Retinal dehydrogenase 2** | B4DH89 | NA | -1.73 | <0.001 | <0.001 | NO |
| **Dematin (Dematin actin-binding protein) (Erythrocyte membrane protein band 4.9)** | Q08495 | DMTN | -1.70 | 0.002 | 0.033 | YES |
| **G-protein coupled receptor 161 (G-protein coupled receptor RE2)** | Q8N6U8 | GPR161 | -1.70 | 0.002 | 0.026 | YES |
| **Band 3 anion transport protein (Anion exchange protein 1) (AE 1) (Anion exchanger 1) (Solute carrier family 4 member 1) (CD antigen CD233)** | P02730 | SLC4A1 | -1.69 | 0.003 | 0.040 | YES |
| **Spectrin alpha chain, erythrocytic 1 (Erythroid alpha-spectrin)** | P02549 | SPTA1 | -1.69 | 0.001 | 0.025 | NO |
| **Mitochondrial enolase superfamily member 1 (EC 4.2.1.68) (Antisense RNA to thymidylate synthase) (rTS) (L-fuconate dehydratase)** | Q7L5Y1 | ENOSF1 | -1.66 | <0.001 | <0.001 | NO |
| **cDNA FLJ55458, highly similar to Programmed cell death 6-interacting protein** | B4DHD2 | NA | -1.64 | 0.003 | 0.041 | NO |
| **Carbonic anhydrase 12 (EC 4.2.1.1) (Carbonate dehydratase XII) (Carbonic anhydrase XII) (CA-XII) (Tumor antigen HOM-RCC-3.1.3)** | O43570 | CA12 | -1.62 | <0.001 | 0.006 | NO |
| **Aminopeptidase B (AP-B) (EC 3.4.11.6) (Arginine aminopeptidase) (Arginyl aminopeptidase)** | Q9H4A4 | RNPEP | -1.62 | <0.001 | <0.001 | NO |
| **Spectrin beta chain, erythrocytic (Beta-I spectrin)** | P11277 | SPTB | -1.58 | 0.002 | 0.029 | YES |
| **Hemoglobin alpha-2 globin mutant** | Q4ZGM8 | NA | -1.57 | 0.002 | 0.032 | NO |
| **Ankyrin-1 (ANK-1) (Ankyrin-R) (Erythrocyte ankyrin)** | P16157 | ANK1 | -1.52 | 0.002 | 0.031 | YES |
| **Sulfotransferase** | A0A024QZB4 | hCG_1993905 | -1.52 | 0.001 | 0.023 | NO |
| **Cytosolic 5'-nucleotidase 3A (EC 3.1.3.5) (7-methylguanosine phosphate-specific 5'-nucleotidase) (7-methylguanosine nucleotidase) (EC 3.1.3.91) (Cytosolic 5'-nucleotidase 3) (Cytosolic 5'-nucleotidase III) (cN-III) (Pyrimidine 5'-nucleotidase 1) (P5'N-1) (P5N-1) (PN-I) (Uridine 5'-monophosphate hydrolase 1) (p36)** | Q9H0P0 | NT5C3A | -1.49 | <0.001 | 0.015 | NO |
| **Cdc42 effector protein 4 (Binder of Rho GTPases 4)** | Q9H3Q1 | CDC42EP4 | -1.48 | <0.001 | 0.009 | YES |
| **Glutamine synthetase (GS) (EC 6.3.1.2) (Glutamate--ammonia ligase) (Palmitoyltransferase GLUL) (EC 2.3.1.225)** | P15104 | GLUL | -1.46 | <0.001 | 0.007 | NO |
| **Arginase-1 (EC 3.5.3.1) (Liver-type arginase) (Type I arginase)** | P05089 | ARG1 | -1.46 | <0.001 | 0.014 | NO |
| **Proteasome assembly chaperone 1 (PAC-1) (Chromosome 21 leucine-rich protein) (C21-LRP) (Down syndrome critical region protein 2) (Proteasome chaperone homolog 1) (Pba1)** | O95456 | PSMG1 | -1.45 | 0.003 | 0.035 | NO |
| **Beta-2-microglobulin [Cleaved into: Beta-2-microglobulin form pI 5.3]** | P61769 | B2M | -1.44 | <0.001 | 0.010 | NO |
| **Annexin** | Q6ICS0 | ANXA11 | -1.44 | <0.001 | 0.017 | NO |
| **Katanin p80 WD40 repeat-containing subunit B1 (Katanin p80 subunit B1) (p80 katanin)** | Q9BVA0 | KATNB1 | -1.42 | 0.001 | 0.021 | NO |
| **6-phosphogluconate dehydrogenase, decarboxylating (EC 1.1.1.44)** | B4DL86 | NA | -1.41 | <0.001 | 0.005 | NO |
| **NA** | P01889 | NA | -1.39 | 0.004 | 0.048 | NO |
| **Tubulin polymerization-promoting protein (TPPP) (EC 3.6.5.-) (25 kDa brain-specific protein) (TPPP/p25) (p24) (p25-alpha)** | O94811 | TPPP | -1.39 | <0.001 | 0.006 | NO |
| **Leukocyte elastase inhibitor (LEI) (Monocyte/neutrophil elastase inhibitor) (EI) (M/NEI) (Peptidase inhibitor 2) (PI-2) (Serpin B1)** | P30740 | SERPINB1 | -1.38 | <0.001 | 0.010 | NO |
| **Ribosyldihydronicotinamide dehydrogenase [quinone] (EC 1.10.5.1) (NRH dehydrogenase [quinone] 2) (NRH:quinone oxidoreductase 2) (Quinone reductase 2) (QR2)** | P16083 | NQO2 | -1.37 | <0.001 | 0.008 | NO |
| **Erythrocyte membrane protein band 4.1 like 3 (cDNA FLJ77757)** | A8K968 | EPB41L3 | -1.36 | <0.001 | 0.010 | NO |
| **UDP-N-acetylglucosamine transferase subunit ALG14 (Asparagine-linked glycosylation 14 homolog)** | Q96F25 | ALG14 | -1.36 | <0.001 | 0.015 | NO |
| **Flavin reductase (NADPH) (FR) (EC 1.5.1.30) (Biliverdin reductase B) (BVR-B) (EC 1.3.1.-) (Biliverdin-IX beta-reductase) (Green heme-binding protein) (GHBP) (NADPH-dependent diaphorase) (NADPH-flavin reductase) (FLR) (S-nitroso-CoA-assisted nitrosyltransferase) (SNO-CoA-assisted nitrosyltransferase) (EC 2.6.99.-)** | P30043 | BLVRB | -1.36 | 0.001 | 0.022 | NO |
| **Aminopeptidase N (AP-N) (hAPN) (EC 3.4.11.2) (Alanyl aminopeptidase) (Aminopeptidase M) (AP-M) (Microsomal aminopeptidase) (Myeloid plasma membrane glycoprotein CD13) (gp150) (CD antigen CD13)** | P15144 | ANPEP | -1.35 | 0.002 | 0.029 | NO |
| **Beta-adducin (Erythrocyte adducin subunit beta)** | P35612 | ADD2 | -1.32 | 0.002 | 0.031 | NO |
| **Alpha-N-acetylglucosaminidase (EC 3.2.1.50) (N-acetyl-alpha-glucosaminidase) (NAG) [Cleaved into: Alpha-N-acetylglucosaminidase 82 kDa form; Alpha-N-acetylglucosaminidase 77 kDa form]** | P54802 | NAGLU | -1.29 | <0.001 | 0.003 | NO |
| **PDZ and LIM domain protein 1 (C-terminal LIM domain protein 1) (Elfin) (LIM domain protein CLP-36)** | O00151 | PDLIM1 | -1.29 | <0.001 | 0.006 | NO |
| **Insulin-like growth factor-binding protein 5 (IBP-5) (IGF-binding protein 5) (IGFBP-5)** | P24593 | IGFBP5 | -1.27 | <0.001 | 0.012 | YES |
| **Epiplakin OS=Homo sapiens GN=EPPK1 PE=1 SV=2** | P58107 | NEPPK1A | -1.27 | <0.001 | 0.004 | NO |
| **Oxidoreductase HTATIP2 (EC 1.1.1.-) (30 kDa HIV-1 TAT-interacting protein) (HIV-1 TAT-interactive protein 2)** | Q9BUP3 | HTATIP2 | -1.27 | <0.001 | 0.001 | NO |
| **Neurofilament medium polypeptide (160 kDa neurofilament protein) (Neurofilament 3) (Neurofilament triplet M protein)** | A5YM63 | NEFM | -1.27 | 0.004 | 0.046 | YES |
| **MHC Class I antigen** | G9I2K8 | HLA-B | -1.27 | 0.002 | 0.030 | NO |
| **Ubiquitin-associated domain-containing protein 1 (UBA domain-containing protein 1) (Glialblastoma cell differentiation-related protein 1) (Kip1 ubiquitination-promoting complex protein 2)** | Q9BSL1 | UBAC1 | -1.26 | <0.001 | 0.006 | NO |
| **Transforming growth factor beta receptor type 3 (TGF-beta receptor type 3) (TGFR-3) (Betaglycan) (Transforming growth factor beta receptor III) (TGF-beta receptor type III)** | Q03167 | TGFBR3 | -1.25 | 0.004 | 0.048 | NO |
| **SAM and SH3 domain-containing protein 1 (Proline-glutamate repeat-containing protein)** | O94885 | SASH1 | -1.25 | <0.001 | 0.001 | YES |
| **NAD(P)H dehydrogenase [quinone] 1 (EC 1.6.5.2) (Azoreductase) (DT-diaphorase) (DTD) (Menadione reductase) (NAD(P)H:quinone oxidoreductase 1) (Phylloquinone reductase) (Quinone reductase 1) (QR1)** | P15559 | NQO1 | -1.24 | 0.002 | 0.028 | NO |
| **Protein S100-A4 (Calvasculin) (Metastasin) (Placental calcium-binding protein) (Protein Mts1) (S100 calcium-binding protein A4)** | P26447 | S100A4 | -1.23 | <0.001 | 0.006 | NO |
| **Parathymosin** | P20962 | PTMS | -1.22 | <0.001 | 0.002 | NO |
| **Ribose-phosphate pyrophosphokinase 2 (EC 2.7.6.1) (PPRibP) (Phosphoribosyl pyrophosphate synthase II) (PRS-II)** | P11908 | PRPS2 | -1.22 | <0.001 | 0.003 | NO |
| **Endosialin (Tumor endothelial marker 1) (CD antigen CD248)** | Q9HCU0 | CD248 | -1.21 | <0.001 | 0.001 | YES |
| **Inactive phospholipase C-like protein 1 (PLC-L1) (Phospholipase C-deleted in lung carcinoma) (Phospholipase C-related but catalytically inactive protein) (PRIP)** | Q15111 | PLCL1 | -1.21 | <0.001 | 0.006 | YES |
| **Activating signal cointegrator 1 complex subunit 2 (ASC-1 complex subunit p100) (Trip4 complex subunit p100)** | Q9H1I8 | ASCC2 | -1.20 | <0.001 | 0.004 | NO |
| **F-box only protein 7** | Q9Y3I1 | FBXO7 | -1.19 | <0.001 | 0.010 | NO |
| **Methanethiol oxidase (MTO) (EC 1.8.3.4) (56 kDa selenium-binding protein) (SBP56) (SP56) (Selenium-binding protein 1)** | Q13228 | SELENBP1 | -1.19 | <0.001 | 0.016 | NO |
| **Sodium/hydrogen exchanger 9 (Na(+)/H(+) exchanger 9) (NHE-9) (Solute carrier family 9 member 9)** | Q8IVB4 | SLC9A9 | -1.18 | <0.001 | 0.014 | NO |
| **Nuclear receptor 2C2-associated protein (TR4 orphan receptor-associated 16 kDa protein)** | Q86WQ0 | NR2C2AP | -1.18 | <0.001 | 0.018 | NO |
| **Aldehyde dehydrogenase family 3 member B1 (EC 1.2.1.28) (EC 1.2.1.5) (EC 1.2.1.7) (Aldehyde dehydrogenase 7) (Long-chain fatty aldehyde dehydrogenase) (EC 1.2.1.48) (Medium-chain fatty aldehyde dehydrogenase)** | P43353 | ALDH3B1 | -1.17 | 0.003 | 0.040 | NO |
| **Protein phosphatase inhibitor 2 (IPP-2)** | P41236 | PPP1R2 | -1.16 | <0.001 | 0.009 | YES |
| **Caspase-6 (CASP-6) (CSP-6) (EC 3.4.22.59) (Apoptotic protease Mch-2) [Cleaved into: Caspase-6 subunit p18 (Caspase-6 subunit p20); Caspase-6 subunit p11 (Caspase-6 subunit p10)]** | P55212 | CASP6 | -1.16 | 0.001 | 0.024 | NO |
| **Serine hydroxymethyltransferase, cytosolic (SHMT) (EC 2.1.2.1) (Glycine hydroxymethyltransferase) (Serine methylase)** | P34896 | SHMT1 | -1.16 | <0.001 | 0.003 | NO |
| **Galactose mutarotase (EC 5.1.3.3) (Aldose 1-epimerase)** | Q96C23 | GALM | -1.16 | <0.001 | 0.006 | NO |
| **Nucleolar protein 3 (Apoptosis repressor with CARD) (Muscle-enriched cytoplasmic protein) (Myp) (Nucleolar protein of 30 kDa) (Nop30)** | O60936 | NOL3 | -1.14 | 0.004 | 0.049 | YES |
| **Putative monooxygenase p33MONOX (EC 1.-.-.-) (Brain-derived rescue factor p60MONOX) (Flavin monooxygenase motif-containing protein of 33 kDa)** | Q96A73 | KIAA1191 | -1.14 | <0.001 | 0.006 | NO |
| **Forkhead box protein O1 (Forkhead box protein O1A) (Forkhead in rhabdomyosarcoma)** | Q12778 | FOXO1 | -1.14 | <0.001 | 0.002 | YES |
| **Acylphosphatase-1 (EC 3.6.1.7) (Acylphosphatase, erythrocyte isozyme) (Acylphosphatase, organ-common type isozyme) (Acylphosphate phosphohydrolase 1)** | P07311 | ACYP1 | -1.13 | 0.003 | 0.043 | NO |
| **Thymidine phosphorylase (TP) (EC 2.4.2.4) (Gliostatin) (Platelet-derived endothelial cell growth factor) (PD-ECGF) (TdRPase)** | P19971 | TYMP | -1.12 | 0.001 | 0.023 | NO |
| **Retinal dehydrogenase 2 (RALDH 2) (RalDH2) (EC 1.2.1.36) (Aldehyde dehydrogenase family 1 member A2) (ALDH1A2) (Retinaldehyde-specific dehydrogenase type 2) (RALDH(II))** | O94788 | ALDH1A2 | -1.12 | <0.001 | 0.001 | NO |
| **Adipose-secreted signaling protein** | Q9GZN8 | ADISSP | -1.12 | 0.002 | 0.027 | NO |
| **MHC class I antigen** | Q546C9 | HLA-B | -1.11 | 0.002 | 0.028 | YES |
| **COUP transcription factor 2 (COUP-TF2) (Apolipoprotein A-I regulatory protein 1) (ARP-1) (COUP transcription factor II) (COUP-TF II) (Nuclear receptor subfamily 2 group F member 2)** | P24468 | NR2F2 | -1.10 | 0.003 | 0.037 | NO |
| **Ubiquitin carboxyl-terminal hydrolase isozyme L3 (UCH-L3) (EC 3.4.19.12) (Ubiquitin thioesterase L3)** | P15374 | UCHL3 | -1.10 | 0.004 | 0.045 | NO |
| **Angiotensin-converting enzyme (ACE) (EC 3.4.15.1) (Dipeptidyl carboxypeptidase I) (Kininase II) (CD antigen CD143) [Cleaved into: Angiotensin-converting enzyme, soluble form]** | P12821 | ACE | -1.10 | <0.001 | 0.015 | NO |
| **UBX domain-containing protein 6 (UBX domain-containing protein 1)** | Q9BZV1 | UBXN6 | -1.09 | <0.001 | 0.007 | NO |
| **Thioredoxin (Trx) (ATL-derived factor) (ADF) (Surface-associated sulphydryl protein) (SASP) (allergen Hom s Trx)** | P10599 | TXN | -1.06 | 0.002 | 0.029 | NO |
| **NmrA-like family domain-containing protein 1** | Q9HBL8 | NMRAL1 | -1.06 | <0.001 | 0.008 | NO |
| **RNA binding motif (RNP1, RRM) protein 3, isoform CRA_c** | A0A024QYX3 | RBM3 | -1.05 | <0.001 | 0.004 | NO |
| **Coiled-coil domain-containing protein 43** | Q96MW1 | CCDC43 | -1.03 | <0.001 | 0.001 | NO |
| **MHC class I antigen** | F6IQY8 | HLA-A | -1.02 | 0.004 | 0.048 | NO |
| **Protein-lysine N-trimethyltransferase SMYD5 (EC 2.1.1.-) (Protein NN8-4AG) (Retinoic acid-induced protein 15) (SET and MYND domain-containing protein 5) ([histone H3]-lysine20 N-trimethyltransferase SMYD5) (EC 2.1.1.372) ([histone H4]-lysine36 N-trimethyltransferase SMYD5) (EC 2.1.1.359)** | Q6GMV2 | SMYD5 | -1.01 | <0.001 | 0.006 | NO |
| **A-kinase anchor protein 12 (AKAP-12) (A-kinase anchor protein 250 kDa) (AKAP 250) (Gravin) (Myasthenia gravis autoantigen)** | Q02952 | AKAP12 | -1.00 | <0.001 | 0.004 | YES |
| **Thymosin beta-10** | P63313 | TMSB10 | -1.00 | 0.001 | 0.024 | NO |
| **Actin-binding LIM protein 1 (abLIM-1) (Actin-binding LIM protein family member 1) (Actin-binding double zinc finger protein) (LIMAB1) (Limatin)** | O14639 | ABLIM1 | -1.00 | <0.001 | 0.016 | YES |
|  |  |  |  |  |  |  |

**Supplementary Information 10:** Total proteins which significantly (p≤0.05) at least doubled and passed FDR (q≤0.05) in the failed IOL group under the contracted-phase during oxytocin-induced contractions condition, ordered by log-fold change.

|  |  |  |  |  |  |  |
| --- | --- | --- | --- | --- | --- | --- |
| **Protein name** | **Accession** | **Gene name** | **Log Fold-change** | **p-value** | **q-value** | **Phosphorylation identified** |
| **Choline transporter-like protein** | A0A088QCU6 | SLC44A2 | 5.76 | <0.001 | 0.006 | No |
| **Desmuslin, isoform CRA_a (Synemin)** | A0A075B7B1 | SYNM | 5.70 | <0.001 | 0.002 | No |
| **Zinc finger, UBR1 type 1, isoform CRA_c** | A0A024RAC9 | ZUBR1 | 4.39 | <0.001 | <0.001 | No |
| **maleylacetoacetate isomerase (EC 5.2.1.2)** | G3V4T6 | GSTZ1 | 4.28 | <0.001 | <0.001 | No |
| **Thrombospondin-4** | P35443 | THBS4 | 4.14 | <0.001 | <0.001 | No |
| **Cartilage oligomeric matrix protein (COMP) (Thrombospondin-5) (TSP5)** | P49747 | COMP | 4.02 | <0.001 | 0.001 | No |
| **Keratin, type II cytoskeletal 5 (Cytokeratin-5) (CK-5) (Keratin-5) (K5) (Type-II keratin Kb5)** | Q5XQN5 | KRT5 | 2.95 | <0.001 | 0.006 | No |
| **Matrix Gla protein (MGP) (Cell growth-inhibiting gene 36 protein)** | P08493 | MGP | 2.90 | <0.001 | <0.001 | No |
| **TNC variant protein** | Q4LE33 | TNC variant protein | 2.74 | <0.001 | <0.001 | YES |
| **Transforming growth factor-beta-induced protein ig-h3 (Beta ig-h3) (Kerato-epithelin) (RGD-containing collagen-associated protein) (RGD-CAP)** | Q15582 | TGFBI | 2.73 | <0.001 | <0.001 | No |
| **Thrombospondin 1, isoform CRA_a** | A0A024R9Q1 | THBS1 | 2.67 | <0.001 | <0.001 | No |
| **Annexin A3 (35-alpha calcimedin) (Annexin III) (Annexin-3) (Inositol 1,2-cyclic phosphate 2-phosphohydrolase) (Lipocortin III) (Placental anticoagulant protein III) (PAP-III)** | P12429 | ANXA3 | 2.62 | <0.001 | <0.001 | No |
| **Keratin, type I cytoskeletal 9 (Cytokeratin-9) (CK-9) (Keratin-9) (K9)** | P35527 | KRT9 | 2.60 | <0.001 | 0.004 | No |
| **Carbonic anhydrase III, muscle specific, isoform CRA_a** | A0A024R825 | CA3 | 2.46 | <0.001 | 0.011 | No |
| **Biglycan** | C9JKG1 | BGN | 2.45 | <0.001 | 0.009 | No |
| **Collagen alpha-1(XII) chain** | Q99715 | COL12A1 | 2.35 | <0.001 | 0.002 | No |
| **N90-VRC38.08 heavy chain variable region** | A0A1W6IYI5 | NA | 2.32 | <0.001 | 0.003 | No |
| **D-3-phosphoglycerate dehydrogenase (3-PGDH) (EC 1.1.1.95) (2-oxoglutarate reductase) (EC 1.1.1.399) (Malate dehydrogenase) (EC 1.1.1.37)** | O43175 | PHGDH | 2.31 | <0.001 | 0.004 | No |
| **Keratin, type II cytoskeletal 1 (Cytokeratin-1) (Keratin-1) (Type-II keratin Kb1)** | H6VRG2 | KRT1 | 2.28 | <0.001 | 0.013 | No |
| **Biglycan (Bone/cartilage proteoglycan I) (PG-S1)** | P21810 | BGN | 2.25 | <0.001 | <0.001 | No |
| **PG-M** | Q6MZK8 | DKFZp686K06110 | 2.25 | <0.001 | 0.006 | YES |
| **Matrilin-2** | O00339 | MATN2 | 2.22 | <0.001 | <0.001 | No |
| **Keratin, type I cytoskeletal 10 (Cytokeratin-10) (CK-10) (Keratin-10) (K10)** | P13645 | KRT10 | 2.16 | 0.002 | 0.040 | No |
| **Tenascin-X** | A0A087WWA5 | TNXB | 2.12 | <0.001 | 0.012 | No |
| **Immunoglobulin heavy constant gamma 4 (Ig gamma-4 chain C region)** | P01861 | IGHG4 | 2.11 | 0.002 | 0.040 | No |
| **Tenascin-X (TN-X) (Hexabrachion-like protein)** | P22105 | TNXB | 2.11 | <0.001 | <0.001 | YES |
| **Polypeptide N-acetylgalactosaminyltransferase 18 (EC 2.4.1.41) (Polypeptide GalNAc transferase 18) (GalNAc-T18) (Polypeptide GalNAc transferase-like protein 4) (GalNAc-T-like protein 4) (pp-GaNTase-like protein 4) (Polypeptide N-acetylgalactosaminyltransferase-like protein 4) (Protein-UDP acetylgalactosaminyltransferase-like protein 4) (UDP-GalNAc:polypeptide N-acetylgalactosaminyltransferase-like protein 4)** | Q6P9A2 | GALNT18 | 2.00 | <0.001 | 0.002 | No |
| **Serpin peptidase inhibitor, clade E (Nexin, plasminogen activator inhibitor type 1), member 1, isoform CRA_b** | A0A024QYT5 | SERPINE1 | 1.98 | <0.001 | 0.003 | No |
| **Prostaglandin G/H synthase 1 (EC 1.14.99.1) (Cyclooxygenase-1) (COX-1) (Prostaglandin H2 synthase 1) (PGH synthase 1) (PGHS-1) (PHS 1) (Prostaglandin-endoperoxide synthase 1)** | P23219 | PTGS1 | 1.97 | <0.001 | <0.001 | No |
| **Cadherin 2, type 1, N-cadherin (Neuronal), isoform CRA_b** | A0A024RC42 | CDH2 | 1.92 | <0.001 | <0.001 | No |
| **DNA repair protein XRCC1 (X-ray repair cross-complementing protein 1)** | P18887 | XRCC1 | 1.91 | 0.003 | 0.047 | No |
| **Periostin, osteoblast specific factor, isoform CRA_a** | A0A024RDT5 | POSTN | 1.88 | 0.001 | 0.032 | No |
| **[Pyruvate dehydrogenase (acetyl-transferring)] kinase isozyme 1, mitochondrial (EC 2.7.11.2) (Pyruvate dehydrogenase kinase isoform 1) (PDH kinase 1)** | Q15118 | PDK1 | 1.82 | <0.001 | 0.002 | No |
| **Collagen alpha-1(XV) chain [Cleaved into: Restin (Endostatin-XV) (Related to endostatin) (Restin-I); Restin-2 (Restin-II); Restin-3 (Restin-III); Restin-4 (Restin-IV)]** | P39059 | COL15A1 | 1.81 | <0.001 | 0.004 | No |
| **L-amino-acid oxidase (LAAO) (LAO) (EC 1.4.3.2) (EC 1.4.3.25) (Interleukin-4-induced protein 1) (IL4-induced protein 1) (hIL4I1) (Protein Fig-1) (hFIG1)** | Q96RQ9 | IL4I1 | 1.79 | 0.002 | 0.036 | No |
| **Fibrillin-2 [Cleaved into: Placensin]** | P35556 | FBN2 | 1.77 | <0.001 | 0.015 | No |
| **Mimecan (Osteoglycin) (Osteoinductive factor) (OIF)** | P20774 | OGN | 1.76 | <0.001 | 0.012 | No |
| **SPARC-related modular calcium-binding protein 1 (Secreted modular calcium-binding protein 1) (SMOC-1)** | Q9H4F8 | SMOC1 | 1.72 | 0.001 | 0.032 | No |
| **Myosin-10 (Cellular myosin heavy chain, type B) (Myosin heavy chain 10) (Myosin heavy chain, non-muscle IIb) (Non-muscle myosin heavy chain B) (NMMHC-B) (Non-muscle myosin heavy chain IIb) (NMMHC II-b) (NMMHC-IIB)** | P35580 | MYH10 | 1.72 | <0.001 | 0.007 | YES |
| **Asporin (Periodontal ligament-associated protein 1) (PLAP-1)** | Q9BXN1 | ASPN | 1.67 | <0.001 | 0.008 | No |
| **Ribonuclease T2 (EC 4.6.1.19) (Ribonuclease 6)** | O00584 | RNASET2 | 1.64 | <0.001 | 0.002 | No |
| **Zinc finger FYVE domain-containing protein 26 (FYVE domain-containing centrosomal protein) (FYVE-CENT) (Spastizin)** | Q68DK2 | ZFYVE26 | 1.64 | <0.001 | 0.022 | No |
| **Lysyl oxidase homolog 2 (EC 1.4.3.13) (Lysyl oxidase-like protein 2) (Lysyl oxidase-related protein 2) (Lysyl oxidase-related protein WS9-14)** | Q9Y4K0 | LOXL2 | 1.62 | <0.001 | <0.001 | No |
| **Latent-transforming growth factor beta-binding protein 2 (LTBP-2)** | Q14767 | LTBP2 | 1.61 | <0.001 | 0.002 | No |
| **Collagen alpha-1(XVI) chain** | Q07092 | COL16A1 | 1.61 | 0.001 | 0.027 | No |
| **Prostacyclin synthase (EC 5.3.99.4) (Hydroperoxy icosatetraenoate dehydratase) (EC 4.2.1.152) (Prostaglandin I2 synthase)** | Q16647 | PTGIS | 1.59 | <0.001 | <0.001 | No |
| **Septin-6** | Q14141 | SEPTIN6 | 1.55 | <0.001 | <0.001 | No |
| **Corrinoid adenosyltransferase MMAB (EC 2.5.1.-) (ATP:co(I)rrinoid adenosyltransferase MMAB) (Methylmalonic aciduria type B protein)** | Q96EY8 | MMAB | 1.55 | <0.001 | 0.005 | No |
| **C14orf159 variant protein** | Q4LE40 | C14orf159 variant protein | 1.54 | <0.001 | <0.001 | No |
| **Synaptophysin-like protein 2** | Q5VXT5 | SYPL2 | 1.52 | <0.001 | 0.004 | No |
| **Core histone macro-H2A.1 (Histone macroH2A1) (mH2A1) (Histone H2A.y) (H2A/y) (Medulloblastoma antigen MU-MB-50.205)** | O75367 | MACROH2A1 | 1.51 | 0.002 | 0.040 | No |
| **Cyclin-dependent kinase 2-associated protein 2 (CDK2-associated protein 2) (DOC-1-related protein) (DOC-1R)** | O75956 | CDK2AP2 | 1.51 | 0.001 | 0.031 | No |
| **cDNA FLJ51896, highly similar to Glia-derived nexin** | B4DMR3 | NA | 1.50 | <0.001 | 0.012 | No |
| **Adipocyte enhancer-binding protein 1 (AE-binding protein 1) (Aortic carboxypeptidase-like protein)** | Q8IUX7 | AEBP1 | 1.49 | <0.001 | 0.006 | No |
| **SAA2-SAA4 readthrough** | A0A096LPE2 | SAA2-SAA4 | 1.38 | <0.001 | 0.002 | No |
| **Inter-alpha-trypsin inhibitor heavy chain H1 (ITI heavy chain H1) (ITI-HC1) (Inter-alpha-inhibitor heavy chain 1) (Inter-alpha-trypsin inhibitor complex component III) (Serum-derived hyaluronan-associated protein) (SHAP)** | P19827 | ITIH1 | 1.38 | <0.001 | 0.001 | No |
| **SH3 domain-binding glutamic acid-rich-like protein 2 (Fovea-associated SH3 domain-binding protein)** | Q9UJC5 | SH3BGRL2 | 1.35 | <0.001 | 0.002 | No |
| **Procollagen-lysine,2-oxoglutarate 5-dioxygenase 2 (EC 1.14.11.4) (Lysyl hydroxylase 2) (LH2)** | O00469 | PLOD2 | 1.35 | <0.001 | 0.005 | No |
| **glutaminase (EC 3.5.1.2)** | B3KM58 | NA | 1.33 | <0.001 | 0.004 | No |
| **Dual adapter for phosphotyrosine and 3-phosphotyrosine and 3-phosphoinositide (hDAPP1) (B lymphocyte adapter protein Bam32) (B-cell adapter molecule of 32 kDa)** | Q9UN19 | DAPP1 | 1.33 | 0.002 | 0.040 | No |
| **Matrix metalloproteinase-19 (MMP-19) (EC 3.4.24.-) (Matrix metalloproteinase RASI) (Matrix metalloproteinase-18) (MMP-18)** | Q99542 | MMP19 | 1.30 | <0.001 | 0.007 | No |
| **Glutaminase kidney isoform, mitochondrial (GLS) (EC 3.5.1.2) (K-glutaminase) (L-glutamine amidohydrolase) [Cleaved into: Glutaminase kidney isoform, mitochondrial 68 kDa chain; Glutaminase kidney isoform, mitochondrial 65 kDa chain]** | O94925 | GLS | 1.29 | <0.001 | 0.006 | No |
| **Thrombospondin-3** | P49746 | THBS3 | 1.29 | <0.001 | 0.003 | No |
| **Extracellular superoxide dismutase [Cu-Zn] (EC-SOD) (EC 1.15.1.1)** | P08294 | SOD3 | 1.28 | <0.001 | 0.009 | No |
| **NADH:ubiquinone oxidoreductase MLRQ subunit homolog, isoform CRA_a** | A0A024RB39 | LOC56901 | 1.26 | <0.001 | 0.021 | No |
| **Twisted gastrulation protein homolog 1** | Q9GZX9 | TWSG1 | 1.26 | <0.001 | 0.016 | No |
| **Pentraxin-related protein PTX3 (Pentaxin-related protein PTX3) (Tumor necrosis factor alpha-induced protein 5) (TNF alpha-induced protein 5) (Tumor necrosis factor-inducible gene 14 protein) (TSG-14)** | P26022 | PTX3 | 1.22 | 0.002 | 0.033 | No |
| **Latent-transforming growth factor beta-binding protein 4 (LTBP-4)** | Q8N2S1 | LTBP4 | 1.22 | <0.001 | 0.019 | No |
| **Zinc transporter 7 (ZnT-7) (Solute carrier family 30 member 7) (Znt-like transporter 2)** | Q8NEW0 | SLC30A7 | 1.21 | <0.001 | 0.012 | No |
| **Uncharacterized protein DKFZp686O1553** | Q5HYM1 | DKFZp686O1553 | 1.21 | <0.001 | 0.002 | No |
| **Liver carboxylesterase 1 (Acyl-coenzyme A:cholesterol acyltransferase) (ACAT) (Brain carboxylesterase hBr1) (Carboxylesterase 1) (CE-1) (hCE-1) (EC 3.1.1.1) (Cholesteryl ester hydrolase) (CEH) (EC 3.1.1.13) (Cocaine carboxylesterase) (Egasyn) (HMSE) (Methylumbelliferyl-acetate deacetylase 1) (EC 3.1.1.56) (Monocyte/macrophage serine esterase) (Retinyl ester hydrolase) (REH) (Serine esterase 1) (Triacylglycerol hydrolase) (TGH)** | P23141 | CES1 | 1.19 | <0.001 | 0.012 | No |
| **Bifunctional methylenetetrahydrofolate dehydrogenase/cyclohydrolase, mitochondrial [Includes: NAD-dependent methylenetetrahydrofolate dehydrogenase (EC 1.5.1.15); Methenyltetrahydrofolate cyclohydrolase (EC 3.5.4.9)]** | P13995 | MTHFD2 | 1.19 | <0.001 | 0.004 | No |
| **Alpha-catulin (Alpha-catenin-related protein) (ACRP) (Catenin alpha-like protein 1)** | Q9UBT7 | CTNNAL1 | 1.18 | <0.001 | 0.026 | No |
| **Chromosome 1 open reading frame 24, isoform CRA_a** | A0A024R978 | C1orf24 | 1.14 | 0.001 | 0.028 | No |
| **FXYD domain containing ion transport regulator 6, isoform CRA_a** | A0A024R3J8 | FXYD6 | 1.13 | 0.002 | 0.036 | No |
| **Glypican-4 (K-glypican) [Cleaved into: Secreted glypican-4]** | O75487 | GPC4 | 1.13 | <0.001 | 0.004 | YES |
| **Acrosomal protein KIAA1210** | Q9ULL0 | KIAA1210 | 1.12 | <0.001 | 0.025 | No |
| **Disheveled-associated activator of morphogenesis 2** | Q86T65 | DAAM2 | 1.12 | 0.002 | 0.035 | No |
| **Vitamin D binding protein** | A0A1B1CYC5 | Gc | 1.11 | <0.001 | 0.025 | No |
| **Myosin-9 (Cellular myosin heavy chain, type A) (Myosin heavy chain 9) (Myosin heavy chain, non-muscle IIa) (Non-muscle myosin heavy chain A) (NMMHC-A) (Non-muscle myosin heavy chain IIa) (NMMHC II-a) (NMMHC-IIA)** | P35579 | MYH9 | 1.09 | 0.001 | 0.032 | YES |
| **RAN binding protein 9, isoform CRA_a** | A0A024QZW3 | RANBP9 | 1.09 | 0.003 | 0.044 | No |
| **Dihydropyrimidinase-related protein 1 (DRP-1) (Collapsin response mediator protein 1) (CRMP-1) (Inactive dihydropyrimidinase) (Unc-33-like phosphoprotein 3) (ULIP-3)** | Q14194 | CRMP1 | 1.07 | 0.003 | 0.044 | No |
| **Latent-transforming growth factor beta-binding protein 1 (LTBP-1) (Transforming growth factor beta-1-binding protein 1) (TGF-beta1-BP-1)** | Q14766 | LTBP1 | 1.04 | 0.003 | 0.044 | No |
| **procollagen-lysine 5-dioxygenase (EC 1.14.11.4)** | B4DHG3 | NA | 1.03 | 0.002 | 0.033 | No |
| **DNA helicase MCM8 (EC 3.6.4.12) (Minichromosome maintenance 8)** | Q9UJA3 | MCM8 | 1.03 | 0.001 | 0.027 | No |
| **Neurofilament light polypeptide isoform 2** | A0A0S2Z4B1 | NEFL | 1.03 | 0.002 | 0.040 | No |
| **von Willebrand factor A domain-containing protein 1** | Q6PCB0 | VWA1 | 1.03 | 0.002 | 0.038 | No |
| **Junctional adhesion molecule B (JAM-B) (Junctional adhesion molecule 2) (JAM-2) (Vascular endothelial junction-associated molecule) (VE-JAM) (CD antigen CD322)** | P57087 | JAM2 | 1.03 | <0.001 | 0.006 | No |
| **Kynurenine--oxoglutarate transaminase 3 (EC 2.6.1.7) (Cysteine-S-conjugate beta-lyase 2) (EC 4.4.1.13) (Kynurenine aminotransferase 3) (Kynurenine aminotransferase III) (KATIII) (Kynurenine--glyoxylate transaminase) (EC 2.6.1.63) (Kynurenine--oxoglutarate transaminase III)** | Q6YP21 | KYAT3 | 1.02 | <0.001 | 0.006 | No |
| **Sarcospan (K-ras oncogene-associated protein) (Kirsten-ras-associated protein)** | Q14714 | SSPN | 1.01 | 0.001 | 0.032 | No |
| **Monocarboxylate transporter 2 (MCT 2) (Solute carrier family 16 member 7)** | O60669 | SLC16A7 | 1.00 | 0.001 | 0.028 | YES |
|  |  |  |  |  |  |  |

**Supplementary Information 11:** Total proteins which significantly (p≤0.05) at least halved and passed FDR (q≤0.05) in the failed IOL group under the contracted-phase during oxytocin-induced contractions condition, ordered by log-fold change.

|  |  |  |  |  |  |  |
| --- | --- | --- | --- | --- | --- | --- |
| **Protein name** | **Accession** | **Gene name** | **Log Fold-change** | **p-value** | **q-value** | **Phosphorylation identified** |
| **Glutathione S-transferase Mu 1 (EC 2.5.1.18) (GST HB subunit 4) (GST class-mu 1) (GSTM1-1) (GSTM1a-1a) (GSTM1b-1b) (GTH4)** | P09488 | GSTM1 | -7.15 | <0.001 | <0.001 | NO |
| **MHC class I antigen** | A0A1G4HPS6 | HLA-A | -7.02 | <0.001 | <0.001 | NO |
| **MHC class I antigen** | A0A1X9I3T8 | HLA-A | -6.05 | <0.001 | <0.001 | NO |
| **MHC class II antigen** | A0A1Y6MRR4 | HLA-DPB1 | -5.28 | <0.001 | <0.001 | NO |
| **Angiotensin-converting enzyme 2 (EC 3.4.17.23) (Angiotensin-converting enzyme homolog) (ACEH) (Angiotensin-converting enzyme-related carboxypeptidase) (ACE-related carboxypeptidase) (EC 3.4.17.-) (Metalloprotease MPROT15) [Cleaved into: Processed angiotensin-converting enzyme 2]** | Q9BYF1 | ACE2 | -5.04 | <0.001 | <0.001 | NO |
| **Endoplasmic reticulum DnaJ/PDI fusion protein 3** | Q6YPB0 | NA | -4.60 | <0.001 | <0.001 | NO |
| **MHC class I antigen** | A0A1X9I444 | HLA-B | -4.08 | <0.001 | <0.001 | NO |
| **RRBP1 protein** | A1A5C4 | RRBP1 | -3.90 | <0.001 | <0.001 | NO |
| **MHC class I antigen** | A0A0S4T3R3 | HLA-C | -3.51 | <0.001 | 0.001 | YES |
| **MHC class I antigen** | Q2LE77 | HLA-Cw | -3.22 | <0.001 | 0.006 | NO |
| **P2X purinoceptor 1 (P2X1) (ATP receptor) (Purinergic receptor)** | P51575 | P2RX1 | -3.11 | 0.002 | 0.040 | NO |
| **MHC class II antigen** | K4RIC6 | HLA-DRB1 | -3.07 | <0.001 | 0.002 | NO |
| **cDNA FLJ55458, highly similar to Programmed cell death 6-interacting protein** | B4DHD2 | NA | -3.04 | <0.001 | 0.002 | NO |
| **Hemoglobin subunit delta** | E9PEW8 | HBD | -2.90 | 0.003 | 0.045 | NO |
| **Hemoglobin subunit mu (Hemoglobin mu chain) (Mu-globin)** | Q6B0K9 | HBM | -2.74 | 0.003 | 0.049 | NO |
| **Endoplasmic reticulum aminopeptidase 2 (EC 3.4.11.-) (Leukocyte-derived arginine aminopeptidase) (L-RAP)** | Q6P179 | ERAP2 | -2.73 | <0.001 | <0.001 | NO |
| **Leukocyte immunoglobulin-like receptor subfamily B member 5 (CD85 antigen-like family member C) (Leukocyte immunoglobulin-like receptor 8) (LIR-8) (CD antigen CD85c)** | O75023 | LILRB5 | -2.70 | <0.001 | <0.001 | NO |
| **Epiplakin (450 kDa epidermal antigen)** | P58107 | EPPK1 | -2.61 | <0.001 | 0.006 | YES |
| **NA** | P01889 | NA | -2.46 | <0.001 | 0.003 | NO |
| **Hemoglobin delta-beta fusion protein** | Q5XTR9 | HBD/HBB | -2.43 | 0.002 | 0.033 | NO |
| **Aminopeptidase N (EC 3.4.11.2)** | B4DPH5 | NA | -2.32 | 0.002 | 0.033 | NO |
| **Glutathione S-transferase theta-2B (EC 2.5.1.18) (Glutathione S-transferase theta-2) (GST class-theta-2)** | P0CG30 | GSTT2B | -2.32 | <0.001 | <0.001 | NO |
| **Beta-globin protein** | Q9UM85 | beta-globin | -2.30 | <0.001 | 0.013 | NO |
| **Intraflagellar transport protein 140 homolog (WD and tetratricopeptide repeats protein 2)** | Q96RY7 | IFT140 | -2.24 | <0.001 | 0.017 | NO |
| **Neprilysin (EC 3.4.24.11) (Atriopeptidase) (Common acute lymphocytic leukemia antigen) (CALLA) (Enkephalinase) (Neutral endopeptidase 24.11) (NEP) (Neutral endopeptidase) (Skin fibroblast elastase) (SFE) (CD antigen CD10)** | P08473 | MME | -2.22 | <0.001 | <0.001 | NO |
| **Ig-like domain-containing protein** | B7ZW57 | NA | -2.15 | <0.001 | 0.005 | NO |
| **Ephrin-B1 (EFL-3) (ELK ligand) (ELK-L) (EPH-related receptor tyrosine kinase ligand 2) (LERK-2) [Cleaved into: Ephrin-B1 C-terminal fragment (Ephrin-B1 CTF); Ephrin-B1 intracellular domain (Ephrin-B1 ICD)]** | P98172 | EFNB1 | -2.15 | <0.001 | <0.001 | NO |
| **Glutathione S-transferase Mu 4 (EC 2.5.1.18) (GST class-mu 4) (GST-Mu2) (GSTM4-4) (Leukotriene C4 synthase GSTM4) (EC 4.4.1.20)** | Q03013 | GSTM4 | -2.13 | <0.001 | 0.010 | NO |
| **Alpha-hemoglobin-stabilizing protein (Erythroid differentiation-related factor) (Erythroid-associated factor)** | Q9NZD4 | AHSP | -2.07 | <0.001 | 0.019 | NO |
| **Carbonic anhydrase 1 (EC 4.2.1.1) (Carbonate dehydratase I) (Carbonic anhydrase B) (CAB) (Carbonic anhydrase I) (CA-I) (Cyanamide hydratase CA1) (EC 4.2.1.69)** | P00915 | CA1 | -2.03 | 0.002 | 0.038 | NO |
| **Aldehyde dehydrogenase family 3 member B1 (EC 1.2.1.28) (EC 1.2.1.5) (EC 1.2.1.7) (Aldehyde dehydrogenase 7) (Long-chain fatty aldehyde dehydrogenase) (EC 1.2.1.48) (Medium-chain fatty aldehyde dehydrogenase)** | P43353 | ALDH3B1 | -2.01 | <0.001 | 0.003 | NO |
| **Glycogen phosphorylase, brain form (EC 2.4.1.1)** | P11216 | PYGB | -2.00 | <0.001 | 0.002 | NO |
| **Hemoglobin beta chain variant Hb.Sinai-Bel Air** | Q8IUL9 | HBB | -1.97 | 0.003 | 0.049 | NO |
| **Scavenger receptor class A member 5 (Scavenger receptor hlg)** | Q6ZMJ2 | SCARA5 | -1.97 | <0.001 | 0.006 | NO |
| **Zinc finger protein 648** | Q5T619 | ZNF648 | -1.95 | 0.003 | 0.044 | NO |
| **Carbonic anhydrase 2 (EC 4.2.1.1) (Carbonate dehydratase II) (Carbonic anhydrase C) (CAC) (Carbonic anhydrase II) (CA-II) (Cyanamide hydratase CA2) (EC 4.2.1.69)** | P00918 | CA2 | -1.94 | 0.002 | 0.039 | NO |
| **Carbonic anhydrase 3 (EC 4.2.1.1) (Carbonate dehydratase III) (Carbonic anhydrase III) (CA-III)** | P07451 | CA3 | -1.93 | <0.001 | 0.013 | NO |
| **Platelet glycoprotein Ib beta chain (GP-Ib beta) (GPIb-beta) (GPIbB) (Antigen CD42b-beta) (CD antigen CD42c)** | P13224 | GP1BB | -1.92 | 0.003 | 0.043 | NO |
| **Phosphatidylcholine transfer protein (PC-TP) (START domain-containing protein 2) (StARD2) (StAR-related lipid transfer protein 2)** | Q9UKL6 | PCTP | -1.92 | <0.001 | 0.002 | NO |
| **Hemoglobin beta subunit variant** | Q9UK54 | HBB | -1.91 | 0.003 | 0.050 | NO |
| **Fatty acid-binding protein 5 (Epidermal-type fatty acid-binding protein) (E-FABP) (Fatty acid-binding protein, epidermal) (Psoriasis-associated fatty acid-binding protein homolog) (PA-FABP)** | Q01469 | FABP5 | -1.86 | <0.001 | 0.020 | NO |
| **MHC class I antigen** | A0A024F8H3 | HLA-B | -1.86 | <0.001 | <0.001 | NO |
| **Fatty acid-binding protein, heart (Fatty acid-binding protein 3) (Heart-type fatty acid-binding protein) (H-FABP) (Mammary-derived growth inhibitor) (MDGI) (Muscle fatty acid-binding protein) (M-FABP)** | P05413 | FABP3 | -1.86 | <0.001 | 0.019 | NO |
| **Elongator complex protein 5 (Dermal papilla-derived protein 6) (S-phase 2 protein)** | Q8TE02 | ELP5 | -1.84 | <0.001 | 0.018 | NO |
| **Carbonic anhydrase 12 (EC 4.2.1.1) (Carbonate dehydratase XII) (Carbonic anhydrase XII) (CA-XII) (Tumor antigen HOM-RCC-3.1.3)** | O43570 | CA12 | -1.83 | <0.001 | 0.013 | NO |
| **Tubulin polymerization-promoting protein family member 3 (TPPP/p20)** | Q9BW30 | TPPP3 | -1.83 | <0.001 | 0.025 | NO |
| **E3 ubiquitin-protein ligase TRIM32 (EC 2.3.2.27) (72 kDa Tat-interacting protein) (RING-type E3 ubiquitin transferase TRIM32) (Tripartite motif-containing protein 32) (Zinc finger protein HT2A)** | Q13049 | TRIM32 | -1.82 | 0.001 | 0.033 | NO |
| **11-beta-hydroxysteroid dehydrogenase 1 (11-DH) (11-beta-HSD1) (EC 1.1.1.146) (7-oxosteroid reductase) (EC 1.1.1.201) (Corticosteroid 11-beta-dehydrogenase isozyme 1) (Short chain dehydrogenase/reductase family 26C member 1)** | P28845 | HSD11B1 | -1.80 | <0.001 | 0.019 | NO |
| **Erythrocyte membrane protein band 4.1 like 3 (cDNA FLJ77757)** | A8K968 | EPB41L3 | -1.79 | <0.001 | 0.013 | NO |
| **G-protein coupled receptor 161 (G-protein coupled receptor RE2)** | Q8N6U8 | GPR161 | -1.76 | 0.002 | 0.036 | YES |
| **NAD(P)H dehydrogenase [quinone] 1 (EC 1.6.5.2) (Azoreductase) (DT-diaphorase) (DTD) (Menadione reductase) (NAD(P)H:quinone oxidoreductase 1) (Phylloquinone reductase) (Quinone reductase 1) (QR1)** | P15559 | NQO1 | -1.75 | <0.001 | 0.006 | NO |
| **Sulfotransferase** | A0A024QZB4 | hCG_1993905 | -1.70 | <0.001 | 0.021 | NO |
| **Actin-binding LIM protein 3 (abLIM-3) (Actin-binding LIM protein family member 3)** | O94929 | ABLIM3 | -1.70 | <0.001 | <0.001 | YES |
| **Aminopeptidase B (AP-B) (EC 3.4.11.6) (Arginine aminopeptidase) (Arginyl aminopeptidase)** | Q9H4A4 | RNPEP | -1.65 | <0.001 | <0.001 | NO |
| **Mitochondrial enolase superfamily member 1 (EC 4.2.1.68) (Antisense RNA to thymidylate synthase) (rTS) (L-fuconate dehydratase)** | Q7L5Y1 | ENOSF1 | -1.61 | <0.001 | 0.001 | NO |
| **Spectrin alpha chain, erythrocytic 1 (Erythroid alpha-spectrin)** | P02549 | SPTA1 | -1.60 | 0.003 | 0.048 | NO |
| **Peptidyl-prolyl cis-trans isomerase-like 3 (PPIase) (EC 5.2.1.8) (Cyclophilin J) (CyPJ) (Cyclophilin-like protein PPIL3) (Rotamase PPIL3)** | Q9H2H8 | PPIL3 | -1.60 | <0.001 | 0.002 | NO |
| **Arginase-1 (EC 3.5.3.1) (Liver-type arginase) (Type I arginase)** | P05089 | ARG1 | -1.57 | 0.002 | 0.036 | NO |
| **cDNA FLJ55560, highly similar to Retinal dehydrogenase 2** | B4DH89 | NA | -1.55 | <0.001 | 0.008 | NO |
| **Insulin-like growth factor-binding protein complex acid labile subunit (ALS)** | P35858 | IGFALS | -1.55 | 0.002 | 0.035 | NO |
| **Katanin p80 WD40 repeat-containing subunit B1 (Katanin p80 subunit B1) (p80 katanin)** | Q9BVA0 | KATNB1 | -1.55 | <0.001 | 0.024 | NO |
| **Insulin-like growth factor-binding protein 5 (IBP-5) (IGF-binding protein 5) (IGFBP-5)** | P24593 | IGFBP5 | -1.55 | <0.001 | 0.006 | YES |
| **Ankyrin-1 (ANK-1) (Ankyrin-R) (Erythrocyte ankyrin)** | P16157 | ANK1 | -1.52 | 0.003 | 0.047 | YES |
| **Alpha-N-acetylglucosaminidase (EC 3.2.1.50) (N-acetyl-alpha-glucosaminidase) (NAG) [Cleaved into: Alpha-N-acetylglucosaminidase 82 kDa form; Alpha-N-acetylglucosaminidase 77 kDa form]** | P54802 | NAGLU | -1.52 | <0.001 | 0.001 | NO |
| **Aldehyde oxidase (EC 1.2.3.1) (Aldehyde oxidase 1) (Azaheterocycle hydroxylase) (EC 1.17.3.-)** | Q06278 | AOX1 | -1.51 | 0.001 | 0.031 | NO |
| **Ribosyldihydronicotinamide dehydrogenase [quinone] (EC 1.10.5.1) (NRH dehydrogenase [quinone] 2) (NRH:quinone oxidoreductase 2) (Quinone reductase 2) (QR2)** | P16083 | NQO2 | -1.51 | <0.001 | 0.021 | NO |
| **Immunoglobulin heavy variable 4-39 (Ig heavy chain V-II region WAH)** | P01824 | IGHV4-39 | -1.50 | 0.001 | 0.031 | NO |
| **Caspase-6 (CASP-6) (CSP-6) (EC 3.4.22.59) (Apoptotic protease Mch-2) [Cleaved into: Caspase-6 subunit p18 (Caspase-6 subunit p20); Caspase-6 subunit p11 (Caspase-6 subunit p10)]** | P55212 | CASP6 | -1.50 | <0.001 | 0.010 | NO |
| **Beta-adducin (Erythrocyte adducin subunit beta)** | P35612 | ADD2 | -1.47 | 0.001 | 0.031 | NO |
| **Transforming growth factor beta receptor type 3 (TGF-beta receptor type 3) (TGFR-3) (Betaglycan) (Transforming growth factor beta receptor III) (TGF-beta receptor type III)** | Q03167 | TGFBR3 | -1.47 | <0.001 | 0.007 | NO |
| **Beta-2-microglobulin [Cleaved into: Beta-2-microglobulin form pI 5.3]** | P61769 | B2M | -1.44 | <0.001 | 0.018 | NO |
| **Ribose-phosphate pyrophosphokinase 2 (EC 2.7.6.1) (PPRibP) (Phosphoribosyl pyrophosphate synthase II) (PRS-II)** | P11908 | PRPS2 | -1.44 | <0.001 | 0.001 | NO |
| **NA** | P58107 | NA | -1.43 | <0.001 | 0.002 | NO |
| **cGMP-dependent 3',5'-cyclic phosphodiesterase (EC 3.1.4.17) (Cyclic GMP-stimulated phosphodiesterase) (CGS-PDE) (cGSPDE)** | O00408 | PDE2A | -1.43 | 0.003 | 0.044 | NO |
| **Aspartoacylase (EC 3.5.1.15) (Aminoacylase-2) (ACY-2)** | P45381 | ASPA | -1.43 | 0.002 | 0.040 | NO |
| **Protein 4.2 (P4.2) (Erythrocyte membrane protein band 4.2) (Erythrocyte protein 4.2)** | P16452 | EPB42 | -1.39 | 0.003 | 0.047 | NO |
| **MHC class I antigen** | F6IQY8 | HLA-A | -1.39 | <0.001 | 0.019 | NO |
| **Inactive phospholipase C-like protein 1 (PLC-L1) (Phospholipase C-deleted in lung carcinoma) (Phospholipase C-related but catalytically inactive protein) (PRIP)** | Q15111 | PLCL1 | -1.39 | <0.001 | 0.004 | YES |
| **Poly(rC)-binding protein 3 (Alpha-CP3) (PCBP3-overlapping transcript) (PCBP3-overlapping transcript 1)** | P57721 | PCBP3 | -1.36 | <0.001 | 0.011 | NO |
| **Calcitonin gene-related peptide type 1 receptor (CGRP type 1 receptor) (Calcitonin receptor-like receptor) (CRLR)** | Q16602 | CALCRL | -1.36 | 0.002 | 0.038 | NO |
| **Tubulin polymerization-promoting protein (TPPP) (EC 3.6.5.-) (25 kDa brain-specific protein) (TPPP/p25) (p24) (p25-alpha)** | O94811 | TPPP | -1.34 | <0.001 | 0.012 | NO |
| **Methanethiol oxidase (MTO) (EC 1.8.3.4) (56 kDa selenium-binding protein) (SBP56) (SP56) (Selenium-binding protein 1)** | Q13228 | SELENBP1 | -1.31 | <0.001 | 0.018 | NO |
| **6-phosphogluconate dehydrogenase, decarboxylating (EC 1.1.1.44)** | B4DL86 | NA | -1.30 | <0.001 | 0.014 | NO |
| **Forkhead box protein O1 (Forkhead box protein O1A) (Forkhead in rhabdomyosarcoma)** | Q12778 | FOXO1 | -1.29 | <0.001 | 0.001 | YES |
| **Oxidoreductase HTATIP2 (EC 1.1.1.-) (30 kDa HIV-1 TAT-interacting protein) (HIV-1 TAT-interactive protein 2)** | Q9BUP3 | HTATIP2 | -1.28 | <0.001 | 0.002 | NO |
| **Pseudouridylate synthase TRUB1 (EC 5.4.99.-) (TruB pseudouridine synthase homolog 1) (tRNA pseudouridine 55 synthase TRUB1) (Psi55 synthase TRUB1) (EC 5.4.99.25)** | Q8WWH5 | TRUB1 | -1.28 | 0.001 | 0.029 | NO |
| **Calpastatin** | D6RBR1 | CAST | -1.28 | <0.001 | 0.006 | NO |
| **Angiotensin-converting enzyme (ACE) (EC 3.4.15.1) (Dipeptidyl carboxypeptidase I) (Kininase II) (CD antigen CD143) [Cleaved into: Angiotensin-converting enzyme, soluble form]** | P12821 | ACE | -1.27 | <0.001 | 0.011 | NO |
| **A-kinase anchor protein 12 (AKAP-12) (A-kinase anchor protein 250 kDa) (AKAP 250) (Gravin) (Myasthenia gravis autoantigen)** | Q02952 | AKAP12 | -1.25 | <0.001 | 0.001 | YES |
| **Cdc42 effector protein 4 (Binder of Rho GTPases 4)** | Q9H3Q1 | CDC42EP4 | -1.25 | 0.002 | 0.038 | YES |
| **Glutamine synthetase (GS) (EC 6.3.1.2) (Glutamate--ammonia ligase) (Palmitoyltransferase GLUL) (EC 2.3.1.225)** | P15104 | GLUL | -1.23 | 0.001 | 0.032 | NO |
| **Erythrocyte membrane protein band 4.1 like 3** | A0A0A0MRA8 | EPB41L3 | -1.22 | <0.001 | 0.001 | YES |
| **Immunoglobulin heavy constant mu (Ig mu chain C region) (Ig mu chain C region BOT) (Ig mu chain C region GAL) (Ig mu chain C region OU)** | P01871 | IGHM | -1.22 | 0.001 | 0.026 | NO |
| **PDZ and LIM domain protein 1 (C-terminal LIM domain protein 1) (Elfin) (LIM domain protein CLP-36)** | O00151 | PDLIM1 | -1.21 | <0.001 | 0.015 | NO |
| **Endosialin (Tumor endothelial marker 1) (CD antigen CD248)** | Q9HCU0 | CD248 | -1.21 | <0.001 | 0.002 | YES |
| **Ras GTPase-activating-like protein IQGAP2** | Q13576 | IQGAP2 | -1.20 | <0.001 | 0.008 | YES |
| **Sodium/hydrogen exchanger 9 (Na(+)/H(+) exchanger 9) (NHE-9) (Solute carrier family 9 member 9)** | Q8IVB4 | SLC9A9 | -1.17 | <0.001 | 0.026 | NO |
| **Proteasome assembly chaperone 2** | Q9P1R6 | NA | -1.16 | <0.001 | 0.025 | NO |
| **Atrial natriuretic peptide-converting enzyme (EC 3.4.21.-) (Corin) (Heart-specific serine proteinase ATC2) (Pro-ANP-converting enzyme) (Transmembrane protease serine 10) [Cleaved into: Atrial natriuretic peptide-converting enzyme, N-terminal propeptide; Atrial natriuretic peptide-converting enzyme, activated protease fragment; Atrial natriuretic peptide-converting enzyme, 180 kDa soluble fragment; Atrial natriuretic peptide-converting enzyme, 160 kDa soluble fragment; Atrial natriuretic peptide-converting enzyme, 100 kDa soluble fragment]** | Q9Y5Q5 | CORIN | -1.16 | <0.001 | 0.020 | NO |
| **Solute carrier organic anion transporter family member 2A1 (SLCO2A1) (OATP2A1) (PHOAR2) (Prostaglandin transporter) (PGT) (Solute carrier family 21 member 2) (SLC21A2)** | Q92959 | SLCO2A1 | -1.15 | <0.001 | 0.006 | NO |
| **Adipose-secreted signaling protein** | Q9GZN8 | ADISSP | -1.15 | 0.002 | 0.039 | NO |
| **Galactose mutarotase (EC 5.1.3.3) (Aldose 1-epimerase)** | Q96C23 | GALM | -1.14 | <0.001 | 0.010 | NO |
| **Leukocyte elastase inhibitor (LEI) (Monocyte/neutrophil elastase inhibitor) (EI) (M/NEI) (Peptidase inhibitor 2) (PI-2) (Serpin B1)** | P30740 | SERPINB1 | -1.13 | 0.003 | 0.044 | NO |
| **Protein S100-A4 (Calvasculin) (Metastasin) (Placental calcium-binding protein) (Protein Mts1) (S100 calcium-binding protein A4)** | P26447 | S100A4 | -1.13 | <0.001 | 0.018 | NO |
| **MHC class I antigen** | Q546C9 | HLA-B | -1.13 | 0.002 | 0.040 | YES |
| **N-myc-interactor (Nmi) (N-myc and STAT interactor)** | Q13287 | NMI | -1.13 | <0.001 | <0.001 | NO |
| **Nicotinate phosphoribosyltransferase (NAPRTase) (EC 6.3.4.21) (FHA-HIT-interacting protein) (Nicotinate phosphoribosyltransferase domain-containing protein 1)** | Q6XQN6 | NAPRT | -1.11 | <0.001 | 0.012 | YES |
| **Lymphatic vessel endothelial hyaluronic acid receptor 1 (LYVE-1) (Cell surface retention sequence-binding protein 1) (CRSBP-1) (Extracellular link domain-containing protein 1) (Hyaluronic acid receptor)** | Q9Y5Y7 | LYVE1 | -1.08 | 0.001 | 0.028 | NO |
| **Retinal dehydrogenase 2 (RALDH 2) (RalDH2) (EC 1.2.1.36) (Aldehyde dehydrogenase family 1 member A2) (ALDH1A2) (Retinaldehyde-specific dehydrogenase type 2) (RALDH(II))** | O94788 | ALDH1A2 | -1.07 | <0.001 | 0.003 | NO |
| **Activating signal cointegrator 1 complex subunit 2 (ASC-1 complex subunit p100) (Trip4 complex subunit p100)** | Q9H1I8 | ASCC2 | -1.07 | <0.001 | 0.013 | NO |
| **F-box only protein 7** | Q9Y3I1 | FBXO7 | -1.06 | 0.002 | 0.033 | NO |
| **Putative monooxygenase p33MONOX (EC 1.-.-.-) (Brain-derived rescue factor p60MONOX) (Flavin monooxygenase motif-containing protein of 33 kDa)** | Q96A73 | KIAA1191 | -1.06 | <0.001 | 0.015 | NO |
| **Serine hydroxymethyltransferase, cytosolic (SHMT) (EC 2.1.2.1) (Glycine hydroxymethyltransferase) (Serine methylase)** | P34896 | SHMT1 | -1.05 | <0.001 | 0.009 | NO |
| **Macrophage mannose receptor 1 (MMR) (C-type lectin domain family 13 member D) (C-type lectin domain family 13 member D-like) (Human mannose receptor) (hMR) (Macrophage mannose receptor 1-like protein 1) (CD antigen CD206)** | P22897 | MRC1 | -1.05 | <0.001 | 0.025 | NO |
| **Sialoadhesin (Sialic acid-binding Ig-like lectin 1) (Siglec-1) (CD antigen CD169)** | Q9BZZ2 | SIGLEC1 | -1.05 | <0.001 | 0.019 | NO |
| **Inositol 1,3,4-triphosphate 5/6 kinase, isoform CRA_a** | A0A024R6H3 | ITPK1 | -1.05 | 0.001 | 0.032 | NO |
| **Alpha-crystallin B chain (Alpha(B)-crystallin) (Heat shock protein beta-5) (HspB5) (Heat shock protein family B member 5) (Renal carcinoma antigen NY-REN-27) (Rosenthal fiber component)** | P02511 | CRYAB | -1.04 | <0.001 | 0.026 | YES |
| **Phosphatase and actin regulator 2** | O75167 | PHACTR2 | -1.03 | 0.002 | 0.033 | NO |
| **Pirin (EC 1.13.11.24) (Probable quercetin 2,3-dioxygenase PIR) (Probable quercetinase)** | O00625 | PIR | -1.03 | <0.001 | 0.007 | NO |
| **Inactive C-alpha-formylglycine-generating enzyme 2 (Paralog of formylglycine-generating enzyme) (pFGE) (Sulfatase-modifying factor 2)** | Q8NBJ7 | SUMF2 | -1.03 | 0.001 | 0.029 | NO |
| **Protein TANC1 (Tetratricopeptide repeat, ankyrin repeat and coiled-coil domain-containing protein 1)** | Q9C0D5 | TANC1 | -1.03 | <0.001 | 0.006 | NO |
| **IgG receptor FcRn large subunit p51 (FcRn) (IgG Fc fragment receptor transporter alpha chain) (Neonatal Fc receptor)** | P55899 | FCGRT | -1.02 | 0.002 | 0.036 | NO |
| **Tumor necrosis factor alpha-induced protein 8 (TNF alpha-induced protein 8) (Head and neck tumor and metastasis-related protein) (MDC-3.13) (NF-kappa-B-inducible DED-containing protein) (NDED) (SCC-S2) (TNF-induced protein GG2-1)** | O95379 | TNFAIP8 | -1.02 | 0.001 | 0.026 | NO |
| **Tumor necrosis factor alpha-induced protein 2 (TNF alpha-induced protein 2) (Primary response gene B94 protein)** | Q03169 | TNFAIP2 | -1.02 | 0.002 | 0.034 | YES |
| **Fructose-bisphosphate aldolase B (EC 4.1.2.13) (Liver-type aldolase)** | P05062 | ALDOB | -1.01 | 0.003 | 0.044 | NO |
| **NmrA-like family domain-containing protein 1** | Q9HBL8 | NMRAL1 | -1.01 | <0.001 | 0.020 | NO |
| **Biogenesis of lysosome-related organelles complex 1 subunit 3 (BLOC-1 subunit 3)** | Q6QNY0 | BLOC1S3 | -1.00 | <0.001 | 0.023 | YES |
|  |  |  |  |  |  |  |

**Supplementary Information 12:** Total proteins that passed FDR (q≤0.05) and significantly (p≤0.05) at least doubled in the failed IOL group compared with the elCS group for at least two of the experimental conditions**.** PRE = pre-contracting; SP-REL = relaxed-phase during spontaneous contractions; OXT-REL = relaxed-phase during oxytocin-induced contractions; SP-CON = contracted-phase during spontaneous contractions; OXT-CON = contracted-phase during oxytocin-induced contractions.

|  |  |  |  |  |  |  |  |
| --- | --- | --- | --- | --- | --- | --- | --- |
| **Protein name** | **Accession** | **Gene name** | **PRE** | **SP-REL** | **OXT-REL** | **SP-CON** | **OXT-CON** |
| **Annexin A3 (35-alpha calcimedin) (Annexin III) (Annexin-3) (Inositol 1,2-cyclic phosphate 2-phosphohydrolase) (Lipocortin III) (Placental anticoagulant protein III) (PAP-III)** | P12429 | ANXA3 | ✓ | ✓ | ✓ | ✓ | ✓ |
| **C14orf159 variant protein** | Q4LE40 | C14orf159 variant protein | ✓ | ✓ | ✓ | ✓ | ✓ |
| **Cartilage oligomeric matrix protein (COMP) (Thrombospondin-5) (TSP5)** | P49747 | COMP | ✓ | ✓ | ✓ | ✓ | ✓ |
| **cDNA FLJ51896, highly similar to Glia-derived nexin** | B4DMR3 | NA | ✓ | ✓ | ✓ | ✓ | ✓ |
| **Collagen alpha-1(XII) chain** | Q99715 | COL12A1 | ✓ | ✓ | ✓ | ✓ | ✓ |
| **Desmuslin, isoform CRA_a (Synemin)** | A0A075B7B1 | SYNM | ✓ | ✓ | ✓ | ✓ | ✓ |
| **Immunoglobulin heavy constant gamma 4 (Ig gamma-4 chain C region)** | P01861 | IGHG4 | ✓ | ✓ | ✓ | ✓ | ✓ |
| **Lysyl oxidase homolog 2 (EC 1.4.3.13) (Lysyl oxidase-like protein 2) (Lysyl oxidase-related protein 2) (Lysyl oxidase-related protein WS9-14)** | Q9Y4K0 | LOXL2 | ✓ | ✓ | ✓ | ✓ | ✓ |
| **maleylacetoacetate isomerase (EC 5.2.1.2)** | G3V4T6 | GSTZ1 | ✓ | ✓ | ✓ | ✓ | ✓ |
| **Matrilin-2** | O00339 | MATN2 | ✓ | ✓ | ✓ | ✓ | ✓ |
| **Matrix Gla protein (MGP) (Cell growth-inhibiting gene 36 protein)** | P08493 | MGP | ✓ | ✓ | ✓ | ✓ | ✓ |
| **N90-VRC38.08 heavy chain variable region** | A0A1W6IYI5 | NA | ✓ | ✓ | ✓ | ✓ | ✓ |
| **Polypeptide N-acetylgalactosaminyltransferase 18 (EC 2.4.1.41) (Polypeptide GalNAc transferase 18) (GalNAc-T18) (Polypeptide GalNAc transferase-like protein 4) (GalNAc-T-like protein 4) (pp-GaNTase-like protein 4) (Polypeptide N-acetylgalactosaminyltransferase-like protein 4) (Protein-UDP acetylgalactosaminyltransferase-like protein 4) (UDP-GalNAc:polypeptide N-acetylgalactosaminyltransferase-like protein 4)** | Q6P9A2 | GALNT18 | ✓ | ✓ | ✓ | ✓ | ✓ |
| **Procollagen-lysine,2-oxoglutarate 5-dioxygenase 2 (EC 1.14.11.4) (Lysyl hydroxylase 2) (LH2)** | O00469 | PLOD2 | ✓ | ✓ | ✓ | ✓ | ✓ |
| **Prostacyclin synthase (EC 5.3.99.4) (Hydroperoxy icosatetraenoate dehydratase) (EC 4.2.1.152) (Prostaglandin I2 synthase)** | Q16647 | PTGIS | ✓ | ✓ | ✓ | ✓ | ✓ |
| **Prostaglandin G/H synthase 1 (EC 1.14.99.1) (Cyclooxygenase-1) (COX-1) (Prostaglandin H2 synthase 1) (PGH synthase 1) (PGHS-1) (PHS 1) (Prostaglandin-endoperoxide synthase 1)** | P23219 | PTGS1 | ✓ | ✓ | ✓ | ✓ | ✓ |
| **SAA2-SAA4 readthrough** | A0A096LPE2 | SAA2-SAA4 | ✓ | ✓ | ✓ | ✓ | ✓ |
| **SH3 domain-binding glutamic acid-rich-like protein 2 (Fovea-associated SH3 domain-binding protein)** | Q9UJC5 | SH3BGRL2 | ✓ | ✓ | ✓ | ✓ | ✓ |
| **Tenascin-X (TN-X) (Hexabrachion-like protein)** | P22105 | TNXB | ✓ | ✓ | ✓ | ✓ | ✓ |
| **Thrombospondin-4** | P35443 | THBS4 | ✓ | ✓ | ✓ | ✓ | ✓ |
| **TNC variant protein** | Q4LE33 | TNC variant protein | ✓ | ✓ | ✓ | ✓ | ✓ |
| **Transforming growth factor-beta-induced protein ig-h3 (Beta ig-h3) (Kerato-epithelin) (RGD-containing collagen-associated protein) (RGD-CAP)** | Q15582 | TGFBI | ✓ | ✓ | ✓ | ✓ | ✓ |
| **Vitamin D binding protein** | A0A1B1CYC5 | Gc | ✓ | ✓ | ✓ | ✓ | ✓ |
| **Apolipoprotein(a) (Apo(a)) (Lp(a)) (EC 3.4.21.-)** | P08519 | LPA | ✓ | ✓ | ✓ | ✓ |  |
| **Carbonic anhydrase III, muscle specific, isoform CRA_a** | A0A024R825 | CA3 | ✓ | ✓ | ✓ | ✓ |  |
| **Haptoglobin (Zonulin) [Cleaved into: Haptoglobin alpha chain; Haptoglobin beta chain]** | P00738 | HP | ✓ | ✓ | ✓ | ✓ |  |
| **IGH@ protein** | Q6P089 | IGH@ | ✓ | ✓ | ✓ | ✓ |  |
| **MHC class II antigen** | A0A1Y6MRR4 | HLA-DPB1 | ✓ | ✓ | ✓ | ✓ |  |
| **Myosin-reactive immunoglobulin heavy chain variable region** | Q9UL90 | NA | ✓ | ✓ | ✓ | ✓ |  |
| **Choline transporter-like protein** | A0A088QCU6 | SLC44A2 | ✓ | ✓ | ✓ |  | ✓ |
| **Uncharacterized protein DKFZp686O1553** | Q5HYM1 | DKFZp686O1553 | ✓ | ✓ | ✓ |  | ✓ |
| **Pregnancy zone protein (C3 and PZP-like alpha-2-macroglobulin domain-containing protein 6)** | P20742 | PZP | ✓ | ✓ | ✓ |  |  |
| **Thrombospondin 1, isoform CRA_a** | A0A024R9Q1 | THBS1 | ✓ | ✓ | ✓ |  |  |
| **Thrombospondin-1 (Glycoprotein G)** | P07996 | THBS1 | ✓ | ✓ | ✓ |  |  |
| **Serpin peptidase inhibitor, clade E (Nexin, plasminogen activator inhibitor type 1), member 1, isoform CRA_b** | A0A024QYT5 | SERPINE1 | ✓ | ✓ |  | ✓ | ✓ |
| **Alkaline phosphatase, tissue-nonspecific isozyme (AP-TNAP) (TNS-ALP) (TNSALP) (EC 3.1.3.1) (Alkaline phosphatase liver/bone/kidney isozyme) (Phosphoamidase) (Phosphocreatine phosphatase) (EC 3.9.1.1)** | P05186 | ALPL | ✓ | ✓ |  | ✓ |  |
| **Ankyrin-3 (ANK-3) (Ankyrin-G)** | Q12955 | ANK3 | ✓ | ✓ |  | ✓ |  |
| **Calpain-2 catalytic subunit (EC 3.4.22.53) (Calcium-activated neutral proteinase 2) (Calpain M-type) (Calpain-2 large subunit) (Millimolar-calpain)** | B2RCM3 | NA | ✓ | ✓ |  | ✓ |  |
| **CCN family member 4 (WNT1-inducible-signaling pathway protein 1) (WISP-1) (Wnt-1-induced secreted protein)** | O95388 | CCN4 | ✓ | ✓ |  | ✓ |  |
| **Large ribosomal subunit protein eL14 (60S ribosomal protein L14)** | Q6IPH7 | RPL14 | ✓ | ✓ |  | ✓ |  |
| **Matrilin 2, isoform CRA_b** | A0A024R9B9 | MATN2 | ✓ | ✓ |  | ✓ |  |
| **Neural cell adhesion molecule 1** | A0A087WTF6 | NCAM1 | ✓ | ✓ |  | ✓ |  |
| **Secreted frizzled-related protein 1 (FRP-1) (sFRP-1) (Secreted apoptosis-related protein 2) (SARP-2)** | Q8N474 | SFRP1 | ✓ | ✓ |  | ✓ |  |
| **Solute carrier family 2, facilitated glucose transporter member 4 (Glucose transporter type 4, insulin-responsive) (GLUT-4)** | P14672 | SLC2A4 | ✓ | ✓ |  | ✓ |  |
| **Unconventional myosin-If (Myosin-Ie)** | O00160 | MYO1F | ✓ | ✓ |  | ✓ |  |
| **FXYD domain containing ion transport regulator 6, isoform CRA_a** | A0A024R3J8 | FXYD6 | ✓ | ✓ |  |  | ✓ |
| **Lipopolysaccharide-binding protein (LBP)** | P18428 | LBP | ✓ | ✓ |  |  |  |
| **Protein bicaudal D homolog 1 (Bic-D 1)** | Q96G01 | BICD1 | ✓ | ✓ |  |  |  |
| **Diphosphoinositol polyphosphate phosphohydrolase 3-alpha (DIPP-3-alpha) (DIPP3-alpha) (hDIPP3alpha) (EC 3.6.1.52) (Diadenosine 5',5'''-P1,P6-hexaphosphate hydrolase 3-alpha) (Diadenosine hexaphosphate hydrolase (AMP-forming)) (EC 3.6.1.60) (Nucleoside diphosphate-linked moiety X motif 10) (Nudix motif 10) (hAps2)** | Q8NFP7 | NUDT10 | ✓ |  | ✓ | ✓ |  |
| **Uncharacterized protein DKFZp686K18196** | Q6N092 | DKFZp686K18196 | ✓ |  | ✓ | ✓ |  |
| **DNA repair protein XRCC1 (X-ray repair cross-complementing protein 1)** | P18887 | XRCC1 | ✓ |  | ✓ |  | ✓ |
| **Keratin, type I cytoskeletal 9 (Cytokeratin-9) (CK-9) (Keratin-9) (K9)** | P35527 | KRT9 | ✓ |  | ✓ |  | ✓ |
| **Keratin, type II cytoskeletal 1 (Cytokeratin-1) (Keratin-1) (Type-II keratin Kb1)** | H6VRG2 | KRT1 | ✓ |  | ✓ |  | ✓ |
| **Keratin, type II cytoskeletal 5 (Cytokeratin-5) (CK-5) (Keratin-5) (K5) (Type-II keratin Kb5)** | Q5XQN5 | KRT5 | ✓ |  | ✓ |  | ✓ |
| **Zinc finger, UBR1 type 1, isoform CRA_c** | A0A024RAC9 | ZUBR1 | ✓ |  | ✓ |  | ✓ |
| **Methylthioribulose-1-phosphate dehydratase (MTRu-1-P dehydratase) (EC 4.2.1.109) (APAF1-interacting protein) (hAPIP)** | Q96GX9 | APIP | ✓ |  | ✓ |  |  |
| **Dihydropyrimidinase-related protein 1 (DRP-1) (Collapsin response mediator protein 1) (CRMP-1) (Inactive dihydropyrimidinase) (Unc-33-like phosphoprotein 3) (ULIP-3)** | Q14194 | CRMP1 | ✓ |  |  | ✓ | ✓ |
| **Alpha-2-macroglobulin (Alpha-2-M) (C3 and PZP-like alpha-2-macroglobulin domain-containing protein 5)** | P01023 | A2M | ✓ |  |  | ✓ |  |
| **Parvalbumin** | H0Y3U0 | PVALB | ✓ |  |  | ✓ |  |
| **[Pyruvate dehydrogenase (acetyl-transferring)] kinase isozyme 1, mitochondrial (EC 2.7.11.2) (Pyruvate dehydrogenase kinase isoform 1) (PDH kinase 1)** | Q15118 | PDK1 |  | ✓ | ✓ | ✓ | ✓ |
| **Adipocyte enhancer-binding protein 1 (AE-binding protein 1) (Aortic carboxypeptidase-like protein)** | Q8IUX7 | AEBP1 |  | ✓ | ✓ | ✓ | ✓ |
| **Asporin (Periodontal ligament-associated protein 1) (PLAP-1)** | Q9BXN1 | ASPN |  | ✓ | ✓ | ✓ | ✓ |
| **Biglycan (Bone/cartilage proteoglycan I) (PG-S1)** | P21810 | BGN |  | ✓ | ✓ | ✓ | ✓ |
| **Cadherin 2, type 1, N-cadherin (Neuronal), isoform CRA_b** | A0A024RC42 | CDH2 |  | ✓ | ✓ | ✓ | ✓ |
| **Collagen alpha-1(XV) chain [Cleaved into: Restin (Endostatin-XV) (Related to endostatin) (Restin-I); Restin-2 (Restin-II); Restin-3 (Restin-III); Restin-4 (Restin-IV)]** | P39059 | COL15A1 |  | ✓ | ✓ | ✓ | ✓ |
| **Corrinoid adenosyltransferase MMAB (EC 2.5.1.-) (ATP:co(I)rrinoid adenosyltransferase MMAB) (Methylmalonic aciduria type B protein)** | Q96EY8 | MMAB |  | ✓ | ✓ | ✓ | ✓ |
| **D-3-phosphoglycerate dehydrogenase (3-PGDH) (EC 1.1.1.95) (2-oxoglutarate reductase) (EC 1.1.1.399) (Malate dehydrogenase) (EC 1.1.1.37)** | O43175 | PHGDH |  | ✓ | ✓ | ✓ | ✓ |
| **Fibrillin-2 [Cleaved into: Placensin]** | P35556 | FBN2 |  | ✓ | ✓ | ✓ | ✓ |
| **glutaminase (EC 3.5.1.2)** | B3KM58 | NA |  | ✓ | ✓ | ✓ | ✓ |
| **Inter-alpha-trypsin inhibitor heavy chain H1 (ITI heavy chain H1) (ITI-HC1) (Inter-alpha-inhibitor heavy chain 1) (Inter-alpha-trypsin inhibitor complex component III) (Serum-derived hyaluronan-associated protein) (SHAP)** | P19827 | ITIH1 |  | ✓ | ✓ | ✓ | ✓ |
| **Latent-transforming growth factor beta-binding protein 2 (LTBP-2)** | Q14767 | LTBP2 |  | ✓ | ✓ | ✓ | ✓ |
| **Matrix metalloproteinase-19 (MMP-19) (EC 3.4.24.-) (Matrix metalloproteinase RASI) (Matrix metalloproteinase-18) (MMP-18)** | Q99542 | MMP19 |  | ✓ | ✓ | ✓ | ✓ |
| **Monocarboxylate transporter 2 (MCT 2) (Solute carrier family 16 member 7)** | O60669 | SLC16A7 |  | ✓ | ✓ | ✓ | ✓ |
| **NA** | Q15063 | NA |  | ✓ | ✓ | ✓ | ✓ |
| **PG-M** | Q6MZK8 | DKFZp686K06110 |  | ✓ | ✓ | ✓ | ✓ |
| **Ribonuclease T2 (EC 4.6.1.19) (Ribonuclease 6)** | O00584 | RNASET2 |  | ✓ | ✓ | ✓ | ✓ |
| **Septin-6** | Q14141 | SEPTIN6 |  | ✓ | ✓ | ✓ | ✓ |
| **Zinc finger FYVE domain-containing protein 26 (FYVE domain-containing centrosomal protein) (FYVE-CENT) (Spastizin)** | Q68DK2 | ZFYVE26 |  | ✓ | ✓ | ✓ | ✓ |
| **Zinc transporter 7 (ZnT-7) (Solute carrier family 30 member 7) (Znt-like transporter 2)** | Q8NEW0 | SLC30A7 |  | ✓ | ✓ | ✓ | ✓ |
| **CCN family member 2 (Cellular communication network factor 2) (Connective tissue growth factor)** | Q5M8T4 | CTGF |  | ✓ | ✓ | ✓ |  |
| **Cytoplasmic dynein 2 heavy chain 1 (Cytoplasmic dynein 2 heavy chain) (Dynein cytoplasmic heavy chain 2) (Dynein heavy chain 11) (hDHC11) (Dynein heavy chain isotype 1B)** | Q8NCM8 | DYNC2H1 |  | ✓ | ✓ | ✓ |  |
| **Fibroleukin (Fibrinogen-like protein 2) (pT49)** | Q14314 | FGL2 |  | ✓ | ✓ | ✓ |  |
| **Metalloproteinase inhibitor 1 (Erythroid-potentiating activity) (EPA) (Fibroblast collagenase inhibitor) (Collagenase inhibitor) (Tissue inhibitor of metalloproteinases 1) (TIMP-1)** | P01033 | TIMP1 |  | ✓ | ✓ | ✓ |  |
| **Midkine (MK) (Amphiregulin-associated protein) (ARAP) (Midgestation and kidney protein) (Neurite outgrowth-promoting factor 2) (Neurite outgrowth-promoting protein)** | P21741 | MDK |  | ✓ | ✓ | ✓ |  |
| **OX-2 membrane glycoprotein (CD antigen CD200)** | P41217 | CD200 |  | ✓ | ✓ | ✓ |  |
| **Protein AMBP (Protein HC) [Cleaved into: Alpha-1-microglobulin (EC 1.6.2.-) (Alpha-1 microglycoprotein) (Complex-forming glycoprotein heterogeneous in charge); Inter-alpha-trypsin inhibitor light chain (ITI-LC) (Bikunin) (EDC1) (HI-30) (Uronic-acid-rich protein); Trypstatin]** | P02760 | AMBP |  | ✓ | ✓ | ✓ |  |
| **Alpha-catulin (Alpha-catenin-related protein) (ACRP) (Catenin alpha-like protein 1)** | Q9UBT7 | CTNNAL1 |  | ✓ | ✓ |  | ✓ |
| **Latent-transforming growth factor beta-binding protein 4 (LTBP-4)** | Q8N2S1 | LTBP4 |  | ✓ | ✓ |  | ✓ |
| **Pentraxin-related protein PTX3 (Pentaxin-related protein PTX3) (Tumor necrosis factor alpha-induced protein 5) (TNF alpha-induced protein 5) (Tumor necrosis factor-inducible gene 14 protein) (TSG-14)** | P26022 | PTX3 |  | ✓ | ✓ |  | ✓ |
| **Synaptophysin-like protein 2** | Q5VXT5 | SYPL2 |  | ✓ | ✓ |  | ✓ |
| **Twisted gastrulation protein homolog 1** | Q9GZX9 | TWSG1 |  | ✓ | ✓ |  | ✓ |
| **ADP/ATP translocase 1 (ADP,ATP carrier protein 1) (ADP,ATP carrier protein, heart/skeletal muscle isoform T1) (Adenine nucleotide translocator 1) (ANT 1) (Solute carrier family 25 member 4)** | P12235 | SLC25A4 |  | ✓ | ✓ |  |  |
| **Alpha-1B-glycoprotein (Alpha-1-B glycoprotein)** | P04217 | A1BG |  | ✓ | ✓ |  |  |
| **Apolipoprotein C-III (Apo-CIII) (ApoC-III) (Apolipoprotein C3)** | P02656 | APOC3 |  | ✓ | ✓ |  |  |
| **CCN family member 1 (Cellular communication network factor 1) (Cysteine-rich angiogenic inducer 61) (Insulin-like growth factor-binding protein 10) (IBP-10) (IGF-binding protein 10) (IGFBP-10) (Protein CYR61) (Protein GIG1)** | O00622 | CCN1 |  | ✓ | ✓ |  |  |
| **Chondroitin sulfate proteoglycan 2 (Versican), isoform CRA_c** | A0A024RAL1 | CSPG2 |  | ✓ | ✓ |  |  |
| **Cysteine-rich motor neuron 1 protein (CRIM-1) (Cysteine-rich repeat-containing protein S52) [Cleaved into: Processed cysteine-rich motor neuron 1 protein]** | Q9NZV1 | CRIM1 |  | ✓ | ✓ |  |  |
| **Extracellular sulfatase Sulf-1 (hSulf-1) (Arylsulfatase) (EC 3.1.6.1) (N-acetylglucosamine-6-sulfatase) (EC 3.1.6.14) [Cleaved into: Extracellular sulfatase Sulf-2 secreted form]** | Q8IWU6 | SULF1 |  | ✓ | ✓ |  |  |
| **Insulin-like growth factor-binding protein 7 (IBP-7) (IGF-binding protein 7) (IGFBP-7) (IGFBP-rP1) (MAC25 protein) (PGI2-stimulating factor) (Prostacyclin-stimulating factor) (Tumor-derived adhesion factor) (TAF)** | Q16270 | IGFBP7 |  | ✓ | ✓ |  |  |
| **Inter-alpha-trypsin inhibitor heavy chain H2 (ITI heavy chain H2) (ITI-HC2) (Inter-alpha-inhibitor heavy chain 2) (Inter-alpha-trypsin inhibitor complex component II) (Serum-derived hyaluronan-associated protein) (SHAP)** | P19823 | ITIH2 |  | ✓ | ✓ |  |  |
| **Laminin subunit alpha-2 (Laminin M chain) (Laminin-12 subunit alpha) (Laminin-2 subunit alpha) (Laminin-4 subunit alpha) (Merosin heavy chain)** | P24043 | LAMA2 |  | ✓ | ✓ |  |  |
| **Microfibril-associated glycoprotein 4** | P55083 | MFAP4 |  | ✓ | ✓ |  |  |
| **Periostin, osteoblast specific factor, isoform CRA_a** | A0A024RDT5 | POSTN |  | ✓ | ✓ |  |  |
| **Tubulointerstitial nephritis antigen-like (Glucocorticoid-inducible protein 5) (Oxidized LDL-responsive gene 2 protein) (OLRG-2) (Tubulointerstitial nephritis antigen-related protein) (TIN Ag-related protein) (TIN-Ag-RP)** | Q9GZM7 | TINAGL1 |  | ✓ | ✓ |  |  |
| **Dual adapter for phosphotyrosine and 3-phosphotyrosine and 3-phosphoinositide (hDAPP1) (B lymphocyte adapter protein Bam32) (B-cell adapter molecule of 32 kDa)** | Q9UN19 | DAPP1 |  | ✓ |  | ✓ | ✓ |
| **Complement factor H (H factor 1)** | P08603 | CFH |  | ✓ |  | ✓ |  |
| **Serine racemase (EC 5.1.1.18) (D-serine ammonia-lyase) (D-serine dehydratase) (EC 4.3.1.18) (L-serine ammonia-lyase) (L-serine dehydratase) (EC 4.3.1.17)** | Q9GZT4 | SRR |  | ✓ |  | ✓ |  |
| **Solute carrier family 16 (Monocarboxylic acid transporters), member 3, isoform CRA_a** | A0A024R8U1 | SLC16A3 |  | ✓ |  | ✓ |  |
| **Thrombospondin-2** | P35442 | THBS2 |  | ✓ |  | ✓ |  |
| **Transforming growth factor beta-2 proprotein (Cetermin) (Glioblastoma-derived T-cell suppressor factor) (G-TSF) [Cleaved into: Latency-associated peptide (LAP); Transforming growth factor beta-2 (TGF-beta-2)]** | P61812 | TGFB2 |  | ✓ |  | ✓ |  |
| **Zinc finger, UBR1 type 1, isoform CRA_c** | A0A024RAC9 | ZUBR1 |  | ✓ |  | ✓ |  |
| **Chromosome 1 open reading frame 24, isoform CRA_a** | A0A024R978 | C1orf24 |  | ✓ |  |  | ✓ |
| **Core histone macro-H2A.1 (Histone macroH2A1) (mH2A1) (Histone H2A.y) (H2A/y) (Medulloblastoma antigen MU-MB-50.205)** | O75367 | MACROH2A1 |  | ✓ |  |  | ✓ |
| **Disheveled-associated activator of morphogenesis 2** | Q86T65 | DAAM2 |  | ✓ |  |  | ✓ |
| **L-amino-acid oxidase (LAAO) (LAO) (EC 1.4.3.2) (EC 1.4.3.25) (Interleukin-4-induced protein 1) (IL4-induced protein 1) (hIL4I1) (Protein Fig-1) (hFIG1)** | Q96RQ9 | IL4I1 |  | ✓ |  |  | ✓ |
| **Latent-transforming growth factor beta-binding protein 1 (LTBP-1) (Transforming growth factor beta-1-binding protein 1) (TGF-beta1-BP-1)** | Q14766 | LTBP1 |  | ✓ |  |  | ✓ |
| **von Willebrand factor A domain-containing protein 1** | Q6PCB0 | VWA1 |  | ✓ |  |  | ✓ |
| **Bifunctional methylenetetrahydrofolate dehydrogenase/cyclohydrolase, mitochondrial [Includes: NAD-dependent methylenetetrahydrofolate dehydrogenase (EC 1.5.1.15); Methenyltetrahydrofolate cyclohydrolase (EC 3.5.4.9)]** | MTHFD2 | P13995 |  |  | ✓ | ✓ | ✓ |
| **Biglycan** | BGN | C9JKG1 |  |  | ✓ | ✓ | ✓ |
| **NADH:ubiquinone oxidoreductase MLRQ subunit homolog, isoform CRA_a** | LOC56901 | A0A024RB39 |  |  | ✓ | ✓ | ✓ |
| **Uncharacterized protein DKFZp686M0562** | DKFZp686M0562 | Q6MZL2 |  |  | ✓ | ✓ |  |
| **Acrosomal protein KIAA1210** | KIAA1210 | Q9ULL0 |  |  | ✓ |  | ✓ |
| **DNA helicase MCM8 (EC 3.6.4.12) (Minichromosome maintenance 8)** | MCM8 | Q9UJA3 |  |  | ✓ |  | ✓ |
| **Glutaminase kidney isoform, mitochondrial (GLS) (EC 3.5.1.2) (K-glutaminase) (L-glutamine amidohydrolase) [Cleaved into: Glutaminase kidney isoform, mitochondrial 68 kDa chain; Glutaminase kidney isoform, mitochondrial 65 kDa chain]** | GLS | O94925 |  |  | ✓ |  | ✓ |
| **Glypican-4 (K-glypican) [Cleaved into: Secreted glypican-4]** | GPC4 | O75487 |  |  | ✓ |  | ✓ |
| **Junctional adhesion molecule B (JAM-B) (Junctional adhesion molecule 2) (JAM-2) (Vascular endothelial junction-associated molecule) (VE-JAM) (CD antigen CD322)** | JAM2 | P57087 |  |  | ✓ |  | ✓ |
| **Keratin, type I cytoskeletal 10 (Cytokeratin-10) (CK-10) (Keratin-10) (K10)** | KRT10 | P13645 |  |  | ✓ |  | ✓ |
| **Liver carboxylesterase 1 (Acyl-coenzyme A:cholesterol acyltransferase) (ACAT) (Brain carboxylesterase hBr1) (Carboxylesterase 1) (CE-1) (hCE-1) (EC 3.1.1.1) (Cholesteryl ester hydrolase) (CEH) (EC 3.1.1.13) (Cocaine carboxylesterase) (Egasyn) (HMSE) (Methylumbelliferyl-acetate deacetylase 1) (EC 3.1.1.56) (Monocyte/macrophage serine esterase) (Retinyl ester hydrolase) (REH) (Serine esterase 1) (Triacylglycerol hydrolase) (TGH)** | CES1 | P23141 |  |  | ✓ |  | ✓ |
| **Mimecan (Osteoglycin) (Osteoinductive factor) (OIF)** | OGN | P20774 |  |  | ✓ |  | ✓ |
| **Neurofilament light polypeptide isoform 2** | NEFL | A0A0S2Z4B1 |  |  | ✓ |  | ✓ |
| **SPARC-related modular calcium-binding protein 1 (Secreted modular calcium-binding protein 1) (SMOC-1)** | SMOC1 | Q9H4F8 |  |  | ✓ |  | ✓ |
| **Thrombospondin-3** | THBS3 | P49746 |  |  | ✓ |  | ✓ |
| **Thrombospondin 1, isoform CRA_a** | A0A024R9Q1 | THBS1 |  |  |  | ✓ | ✓ |
|  |  |  |  |  |  |  |  |

Supplementary Information 13: Total proteins which passed FDR (q≤0.05) and significantly (p≤0.05) at least halved and in the failed IOL group compared to the elCS group for at least two of the conditions. PRE = pre-contracting; SP-REL = relaxed-phase during spontaneous contractions; OXT-REL = relaxed-phase during oxytocin-induced contractions; SP-CON = contracted-phase during spontaneous contractions; OXT-CON = contracted-phase during oxytocin-induced contractions.

|  |  |  |  |  |  |  |  |
| --- | --- | --- | --- | --- | --- | --- | --- |
| **Protein name** | **Accession** | **Gene name** | **PRE** | **SP-REL** | **OXT-REL** | **SP-CON** | **OXT-CON** |
| **A-kinase anchor protein 12 (AKAP-12) (A-kinase anchor protein 250 kDa) (AKAP 250) (Gravin) (Myasthenia gravis autoantigen)** | Q02952 | AKAP12 | ✓ | ✓ | ✓ | ✓ | ✓ |
| **Activating signal cointegrator 1 complex subunit 2 (ASC-1 complex subunit p100) (Trip4 complex subunit p100)** | Q9H1I8 | ASCC2 | ✓ | ✓ | ✓ | ✓ | ✓ |
| **Alpha-N-acetylglucosaminidase (EC 3.2.1.50) (N-acetyl-alpha-glucosaminidase) (NAG) [Cleaved into: Alpha-N-acetylglucosaminidase 82 kDa form; Alpha-N-acetylglucosaminidase 77 kDa form]** | P54802 | NAGLU | ✓ | ✓ | ✓ | ✓ | ✓ |
| **Aminopeptidase B (AP-B) (EC 3.4.11.6) (Arginine aminopeptidase) (Arginyl aminopeptidase)** | Q9H4A4 | RNPEP | ✓ | ✓ | ✓ | ✓ | ✓ |
| **Aminopeptidase N (EC 3.4.11.2)** | B4DPH5 | NA | ✓ | ✓ | ✓ | ✓ | ✓ |
| **Angiotensin-converting enzyme (ACE) (EC 3.4.15.1) (Dipeptidyl carboxypeptidase I) (Kininase II) (CD antigen CD143) [Cleaved into: Angiotensin-converting enzyme, soluble form]** | P12821 | ACE | ✓ | ✓ | ✓ | ✓ | ✓ |
| **Angiotensin-converting enzyme 2 (EC 3.4.17.23) (Angiotensin-converting enzyme homolog) (ACEH) (Angiotensin-converting enzyme-related carboxypeptidase) (ACE-related carboxypeptidase) (EC 3.4.17.-) (Metalloprotease MPROT15) [Cleaved into: Processed angiotensin-converting enzyme 2]** | Q9BYF1 | ACE2 | ✓ | ✓ | ✓ | ✓ | ✓ |
| **cDNA FLJ55560, highly similar to Retinal dehydrogenase 2** | B4DH89 | NA | ✓ | ✓ | ✓ | ✓ | ✓ |
| **Endoplasmic reticulum aminopeptidase 2 (EC 3.4.11.-) (Leukocyte-derived arginine aminopeptidase) (L-RAP)** | Q6P179 | ERAP2 | ✓ | ✓ | ✓ | ✓ | ✓ |
| **Endoplasmic reticulum DnaJ/PDI fusion protein 3** | Q6YPB0 | NA | ✓ | ✓ | ✓ | ✓ | ✓ |
| **Endosialin (Tumor endothelial marker 1) (CD antigen CD248)** | Q9HCU0 | CD248 | ✓ | ✓ | ✓ | ✓ | ✓ |
| **Ephrin-B1 (EFL-3) (ELK ligand) (ELK-L) (EPH-related receptor tyrosine kinase ligand 2) (LERK-2) [Cleaved into: Ephrin-B1 C-terminal fragment (Ephrin-B1 CTF); Ephrin-B1 intracellular domain (Ephrin-B1 ICD)]** | P98172 | EFNB1 | ✓ | ✓ | ✓ | ✓ | ✓ |
| **Galactose mutarotase (EC 5.1.3.3) (Aldose 1-epimerase)** | Q96C23 | GALM | ✓ | ✓ | ✓ | ✓ | ✓ |
| **Glutathione S-transferase Mu 1 (EC 2.5.1.18) (GST HB subunit 4) (GST class-mu 1) (GSTM1-1) (GSTM1a-1a) (GSTM1b-1b) (GTH4)** | P09488 | GSTM1 | ✓ | ✓ | ✓ | ✓ | ✓ |
| **Glutathione S-transferase Mu 4 (EC 2.5.1.18) (GST class-mu 4) (GST-Mu2) (GSTM4-4) (Leukotriene C4 synthase GSTM4) (EC 4.4.1.20)** | Q03013 | GSTM4 | ✓ | ✓ | ✓ | ✓ | ✓ |
| **Glutathione S-transferase theta-2B (EC 2.5.1.18) (Glutathione S-transferase theta-2) (GST class-theta-2)** | P0CG30 | GSTT2B | ✓ | ✓ | ✓ | ✓ | ✓ |
| **Glycogen phosphorylase, brain form (EC 2.4.1.1)** | P11216 | PYGB | ✓ | ✓ | ✓ | ✓ | ✓ |
| **Ig-like domain-containing protein** | B7ZW57 | NA | ✓ | ✓ | ✓ | ✓ | ✓ |
| **Leukocyte elastase inhibitor (LEI) (Monocyte/neutrophil elastase inhibitor) (EI) (M/NEI) (Peptidase inhibitor 2) (PI-2) (Serpin B1)** | P30740 | SERPINB1 | ✓ | ✓ | ✓ | ✓ | ✓ |
| **Leukocyte immunoglobulin-like receptor subfamily B member 5 (CD85 antigen-like family member C) (Leukocyte immunoglobulin-like receptor 8) (LIR-8) (CD antigen CD85c)** | O75023 | LILRB5 | ✓ | ✓ | ✓ | ✓ | ✓ |
| **MHC class I antigen** | A0A1X9I3T8 | HLA-A | ✓ | ✓ | ✓ | ✓ | ✓ |
| **MHC class I antigen** | A0A1X9I444 | HLA-B | ✓ | ✓ | ✓ | ✓ | ✓ |
| **MHC class I antigen** | A0A0S4T3R3 | HLA-C | ✓ | ✓ | ✓ | ✓ | ✓ |
| **MHC class I antigen** | A0A024F8H3 | HLA-B | ✓ | ✓ | ✓ | ✓ | ✓ |
| **NA** | P01889 | NA | ✓ | ✓ | ✓ | ✓ | ✓ |
| **NA** | P58107 | NA | ✓ | ✓ | ✓ | ✓ | ✓ |
| **Neprilysin (EC 3.4.24.11) (Atriopeptidase) (Common acute lymphocytic leukemia antigen) (CALLA) (Enkephalinase) (Neutral endopeptidase 24.11) (NEP) (Neutral endopeptidase) (Skin fibroblast elastase) (SFE) (CD antigen CD10)** | P08473 | MME | ✓ | ✓ | ✓ | ✓ | ✓ |
| **Oxidoreductase HTATIP2 (EC 1.1.1.-) (30 kDa HIV-1 TAT-interacting protein) (HIV-1 TAT-interactive protein 2)** | Q9BUP3 | HTATIP2 | ✓ | ✓ | ✓ | ✓ | ✓ |
| **Peptidyl-prolyl cis-trans isomerase-like 3 (PPIase) (EC 5.2.1.8) (Cyclophilin J) (CyPJ) (Cyclophilin-like protein PPIL3) (Rotamase PPIL3)** | Q9H2H8 | PPIL3 | ✓ | ✓ | ✓ | ✓ | ✓ |
| **Protein S100-A4 (Calvasculin) (Metastasin) (Placental calcium-binding protein) (Protein Mts1) (S100 calcium-binding protein A4)** | P26447 | S100A4 | ✓ | ✓ | ✓ | ✓ | ✓ |
| **RRBP1 protein** | A1A5C4 | RRBP1 | ✓ | ✓ | ✓ | ✓ | ✓ |
| **Tubulin polymerization-promoting protein (TPPP) (EC 3.6.5.-) (25 kDa brain-specific protein) (TPPP/p25) (p24) (p25-alpha)** | O94811 | TPPP | ✓ | ✓ | ✓ | ✓ | ✓ |
| **Tubulin polymerization-promoting protein family member 3 (TPPP/p20)** | Q9BW30 | TPPP3 | ✓ | ✓ | ✓ | ✓ | ✓ |
| **Neurofilament medium polypeptide (160 kDa neurofilament protein) (Neurofilament 3) (Neurofilament triplet M protein)** | A5YM63 | NEFM | ✓ | ✓ | ✓ | ✓ |  |
| **SAM and SH3 domain-containing protein 1 (Proline-glutamate repeat-containing protein)** | O94885 | SASH1 | ✓ | ✓ | ✓ | ✓ |  |
| **Forkhead box protein O1 (Forkhead box protein O1A) (Forkhead in rhabdomyosarcoma)** | Q12778 | FOXO1 | ✓ | ✓ | ✓ |  | ✓ |
| **Aminopeptidase N (AP-N) (hAPN) (EC 3.4.11.2) (Alanyl aminopeptidase) (Aminopeptidase M) (AP-M) (Microsomal aminopeptidase) (Myeloid plasma membrane glycoprotein CD13) (gp150) (CD antigen CD13)** | P15144 | ANPEP | ✓ | ✓ | ✓ |  |  |
| **Elongator complex protein 5 (Dermal papilla-derived protein 6) (S-phase 2 protein)** | Q8TE02 | ELP5 | ✓ | ✓ |  | ✓ | ✓ |
| **Inactive C-alpha-formylglycine-generating enzyme 2 (Paralog of formylglycine-generating enzyme) (pFGE) (Sulfatase-modifying factor 2)** | Q8NBJ7 | SUMF2 | ✓ | ✓ |  | ✓ | ✓ |
| **cDNA FLJ39696 fis, clone SMINT2011033, highly similar to Sorting and assembly machinery component 50 homolog** | B3KUE6 |  | ✓ | ✓ |  | ✓ |  |
| **Cytochrome b** | Q6VHB3 | CYTB | ✓ | ✓ |  | ✓ |  |
| **Multidrug and toxin extrusion protein 1 (MATE-1) (hMATE-1) (Solute carrier family 47 member 1)** | Q96FL8 | SLC47A1 | ✓ | ✓ |  | ✓ |  |
| **Protein-glutamine gamma-glutamyltransferase 2 (EC 2.3.2.13) (Erythrocyte transglutaminase) (Heart G alpha(h)) (hhG alpha(h)) (Isopeptidase TGM2) (EC 3.4.-.-) (Protein G alpha(h)) (G(h)) (Protein-glutamine deamidase TGM2) (EC 3.5.1.44) (Protein-glutamine dopaminyltransferase TGM2) (EC 2.3.1.-) (Protein-glutamine histaminyltransferase TGM2) (EC 2.3.1.-) (Protein-glutamine noradrenalinyltransferase TGM2) (EC 2.3.1.-) (Protein-glutamine serotonyltransferase TGM2) (EC 2.3.1.-) (Tissue transglutaminase) (tTG) (tTgase) (Transglutaminase C) (TG(C)) (TGC) (TGase C) (Transglutaminase H) (TGase H) (Transglutaminase II) (TGase II) (Transglutaminase-2) (TG2) (TGase-2) (hTG2)** | P21980 | TGM2 | ✓ | ✓ |  | ✓ |  |
| **Thioredoxin-like protein 4A (DIM1 protein homolog) (Spliceosomal U5 snRNP-specific 15 kDa protein) (Thioredoxin-like U5 snRNP protein U5-15kD)** | P83876 | TXNL4A | ✓ | ✓ |  | ✓ |  |
| **Mitogen-activated protein kinase kinase kinase 5 (EC 2.7.11.25) (Apoptosis signal-regulating kinase 1) (ASK-1) (MAPK/ERK kinase kinase 5) (MEK kinase 5) (MEKK 5)** | Q99683 | MAP3K5 | ✓ | ✓ |  |  |  |
| **Protein N-terminal asparagine amidohydrolase (EC 3.5.1.121) (Protein NH2-terminal asparagine amidohydrolase) (PNAA) (Protein NH2-terminal asparagine deamidase) (PNAD) (Protein N-terminal Asn amidase) (Protein N-terminal asparagine amidase) (Protein NTN-amidase)** | Q96AB6 | NTAN1 | ✓ | ✓ |  |  |  |
| **Sphingosine 1-phosphate receptor 3 (S1P receptor 3) (S1P3) (Endothelial differentiation G-protein coupled receptor 3) (Sphingosine 1-phosphate receptor Edg-3) (S1P receptor Edg-3)** | Q99500 | S1PR3 | ✓ | ✓ |  |  |  |
| **11-beta-hydroxysteroid dehydrogenase 1 (11-DH) (11-beta-HSD1) (EC 1.1.1.146) (7-oxosteroid reductase) (EC 1.1.1.201) (Corticosteroid 11-beta-dehydrogenase isozyme 1) (Short chain dehydrogenase/reductase family 26C member 1)** | P28845 | HSD11B1 | ✓ |  | ✓ |  | ✓ |
| **Glutamine synthetase (GS) (EC 6.3.1.2) (Glutamate--ammonia ligase) (Palmitoyltransferase GLUL) (EC 2.3.1.225)** | P15104 | GLUL | ✓ |  | ✓ |  | ✓ |
| **RNA binding motif (RNP1, RRM) protein 3, isoform CRA_c** | A0A024QYX3 | RBM3 | ✓ |  | ✓ |  |  |
| **UBX domain-containing protein 6 (UBX domain-containing protein 1)** | Q9BZV1 | UBXN6 | ✓ |  | ✓ |  |  |
| **Constitutive coactivator of peroxisome proliferator-activated receptor gamma (Constitutive coactivator of PPAR-gamma) (Constitutive coactivator of PPARG) (PPARG constitutive coactivator 1) (PGCC1) (Protein FAM120B)** | Q96EK7 | FAM120B | ✓ |  |  | ✓ |  |
| **Atrial natriuretic peptide-converting enzyme (EC 3.4.21.-) (Corin) (Heart-specific serine proteinase ATC2) (Pro-ANP-converting enzyme) (Transmembrane protease serine 10) [Cleaved into: Atrial natriuretic peptide-converting enzyme, N-terminal propeptide; Atrial natriuretic peptide-converting enzyme, activated protease fragment; Atrial natriuretic peptide-converting enzyme, 180 kDa soluble fragment; Atrial natriuretic peptide-converting enzyme, 160 kDa soluble fragment; Atrial natriuretic peptide-converting enzyme, 100 kDa soluble fragment]** | Q9Y5Q5 | CORIN | ✓ |  |  |  | ✓ |
| **Biogenesis of lysosome-related organelles complex 1 subunit 3 (BLOC-1 subunit 3)** | Q6QNY0 | BLOC1S3 | ✓ |  |  |  | ✓ |
| **Inositol 1,3,4-triphosphate 5/6 kinase, isoform CRA_a** | A0A024R6H3 | ITPK1 | ✓ |  |  |  | ✓ |
| **Carbonic anhydrase 12 (EC 4.2.1.1) (Carbonate dehydratase XII) (Carbonic anhydrase XII) (CA-XII) (Tumor antigen HOM-RCC-3.1.3)** | O43570 | CA12 |  | ✓ | ✓ | ✓ | ✓ |
| **cDNA FLJ55458, highly similar to Programmed cell death 6-interacting protein** | B4DHD2 | NA |  | ✓ | ✓ | ✓ | ✓ |
| **Erythrocyte membrane protein band 4.1 like 3 (cDNA FLJ77757)** | A8K968 | EPB41L3 |  | ✓ | ✓ | ✓ | ✓ |
| **Inactive phospholipase C-like protein 1 (PLC-L1) (Phospholipase C-deleted in lung carcinoma) (Phospholipase C-related but catalytically inactive protein) (PRIP)** | Q15111 | PLCL1 |  | ✓ | ✓ | ✓ | ✓ |
| **Insulin-like growth factor-binding protein 5 (IBP-5) (IGF-binding protein 5) (IGFBP-5)** | P24593 | IGFBP5 |  | ✓ | ✓ | ✓ | ✓ |
| **Methanethiol oxidase (MTO) (EC 1.8.3.4) (56 kDa selenium-binding protein) (SBP56) (SP56) (Selenium-binding protein 1)** | Q13228 | SELENBP1 |  | ✓ | ✓ | ✓ | ✓ |
| **Mitochondrial enolase superfamily member 1 (EC 4.2.1.68) (Antisense RNA to thymidylate synthase) (rTS) (L-fuconate dehydratase)** | Q7L5Y1 | ENOSF1 |  | ✓ | ✓ | ✓ | ✓ |
| **NAD(P)H dehydrogenase [quinone] 1 (EC 1.6.5.2) (Azoreductase) (DT-diaphorase) (DTD) (Menadione reductase) (NAD(P)H:quinone oxidoreductase 1) (Phylloquinone reductase) (Quinone reductase 1) (QR1)** | P15559 | NQO1 |  | ✓ | ✓ | ✓ | ✓ |
| **PDZ and LIM domain protein 1 (C-terminal LIM domain protein 1) (Elfin) (LIM domain protein CLP-36)** | O00151 | PDLIM1 |  | ✓ | ✓ | ✓ | ✓ |
| **Platelet glycoprotein Ib beta chain (GP-Ib beta) (GPIb-beta) (GPIbB) (Antigen CD42b-beta) (CD antigen CD42c)** | P13224 | GP1BB |  | ✓ | ✓ | ✓ | ✓ |
| **Putative monooxygenase p33MONOX (EC 1.-.-.-) (Brain-derived rescue factor p60MONOX) (Flavin monooxygenase motif-containing protein of 33 kDa)** | Q96A73 | KIAA1191 |  | ✓ | ✓ | ✓ | ✓ |
| **Ribose-phosphate pyrophosphokinase 2 (EC 2.7.6.1) (PPRibP) (Phosphoribosyl pyrophosphate synthase II) (PRS-II)** | P11908 | PRPS2 |  | ✓ | ✓ | ✓ | ✓ |
| **Ribosyldihydronicotinamide dehydrogenase [quinone] (EC 1.10.5.1) (NRH dehydrogenase [quinone] 2) (NRH:quinone oxidoreductase 2) (Quinone reductase 2) (QR2)** | P16083 | NQO2 |  | ✓ | ✓ | ✓ | ✓ |
| **Transforming growth factor beta receptor type 3 (TGF-beta receptor type 3) (TGFR-3) (Betaglycan) (Transforming growth factor beta receptor III) (TGF-beta receptor type III)** | Q03167 | TGFBR3 |  | ✓ | ✓ | ✓ | ✓ |
| **MHC class I antigen (MHC class I protein)** | K7WT83 | HLA-A |  | ✓ | ✓ | ✓ |  |
| **Adipose-secreted signaling protein** | Q9GZN8 | ADISSP |  | ✓ | ✓ |  | ✓ |
| **Aldehyde dehydrogenase family 3 member B1 (EC 1.2.1.28) (EC 1.2.1.5) (EC 1.2.1.7) (Aldehyde dehydrogenase 7) (Long-chain fatty aldehyde dehydrogenase) (EC 1.2.1.48) (Medium-chain fatty aldehyde dehydrogenase)** | P43353 | ALDH3B1 |  | ✓ | ✓ |  | ✓ |
| **Beta-2-microglobulin [Cleaved into: Beta-2-microglobulin form pI 5.3]** | P61769 | B2M |  | ✓ | ✓ |  | ✓ |
| **Fatty acid-binding protein 5 (Epidermal-type fatty acid-binding protein) (E-FABP) (Fatty acid-binding protein, epidermal) (Psoriasis-associated fatty acid-binding protein homolog) (PA-FABP)** | Q01469 | FABP5 |  | ✓ | ✓ |  | ✓ |
| **Fatty acid-binding protein, heart (Fatty acid-binding protein 3) (Heart-type fatty acid-binding protein) (H-FABP) (Mammary-derived growth inhibitor) (MDGI) (Muscle fatty acid-binding protein) (M-FABP)** | P05413 | FABP3 |  | ✓ | ✓ |  | ✓ |
| **Poly(rC)-binding protein 3 (Alpha-CP3) (PCBP3-overlapping transcript) (PCBP3-overlapping transcript 1)** | P57721 | PCBP3 |  | ✓ | ✓ |  | ✓ |
| **Sodium/hydrogen exchanger 9 (Na(+)/H(+) exchanger 9) (NHE-9) (Solute carrier family 9 member 9)** | Q8IVB4 | SLC9A9 |  | ✓ | ✓ |  | ✓ |
| **Sulfotransferase** | A0A024QZB4 | hCG_1993905 |  | ✓ | ✓ |  | ✓ |
| **COUP transcription factor 2 (COUP-TF2) (Apolipoprotein A-I regulatory protein 1) (ARP-1) (COUP transcription factor II) (COUP-TF II) (Nuclear receptor subfamily 2 group F member 2)** | P24468 | NR2F2 |  | ✓ | ✓ |  |  |
| **HLA DR-beta-III** | Q30131 | NA |  | ✓ | ✓ |  |  |
| **Parathymosin** | P20962 | PTMS |  | ✓ | ✓ |  |  |
| **Protein phosphatase inhibitor 2 (IPP-2)** | P41236 | PPP1R2 |  | ✓ | ✓ |  |  |
| **Calcitonin gene-related peptide type 1 receptor (CGRP type 1 receptor) (Calcitonin receptor-like receptor) (CRLR)** | Q16602 | CALCRL |  | ✓ |  | ✓ | ✓ |
| **IgG receptor FcRn large subunit p51 (FcRn) (IgG Fc fragment receptor transporter alpha chain) (Neonatal Fc receptor)** | P55899 | FCGRT |  | ✓ |  | ✓ | ✓ |
| **Lymphatic vessel endothelial hyaluronic acid receptor 1 (LYVE-1) (Cell surface retention sequence-binding protein 1) (CRSBP-1) (Extracellular link domain-containing protein 1) (Hyaluronic acid receptor)** | Q9Y5Y7 | LYVE1 |  | ✓ |  | ✓ | ✓ |
| **Phosphatidylcholine transfer protein (PC-TP) (START domain-containing protein 2) (StARD2) (StAR-related lipid transfer protein 2)** | Q9UKL6 | PCTP |  | ✓ |  | ✓ | ✓ |
| **Solute carrier organic anion transporter family member 2A1 (SLCO2A1) (OATP2A1) (PHOAR2) (Prostaglandin transporter) (PGT) (Solute carrier family 21 member 2) (SLC21A2)** | Q92959 | SLCO2A1 |  | ✓ |  | ✓ | ✓ |
| **Tumor necrosis factor alpha-induced protein 2 (TNF alpha-induced protein 2) (Primary response gene B94 protein)** | Q03169 | TNFAIP2 |  | ✓ |  | ✓ | ✓ |
| **Acid ceramidase (EC 3.5.1.23)** | A8K0B6 | NA |  | ✓ |  | ✓ |  |
| **Aldehyde oxidase (EC 1.2.3.1) (Aldehyde oxidase 1) (Azaheterocycle hydroxylase) (EC 1.17.3.-)** | Q06278 | AOX1 |  | ✓ |  |  | ✓ |
| **Aspartoacylase (EC 3.5.1.15) (Aminoacylase-2) (ACY-2)** | P45381 | ASPA |  | ✓ |  |  | ✓ |
| **Calpastatin** | D6RBR1 | CAST |  | ✓ |  |  | ✓ |
| **cGMP-dependent 3',5'-cyclic phosphodiesterase (EC 3.1.4.17) (Cyclic GMP-stimulated phosphodiesterase) (CGS-PDE) (cGSPDE)** | O00408 | PDE2A |  | ✓ |  |  | ✓ |
| **E3 ubiquitin-protein ligase TRIM32 (EC 2.3.2.27) (72 kDa Tat-interacting protein) (RING-type E3 ubiquitin transferase TRIM32) (Tripartite motif-containing protein 32) (Zinc finger protein HT2A)** | Q13049 | TRIM32 |  | ✓ |  |  | ✓ |
| **MHC class II antigen** | K4RIC6 | HLA-DRB1 |  | ✓ |  |  | ✓ |
| **Phosphatase and actin regulator 2** | O75167 | PHACTR2 |  | ✓ |  |  | ✓ |
| **Pseudouridylate synthase TRUB1 (EC 5.4.99.-) (TruB pseudouridine synthase homolog 1) (tRNA pseudouridine 55 synthase TRUB1) (Psi55 synthase TRUB1) (EC 5.4.99.25)** | Q8WWH5 | TRUB1 |  | ✓ |  |  | ✓ |
| **Scavenger receptor class A member 5 (Scavenger receptor hlg)** | Q6ZMJ2 | SCARA5 |  | ✓ |  |  | ✓ |
| **Epiplakin (450 kDa epidermal antigen)** | P58107 | EPPK1 |  |  | ✓ | ✓ | ✓ |
| **Katanin p80 WD40 repeat-containing subunit B1 (Katanin p80 subunit B1) (p80 katanin)** | Q9BVA0 | KATNB1 |  |  | ✓ | ✓ | ✓ |
| **Proteasome assembly chaperone 2** | Q9P1R6 | NA |  |  | ✓ | ✓ | ✓ |
| **6-phosphogluconate dehydrogenase, decarboxylating (EC 1.1.1.44)** | B4DL86 | NA |  |  | ✓ |  | ✓ |
| **Alpha-hemoglobin-stabilizing protein (Erythroid differentiation-related factor) (Erythroid-associated factor)** | Q9NZD4 | AHSP |  |  | ✓ |  | ✓ |
| **Ankyrin-1 (ANK-1) (Ankyrin-R) (Erythrocyte ankyrin)** | P16157 | ANK1 |  |  | ✓ |  | ✓ |
| **Arginase-1 (EC 3.5.3.1) (Liver-type arginase) (Type I arginase)** | P05089 | ARG1 |  |  | ✓ |  | ✓ |
| **Beta-adducin (Erythrocyte adducin subunit beta)** | P35612 | ADD2 |  |  | ✓ |  | ✓ |
| **Beta-globin protein** | Q9UM85 | beta-globin |  |  | ✓ |  | ✓ |
| **Carbonic anhydrase 1 (EC 4.2.1.1) (Carbonate dehydratase I) (Carbonic anhydrase B) (CAB) (Carbonic anhydrase I) (CA-I) (Cyanamide hydratase CA1) (EC 4.2.1.69)** | P00915 | CA1 |  |  | ✓ |  | ✓ |
| **Carbonic anhydrase 2 (EC 4.2.1.1) (Carbonate dehydratase II) (Carbonic anhydrase C) (CAC) (Carbonic anhydrase II) (CA-II) (Cyanamide hydratase CA2) (EC 4.2.1.69)** | P00918 | CA2 |  |  | ✓ |  | ✓ |
| **Caspase-6 (CASP-6) (CSP-6) (EC 3.4.22.59) (Apoptotic protease Mch-2) [Cleaved into: Caspase-6 subunit p18 (Caspase-6 subunit p20); Caspase-6 subunit p11 (Caspase-6 subunit p10)]** | P55212 | CASP6 |  |  | ✓ |  | ✓ |
| **Cdc42 effector protein 4 (Binder of Rho GTPases 4)** | Q9H3Q1 | CDC42EP4 |  |  | ✓ |  | ✓ |
| **F-box only protein 7** | Q9Y3I1 | FBXO7 |  |  | ✓ |  | ✓ |
| **G-protein coupled receptor 161 (G-protein coupled receptor RE2)** | Q8N6U8 | GPR161 |  |  | ✓ |  | ✓ |
| **Hemoglobin delta-beta fusion protein** | Q5XTR9 | HBD/HBB |  |  | ✓ |  | ✓ |
| **Hemoglobin subunit delta** | E9PEW8 | HBD |  |  | ✓ |  | ✓ |
| **NmrA-like family domain-containing protein 1** | Q9HBL8 | NMRAL1 |  |  | ✓ |  | ✓ |
| **Retinal dehydrogenase 2 (RALDH 2) (RalDH2) (EC 1.2.1.36) (Aldehyde dehydrogenase family 1 member A2) (ALDH1A2) (Retinaldehyde-specific dehydrogenase type 2) (RALDH(II))** | O94788 | ALDH1A2 |  |  | ✓ |  | ✓ |
| **Serine hydroxymethyltransferase, cytosolic (SHMT) (EC 2.1.2.1) (Glycine hydroxymethyltransferase) (Serine methylase)** | P34896 | SHMT1 |  |  | ✓ |  | ✓ |
| **Spectrin alpha chain, erythrocytic 1 (Erythroid alpha-spectrin)** | P02549 | SPTA1 |  |  | ✓ |  | ✓ |
| **Actin-binding LIM protein 3 (abLIM-3) (Actin-binding LIM protein family member 3)** | O94929 | ABLIM3 |  |  |  | ✓ | ✓ |
|  |  |  |  |  |  |  |  |

**Supplementary information 14:** Table showing the results of functional enrichment analysis of KEGG pathways performed using the STRING database on all proteins for which a log-fold change was available between the IOL and elCS group under the pre-contracting condition (n=7341). Proteins were ranked by their log-fold change, and the Kolmogorov-Smirnov test was applied to detect whether proteins associated with each pathway were non-randomly distributed across the ranked list. The “Genes mapped” refers to the number of proteins under the pre-contracting condition found in the pathway, and the “Total genes in set” refers to the total number of proteins within the KEGG pathway; higher enrichment scores indicate stronger enrichment, and the direction of enrichment indicates the direction of enrichment (bottom = downregulated in IOL group, top = upregulated in IOL group; both ends = proteins both upregulated and downregulated); FDR is the false discovery rate, considered significant if ≤0.05.

|  |  |  |  |  |  |
| --- | --- | --- | --- | --- | --- |
| **Significantly enriched pathway** | **Genes mapped** | **Total genes in set** | **Enrichment score** | **Direction of enrichment** | **FDR** |
| **ECM-receptor interaction** | 43 | 88 | 0.90 | bottom | <0.001 |
| **Complement and coagulation cascades** | 54 | 82 | 0.86 | bottom | <0.001 |
| **Staphylococcus aureus infection** | 34 | 86 | 0.82 | both ends | <0.001 |
| **Systemic lupus erythematosus** | 40 | 94 | 0.75 | bottom | <0.001 |
| **Amoebiasis** | 49 | 101 | 0.42 | bottom | <0.001 |
| **Protein digestion and absorption** | 28 | 100 | 0.39 | bottom | <0.001 |
| **Citrate cycle (TCA cycle)** | 21 | 28 | 0.37 | bottom | <0.001 |
| **Spliceosome** | 92 | 132 | 0.32 | top | <0.001 |
| **Carbon metabolism** | 75 | 116 | 0.27 | bottom | <0.001 |
| **Retrograde endocannabinoid signalling** | 66 | 142 | 0.25 | bottom | <0.001 |
| **Aminoacyl-tRNA biosynthesis** | 32 | 44 | 0.23 | bottom | 0.001 |
| **Endocytosis** | 134 | 241 | 0.18 | top | <0.001 |
| **Oxidative phosphorylation** | 86 | 128 | 0.17 | bottom | <0.001 |
| **Prion disease** | 178 | 263 | 0.12 | bottom | <0.001 |
| **Thermogenesis** | 120 | 226 | 0.10 | bottom | <0.001 |
| **Parkinson disease** | 157 | 236 | 0.06 | bottom | <0.001 |
| **Non-alcoholic fatty liver disease** | 88 | 146 | 0.01 | bottom | <0.001 |
|  |  |  |  |  |  |

**Supplementary information 15:** Table showing the results of functional enrichment analysis of local network clusters performed using the STRING database on all proteins for which there was a log-fold change available between the IOL and elCS group under the pre-contracting condition (n=7341). The proteins were ranked by their log-fold change. Two statistical methods were used depending on the cluster: the Kolmogorov-Smirnov (KS) test was applied to detect whether proteins associated with each cluster were non-randomly distributed across the ranked list; and the average fold change (AFC) method, which calculates mean log-fold change of proteins within each cluster compared to background. The “Genes mapped” refers to the number of proteins under the pre-contracting condition found in the pathway, and the “Total genes in cluster” refers to the total number of proteins within the STRING cluster; higher enrichment scores indicate stronger enrichment, and “Direction of enrichment” indicates the direction of enrichment (bottom = downregulated in IOL group, top = upregulated in IOL group; both ends = proteins both upregulated and downregulated); FDR is the false discovery rate, considered significant if ≤0.05.

| **Significantly enriched cluster** | **Genes mapped** | **total genes in cluster** | **Enrichment score** | **Direction of enrichment** | **FDR** | **Method** |
| --- | --- | --- | --- | --- | --- | --- |
| **Alpha-1-acid glycoprotein, and Protein-losing enteropathy** | 5 | 5 | 2.61 | bottom | 0.009 | AFC |
| **Mixed, incl. Inter-alpha-trypsin inhibitor heavy chain C-terminus, and Alpha-1-acid glycoprotein** | 9 | 10 | 2.10 | bottom | <0.001 | AFC |
| **Mixed, incl. COVID-19, thrombosis and anticoagulation, and Inter-alpha-trypsin inhibitor heavy chain C-terminus** | 19 | 21 | 1.46 | bottom | <0.001 | AFC |
| **Mixed, incl. COVID-19, thrombosis and anticoagulation, and Scavenging of heme from plasma** | 24 | 26 | 1.44 | bottom | <0.001 | KS |
| **Complement cascade** | 25 | 40 | 1.18 | bottom | <0.001 | KS |
| **Collagen chain trimerization** | 21 | 38 | 1.16 | bottom | <0.001 | KS |
| **Striated muscle contraction pathway, and Myosin II complex** | 16 | 44 | 1.16 | bottom | 0.01 | AFC |
| **Complement and coagulation cascades, and Positive regulation of opsonization** | 73 | 115 | 1.15 | bottom | <0.001 | KS |
| **Collagen chain trimerization** | 19 | 32 | 1.14 | bottom | 0.002 | AFC |
| **Collagen chain trimerization** | 22 | 43 | 1.13 | bottom | <0.001 | KS |
| **Complement and coagulation cascades, and Positive regulation of opsonization** | 69 | 109 | 1.12 | bottom | <0.001 | KS |
| **Complement and coagulation cascades, and Protein-lipid complex** | 93 | 161 | 1.11 | bottom | <0.001 | KS |
| **Formation of the cornified envelope, and Autosomal recessive congenital ichthyosis** | 17 | 125 | 1.11 | bottom | 0.007 | AFC |
| **Complement cascade** | 30 | 50 | 1.10 | bottom | <0.001 | KS |
| **Initial triggering of complement, and Regulation of complement activation** | 17 | 31 | 1.10 | bottom | 0.007 | AFC |
| **Complement and coagulation cascades, and Protein-lipid complex** | 94 | 166 | 1.10 | bottom | <0.001 | KS |
| **Mixed, incl. Complement and coagulation cascades, and Protein-lipid complex** | 96 | 172 | 1.09 | bottom | <0.001 | KS |
| **Hemostasis, and Dissolution of Fibrin Clot** | 35 | 50 | 1.06 | bottom | <0.001 | KS |
| **Hemostasis, and Dissolution of Fibrin Clot** | 36 | 55 | 1.05 | bottom | <0.001 | KS |
| **Collagen biosynthesis and modifying enzymes** | 26 | 49 | 1.04 | bottom | <0.001 | KS |
| **Muscle protein, and Myosin light chain kinase activity** | 22 | 57 | 0.93 | bottom | <0.001 | KS |
| **Mixed, incl. Complement and coagulation cascades, and Protein-lipid complex** | 109 | 196 | 0.89 | bottom | <0.001 | KS |
| **Elastic fibre formation, and Tolloid/BMP1 peptidase domain** | 22 | 34 | 0.86 | bottom | 0.002 | KS |
| **Collagen biosynthesis and modifying enzymes** | 34 | 60 | 0.86 | bottom | <0.001 | KS |
| **Muscle protein, and Sarcomere organization** | 23 | 73 | 0.84 | bottom | <0.001 | KS |
| **Collagen formation, and Protein complex involved in cell adhesion** | 70 | 119 | 0.78 | bottom | <0.001 | KS |
| **Extracellular matrix organization** | 102 | 180 | 0.78 | bottom | <0.001 | KS |
| **Muscle protein, and Myofibril assembly** | 25 | 79 | 0.77 | bottom | <0.001 | KS |
| **Elastic fibre formation, and Matrix metalloproteinases** | 32 | 61 | 0.77 | bottom | <0.001 | KS |
| **Mixed, incl. Chemokine-mediated signaling pathway, and Adaptive immunity** | 28 | 185 | 0.66 | both ends | 0.009 | KS |
| **Precatalytic spliceosome, and Renpenning syndrome** | 36 | 52 | 0.65 | top | <0.001 | KS |
| **Precatalytic spliceosome, and U4/U6 x U5 tri-snRNP complex** | 31 | 46 | 0.64 | top | <0.001 | KS |
| **Muscle protein, and Sarcoplasmic reticulum membrane** | 35 | 104 | 0.59 | bottom | 0.003 | KS |
| **Mixed, incl. Muscle protein, and Sarcoplasmic reticulum membrane** | 36 | 126 | 0.57 | bottom | 0.005 | KS |
| **Spliceosomal snRNP complex, and mRNA cis splicing, via spliceosome** | 45 | 65 | 0.56 | top | <0.001 | KS |
| **Precatalytic spliceosome** | 28 | 39 | 0.49 | top | 0.001 | KS |
| **U2-type spliceosomal complex, and mRNA Splicing - Major Pathway** | 68 | 127 | 0.46 | top | <0.001 | KS |
| **Citrate cycle (TCA cycle), and Lactate dehydrogenase activity** | 31 | 46 | 0.45 | bottom | <0.001 | KS |
| **U2-type spliceosomal complex, and mRNA Splicing - Major Pathway** | 62 | 114 | 0.45 | top | <0.001 | KS |
| **Citrate cycle (TCA cycle), and Pyruvate metabolism** | 37 | 56 | 0.44 | bottom | <0.001 | KS |
| **U2-type spliceosomal complex, and mRNA Splicing - Major Pathway** | 61 | 106 | 0.43 | top | <0.001 | KS |
| **Carbon metabolism, and Pyruvate metabolism** | 76 | 134 | 0.39 | bottom | <0.001 | KS |
| **U2-type spliceosomal complex, and Sm-like protein family complex** | 80 | 150 | 0.37 | top | <0.001 | KS |
| **Carbon metabolism, and Pyruvate metabolism** | 63 | 100 | 0.36 | bottom | <0.001 | KS |
| **Carbon metabolism, and Pyruvate metabolism** | 65 | 112 | 0.35 | bottom | <0.001 | KS |
| **mRNA processing, and RNA recognition motif domain** | 61 | 82 | 0.34 | top | 0.007 | KS |
| **NADH dehydrogenase (ubiquinone) activity** | 34 | 42 | 0.32 | bottom | <0.001 | KS |
| **NADH dehydrogenase (ubiquinone) activity** | 31 | 36 | 0.31 | bottom | <0.001 | KS |
| **Clathrin coat, and Presynaptic endocytosis** | 26 | 48 | 0.31 | top | 0.003 | KS |
| **Mixed, incl. Protein targeting to mitochondrion, and Mitochondrial calcium ion transport** | 69 | 104 | 0.29 | bottom | 0.009 | KS |
| **Clathrin coat, and Presynaptic endocytosis** | 43 | 85 | 0.26 | top | 0.002 | KS |
| **Respiratory chain complex** | 55 | 73 | 0.26 | bottom | <0.001 | KS |
| **Mixed, incl. Membrane coat, and Sec7, C-terminal domain superfamily** | 53 | 117 | 0.26 | top | <0.001 | KS |
| **Respiratory chain complex** | 57 | 78 | 0.26 | bottom | <0.001 | KS |
| **Clathrin coat, and Presynaptic endocytosis** | 38 | 73 | 0.24 | top | 0.008 | KS |
| **tRNA Aminoacylation** | 32 | 42 | 0.24 | bottom | 0.009 | KS |
| **tRNA Aminoacylation, and glutamyl-tRNA(Gln) amidotransferase complex** | 35 | 48 | 0.21 | bottom | 0.008 | KS |
| **Respiratory chain complex, and Complex I biogenesis** | 70 | 97 | 0.21 | bottom | <0.001 | KS |
| **Respiratory electron transport, ATP synthesis by chemiosmotic coupling, and heat production by uncoupling proteins.** | 85 | 121 | 0.19 | bottom | <0.001 | KS |
| **Respiratory electron transport, ATP synthesis by chemiosmotic coupling, and heat production by uncoupling proteins., and Proton-transporting ATP synthase complex** | 87 | 126 | 0.15 | bottom | <0.001 | KS |
| **Respiratory electron transport, ATP synthesis by chemiosmotic coupling, and heat production by uncoupling proteins., and Cytochrome complex** | 102 | 160 | 0.13 | bottom | <0.001 | KS |
| **Respiratory electron transport, ATP synthesis by chemiosmotic coupling, and heat production by uncoupling proteins., and Cytochrome complex** | 109 | 180 | 0.11 | bottom | <0.001 | KS |
| **Respiratory electron transport, ATP synthesis by chemiosmotic coupling, and heat production by uncoupling proteins., and Cytochrome complex** | 106 | 169 | 0.11 | bottom | <0.001 | KS |
|  |  |  |  |  |  |  |

**Supplementary information 16:** Table showing the results of functional enrichment analysis of KEGG pathways performed using the STRING database on all proteins for which there was a log-fold change available between the IOL and elCS group under the relaxed-phase during spontaneous contractions condition (n=7341). The proteins were ranked by their log-fold change, and the Kolmogorov-Smirnov test was applied to detect whether proteins associated with each pathway were non-randomly distributed across the ranked list. The “Genes mapped” refers to the number of proteins under the relaxed-phase during spontaneous contractions condition found in the pathway, and the “Total genes in set” refers to the total number of proteins within the KEGG pathway; higher enrichment scores indicate stronger enrichment, and the direction of enrichment indicates the direction of enrichment (bottom = downregulated in IOL group, top = upregulated in IOL group; both ends = proteins both upregulated and downregulated); FDR is the false discovery rate, considered significant if ≤0.05.

|  |  |  |  |  |  |
| --- | --- | --- | --- | --- | --- |
| **Significantly enriched pathway** | **Genes mapped** | **Total genes in set** | **Enrichment score** | **Direction of enrichment** | **FDR** |
| **ECM-receptor interaction** | 43 | 88 | 1.34 | bottom | <0.001 |
| **Drug metabolism - cytochrome P450** | 18 | 64 | 1.25 | top | <0.001 |
| **Systemic lupus erythematosus** | 40 | 94 | 0.75 | bottom | <0.001 |
| **Proteasome** | 38 | 43 | 0.63 | top | 0.004 |
| **Complement and coagulation cascades** | 54 | 82 | 0.59 | bottom | <0.001 |
| **Oxidative phosphorylation** | 86 | 128 | 0.57 | bottom | <0.001 |
| **Amoebiasis** | 49 | 101 | 0.55 | bottom | 0.001 |
| **Staphylococcus aureus infection** | 34 | 86 | 0.53 | bottom | 0.01 |
| **Citrate cycle (TCA cycle)** | 21 | 28 | 0.48 | bottom | 0.009 |
| **Cardiac muscle contraction** | 40 | 87 | 0.47 | bottom | <0.001 |
| **Retrograde endocannabinoid signaling** | 66 | 142 | 0.45 | bottom | <0.001 |
| **Arrhythmogenic right ventricular cardiomyopathy** | 35 | 77 | 0.44 | bottom | 0.001 |
| **Dilated cardiomyopathy** | 41 | 94 | 0.43 | bottom | <0.001 |
| **Protein digestion and absorption** | 28 | 100 | 0.43 | bottom | <0.001 |
| **Thermogenesis** | 120 | 226 | 0.41 | bottom | <0.001 |
| **Non-alcoholic fatty liver disease** | 88 | 146 | 0.40 | bottom | <0.001 |
| **Ribosome** | 103 | 131 | 0.38 | bottom | <0.001 |
| **Hypertrophic cardiomyopathy** | 42 | 88 | 0.36 | bottom | 0.005 |
| **Endocytosis** | 134 | 241 | 0.24 | top | <0.001 |
| **Prion disease** | 178 | 263 | 0.16 | bottom | <0.001 |
| **Parkinson disease** | 157 | 236 | 0.14 | bottom | <0.001 |
| **Huntington disease** | 174 | 295 | 0.12 | bottom | 0.002 |

**Supplementary information 17:** Table showing the results of functional enrichment analysis of local network clusters performed using the STRING database on all proteins for which there was a log-fold change available between the IOL and elCS group under the relaxed-phase during spontaneous contractions condition (n=7341). The proteins were ranked by their log-fold change. Two statistical methods were used depending on the cluster: the Kolmogorov-Smirnov (KS) test was applied to detect whether proteins associated with each cluster were non-randomly distributed across the ranked list; and the average fold change (AFC) method, which calculates mean log-fold change of proteins within each cluster compared to background. The “Genes mapped” refers to the number of proteins under the relaxed-phase during spontaneous contractions condition found in the pathway, and the “Total genes in cluster” refers to the total number of proteins within the STRING cluster; higher enrichment scores indicate stronger enrichment, and “Direction of enrichment” indicates the direction of enrichment (bottom = downregulated in IOL group, top = upregulated in IOL group; both ends = proteins both upregulated and downregulated); FDR is the false discovery rate, considered significant if ≤0.05.

|  |  |  |  |  |  |  |
| --- | --- | --- | --- | --- | --- | --- |
| **Significantly enriched cluster** | **Genes mapped** | **total genes in cluster** | **Enrichment score** | **Direction of enrichment** | **FDR** | **Method** |
| **Glutathione S-transferase, N-terminal domain, and Benzo(a)pyrene metabolism** | 9 | 17 | 2.38 | top | <0.001 | AFC |
| **Elastic fibre formation, and Tolloid/BMP1 peptidase domain** | 22 | 34 | 2.04 | bottom | <0.001 | KS |
| **Elastic fibre formation, and Transforming growth factor beta receptor complex assembly** | 18 | 27 | 2.04 | bottom | <0.001 | KS |
| **Elastic fibre formation** | 10 | 14 | 1.96 | bottom | 0.002 | AFC |
| **Elastic fibre formation, and Matrix metalloproteinases** | 32 | 61 | 1.66 | bottom | <0.001 | KS |
| **Collagen chain trimerization** | 21 | 38 | 1.44 | bottom | <0.001 | KS |
| **Collagen chain trimerization** | 22 | 43 | 1.43 | bottom | <0.001 | KS |
| **Collagen chain trimerization** | 19 | 32 | 1.35 | bottom | <0.001 | AFC |
| **Collagen biosynthesis and modifying enzymes** | 26 | 49 | 1.35 | bottom | <0.001 | KS |
| **Extracellular matrix organization** | 102 | 180 | 1.31 | bottom | <0.001 | KS |
| **Collagen formation, and Protein complex involved in cell adhesion** | 70 | 119 | 1.14 | bottom | <0.001 | KS |
| **Protein complex involved in cell adhesion, and Junctional epidermolysis bullosa** | 33 | 53 | 1.14 | bottom | 0.002 | KS |
| **Collagen biosynthesis and modifying enzymes** | 34 | 60 | 1.13 | bottom | <0.001 | KS |
| **Mixed, incl. COVID-19, thrombosis and anticoagulation, and Inter-alpha-trypsin inhibitor heavy chain C-terminus** | 19 | 21 | 1.09 | bottom | 0.008 | AFC |
| **Mixed, incl. COVID-19, thrombosis and anticoagulation, and Scavenging of heme from plasma** | 24 | 26 | 0.98 | bottom | <0.001 | KS |
| **Complement cascade** | 30 | 50 | 0.91 | bottom | <0.001 | KS |
| **Complement cascade** | 25 | 40 | 0.90 | bottom | <0.001 | KS |
| **Muscle protein, and Sarcomere organization** | 23 | 73 | 0.87 | bottom | <0.001 | KS |
| **Muscle protein, and Myosin light chain kinase activity** | 22 | 57 | 0.85 | bottom | <0.001 | KS |
| **NADH dehydrogenase (ubiquinone) activity** | 34 | 42 | 0.81 | bottom | <0.001 | KS |
| **Muscle protein, and Myofibril assembly** | 25 | 79 | 0.80 | bottom | <0.001 | KS |
| **Respiratory chain complex** | 55 | 73 | 0.77 | bottom | <0.001 | KS |
| **NADH dehydrogenase (ubiquinone) activity** | 31 | 36 | 0.76 | bottom | <0.001 | KS |
| **Respiratory chain complex** | 57 | 78 | 0.75 | bottom | <0.001 | KS |
| **NADH dehydrogenase (ubiquinone) activity** | 22 | 24 | 0.74 | bottom | <0.001 | KS |
| **Complement and coagulation cascades, and Positive regulation of opsonization** | 73 | 115 | 0.74 | bottom | <0.001 | KS |
| **Complement and coagulation cascades, and Protein-lipid complex** | 93 | 161 | 0.74 | bottom | <0.001 | KS |
| **Mixed, incl. Complement and coagulation cascades, and Protein-lipid complex** | 96 | 172 | 0.73 | bottom | <0.001 | KS |
| **Complement and coagulation cascades, and Protein-lipid complex** | 94 | 166 | 0.73 | bottom | <0.001 | KS |
| **Complement and coagulation cascades, and Positive regulation of opsonization** | 69 | 109 | 0.72 | bottom | <0.001 | KS |
| **Mixed, incl. Mitochondrial calcium ion transport, and Miro GTPase Cycle** | 32 | 52 | 0.67 | bottom | <0.001 | KS |
| **Muscle protein, and Sarcoplasmic reticulum membrane** | 35 | 104 | 0.66 | bottom | <0.001 | KS |
| **Mixed, incl. Muscle protein, and Sarcoplasmic reticulum membrane** | 36 | 126 | 0.65 | bottom | <0.001 | KS |
| **Mixed, incl. Hemoglobin complex, and Actin filament capping** | 29 | 49 | 0.62 | top | <0.001 | KS |
| **Respiratory chain complex, and Complex I biogenesis** | 70 | 97 | 0.61 | bottom | <0.001 | KS |
| **Mixed, incl. Hemoglobin complex, and Actin filament capping** | 31 | 60 | 0.60 | top | <0.001 | KS |
| **Mixed, incl. Protein targeting to mitochondrion, and Mitochondrial calcium ion transport** | 69 | 104 | 0.60 | bottom | <0.001 | KS |
| **Hemostasis, and Dissolution of Fibrin Clot** | 36 | 55 | 0.60 | bottom | <0.001 | KS |
| **Mixed, incl. Hemoglobin complex, and Actin filament capping** | 32 | 68 | 0.60 | top | <0.001 | KS |
| **Hemostasis, and Dissolution of Fibrin Clot** | 35 | 50 | 0.59 | bottom | <0.001 | KS |
| **Mixed, incl. Muscle protein, and Sarcoplasmic reticulum membrane** | 38 | 139 | 0.59 | bottom | 0.001 | KS |
| **Respiratory electron transport, ATP synthesis by chemiosmotic coupling, and heat production by uncoupling proteins.** | 85 | 121 | 0.58 | bottom | <0.001 | KS |
| **Mixed, incl. Complement and coagulation cascades, and Protein-lipid complex** | 109 | 196 | 0.57 | bottom | <0.001 | KS |
| **Proteasome** | 39 | 42 | 0.57 | top | 0.004 | KS |
| **Proteasome, and Proteasome assembly** | 49 | 59 | 0.57 | top | <0.001 | KS |
| **Proteasome** | 44 | 47 | 0.57 | top | <0.001 | KS |
| **Proteasome** | 47 | 53 | 0.56 | top | <0.001 | KS |
| **Respiratory electron transport, ATP synthesis by chemiosmotic coupling, and heat production by uncoupling proteins., and Proton-transporting ATP synthase complex** | 87 | 126 | 0.55 | bottom | <0.001 | KS |
| **Mitochondrial outer membrane translocase complex, and TIM23 mitochondrial import inner membrane translocase complex** | 30 | 42 | 0.54 | bottom | <0.001 | KS |
| **Mitochondrial protein import, and SAM complex** | 37 | 52 | 0.54 | bottom | <0.001 | KS |
| **Cytoplasmic ribosomal proteins** | 23 | 24 | 0.51 | bottom | 0.002 | KS |
| **Oxygen-dependent proline hydroxylation of Hypoxia-inducible Factor Alpha, and Proteasome assembly** | 56 | 81 | 0.51 | top | <0.001 | KS |
| **Organellar large ribosomal subunit** | 34 | 44 | 0.51 | bottom | <0.001 | KS |
| **Mixed, incl. Contractile fiber, and Microfilament motor activity** | 66 | 185 | 0.51 | bottom | <0.001 | KS |
| **Organellar large ribosomal subunit** | 37 | 50 | 0.50 | bottom | <0.001 | KS |
| **Oxygen-dependent proline hydroxylation of Hypoxia-inducible Factor Alpha, and Proteasome assembly** | 59 | 88 | 0.50 | top | <0.001 | KS |
| **Respiratory electron transport, ATP synthesis by chemiosmotic coupling, and heat production by uncoupling proteins., and Cytochrome complex** | 102 | 160 | 0.49 | bottom | <0.001 | KS |
| **Organellar large ribosomal subunit** | 25 | 35 | 0.49 | bottom | 0.003 | KS |
| **Organellar ribosome** | 55 | 77 | 0.47 | bottom | <0.001 | KS |
| **Citrate cycle (TCA cycle), and Pyruvate metabolism** | 37 | 56 | 0.47 | bottom | 0.005 | KS |
| **Respiratory electron transport, ATP synthesis by chemiosmotic coupling, and heat production by uncoupling proteins., and Cytochrome complex** | 109 | 180 | 0.46 | bottom | <0.001 | KS |
| **Respiratory electron transport, ATP synthesis by chemiosmotic coupling, and heat production by uncoupling proteins., and Cytochrome complex** | 106 | 169 | 0.46 | bottom | <0.001 | KS |
| **Organellar ribosome, and Regulation of mitochondrial gene expression** | 67 | 115 | 0.45 | bottom | <0.001 | KS |
| **Cytoplasmic ribosomal proteins** | 51 | 53 | 0.45 | bottom | <0.001 | KS |
| **Mitochondrial translation initiation** | 59 | 82 | 0.44 | bottom | <0.001 | KS |
| **Cytoplasmic ribosomal proteins** | 44 | 45 | 0.44 | bottom | <0.001 | KS |
| **Cytoplasmic ribosomal proteins** | 31 | 32 | 0.44 | bottom | <0.001 | KS |
| **Mitochondrial translation initiation** | 63 | 87 | 0.44 | bottom | <0.001 | KS |
| **Cytoplasmic ribosomal proteins** | 57 | 61 | 0.43 | bottom | <0.001 | KS |
| **Cytoplasmic ribosomal proteins** | 39 | 40 | 0.43 | bottom | <0.001 | KS |
| **Mixed, incl. Oxygen-dependent proline hydroxylation of Hypoxia-inducible Factor Alpha, and Ubiquitin conjugating enzyme activity** | 86 | 146 | 0.41 | top | <0.001 | KS |
| **Cytoplasmic ribosomal proteins** | 62 | 66 | 0.40 | bottom | <0.001 | KS |
| **Viral mRNA Translation** | 63 | 71 | 0.39 | bottom | <0.001 | KS |
| **Viral mRNA Translation** | 65 | 76 | 0.38 | bottom | <0.001 | KS |
| **Viral mRNA Translation** | 66 | 81 | 0.37 | bottom | <0.001 | KS |
| **Viral mRNA Translation** | 67 | 87 | 0.36 | bottom | <0.001 | KS |
| **Clathrin coat, and Eps15 homology domain** | 23 | 37 | 0.33 | top | 0.007 | KS |
| **Viral mRNA Translation, and Sec61 translocon complex** | 77 | 108 | 0.32 | bottom | <0.001 | KS |
| **Clathrin coat, and Presynaptic endocytosis** | 26 | 48 | 0.32 | top | 0.002 | KS |
| **Clathrin coat, and Presynaptic endocytosis** | 43 | 85 | 0.31 | top | 0.005 | KS |
| **Mixed, incl. Membrane coat, and Sec7, C-terminal domain superfamily** | 53 | 117 | 0.30 | top | 0.004 | KS |
| **Clathrin coat, and Presynaptic endocytosis** | 38 | 73 | 0.27 | top | 0.01 | KS |
| **Mixed, incl. Sensory processing of sound by outer hair cells of the cochlea, and Hemolytic anemia** | 42 | 148 | 0.27 | top | <0.001 | KS |
| **Eukaryotic Translation Elongation, and Sec61 translocon complex** | 81 | 116 | 0.25 | bottom | <0.001 | KS |
| **Eukaryotic Translation Elongation, and This family consists of several GAGE and XAGE proteins which are found exclusively in humans. The function of this family is unknown although they have been implicated in human cancers (PMID:11992404)** | 82 | 131 | 0.25 | bottom | <0.001 | KS |
| **Eukaryotic Translation Elongation, and This family consists of several GAGE and XAGE proteins which are found exclusively in humans. The function of this family is unknown although they have been implicated in human cancers (PMID:11992404)** | 85 | 140 | 0.24 | bottom | <0.001 | KS |
| **Eukaryotic Translation Elongation, and This family consists of several GAGE and XAGE proteins which are found exclusively in humans. The function of this family is unknown although they have been implicated in human cancers (PMID:11992404)** | 88 | 150 | 0.22 | bottom | <0.001 | KS |
| **Mixed, incl. Eukaryotic Translation Elongation, and This family consists of several GAGE and XAGE proteins which are found exclusively in humans. The function of this family is unknown although they have been implicated in human cancers (PMID:11992404)** | 94 | 163 | 0.21 | bottom | <0.001 | KS |
| **Mixed, incl. Eukaryotic Translation Elongation, and This family consists of several GAGE and XAGE proteins which are found exclusively in humans. The function of this family is unknown although they have been implicated in human cancers (PMID:11992404)** | 97 | 168 | 0.21 | bottom | <0.001 | KS |
| **Mixed, incl. Protein targeting to ER, and Oligosaccharyltransferase complex** | 59 | 80 | 0.10 | bottom | 0.009 | KS |
|  |  |  |  |  |  |  |

**Supplementary information 18:** Table showing the results of functional enrichment analysis of KEGG pathways performed using the STRING database on all proteins for which there was a log-fold change available between the IOL and elCS group under the contracted-phase during spontaneous contractions condition (n=7340). The proteins were ranked by their log-fold change, and the Kolmogorov-Smirnov test was applied to detect whether proteins associated with each pathway were non-randomly distributed across the ranked list. The “Genes mapped” refers to the number of proteins under the contracted-phase during spontaneous contractions condition found in the pathway, and the “Total genes in set” refers to the total number of proteins within the KEGG pathway; higher enrichment scores indicate stronger enrichment, and the direction of enrichment indicates the direction of enrichment (bottom = downregulated in IOL group, top = upregulated in IOL group; both ends = proteins both upregulated and downregulated); FDR is the false discovery rate, considered significant if ≤0.05.

|  |  |  |  |  |  |
| --- | --- | --- | --- | --- | --- |
| **Significantly enriched pathway** | **Genes mapped** | **Total genes in set** | **Enrichment score** | **Direction of enrichment** | **FDR** |
| **ECM-receptor interaction** | 43 | 88 | 0.96 | bottom | 0.002 |
| **Complement and coagulation cascades** | 54 | 82 | 0.61 | bottom | <0.001 |
| **Systemic lupus erythematosus** | 40 | 94 | 0.52 | bottom | <0.001 |
| **Staphylococcus aureus infection** | 34 | 86 | 0.50 | bottom | 0.003 |
| **Amoebiasis** | 49 | 101 | 0.45 | bottom | 0.003 |
| **Citrate cycle (TCA cycle)** | 21 | 28 | 0.44 | bottom | 0.009 |
| **Oxidative phosphorylation** | 86 | 128 | 0.44 | bottom | <0.001 |
| **Thermogenesis** | 120 | 226 | 0.33 | bottom | <0.001 |
| **Retrograde endocannabinoid signaling** | 66 | 142 | 0.31 | bottom | <0.001 |
| **Non-alcoholic fatty liver disease** | 88 | 146 | 0.28 | bottom | <0.001 |
| **Cardiac muscle contraction** | 40 | 87 | 0.24 | bottom | <0.001 |
| **Protein digestion and absorption** | 28 | 100 | 0.22 | bottom | 0.003 |
| **Prion disease** | 178 | 263 | 0.20 | bottom | <0.001 |
| **Parkinson disease** | 157 | 236 | 0.19 | bottom | <0.001 |
| **Huntington disease** | 174 | 295 | 0.17 | bottom | 0.003 |
|  |  |  |  |  |  |

**Supplementary information 19:** Table showing the results of functional enrichment analysis of local network clusters performed using the STRING database on all proteins for which there was a log-fold change available between the IOL and elCS group under the contracted-phase during spontaneous contractions condition (n=7340). The proteins were ranked by their log-fold change. Two statistical methods were used depending on the cluster: the Kolmogorov-Smirnov (KS) test was applied to detect whether proteins associated with each cluster were non-randomly distributed across the ranked list; and the average fold change (AFC) method, which calculates mean log-fold change of proteins within each cluster compared to background. The “Genes mapped” refers to the number of proteins under the contracted-phase during spontaneous contractions condition found in the pathway, and the “Total genes in cluster” refers to the total number of proteins within the STRING cluster; higher enrichment scores indicate stronger enrichment, and “Direction of enrichment” indicates the direction of enrichment (bottom = downregulated in IOL group, top = upregulated in IOL group; both ends = proteins both upregulated and downregulated); FDR is the false discovery rate, considered significant if ≤0.05.

|  |  |  |  |  |  |  |
| --- | --- | --- | --- | --- | --- | --- |
| **Significantly enriched cluster** | **Genes mapped** | **Total genes in cluster** | **Enrichment score** | **Direction of enrichment** | **FDR** | **Method** |
| **Glutathione S-transferase, N-terminal domain, and Benzo(a)pyrene metabolism** | 9 | 17 | 2.25 | top | <0.001 | AFC |
| **Type III transforming growth factor beta receptor binding, and Transforming growth factor beta receptor complex assembly** | 6 | 8 | 2.22 | both ends | 0.005 | AFC |
| **Elastic fibre formation, and Tolloid/BMP1 peptidase domain** | 22 | 34 | 1.49 | bottom | <0.001 | KS |
| **Elastic fibre formation, and Transforming growth factor beta receptor complex assembly** | 18 | 27 | 1.45 | bottom | <0.001 | AFC |
| **Mixed, incl. Intermediate filament head, DNA-binding domain, and Oligodendrocyte specification and differentiation, leading to myelin components for CNS** | 15 | 46 | 1.27 | bottom | 0.01 | AFC |
| **Elastic fibre formation, and Matrix metalloproteinases** | 32 | 61 | 1.25 | bottom | <0.001 | KS |
| **Collagen chain trimerization** | 21 | 38 | 1.15 | bottom | <0.001 | KS |
| **Collagen chain trimerization** | 22 | 43 | 1.12 | bottom | <0.001 | KS |
| **Collagen chain trimerization** | 19 | 32 | 1.07 | bottom | 0.009 | AFC |
| **Collagen biosynthesis and modifying enzymes** | 26 | 49 | 1.06 | bottom | <0.001 | KS |
| **Mixed, incl. COVID-19, thrombosis and anticoagulation, and Scavenging of heme from plasma** | 24 | 26 | 1.04 | bottom | <0.001 | KS |
| **Extracellular matrix organization** | 102 | 180 | 0.98 | bottom | <0.001 | KS |
| **Collagen biosynthesis and modifying enzymes** | 34 | 60 | 0.88 | bottom | <0.001 | KS |
| **Complement cascade** | 25 | 40 | 0.87 | bottom | <0.001 | KS |
| **Muscle protein, and Sarcomere organization** | 23 | 73 | 0.87 | bottom | <0.001 | KS |
| **Complement cascade** | 30 | 50 | 0.86 | bottom | <0.001 | KS |
| **Muscle protein, and Myosin light chain kinase activity** | 22 | 57 | 0.86 | bottom | 0.001 | KS |
| **Collagen formation, and Protein complex involved in cell adhesion** | 70 | 119 | 0.85 | bottom | <0.001 | KS |
| **Muscle protein, and Myofibril assembly** | 25 | 79 | 0.83 | bottom | <0.001 | KS |
| **Complement and coagulation cascades, and Protein-lipid complex** | 93 | 161 | 0.74 | bottom | <0.001 | KS |
| **Complement and coagulation cascades, and Positive regulation of opsonization** | 73 | 115 | 0.74 | bottom | <0.001 | KS |
| **Complement and coagulation cascades, and Protein-lipid complex** | 94 | 166 | 0.73 | bottom | <0.001 | KS |
| **Mixed, incl. Complement and coagulation cascades, and Protein-lipid complex** | 96 | 172 | 0.73 | bottom | <0.001 | KS |
| **Complement and coagulation cascades, and Positive regulation of opsonization** | 69 | 109 | 0.72 | bottom | <0.001 | KS |
| **Mixed, incl. Hemoglobin complex, and Actin filament capping** | 29 | 49 | 0.70 | top | <0.001 | KS |
| **Mixed, incl. Hemoglobin complex, and Actin filament capping** | 31 | 60 | 0.70 | top | <0.001 | KS |
| **Mixed, incl. Hemoglobin complex, and Actin filament capping** | 32 | 68 | 0.68 | top | <0.001 | KS |
| **Hemostasis, and Dissolution of Fibrin Clot** | 35 | 50 | 0.67 | bottom | 0.002 | KS |
| **Muscle protein, and Sarcoplasmic reticulum membrane** | 35 | 104 | 0.66 | bottom | 0.005 | KS |
| **NADH dehydrogenase (ubiquinone) activity** | 34 | 42 | 0.63 | bottom | <0.001 | KS |
| **NADH dehydrogenase (ubiquinone) activity** | 31 | 36 | 0.63 | bottom | <0.001 | KS |
| **Mixed, incl. Muscle protein, and Sarcoplasmic reticulum membrane** | 36 | 126 | 0.62 | bottom | 0.01 | KS |
| **Hemostasis, and Dissolution of Fibrin Clot** | 36 | 55 | 0.62 | bottom | 0.003 | KS |
| **NADH dehydrogenase (ubiquinone) activity** | 22 | 24 | 0.62 | bottom | <0.001 | KS |
| **Mixed, incl. Complement and coagulation cascades, and Protein-lipid complex** | 109 | 196 | 0.59 | bottom | <0.001 | KS |
| **Mixed, incl. Mitochondrial calcium ion transport, and Miro GTPase Cycle** | 32 | 52 | 0.57 | bottom | 0.001 | KS |
| **Respiratory chain complex** | 55 | 73 | 0.57 | bottom | <0.001 | KS |
| **Respiratory chain complex** | 57 | 78 | 0.55 | bottom | <0.001 | KS |
| **Mixed, incl. Sensory processing of sound by outer hair cells of the cochlea, and Hemolytic anemia** | 41 | 148 | 0.50 | top | <0.001 | KS |
| **Mixed, incl. Contractile fiber, and Microfilament motor activity** | 66 | 185 | 0.46 | bottom | 0.008 | KS |
| **Mixed, incl. Protein targeting to mitochondrion, and Mitochondrial calcium ion transport** | 69 | 104 | 0.45 | bottom | <0.001 | KS |
| **Respiratory electron transport, ATP synthesis by chemiosmotic coupling, and heat production by uncoupling proteins.** | 85 | 121 | 0.45 | bottom | <0.001 | KS |
| **Respiratory chain complex, and Complex I biogenesis** | 70 | 97 | 0.44 | bottom | <0.001 | KS |
| **Citrate cycle (TCA cycle), and Pyruvate metabolism** | 37 | 56 | 0.43 | bottom | 0.004 | KS |
| **Respiratory electron transport, ATP synthesis by chemiosmotic coupling, and heat production by uncoupling proteins., and Proton-transporting ATP synthase complex** | 87 | 126 | 0.40 | bottom | <0.001 | KS |
| **Respiratory electron transport, ATP synthesis by chemiosmotic coupling, and heat production by uncoupling proteins., and Cytochrome complex** | 102 | 160 | 0.38 | bottom | <0.001 | KS |
| **Respiratory electron transport, ATP synthesis by chemiosmotic coupling, and heat production by uncoupling proteins., and Cytochrome complex** | 106 | 169 | 0.37 | bottom | <0.001 | KS |
| **Respiratory electron transport, ATP synthesis by chemiosmotic coupling, and heat production by uncoupling proteins., and Cytochrome complex** | 109 | 180 | 0.37 | bottom | <0.001 | KS |
| **Clathrin coat, and Eps15 homology domain** | 23 | 37 | 0.35 | top | 0.003 | KS |
| **Organellar ribosome, and Regulation of mitochondrial gene expression** | 67 | 115 | 0.35 | bottom | 0.003 | KS |
| **tRNA Aminoacylation** | 27 | 32 | 0.34 | bottom | 0.005 | KS |
| **Organellar large ribosomal subunit** | 37 | 50 | 0.34 | bottom | 0.009 | KS |
| **Clathrin coat, and Presynaptic endocytosis** | 26 | 48 | 0.32 | top | 0.002 | KS |
| **Mitochondrial translation initiation** | 63 | 87 | 0.32 | bottom | 0.004 | KS |
| **Organellar ribosome** | 55 | 77 | 0.30 | bottom | 0.007 | KS |
| **Mixed, incl. Antiviral defense, and Guanylate-binding protein, C-terminal** | 32 | 67 | 0.30 | both ends | 0.01 | KS |
| **Clathrin coat, and Presynaptic endocytosis** | 43 | 85 | 0.28 | top | 0.003 | KS |
| **Mixed, incl. Basic-leucine zipper domain, and MAPK signaling pathway** | 33 | 140 | 0.26 | top | 0.004 | KS |
| **Clathrin coat, and Presynaptic endocytosis** | 38 | 73 | 0.26 | top | 0.005 | KS |
| **Mixed, incl. Membrane coat, and Sec7, C-terminal domain superfamily** | 53 | 117 | 0.26 | top | 0.005 | KS |
|  |  |  |  |  |  |  |

**Supplementary information 20:** Table showing the results of functional enrichment analysis of KEGG pathways performed using the STRING database on all proteins for which there was a log-fold change available between the IOL and elCS group under the relaxed-phase during oxytocin-induced contractions condition (n=6508). The proteins were ranked by their log-fold change. Two statistical methods were used depending on the pathway: the Kolmogorov-Smirnov (KS) test was applied to detect whether proteins associated with each pathway were non-randomly distributed across the ranked list; and the average fold change (AFC) method, which calculates mean log-fold change of proteins within each pathway compared to background. The “Genes mapped” refers to the number of proteins under the relaxed-phase during oxytocin-induced contractions condition found in the pathway, and the “Total genes in set” refers to the total number of proteins within the KEGG pathway; higher enrichment scores indicate stronger enrichment, and the direction of enrichment indicates the direction of enrichment (bottom = downregulated in IOL group, top = upregulated in IOL group; both ends = proteins both upregulated and downregulated); FDR is the false discovery rate, considered significant if ≤0.05.

|  |  |  |  |  |  |  |
| --- | --- | --- | --- | --- | --- | --- |
| **Significantly enriched pathway** | **Genes mapped** | **Total genes in set** | **Enrichment score** | **Direction of enrichment** | **FDR** | **Method** |
| **Metabolism of xenobiotics by cytochrome P450** | 20 | 69 | 1.21 | top | <0.001 | AFC |
| **ECM-receptor interaction** | 41 | 88 | 1.09 | bottom | 0.002 | KS |
| **Systemic lupus erythematosus** | 37 | 94 | 0.78 | bottom | <0.001 | KS |
| **Complement and coagulation cascades** | 49 | 82 | 0.71 | bottom | <0.001 | KS |
| **Proteasome** | 37 | 43 | 0.70 | top | <0.001 | KS |
| **Oxidative phosphorylation** | 82 | 128 | 0.57 | bottom | <0.001 | KS |
| **Staphylococcus aureus infection** | 32 | 86 | 0.56 | both ends | 0.008 | KS |
| **Thermogenesis** | 110 | 226 | 0.52 | bottom | <0.001 | KS |
| **Retrograde endocannabinoid signaling** | 64 | 142 | 0.51 | bottom | <0.001 | KS |
| **Protein digestion and absorption** | 26 | 100 | 0.49 | bottom | <0.001 | KS |
| **Cardiac muscle contraction** | 39 | 87 | 0.42 | bottom | <0.001 | KS |
| **Non-alcoholic fatty liver disease** | 84 | 146 | 0.41 | bottom | <0.001 | KS |
| **Valine, leucine and isoleucine degradation** | 37 | 46 | 0.40 | bottom | <0.001 | KS |
| **Ribosome** | 93 | 131 | 0.39 | bottom | <0.001 | KS |
| **Endocytosis** | 126 | 241 | 0.24 | top | <0.001 | KS |
| **Parkinson disease** | 150 | 236 | 0.16 | bottom | <0.001 | KS |
| **Prion disease** | 167 | 263 | 0.14 | bottom | 0.003 | KS |
|  |  |  |  |  |  |  |

**Supplementary information 21:** Table showing the results of functional enrichment analysis of local network clusters performed using the STRING database on all proteins for which there was a log-fold change available between the IOL and elCS group under the relaxed-phase during oxytocin-induced contractions condition (n=6508). The proteins were ranked by their log-fold change. Two statistical methods were used depending on the cluster: the Kolmogorov-Smirnov (KS) test was applied to detect whether proteins associated with each cluster were non-randomly distributed across the ranked list; and the average fold change (AFC) method, which calculates mean log-fold change of proteins within each cluster compared to background. The “Genes mapped” refers to the number of proteins under the relaxed-phase during oxytocin-induced contractions condition found in the pathway, and the “Total genes in cluster” refers to the total number of proteins within the STRING cluster; higher enrichment scores indicate stronger enrichment, and “Direction of enrichment” indicates the direction of enrichment (bottom = downregulated in IOL group, top = upregulated in IOL group; both ends = proteins both upregulated and downregulated); FDR is the false discovery rate, considered significant if ≤0.05.

|  |  |  |  |  |  |  |
| --- | --- | --- | --- | --- | --- | --- |
| **Significantly enriched cluster** | **Genes mapped** | **total genes in cluster** | **Enrichment score** | **Direction of enrichment** | **FDR** | **Method** |
| **Hemoglobin complex, and Ammonium Transporter Family** | 9 | 21 | 2.39 | top | <0.001 | AFC |
| **Glutathione S-transferase, N-terminal domain, and Benzo(a)pyrene metabolism** | 9 | 17 | 2.33 | top | <0.001 | AFC |
| **Elastic fibre formation, and Tolloid/BMP1 peptidase domain** | 19 | 34 | 1.74 | bottom | <0.001 | AFC |
| **Elastic fibre formation, and Transforming growth factor beta receptor complex assembly** | 16 | 27 | 1.71 | bottom | <0.001 | AFC |
| **Elastic fibre formation** | 10 | 14 | 1.68 | bottom | 0.01 | AFC |
| **Mixed, incl. Hemoglobin complex, and Actin filament capping** | 27 | 49 | 1.48 | top | <0.001 | KS |
| **Elastic fibre formation, and Matrix metalloproteinases** | 28 | 61 | 1.44 | bottom | <0.001 | KS |
| **Collagen chain trimerization** | 19 | 38 | 1.44 | bottom | <0.001 | AFC |
| **Collagen chain trimerization** | 20 | 43 | 1.42 | bottom | <0.001 | AFC |
| **Collagen chain trimerization** | 17 | 32 | 1.40 | bottom | 0.002 | AFC |
| **Mixed, incl. Hemoglobin complex, and Actin filament capping** | 29 | 60 | 1.40 | top | <0.001 | KS |
| **Mixed, incl. Hemoglobin complex, and Actin filament capping** | 30 | 68 | 1.34 | top | <0.001 | KS |
| **Collagen biosynthesis and modifying enzymes** | 23 | 49 | 1.32 | bottom | <0.001 | KS |
| **Spectrin-associated cytoskeleton, and Class II aldolase/adducin N-terminal** | 13 | 16 | 1.26 | top | 0.005 | AFC |
| **Extracellular matrix organization** | 93 | 180 | 1.14 | bottom | <0.001 | KS |
| **Collagen biosynthesis and modifying enzymes** | 31 | 60 | 1.12 | bottom | <0.001 | KS |
| **Mixed, incl. COVID-19, thrombosis and anticoagulation, and Scavenging of heme from plasma** | 23 | 26 | 1.11 | bottom | <0.001 | KS |
| **Spectrin, and Spectrin binding** | 18 | 23 | 1.03 | top | 0.004 | AFC |
| **Collagen formation, and Protein complex involved in cell adhesion** | 65 | 119 | 1.02 | bottom | <0.001 | KS |
| **Complement cascade** | 24 | 40 | 0.88 | bottom | 0.002 | KS |
| **Complement and coagulation cascades, and Positive regulation of opsonization** | 67 | 115 | 0.87 | bottom | <0.001 | KS |
| **Complement and coagulation cascades, and Positive regulation of opsonization** | 63 | 109 | 0.86 | bottom | <0.001 | KS |
| **Complement and coagulation cascades, and Protein-lipid complex** | 87 | 161 | 0.86 | bottom | <0.001 | KS |
| **Hemostasis, and Dissolution of Fibrin Clot** | 33 | 50 | 0.86 | bottom | <0.001 | KS |
| **Complement and coagulation cascades, and Protein-lipid complex** | 88 | 166 | 0.85 | bottom | <0.001 | KS |
| **Mixed, incl. Complement and coagulation cascades, and Protein-lipid complex** | 90 | 172 | 0.85 | bottom | <0.001 | KS |
| **Complement cascade** | 28 | 50 | 0.82 | bottom | 0.001 | KS |
| **Mixed, incl. Sensory processing of sound by outer hair cells of the cochlea, and Hemolytic anemia** | 39 | 148 | 0.81 | top | <0.001 | KS |
| **NADH dehydrogenase (ubiquinone) activity** | 22 | 24 | 0.80 | bottom | <0.001 | KS |
| **NADH dehydrogenase (ubiquinone) activity** | 33 | 42 | 0.79 | bottom | <0.001 | KS |
| **NADH dehydrogenase (ubiquinone) activity** | 31 | 36 | 0.78 | bottom | <0.001 | KS |
| **Respiratory chain complex** | 53 | 73 | 0.75 | bottom | <0.001 | KS |
| **Mixed, incl. Complement and coagulation cascades, and Protein-lipid complex** | 102 | 196 | 0.74 | bottom | <0.001 | KS |
| **Respiratory chain complex** | 55 | 78 | 0.74 | bottom | <0.001 | KS |
| **Mixed, incl. Mitochondrial calcium ion transport, and Miro GTPase Cycle** | 25 | 52 | 0.72 | bottom | 0.004 | KS |
| **Proteasome** | 45 | 53 | 0.69 | top | <0.001 | KS |
| **Proteasome, and Proteasome assembly** | 47 | 59 | 0.67 | top | <0.001 | KS |
| **Proteasome** | 42 | 47 | 0.66 | top | <0.001 | KS |
| **Respiratory electron transport, ATP synthesis by chemiosmotic coupling, and heat production by uncoupling proteins.** | 80 | 121 | 0.65 | bottom | <0.001 | KS |
| **Proteasome** | 38 | 42 | 0.65 | top | <0.001 | KS |
| **Respiratory chain complex, and Complex I biogenesis** | 66 | 97 | 0.65 | bottom | <0.001 | KS |
| **Respiratory electron transport, ATP synthesis by chemiosmotic coupling, and heat production by uncoupling proteins., and Proton-transporting ATP synthase complex** | 81 | 126 | 0.64 | bottom | <0.001 | KS |
| **Mixed, incl. Protein targeting to mitochondrion, and Mitochondrial calcium ion transport** | 60 | 104 | 0.63 | bottom | <0.001 | KS |
| **Respiratory electron transport, ATP synthesis by chemiosmotic coupling, and heat production by uncoupling proteins., and Cytochrome complex** | 98 | 180 | 0.62 | bottom | <0.001 | KS |
| **Respiratory electron transport, ATP synthesis by chemiosmotic coupling, and heat production by uncoupling proteins., and Cytochrome complex** | 96 | 169 | 0.61 | bottom | <0.001 | KS |
| **Oxygen-dependent proline hydroxylation of Hypoxia-inducible Factor Alpha, and Proteasome assembly** | 54 | 81 | 0.61 | top | <0.001 | KS |
| **Respiratory electron transport, ATP synthesis by chemiosmotic coupling, and heat production by uncoupling proteins., and Cytochrome complex** | 94 | 160 | 0.61 | bottom | <0.001 | KS |
| **Oxygen-dependent proline hydroxylation of Hypoxia-inducible Factor Alpha, and Proteasome assembly** | 57 | 88 | 0.60 | top | <0.001 | KS |
| **Organellar ribosome** | 43 | 77 | 0.60 | bottom | <0.001 | KS |
| **Mitochondrial translation initiation** | 49 | 87 | 0.57 | bottom | <0.001 | KS |
| **Mitochondrial protein import, and SAM complex** | 35 | 52 | 0.57 | bottom | <0.001 | KS |
| **Mitochondrial outer membrane translocase complex, and TIM23 mitochondrial import inner membrane translocase complex** | 29 | 42 | 0.57 | bottom | <0.001 | KS |
| **Mitochondrial translation initiation** | 47 | 82 | 0.56 | bottom | <0.001 | KS |
| **Organellar large ribosomal subunit** | 28 | 44 | 0.56 | bottom | <0.001 | KS |
| **Organellar ribosome, and Regulation of mitochondrial gene expression** | 51 | 115 | 0.56 | bottom | <0.001 | KS |
| **Proteasome regulatory particle, and Proteasome alpha-type subunit** | 31 | 31 | 0.55 | top | <0.001 | KS |
| **Organellar large ribosomal subunit** | 29 | 50 | 0.54 | bottom | 0.002 | KS |
| **Organellar large ribosomal subunit** | 21 | 35 | 0.53 | bottom | 0.003 | KS |
| **Mixed, incl. Oxygen-dependent proline hydroxylation of Hypoxia-inducible Factor Alpha, and Ubiquitin conjugating enzyme activity** | 82 | 146 | 0.51 | top | <0.001 | KS |
| **Mixed, incl. Basic-leucine zipper domain, and MAPK signaling pathway** | 27 | 140 | 0.49 | top | <0.001 | KS |
| **Citrate cycle (TCA cycle), and Pyruvate metabolism** | 36 | 56 | 0.48 | bottom | 0.002 | KS |
| **Mixed, incl. Basic-leucine zipper domain, and MAPK signaling pathway** | 28 | 163 | 0.46 | top | 0.001 | KS |
| **Cytoplasmic ribosomal proteins** | 56 | 61 | 0.42 | bottom | <0.001 | KS |
| **Cytoplasmic ribosomal proteins** | 50 | 53 | 0.42 | bottom | <0.001 | KS |
| **Clathrin coat, and Presynaptic endocytosis** | 25 | 48 | 0.41 | top | <0.001 | KS |
| **Cytoplasmic ribosomal proteins** | 43 | 45 | 0.41 | bottom | <0.001 | KS |
| **Clathrin coat, and Eps15 homology domain** | 22 | 37 | 0.40 | top | 0.001 | KS |
| **Cytoplasmic ribosomal proteins** | 61 | 66 | 0.39 | bottom | <0.001 | KS |
| **Viral mRNA Translation** | 62 | 71 | 0.39 | bottom | <0.001 | KS |
| **Viral mRNA Translation** | 64 | 76 | 0.38 | bottom | <0.001 | KS |
| **Cytoplasmic ribosomal proteins** | 38 | 40 | 0.38 | bottom | <0.001 | KS |
| **Viral mRNA Translation** | 65 | 81 | 0.37 | bottom | <0.001 | KS |
| **Clathrin coat, and Presynaptic endocytosis** | 40 | 85 | 0.37 | top | <0.001 | KS |
| **Mixed, incl. Membrane coat, and Sec7, C-terminal domain superfamily** | 49 | 117 | 0.36 | top | <0.001 | KS |
| **Clathrin coat, and Presynaptic endocytosis** | 35 | 73 | 0.36 | top | <0.001 | KS |
| **Viral mRNA Translation** | 66 | 87 | 0.35 | bottom | <0.001 | KS |
| **Viral mRNA Translation, and Sec61 translocon complex** | 75 | 108 | 0.34 | bottom | <0.001 | KS |
| **Eukaryotic Translation Elongation, and Sec61 translocon complex** | 79 | 116 | 0.32 | bottom | <0.001 | KS |
| **Mixed, incl. Endoplasmic reticulum protein-containing complex, and Cotranslational protein targeting to membrane** | 75 | 111 | 0.31 | bottom | <0.001 | KS |
| **Eukaryotic Translation Elongation, and This family consists of several GAGE and XAGE proteins which are found exclusively in humans. The function of this family is unknown although they have been implicated in human cancers (PMID:11992404)** | 82 | 140 | 0.30 | bottom | <0.001 | KS |
| **Eukaryotic Translation Elongation, and This family consists of several GAGE and XAGE proteins which are found exclusively in humans. The function of this family is unknown although they have been implicated in human cancers (PMID:11992404)** | 85 | 150 | 0.29 | bottom | <0.001 | KS |
| **Mixed, incl. Protein targeting to ER, and Oligosaccharyltransferase complex** | 55 | 80 | 0.28 | bottom | <0.001 | KS |
| **Mixed, incl. Eukaryotic Translation Elongation, and This family consists of several GAGE and XAGE proteins which are found exclusively in humans. The function of this family is unknown although they have been implicated in human cancers (PMID:11992404)** | 91 | 163 | 0.27 | bottom | <0.001 | KS |
| **Protein processing in endoplasmic reticulum, and Protein export** | 62 | 90 | 0.27 | bottom | <0.001 | KS |
| **Mixed, incl. Eukaryotic Translation Elongation, and This family consists of several GAGE and XAGE proteins which are found exclusively in humans. The function of this family is unknown although they have been implicated in human cancers (PMID:11992404)** | 93 | 168 | 0.25 | bottom | <0.001 | KS |
| **Mixed, incl. Regulation of actin dynamics for phagocytic cup formation, and CDC42 GTPase cycle** | 93 | 155 | 0.20 | top | 0.005 | KS |
| **Mixed, incl. Actin filament organization, and CDC42 GTPase cycle** | 115 | 193 | 0.18 | top | <0.001 | KS |
|  |  |  |  |  |  |  |

**Supplementary information 22:** Table showing the results of functional enrichment analysis of KEGG pathways performed using the STRING database on all proteins for which there was a log-fold change available between the IOL and elCS group under the contracted-phase during oxytocin-induced contractions condition (n=6507). The proteins were ranked by their log-fold change. Two statistical methods were used depending on the pathway: the Kolmogorov-Smirnov (KS) test was applied to detect whether proteins associated with each pathway were non-randomly distributed across the ranked list; and the average fold change (AFC) method, which calculates mean log-fold change of proteins within each pathway compared to background. The “Genes mapped” refers to the number of proteins under the contracted-phase during oxytocin-induced contractions condition found in the pathway, and the “Total genes in set” refers to the total number of proteins within the KEGG pathway; higher enrichment scores indicate stronger enrichment, and the direction of enrichment indicates the direction of enrichment (bottom = downregulated in IOL group, top = upregulated in IOL group; both ends = proteins both upregulated and downregulated); FDR is the false discovery rate, considered significant if ≤0.05.

|  |  |  |  |  |  |  |
| --- | --- | --- | --- | --- | --- | --- |
| **Significantly enriched pathway** | **Genes mapped** | **Total genes in set** | **Enrichment score** | **Direction of enrichment** | **FDR** | **Method** |
| **Renin-angiotensin system** | 12 | 23 | 1.21 | top | 0.007 | AFC |
| **Metabolism of xenobiotics by cytochrome P450** | 20 | 69 | 1.18 | top | <0.001 | AFC |
| **ECM-receptor interaction** | 41 | 88 | 1.08 | bottom | 0.004 | KS |
| **Protein digestion and absorption** | 26 | 100 | 0.66 | bottom | <0.001 | KS |
| **Systemic lupus erythematosus** | 37 | 94 | 0.65 | bottom | <0.001 | KS |
| **Oxidative phosphorylation** | 82 | 128 | 0.64 | bottom | <0.001 | KS |
| **Thermogenesis** | 110 | 226 | 0.57 | bottom | <0.001 | KS |
| **Proteasome** | 37 | 43 | 0.57 | top | <0.001 | KS |
| **Retrograde endocannabinoid signaling** | 64 | 142 | 0.52 | bottom | <0.001 | KS |
| **Arrhythmogenic right ventricular cardiomyopathy** | 34 | 77 | 0.51 | bottom | 0.001 | KS |
| **Valine, leucine and isoleucine degradation** | 37 | 46 | 0.48 | bottom | 0.001 | KS |
| **Non-alcoholic fatty liver disease** | 84 | 146 | 0.45 | bottom | <0.001 | KS |
| **Cardiac muscle contraction** | 39 | 87 | 0.40 | bottom | <0.001 | KS |
| **Propanoate metabolism** | 27 | 33 | 0.40 | bottom | 0.003 | KS |
| **Dilated cardiomyopathy** | 39 | 94 | 0.26 | bottom | 0.003 | KS |
| **Epstein-Barr virus infection** | 84 | 192 | 0.23 | top | 0.006 | KS |
| **Parkinson disease** | 150 | 236 | 0.23 | bottom | <0.001 | KS |
| **Carbon metabolism** | 74 | 116 | 0.19 | bottom | 0.002 | KS |
| **Endocytosis** | 126 | 241 | 0.18 | top | <0.001 | KS |
| **Huntington disease** | 161 | 295 | 0.16 | bottom | <0.001 | KS |
| **Prion disease** | 167 | 263 | 0.16 | bottom | 0.005 | KS |
| **Alzheimer disease** | 188 | 354 | 0.14 | bottom | 0.005 | KS |
|  |  |  |  |  |  |  |

**Supplementary information 23:** Table showing the results of functional enrichment analysis of local network clusters performed using the STRING database on all proteins for which there was a log-fold change available between the IOL and elCS group under the contracted-phase during oxytocin-induced contractions condition (n=6507). The proteins were ranked by their log-fold change. Two statistical methods were used depending on the cluster: the Kolmogorov-Smirnov (KS) test was applied to detect whether proteins associated with each cluster were non-randomly distributed across the ranked list; and the average fold change (AFC) method, which calculates mean log-fold change of proteins within each cluster compared to background. The “Genes mapped” refers to the number of proteins under the contracted-phase during oxytocin-induced contractions condition found in the pathway, and the “Total genes in cluster” refers to the total number of proteins within the STRING cluster; higher enrichment scores indicate stronger enrichment, and “Direction of enrichment” indicates the direction of enrichment (bottom = downregulated in IOL group, top = upregulated in IOL group; both ends = proteins both upregulated and downregulated); FDR is the false discovery rate, considered significant if ≤0.05.

| **Significantly enriched cluster** | **Genes mapped** | **total genes in cluster** | **Enrichment score** | **Direction of enrichment** | **FDR** | **Method** |
| --- | --- | --- | --- | --- | --- | --- |
| **Hemoglobin complex, and Ammonium Transporter Family** | 9 | 21 | 2.49 | top | <0.001 | AFC |
| **Glutathione S-transferase, N-terminal domain, and Benzo(a)pyrene metabolism** | 9 | 17 | 2.04 | top | <0.001 | AFC |
| **Banded collagen fibril, and FACIT collagen trimer** | 10 | 18 | 2.01 | bottom | 0.001 | AFC |
| **Elastic fibre formation** | 10 | 14 | 1.80 | bottom | 0.004 | AFC |
| **Elastic fibre formation, and Transforming growth factor beta receptor complex assembly** | 16 | 27 | 1.76 | bottom | <0.001 | KS |
| **Elastic fibre formation, and Tolloid/BMP1 peptidase domain** | 19 | 34 | 1.75 | bottom | <0.001 | KS |
| **Collagen chain trimerization** | 19 | 38 | 1.74 | bottom | <0.001 | AFC |
| **Collagen chain trimerization** | 17 | 32 | 1.71 | bottom | <0.001 | AFC |
| **Collagen chain trimerization** | 20 | 43 | 1.68 | bottom | <0.001 | AFC |
| **Collagen biosynthesis and modifying enzymes** | 23 | 49 | 1.61 | bottom | <0.001 | KS |
| **Mixed, incl. Hemoglobin complex, and Actin filament capping** | 27 | 49 | 1.43 | top | <0.001 | KS |
| **Elastic fibre formation, and Matrix metalloproteinases** | 28 | 61 | 1.37 | bottom | <0.001 | KS |
| **Mixed, incl. Hemoglobin complex, and Actin filament capping** | 29 | 60 | 1.36 | top | <0.001 | KS |
| **Collagen biosynthesis and modifying enzymes** | 31 | 60 | 1.31 | bottom | <0.001 | KS |
| **Mixed, incl. Hemoglobin complex, and Actin filament capping** | 30 | 68 | 1.30 | top | <0.001 | KS |
| **Extracellular matrix organization** | 93 | 180 | 1.16 | bottom | <0.001 | KS |
| **Collagen formation, and Protein complex involved in cell adhesion** | 65 | 119 | 1.08 | bottom | <0.001 | KS |
| **NADH dehydrogenase (ubiquinone) activity** | 22 | 24 | 0.87 | bottom | <0.001 | KS |
| **NADH dehydrogenase (ubiquinone) activity** | 33 | 42 | 0.86 | bottom | <0.001 | KS |
| **Respiratory chain complex** | 53 | 73 | 0.85 | bottom | <0.001 | KS |
| **NADH dehydrogenase (ubiquinone) activity** | 31 | 36 | 0.84 | bottom | <0.001 | KS |
| **Respiratory chain complex** | 55 | 78 | 0.83 | bottom | <0.001 | KS |
| **Mixed, incl. Sensory processing of sound by outer hair cells of the cochlea, and Hemolytic anemia** | 39 | 148 | 0.81 | top | <0.001 | KS |
| **Respiratory chain complex, and Complex I biogenesis** | 66 | 97 | 0.75 | bottom | <0.001 | KS |
| **Respiratory electron transport, ATP synthesis by chemiosmotic coupling, and heat production by uncoupling proteins.** | 80 | 121 | 0.73 | bottom | <0.001 | KS |
| **Respiratory electron transport, ATP synthesis by chemiosmotic coupling, and heat production by uncoupling proteins., and Proton-transporting ATP synthase complex** | 81 | 126 | 0.72 | bottom | <0.001 | KS |
| **Respiratory electron transport, ATP synthesis by chemiosmotic coupling, and heat production by uncoupling proteins., and Cytochrome complex** | 94 | 160 | 0.65 | bottom | <0.001 | KS |
| **Respiratory electron transport, ATP synthesis by chemiosmotic coupling, and heat production by uncoupling proteins., and Cytochrome complex** | 96 | 169 | 0.63 | bottom | <0.001 | KS |
| **Respiratory electron transport, ATP synthesis by chemiosmotic coupling, and heat production by uncoupling proteins., and Cytochrome complex** | 98 | 180 | 0.63 | bottom | <0.001 | KS |
| **Citrate cycle (TCA cycle), and Pyruvate metabolism** | 36 | 56 | 0.62 | bottom | <0.001 | KS |
| **Mixed, incl. Mitochondrial calcium ion transport, and Miro GTPase Cycle** | 25 | 52 | 0.61 | bottom | 0.009 | KS |
| **Mixed, incl. Protein targeting to mitochondrion, and Mitochondrial calcium ion transport** | 60 | 104 | 0.58 | bottom | <0.001 | KS |
| **Mitochondrial protein import, and SAM complex** | 35 | 52 | 0.56 | bottom | <0.001 | KS |
| **Proteasome** | 45 | 53 | 0.54 | top | <0.001 | KS |
| **Proteasome, and Proteasome assembly** | 47 | 59 | 0.53 | top | <0.001 | KS |
| **Muscle protein, and Myofibril assembly** | 22 | 79 | 0.53 | bottom | 0.004 | KS |
| **Mitochondrial outer membrane translocase complex, and TIM23 mitochondrial import inner membrane translocase complex** | 29 | 42 | 0.52 | bottom | <0.001 | KS |
| **Proteasome** | 42 | 47 | 0.52 | top | <0.001 | KS |
| **Organellar large ribosomal subunit** | 21 | 35 | 0.51 | bottom | <0.001 | KS |
| **Proteasome** | 38 | 42 | 0.51 | top | <0.001 | KS |
| **Oxygen-dependent proline hydroxylation of Hypoxia-inducible Factor Alpha, and Proteasome assembly** | 54 | 81 | 0.50 | top | <0.001 | KS |
| **Citrate cycle (TCA cycle), and Lactate dehydrogenase activity** | 30 | 46 | 0.49 | bottom | 0.003 | KS |
| **Mixed, incl. Muscle protein, and Sarcoplasmic reticulum membrane** | 31 | 126 | 0.48 | bottom | 0.007 | KS |
| **Organellar ribosome** | 43 | 77 | 0.47 | bottom | <0.001 | KS |
| **Organellar large ribosomal subunit** | 28 | 44 | 0.47 | bottom | <0.001 | KS |
| **Mitochondrial translation initiation** | 49 | 87 | 0.47 | bottom | <0.001 | KS |
| **Mitochondrial translation initiation** | 47 | 82 | 0.47 | bottom | <0.001 | KS |
| **Organellar large ribosomal subunit** | 29 | 50 | 0.46 | bottom | <0.001 | KS |
| **Organellar ribosome, and Regulation of mitochondrial gene expression** | 51 | 115 | 0.46 | bottom | <0.001 | KS |
| **Oxygen-dependent proline hydroxylation of Hypoxia-inducible Factor Alpha, and Proteasome assembly** | 57 | 88 | 0.46 | top | <0.001 | KS |
| **Mixed, incl. Muscle protein, and Sarcoplasmic reticulum membrane** | 33 | 139 | 0.43 | bottom | 0.007 | KS |
| **Mixed, incl. Basic-leucine zipper domain, and MAPK signaling pathway** | 27 | 140 | 0.42 | top | 0.006 | KS |
| **Mixed, incl. Basic-leucine zipper domain, and MAPK signaling pathway** | 28 | 163 | 0.41 | top | 0.009 | KS |
| **Proteasome regulatory particle, and Proteasome alpha-type subunit** | 31 | 31 | 0.40 | top | <0.001 | KS |
| **Mixed, incl. Oxygen-dependent proline hydroxylation of Hypoxia-inducible Factor Alpha, and Ubiquitin conjugating enzyme activity** | 82 | 146 | 0.37 | top | <0.001 | KS |
| **Fatty acid beta-oxidation, and Valine catabolic process** | 32 | 35 | 0.37 | bottom | 0.008 | KS |
| **Clathrin coat, and Presynaptic endocytosis** | 25 | 48 | 0.34 | top | 0.01 | KS |
| **Fatty acid beta-oxidation, and Valine, leucine and isoleucine degradation** | 48 | 65 | 0.33 | bottom | 0.002 | KS |
| **Mixed, incl. Membrane coat, and Sec7, C-terminal domain superfamily** | 49 | 117 | 0.31 | top | 0.002 | KS |
| **Fatty acid beta-oxidation, and Valine, leucine and isoleucine degradation** | 49 | 75 | 0.31 | bottom | 0.003 | KS |
| **Clathrin coat, and Presynaptic endocytosis** | 40 | 85 | 0.30 | top | 0.007 | KS |
| **Clathrin coat, and Presynaptic endocytosis** | 35 | 73 | 0.29 | top | 0.006 | KS |
| **Mixed, incl. Cul4-RING E3 ubiquitin ligase complex, and Protein neddylation** | 38 | 85 | 0.28 | top | 0.004 | KS |
| **cullin-RING ubiquitin ligase complex, and Protein neddylation** | 58 | 187 | 0.22 | top | 0.004 | KS |
| **Initiation factor** | 35 | 43 | 0.22 | top | 0.007 | KS |
| **Neddylation, and Cul3-RING ubiquitin ligase complex** | 49 | 122 | 0.21 | top | 0.009 | KS |
| **Mixed, incl. Endoplasmic reticulum protein-containing complex, and Cotranslational protein targeting to membrane** | 75 | 111 | 0.21 | bottom | <0.001 | KS |
| **Protein processing in endoplasmic reticulum, and Protein export** | 62 | 90 | 0.18 | bottom | 0.007 | KS |
| **Mixed, incl. Protein targeting to ER, and Oligosaccharyltransferase complex** | 55 | 80 | 0.18 | bottom | 0.009 | KS |
| **Mixed, incl. Actin filament organization, and CDC42 GTPase cycle** | 115 | 193 | 0.11 | top | 0.009 | KS |
|  |  |  |  |  |  |  |

**Supplementary information 24:** Table showing the results of functional enrichment analysis of KEGG pathways performed using the STRING database on all proteins for which there was a log-fold change available between the IOL and elCS group under all experimental conditions and for which a KEGG pathway was shared between at least two conditions and the FDR was significant (≤0.05). PRE = pre-contracting; SP-REL = relaxed-phase during spontaneous contractions; OXT-REL = relaxed-phase during oxytocin-induced contractions; SP-CON = contracted-phase during spontaneous contractions; OXT-CON = contracted-phase during oxytocin-induced contractions.

|  |  |  |  |  |  |
| --- | --- | --- | --- | --- | --- |
| **KEGG pathway** | **PRE** | **SP-REL** | **SP-CON** | **OXT-REL** | **OXT-CON** |
| **ECM-receptor interaction** | ✓ | ✓ | ✓ | ✓ | ✓ |
| **Non-alcoholic fatty liver disease** | ✓ | ✓ | ✓ | ✓ | ✓ |
| **Oxidative phosphorylation** | ✓ | ✓ | ✓ | ✓ | ✓ |
| **Parkinson disease** | ✓ | ✓ | ✓ | ✓ | ✓ |
| **Prion disease** | ✓ | ✓ | ✓ | ✓ | ✓ |
| **Protein digestion and absorption** | ✓ | ✓ | ✓ | ✓ | ✓ |
| **Retrograde endocannabinoid signalling** | ✓ | ✓ | ✓ | ✓ | ✓ |
| **Systemic lupus erythematosus** | ✓ | ✓ | ✓ | ✓ | ✓ |
| **Thermogenesis** | ✓ | ✓ | ✓ | ✓ | ✓ |
| **Complement and coagulation cascades** | ✓ | ✓ | ✓ | ✓ |  |
| **Staphylococcus aureus infection** | ✓ | ✓ | ✓ | ✓ |  |
| **Amoebiasis** | ✓ | ✓ | ✓ |  |  |
| **Citrate cycle (TCA cycle)** | ✓ | ✓ | ✓ |  |  |
| **Endocytosis** | ✓ | ✓ |  | ✓ | ✓ |
| **Carbon metabolism** | ✓ |  |  |  | ✓ |
| **Aminoacyl-tRNA biosynthesis** | ✓ |  |  |  |  |
| **Spliceosome** | ✓ |  |  |  |  |
| **Cardiac muscle contraction** |  | ✓ | ✓ | ✓ | ✓ |
| **Huntington disease** |  | ✓ | ✓ |  | ✓ |
| **Proteasome** |  | ✓ |  | ✓ | ✓ |
| **Ribosome** |  | ✓ |  | ✓ |  |
| **Arrhythmogenic right ventricular cardiomyopathy** |  | ✓ |  |  | ✓ |
| **Dilated cardiomyopathy** |  | ✓ |  |  | ✓ |
| **Drug metabolism - cytochrome P450** |  | ✓ |  |  |  |
| **Hypertrophic cardiomyopathy** |  | ✓ |  |  |  |
| **Metabolism of xenobiotics by cytochrome P450** |  |  |  | ✓ | ✓ |
| **Valine, leucine and isoleucine degradation** |  |  |  | ✓ | ✓ |
| **Alzheimer disease** |  |  |  |  | ✓ |
| **Epstein-Barr virus infection** |  |  |  |  | ✓ |
| **Propanoate metabolism** |  |  |  |  | ✓ |
| **Renin-angiotensin system** |  |  |  |  | ✓ |
|  |  |  |  |  |  |

**Supplementary information 25:** Table showing the results of functional enrichment analysis of local network clusters performed using the STRING database on all proteins for which there was a log-fold change available between the IOL and elCS group under all experimental conditions and for which a network cluster was shared between at least two conditions and the FDR was significant (≤0.05). PRE = pre-contracting; SP-REL = relaxed-phase during spontaneous contractions; OXT-REL = relaxed-phase during oxytocin-induced contractions; SP-CON = contracted-phase during spontaneous contractions; OXT-CON = contracted-phase during oxytocin-induced contractions.

| **Cluster** | **PRE** | **SP-REL** | **SP-CON** | **OXT-REL** | **OXT-CON** |
| --- | --- | --- | --- | --- | --- |
| **Citrate cycle (TCA cycle), and Pyruvate metabolism** | ✓ | ✓ | ✓ | ✓ | ✓ |
| **Clathrin coat, and Presynaptic endocytosis** | ✓ | ✓ | ✓ | ✓ | ✓ |
| **Elastic fibre formation, and Matrix metalloproteinases** | ✓ | ✓ | ✓ | ✓ | ✓ |
| **Elastic fibre formation, and Tolloid/BMP1 peptidase domain** | ✓ | ✓ | ✓ | ✓ | ✓ |
| **Extracellular matrix organization** | ✓ | ✓ | ✓ | ✓ | ✓ |
| **Mixed, incl. Membrane coat, and Sec7, C-terminal domain superfamily** | ✓ | ✓ | ✓ | ✓ | ✓ |
| **Mixed, incl. Protein targeting to mitochondrion, and Mitochondrial calcium ion transport** | ✓ | ✓ | ✓ | ✓ | ✓ |
| **NADH dehydrogenase (ubiquinone) activity** | ✓ | ✓ | ✓ | ✓ | ✓ |
| **Respiratory chain complex** | ✓ | ✓ | ✓ | ✓ | ✓ |
| **Respiratory chain complex, and Complex I biogenesis** | ✓ | ✓ | ✓ | ✓ | ✓ |
| **Respiratory electron transport, ATP synthesis by chemiosmotic coupling, and heat production by uncoupling proteins.** | ✓ | ✓ | ✓ | ✓ | ✓ |
| **Respiratory electron transport, ATP synthesis by chemiosmotic coupling, and heat production by uncoupling proteins., and Cytochrome complex** | ✓ | ✓ | ✓ | ✓ | ✓ |
| **Respiratory electron transport, ATP synthesis by chemiosmotic coupling, and heat production by uncoupling proteins., and Proton-transporting ATP synthase complex** | ✓ | ✓ | ✓ | ✓ | ✓ |
| **Collagen biosynthesis and modifying enzymes** | ✓ | ✓ | ✓ | ✓ |  |
| **Collagen chain trimerization** | ✓ | ✓ | ✓ | ✓ |  |
| **Complement and coagulation cascades, and Positive regulation of opsonization** | ✓ | ✓ | ✓ | ✓ |  |
| **Complement and coagulation cascades, and Protein-lipid complex** | ✓ | ✓ | ✓ | ✓ |  |
| **Complement cascade** | ✓ | ✓ | ✓ | ✓ |  |
| **Hemostasis, and Dissolution of Fibrin Clot** | ✓ | ✓ | ✓ | ✓ |  |
| **Mixed, incl. Complement and coagulation cascades, and Protein-lipid complex** | ✓ | ✓ | ✓ | ✓ |  |
| **Mixed, incl. COVID-19, thrombosis and anticoagulation, and Scavenging of heme from plasma** | ✓ | ✓ | ✓ | ✓ |  |
| **Mixed, incl. Muscle protein, and Sarcoplasmic reticulum membrane** | ✓ | ✓ | ✓ |  | ✓ |
| **Muscle protein, and Myofibril assembly** | ✓ | ✓ | ✓ |  | ✓ |
| **Collagen formation, and Protein complex involved in cell adhesion** | ✓ | ✓ | ✓ |  |  |
| **Muscle protein, and Myosin light chain kinase activity** | ✓ | ✓ | ✓ |  |  |
| **Muscle protein, and Sarcomere organization** | ✓ | ✓ | ✓ |  |  |
| **Muscle protein, and Sarcoplasmic reticulum membrane** | ✓ | ✓ | ✓ |  |  |
| **Mixed, incl. COVID-19, thrombosis and anticoagulation, and Inter-alpha-trypsin inhibitor heavy chain C-terminus** | ✓ | ✓ |  |  |  |
| **tRNA Aminoacylation** | ✓ |  | ✓ |  |  |
| **Citrate cycle (TCA cycle), and Lactate dehydrogenase activity** | ✓ |  |  |  | ✓ |
| **Alpha-1-acid glycoprotein, and Protein-losing enteropathy** | ✓ |  |  |  |  |
| **Carbon metabolism, and Pyruvate metabolism** | ✓ |  |  |  |  |
| **Formation of the cornified envelope, and Autosomal recessive congenital ichthyosis** | ✓ |  |  |  |  |
| **Initial triggering of complement, and Regulation of complement activation** | ✓ |  |  |  |  |
| **Mixed, incl. Chemokine-mediated signaling pathway, and Adaptive immunity** | ✓ |  |  |  |  |
| **Mixed, incl. Inter-alpha-trypsin inhibitor heavy chain C-terminus, and Alpha-1-acid glycoprotein** | ✓ |  |  |  |  |
| **mRNA processing, and RNA recognition motif domain** | ✓ |  |  |  |  |
| **Precatalytic spliceosome** | ✓ |  |  |  |  |
| **Precatalytic spliceosome, and Renpenning syndrome** | ✓ |  |  |  |  |
| **Precatalytic spliceosome, and U4/U6 x U5 tri-snRNP complex** | ✓ |  |  |  |  |
| **Spliceosomal snRNP complex, and mRNA cis splicing, via spliceosome** | ✓ |  |  |  |  |
| **Striated muscle contraction pathway, and Myosin II complex** | ✓ |  |  |  |  |
| **tRNA Aminoacylation, and glutamyl-tRNA(Gln) amidotransferase complex** | ✓ |  |  |  |  |
| **U2-type spliceosomal complex, and mRNA Splicing - Major Pathway** | ✓ |  |  |  |  |
| **U2-type spliceosomal complex, and Sm-like protein family complex** | ✓ |  |  |  |  |
| **Elastic fibre formation, and Transforming growth factor beta receptor complex assembly** |  | ✓ | ✓ | ✓ | ✓ |
| **Glutathione S-transferase, N-terminal domain, and Benzo(a)pyrene metabolism** |  | ✓ | ✓ | ✓ | ✓ |
| **Mitochondrial translation initiation** |  | ✓ | ✓ | ✓ | ✓ |
| **Mixed, incl. Hemoglobin complex, and Actin filament capping** |  | ✓ | ✓ | ✓ | ✓ |
| **Mixed, incl. Mitochondrial calcium ion transport, and Miro GTPase Cycle** |  | ✓ | ✓ | ✓ | ✓ |
| **Mixed, incl. Sensory processing of sound by outer hair cells of the cochlea, and Hemolytic anemia** |  | ✓ | ✓ | ✓ | ✓ |
| **Organellar large ribosomal subunit** |  | ✓ | ✓ | ✓ | ✓ |
| **Organellar ribosome** |  | ✓ | ✓ | ✓ | ✓ |
| **Organellar ribosome, and Regulation of mitochondrial gene expression** |  | ✓ | ✓ | ✓ | ✓ |
| **Clathrin coat, and Eps15 homology domain** |  | ✓ | ✓ | ✓ |  |
| **Mixed, incl. Eukaryotic Translation Elongation, and This family consists of several GAGE and XAGE proteins which are found exclusively in humans. The function of this family is unknown although they have been implicated in human cancers (PMID:11992404)** |  | ✓ | ✓ | ✓ |  |
| **Mixed, incl. Contractile fiber, and Microfilament motor activity** |  | ✓ | ✓ |  |  |
| **Elastic fibre formation** |  | ✓ |  | ✓ | ✓ |
| **Mitochondrial outer membrane translocase complex, and TIM23 mitochondrial import inner membrane translocase complex** |  | ✓ |  | ✓ | ✓ |
| **Mitochondrial protein import, and SAM complex** |  | ✓ |  | ✓ | ✓ |
| **Mixed, incl. Oxygen-dependent proline hydroxylation of Hypoxia-inducible Factor Alpha, and Ubiquitin conjugating enzyme activity** |  | ✓ |  | ✓ | ✓ |
| **Mixed, incl. Protein targeting to ER, and Oligosaccharyltransferase complex** |  | ✓ |  | ✓ | ✓ |
| **Oxygen-dependent proline hydroxylation of Hypoxia-inducible Factor Alpha, and Proteasome assembly** |  | ✓ |  | ✓ | ✓ |
| **Proteasome** |  | ✓ |  | ✓ | ✓ |
| **Cytoplasmic ribosomal proteins** |  | ✓ |  | ✓ |  |
| **Eukaryotic Translation Elongation, and Sec61 translocon complex** |  | ✓ |  | ✓ |  |
| **Eukaryotic Translation Elongation, and This family consists of several GAGE and XAGE proteins which are found exclusively in humans. The function of this family is unknown although they have been implicated in human cancers (PMID:11992404)** |  | ✓ |  | ✓ |  |
| **Viral mRNA Translation** |  | ✓ |  | ✓ |  |
| **Viral mRNA Translation, and Sec61 translocon complex** |  | ✓ |  | ✓ |  |
| **Proteasome, and Proteasome assembly** |  | ✓ |  |  |  |
| **Protein complex involved in cell adhesion, and Junctional epidermolysis bullosa** |  | ✓ |  |  |  |
| **Mixed, incl. Basic-leucine zipper domain, and MAPK signaling pathway** |  |  | ✓ | ✓ | ✓ |
| **Mixed, incl. Antiviral defense, and Guanylate-binding protein, C-terminal** |  |  | ✓ |  |  |
| **Mixed, incl. Intermediate filament head, DNA-binding domain, and Oligodendrocyte specification and differentiation, leading to myelin components for CNS** |  |  | ✓ |  |  |
| **Type III transforming growth factor beta receptor binding, and Transforming growth factor beta receptor complex assembly** |  |  | ✓ |  |  |
| **Hemoglobin complex, and Ammonium Transporter Family** |  |  |  | ✓ | ✓ |
| **Mixed, incl. Actin filament organization, and CDC42 GTPase cycle** |  |  |  | ✓ | ✓ |
| **Mixed, incl. Endoplasmic reticulum protein-containing complex, and Cotranslational protein targeting to membrane** |  |  |  | ✓ | ✓ |
| **Proteasome regulatory particle, and Proteasome alpha-type subunit** |  |  |  | ✓ | ✓ |
| **Proteasome, and Proteasome assembly** |  |  |  | ✓ | ✓ |
| **Protein processing in endoplasmic reticulum, and Protein export** |  |  |  | ✓ | ✓ |
| **Mixed, incl. Regulation of actin dynamics for phagocytic cup formation, and CDC42 GTPase cycle** |  |  |  | ✓ |  |
| **Spectrin-associated cytoskeleton, and Class II aldolase/adducin N-terminal** |  |  |  | ✓ |  |
| **Spectrin, and Spectrin binding** |  |  |  | ✓ |  |
| **Banded collagen fibril, and FACIT collagen trimer** |  |  |  |  | ✓ |
| **Collagen formation, and Protein complex involved in cell adhesion** |  |  |  |  | ✓ |
| **cullin-RING ubiquitin ligase complex, and Protein neddylation** |  |  |  |  | ✓ |
| **Fatty acid beta-oxidation, and Valine catabolic process** |  |  |  |  | ✓ |
| **Fatty acid beta-oxidation, and Valine, leucine and isoleucine degradation** |  |  |  |  | ✓ |
| **Initiation factor** |  |  |  |  | ✓ |
| **Mixed, incl. Cul4-RING E3 ubiquitin ligase complex, and Protein neddylation** |  |  |  |  | ✓ |
| **Neddylation, and Cul3-RING ubiquitin ligase complex** |  |  |  |  | ✓ |
|  |  |  |  |  |  |

Supplementary Information 26: Phosphopeptides that significantly (p≤0.05) at least halved and passed FDR (q≤0.05) in the failed IOL group under the pre-contracting condition.

|  |  |  |  |  |  |  |  |
| --- | --- | --- | --- | --- | --- | --- | --- |
| **Protein** | **Accession** | **Gene name** | **Sequence detected** | **Site** | **log 2 fc** | **p value** | **q value** |
| **Nexilin (F-actin-binding protein) (Nelin)** | Q0ZGT2 | NEXN | ESLSPGK | 1xPhospho [S179(100)] | -3.76 | <0.001 | 0.010 |
| **Protein prune homolog 2 (BNIP2 motif-containing molecule at the C-terminal region 1)** | D6RTK6 | PRUNE2 | RASDSVFQPK | 1xPhospho [S699(100)] | -3.57 | <0.001 | 0.010 |
|  |  |  |  |  |  |  |  |

Supplementary Information 27: Phosphopeptides that significantly (p≤0.05) at least doubled and passed FDR (q≤0.05) in the failed IOL group under the relaxed-phase during spontaneous contractions condition.

|  |  |  |  |  |  |  |  |
| --- | --- | --- | --- | --- | --- | --- | --- |
| **Protein** | **Accession** | **Gene name** | **Sequence detected** | **Site** | **log 2 fc** | **p value** | **q value** |
| **Filamin-A (FLN-A) (Actin-binding protein 280) (ABP-280) (Alpha-filamin) (Endothelial actin-binding protein) (Filamin-1) (Non-muscle filamin)** | P21333 | FLNA | SPFSVAVSPSLDLSK | 1xPhospho [S968(99.8)] | 1.76 | <0.001 | 0.003 |
| **Filamin-A (FLN-A) (Actin-binding protein 280) (ABP-280) (Alpha-filamin) (Endothelial actin-binding protein) (Filamin-1) (Non-muscle filamin)** | P21333 | FLNA | IPEISIQDMTAQVTSPSGK | 1xPhospho [S2180(99.6)] | 1.40 | <0.001 | 0.021 |
|  |  |  |  |  |  |  |  |

Supplementary Information 28: Phosphopeptides that significantly (p≤0.05) at least halved and passed FDR (q≤0.05) in the failed IOL group under the relaxed-phase during spontaneous contractions condition.

|  |  |  |  |  |  |  |  |
| --- | --- | --- | --- | --- | --- | --- | --- |
| **Protein** | **Accession** | **Gene name** | **Sequence detected** | **Site** | **log 2 fc** | **p value** | **q value** |
| **Nexilin (F-actin-binding protein) (Nelin)** | Q0ZGT2 | NEXN | ESLSPGK | 1xPhospho [S179(100)] | -3.62 | <0.001 | 0.005 |
|  |  |  |  |  |  |  |  |

Supplementary Information 29: Phosphopeptides that significantly (p≤0.05) at least doubled and passed FDR (q≤0.05) in the failed IOL group under the contracted-phase during spontaneous contractions condition.

|  |  |  |  |  |  |  |  |
| --- | --- | --- | --- | --- | --- | --- | --- |
| **Protein** | **Accession** | **Gene name** | **Sequence detected** | **Site** | **log 2 fc** | **p value** | **q value** |
| **Monocarboxylate transporter 1 (MCT 1) (Solute carrier family 16 member 1)** | P53985 | SLC16A1 | KESKEEETSIDVAGKPNEVTK | 2xPhospho [S461(99.8); S467(89.3)] | 1.9 | <0.001 | 0.048 |
|  |  |  |  |  |  |  |  |

Supplementary Information 30: Phosphopeptides that significantly (p≤0.05) at least halved and passed FDR (q≤0.05) in the failed IOL group under the contracted-phase during spontaneous contractions condition.

|  |  |  |  |  |  |  |  |
| --- | --- | --- | --- | --- | --- | --- | --- |
| **Protein** | **Accession** | **Gene name** | **Sequence detected** | **Site** | **log 2 fc** | **p value** | **q value** |
| **Nexilin (F-actin-binding protein) (Nelin)** | Q0ZGT2 | NEXN | ESLSPGK | 1xPhospho [S179(100)] | -3.62 | <0.001 | 0.013 |
| **Nuclear mitotic apparatus protein 1 (Nuclear matrix protein-22) (NMP-22) (Nuclear mitotic apparatus protein) (NuMA protein) (SP-H antigen)** | Q14980 | NUMA1 | TQPDGTSVPGEPASPISQR | 1xPhospho [S1763(99)] | -2.37 | <0.001 | 0.048 |
| **Beta-2-syntrophin (59 kDa dystrophin-associated protein A1 basic component 2) (Syntrophin-3) (SNT3) (Syntrophin-like) (SNTL)** | Q13425 | SNTB2 | GPAGEAGASPPVRR | 1xPhospho [S110(100)] | -2.28 | <0.001 | 0.048 |
| **Serine/threonine-protein kinase 10 (EC 2.7.11.1) (Lymphocyte-oriented kinase)** | O94804 | STK10 | QVAEQGGDLSPAANR | 1xPhospho [S438(100)] | -2.25 | <0.001 | 0.048 |
|  |  |  |  |  |  |  |  |

Supplementary Information 31: Phosphopeptides that significantly (p≤0.05) at least doubled and passed FDR (q≤0.05) in the failed IOL group under the relaxed-phase during oxytocin-induced contractions condition.

|  |  |  |  |  |  |  |  |
| --- | --- | --- | --- | --- | --- | --- | --- |
| **Protein** | **Accession** | **Gene name** | **Sequence detected** | **Site** | **log 2 fc** | **p value** | **q value** |
| **Synaptopodin 2** | B9EG60 | SYNPO2 | GTGAGGDSGPEEDYLSLGAEACNFMQSSSAK | 1xPhospho [Y735(97.9)] | 2.01 | <0.001 | 0.021 |
|  |  |  |  |  |  |  |  |

Supplementary Information 32: Phosphopeptides that significantly (p≤0.05) at least halved and passed FDR (q≤0.05) in the failed IOL group under the relaxed-phase during oxytocin-induced contractions condition.

|  |  |  |  |  |  |  |  |
| --- | --- | --- | --- | --- | --- | --- | --- |
| **Protein** | **Accession** | **Gene name** | **Sequence detected** | **Site** | **log 2 fc** | **p value** | **q value** |
| **Nexilin (F-actin-binding protein) (Nelin)** | Q0ZGT2 | NEXN | ESLSPGK | 1xPhospho [S179(100)] | -4.32 | <0.001 | <0.001 |
| **Chondroitin sulfate proteoglycan 2 (Versican), isoform CRA_c OS=Homo sapiens** | A0A024RAL1 | CSPG2 | TDGQVSGEAIK | 1xPhospho  [S2955(100)] | -4.33 | <0.001 | 0.021 |
|  |  |  |  |  |  |  |  |

Supplementary Information 33: Phosphopeptides that significantly (p≤0.05) at least doubled and passed FDR (q≤0.05) in the failed IOL group under the contracted-phase during oxytocin-induced contractions condition.

|  |  |  |  |  |  |  |  |
| --- | --- | --- | --- | --- | --- | --- | --- |
| **Protein** | **Accession** | **Gene name** | **Sequence detected** | **Site** | **log 2 fc** | **p value** | **q value** |
| **Protein transport protein Sec31A (SEC31-like protein 1) (SEC31-related protein A)** | D6REX3 | SEC31A | AQGEPVAGHESPK | 1xPhospho [S799(100)] | 1.40 | <0.001 | 0.041 |
|  |  |  |  |  |  |  |  |

Supplementary Information 34: Phosphopeptides that significantly (p≤0.05) at least halved and passed FDR (q≤0.05) in the failed IOL group under the contracted-phase during oxytocin-induced contractions condition.

|  |  |  |  |  |  |  |  |
| --- | --- | --- | --- | --- | --- | --- | --- |
| **Protein** | **Accession** | **Gene name** | **Sequence detected** | **Site** | **log 2 fc** | **p value** | **q value** |
| **Nexilin (F-actin-binding protein) (Nelin)** | Q0ZGT2 | NEXN | ESLSPGK | 1xPhospho [S179(100)] | -3.70 | <0.001 | 0.001 |

**Supplementary Information 35:** Total proteins for which the ratio between the contracted-phase during spontaneous contractions condition and the pre-contracting condition (contracted-phase during spontaneous contractions /pre-contracting) significantly (p≤0.05) at least halved and passed FDR (q≤0.05) in the elective caesarean section group.

|  |  |  |  |  |  |  |
| --- | --- | --- | --- | --- | --- | --- |
| **Protein name** | **Accession** | **Gene name** | **Log fold-change** | **p-value** | **q-value** | **Phosphorylation identified** |
| **Uncharacterized protein DKFZp686N08224** | Q6MZY5 | DKFZp686N08224 | -2.97 | <0.001 | 0.014 | NO |
|  |  |  |  |  |  |  |

**Supplementary Information 36:** Total proteins for which the ratio between the relaxed-phase during spontaneous contractions condition and the pre-contracting condition (relaxed-phase during spontaneous contractions / pre-contracting) significantly (p≤0.05) at least doubled and passed FDR (q≤0.05) in the elective caesarean section group.

|  |  |  |  |  |  |  |
| --- | --- | --- | --- | --- | --- | --- |
| **Protein name** | **Accession** | **Gene name** | **Log-FC** | **p-value** | **q-value** | **Phosphorylation identified** |
| **Parvalbumin** | H0Y3U0 | PVALB | 4.70 | <0.001 | 0.048 | NO |
| **Heat shock 70 kDa protein 6 (Heat shock 70 kDa protein B') (Heat shock protein family A member 6)** | P17066 | HSPA6 | 3.34 | <0.001 | 0.010 | NO |
| **DNA repair protein XRCC1 (X-ray repair cross-complementing protein 1)** | P18887 | XRCC1 | 2.60 | <0.001 | 0.048 | NO |
| **GTP binding protein overexpressed in skeletal muscle, isoform CRA_a** | A0A024R9F5 | GEM | 1.75 | <0.001 | 0.013 | NO |

**Supplementary Information 37:** Total proteins for which the ratio between the relaxed-phase during spontaneous contractions condition and the pre-contracting condition (relaxed-phase during spontaneous contractions / pre-contracting) significantly (p≤0.05) at least halved and passed FDR (q≤0.05) in the elective caesarean section group.

|  |  |  |  |  |  |  |
| --- | --- | --- | --- | --- | --- | --- |
| **Protein name** | **Accession** | **Gene name** | **Log-FC** | **p-value** | **q-value** | **Phosphorylation identified** |
| **P2Y purinoceptor 14 (P2Y14) (G-protein coupled receptor 105) (UDP-glucose receptor)** | Q15391 | P2RY14 | -3.19 | <0.001 | 0.049 | YES |
| **Sodium-dependent phosphate transporter 2 (Gibbon ape leukemia virus receptor 2) (GLVR-2) (Phosphate transporter 2) (PiT-2) (Pit2) (hPit2) (Solute carrier family 20 member 2)** | Q08357 | SLC20A2 | -2.41 | <0.001 | <0.001 | NO |
| **Transforming growth factor beta-2 proprotein (Cetermin) (Glioblastoma-derived T-cell suppressor factor) (G-TSF) [Cleaved into: Latency-associated peptide (LAP); Transforming growth factor beta-2 (TGF-beta-2)]** | P61812 | TGFB2 | -2.29 | <0.001 | 0.011 | NO |
| **Fibroblast growth factor 1 (FGF-1) (Acidic fibroblast growth factor) (aFGF) (Endothelial cell growth factor) (ECGF) (Heparin-binding growth factor 1) (HBGF-1)** | P05230 | FGF1 | -2.15 | <0.001 | 0.048 | NO |
| **Solute carrier family 16 (Monocarboxylic acid transporters), member 3, isoform CRA_a** | A0A024R8U1 | SLC16A3 | -1.92 | <0.001 | 0.048 | NO |
| **Large ribosomal subunit protein eL14 (60S ribosomal protein L14)** | Q6IPH7 | RPL14 | -1.58 | <0.001 | 0.013 | NO |
| **EGF-containing fibulin-like extracellular matrix protein 1 (Extracellular protein S1-5) (Fibrillin-like protein) (Fibulin-3) (FIBL-3)** | Q12805 | EFEMP1 | -1.47 | <0.001 | 0.048 | NO |
| **Cadherin 2, type 1, N-cadherin (Neuronal), isoform CRA_b** | A0A024RC42 | CDH2 | -1.43 | <0.001 | 0.011 | NO |
| **Latent-transforming growth factor beta-binding protein 1 (LTBP-1) (Transforming growth factor beta-1-binding protein 1) (TGF-beta1-BP-1)** | Q14766 | LTBP1 | -1.29 | <0.001 | 0.049 | NO |
| **Complement factor H (H factor 1)** | P08603 | CFH | -1.20 | <0.001 | 0.013 | NO |
| **Histidine-rich glycoprotein (Histidine-proline-rich glycoprotein)** | B2R8I2 | NA | -1.18 | <0.001 | 0.049 | NO |
|  |  |  |  |  |  |  |

Supplementary Information 38: Phosphopeptides for which the ratio between the contracted-phase during spontaneous contractions condition and the pre-contracting condition (contracted-phase during spontaneous contractions / pre-contracting) significantly (p≤0.05) at least halved and passed FDR (q≤0.05) in the elective caesarean section group.

|  |  |  |  |  |  |  |  |
| --- | --- | --- | --- | --- | --- | --- | --- |
| **Protein** | **Accession** | **Gene name** | **Sequence detected** | **Site** | **log 2 fc** | **p value** | **q value** |
| **Protein prune homolog 2 (BNIP2 motif-containing molecule at the C-terminal region 1)** | D6RTK6 | PRUNE2 | RASDSVFQPK | 1xPhospho [S699(100)] | -4.01 | <0.001 | 0.007 |
| **Monocarboxylate transporter 1 (MCT 1) (Solute carrier family 16 member 1)** | P53985 | SLC16A1 | KESKEEETSIDVAGKPNEVTK | 2xPhospho [S461(99.8); S467(89.3)] | -2.54 | <0.001 | 0.044 |
|  |  |  |  |  |  |  |  |

Supplementary Information 39: Phosphopeptides for which the ratio between the relaxed-phase during spontaneous contractions condition and the pre-contracting condition (relaxed-phase during spontaneous contractions/pre-contracting) significantly (p≤0.05) at least halved and passed FDR (q≤0.05) in the elective caesarean section group.

|  |  |  |  |  |  |  |  |
| --- | --- | --- | --- | --- | --- | --- | --- |
| **Protein** | **Accession** | **Gene name** | **Sequence detected** | **Site** | **log 2 fc** | **p value** | **q value** |
| **Protein prune homolog 2 (BNIP2 motif-containing molecule at the C-terminal region 1)** | D6RTK6 | PRUNE2 | RASDSVFQPK | 1xPhospho [S699(100)] | -3.95 | <0.001 | 0.00254 |
|  |  |  |  |  |  |  |  |

**Supplementary information 40:** Table showing results of functional enrichment analysis of KEGG pathways performed using the STRING database on all proteins for which there was a log-fold change available between the relaxed-phase during spontaneous contractions and pre-contracting (relaxed-phase during spontaneous contractions/pre-contracting) conditions in the elCS group (n=7670). The proteins were ranked by their log-fold change, and the Kolmogorov-Smirnov test was applied to detect whether proteins associated with each pathway were non-randomly distributed across the ranked list. The “Genes mapped” refers to the number of proteins identified in the comparison between the relaxed-phase during spontaneous contractions and pre-contracting (relaxed-phase during spontaneous contractions/pre-contracting) conditions found in the pathway, and the “Total genes in set” refers to the total number of proteins within the KEGG pathway; higher enrichment scores indicate stronger enrichment, and the direction of enrichment indicates the direction of enrichment (bottom = downregulated in IOL group, top = upregulated in IOL group; both ends = proteins both upregulated and downregulated); FDR is the false discovery rate, considered significant if ≤0.05.

|  |  |  |  |  |  |
| --- | --- | --- | --- | --- | --- |
| **Significantly enriched pathway** | **Genes mapped** | **Total genes in set** | **Enrichment score** | **Direction of enrichment** | **FDR** |
| **Malaria** | 22 | 46 | 0.77 | both ends | <0.001 |
| **Complement and coagulation cascades** | 54 | 82 | 0.72 | top | <0.001 |
| **Staphylococcus aureus infection** | 36 | 86 | 0.58 | both ends | <0.001 |
| **Systemic lupus erythematosus** | 40 | 94 | 0.47 | top | 0.008 |
| **Ribosome** | 103 | 131 | 0.26 | top | <0.001 |
| **Spliceosome** | 94 | 132 | 0.10 | top | <0.001 |
|  |  |  |  |  |  |

**Supplementary information 41:** Table showing results functional enrichment analysis of local network clusters performed using the STRING database on all proteins for which there was a log-fold change available between the relaxed-phase during spontaneous contractions and pre-contracting (relaxed-phase during spontaneous contractions/pre-contracting) conditions in the elCS group (n=7670). The proteins were ranked by their log-fold change, and the Kolmogorov-Smirnov test was applied to detect whether proteins associated with each cluster were non-randomly distributed across the ranked list. The “Genes mapped” refers to the number of proteins identified in the comparison between the relaxed-phase during spontaneous contractions and pre-contracting (relaxed-phase during spontaneous contractions/pre-contracting) conditions found in the pathway, and the “Total genes in cluster” refers to the total number of proteins within the STRING cluster; higher enrichment scores indicate stronger enrichment, and “Direction of enrichment” indicates the direction of enrichment (bottom = downregulated in IOL group, top = upregulated in IOL group; both ends = proteins both upregulated and downregulated); FDR is the false discovery rate, considered significant if ≤0.05.

|  |  |  |  |  |  |
| --- | --- | --- | --- | --- | --- |
| **Significantly enriched cluster** | **Genes mapped** | **Total genes in cluster** | **Enrichment score** | **Direction of enrichment** | **FDR** |
| **Mixed, incl. COVID-19, thrombosis and anticoagulation, and Inter-alpha-trypsin inhibitor heavy chain C-terminus** | 19 | 21 | 1.25 | top | <0.001 |
| **Mixed, incl. COVID-19, thrombosis and anticoagulation, and Scavenging of heme from plasma** | 24 | 26 | 1.15 | top | <0.001 |
| **Complement cascade** | 25 | 40 | 0.95 | top | <0.001 |
| **Complement and coagulation cascades, and Positive regulation of opsonization** | 74 | 115 | 0.91 | top | <0.001 |
| **Complement cascade** | 30 | 50 | 0.89 | top | <0.001 |
| **Complement and coagulation cascades, and Positive regulation of opsonization** | 69 | 109 | 0.87 | top | <0.001 |
| **Hemostasis, and Dissolution of Fibrin Clot** | 36 | 55 | 0.80 | top | <0.001 |
| **Elastic fibre formation, and Tolloid/BMP1 peptidase domain** | 23 | 34 | 0.79 | top | 0.002 |
| **Complement and coagulation cascades, and Protein-lipid complex** | 95 | 161 | 0.77 | top | <0.001 |
| **Hemostasis, and Dissolution of Fibrin Clot** | 35 | 50 | 0.76 | top | <0.001 |
| **Complement and coagulation cascades, and Protein-lipid complex** | 96 | 166 | 0.76 | top | <0.001 |
| **Mixed, incl. Complement and coagulation cascades, and Protein-lipid complex** | 98 | 172 | 0.74 | top | <0.001 |
| **Mixed, incl. Complement and coagulation cascades, and Protein-lipid complex** | 112 | 196 | 0.66 | top | <0.001 |
| **Mixed, incl. Interferon alpha/beta signaling, and Guanylate-binding protein, C-terminal** | 30 | 46 | 0.56 | top | 0.01 |
| **Mixed, incl. Hemoglobin complex, and Actin filament capping** | 32 | 49 | 0.50 | bottom | 0.008 |
| **Mixed, incl. S100/CaBP-9k-type, calcium binding, subdomain, and Cystatin superfamily** | 51 | 103 | 0.41 | bottom | 0.005 |
| **Mixed, incl. Glutathione metabolism, and Detoxification of Reactive Oxygen Species** | 45 | 76 | 0.40 | bottom | 0.01 |
| **Preribosome, and Ribosome biogenesis** | 36 | 133 | 0.39 | top | 0.009 |
| **Preribosome, and Ribosome biogenesis** | 40 | 144 | 0.38 | top | 0.009 |
| **Mixed, incl. Glutathione metabolism, and Detoxification of Reactive Oxygen Species** | 54 | 93 | 0.35 | bottom | 0.008 |
| **Cytoplasmic ribosomal proteins** | 23 | 24 | 0.31 | top | <0.001 |
| **Proteasome** | 47 | 53 | 0.30 | bottom | 0.002 |
| **Proteasome** | 39 | 42 | 0.30 | bottom | 0.006 |
| **Proteasome, and Proteasome assembly** | 49 | 59 | 0.30 | bottom | 0.003 |
| **Cytoplasmic ribosomal proteins** | 51 | 53 | 0.29 | top | <0.001 |
| **Cytoplasmic ribosomal proteins** | 31 | 32 | 0.29 | top | <0.001 |
| **Cytoplasmic ribosomal proteins** | 39 | 40 | 0.29 | top | <0.001 |
| **Cytoplasmic ribosomal proteins** | 44 | 45 | 0.29 | top | <0.001 |
| **Cytoplasmic ribosomal proteins** | 57 | 61 | 0.28 | top | <0.001 |
| **Proteasome** | 44 | 47 | 0.28 | bottom | 0.004 |
| **Cytoplasmic ribosomal proteins** | 62 | 66 | 0.28 | top | <0.001 |
| **Viral mRNA Translation** | 63 | 71 | 0.28 | top | <0.001 |
| **Extracellular matrix organization** | 105 | 180 | 0.27 | top | 0.001 |
| **Viral mRNA Translation** | 65 | 76 | 0.27 | top | <0.001 |
| **Viral mRNA Translation** | 66 | 81 | 0.27 | top | <0.001 |
| **Viral mRNA Translation** | 67 | 87 | 0.27 | top | <0.001 |
| **Viral mRNA Translation, and Sec61 translocon complex** | 77 | 108 | 0.24 | top | <0.001 |
| **Eukaryotic Translation Elongation, and This family consists of several GAGE and XAGE proteins which are found exclusively in humans. The function of this family is unknown although they have been implicated in human cancers (PMID:11992404)** | 82 | 131 | 0.23 | top | <0.001 |
| **Eukaryotic Translation Elongation, and Sec61 translocon complex** | 81 | 116 | 0.22 | top | <0.001 |
| **Eukaryotic Translation Elongation, and This family consists of several GAGE and XAGE proteins which are found exclusively in humans. The function of this family is unknown although they have been implicated in human cancers (PMID:11992404)** | 85 | 140 | 0.22 | top | <0.001 |
| **Eukaryotic Translation Elongation, and This family consists of several GAGE and XAGE proteins which are found exclusively in humans. The function of this family is unknown although they have been implicated in human cancers (PMID:11992404)** | 88 | 150 | 0.21 | top | <0.001 |
| **Mixed, incl. Eukaryotic Translation Elongation, and This family consists of several GAGE and XAGE proteins which are found exclusively in humans. The function of this family is unknown although they have been implicated in human cancers (PMID:11992404)** | 95 | 163 | 0.19 | top | <0.001 |
| **Mixed, incl. Eukaryotic Translation Elongation, and This family consists of several GAGE and XAGE proteins which are found exclusively in humans. The function of this family is unknown although they have been implicated in human cancers (PMID:11992404)** | 98 | 168 | 0.19 | top | <0.001 |
|  |  |  |  |  |  |

**Supplementary information 42:** Table showing results of functional enrichment analysis of KEGG pathways performed using the STRING database on all proteins for which there was a log-fold change available between the contracted-phase during spontaneous contractions and the pre-contracting conditions (contracted-phase during spontaneous contractions / pre-contracting) in the elCS group (n=7667). The proteins were ranked by their log-fold change, and the Kolmogorov-Smirnov test was applied to detect whether proteins associated with each pathway were non-randomly distributed across the ranked list. The “Genes mapped” refers to the number of proteins identified in the comparison between the contracted-phase during spontaneous contractions and pre-contracting (contracted-phase during spontaneous contractions /pre-contracting) conditions found in the pathway, and the “Total genes in set” refers to the total number of proteins within the KEGG pathway; higher enrichment scores indicate stronger enrichment, and the direction of enrichment indicates the direction of enrichment (bottom = downregulated in IOL group, top = upregulated in IOL group; both ends = proteins both upregulated and downregulated); FDR is the false discovery rate, considered significant if ≤0.05.

|  |  |  |  |  |  |
| --- | --- | --- | --- | --- | --- |
| **Significantly enriched pathway** | **Genes mapped** | **Total genes in set** | **Enrichment score** | **Direction of enrichment** | **FDR** |
| **Complement and coagulation cascades** | 54 | 82 | 1.16 | top | <0.001 |
| **Staphylococcus aureus infection** | 36 | 86 | 1.04 | both ends | <0.001 |
| **Systemic lupus erythematosus** | 40 | 94 | 0.67 | both ends | <0.001 |
| **Ribosome** | 103 | 131 | 0.35 | top | <0.001 |
|  |  |  |  |  |  |

**Supplementary information 43:** Table showing results functional enrichment analysis of local network clusters performed using the STRING database on all proteins for which there was a log-fold change available between the contracted-phase during spontaneous contractions and the pre-contracting conditions (contracted-phase during spontaneous contractions / pre-contracting) in the elCS group (n=7667). The proteins were ranked by their log-fold change. Two statistical methods were used depending on the cluster: the Kolmogorov-Smirnov (KS) test was applied to detect whether proteins associated with each cluster were non-randomly distributed across the ranked list; and the average fold change (AFC) method, which calculates mean log-fold change of proteins within each cluster compared to background. The “Genes mapped” refers to the number of proteins identified in the comparison between the contracted-phase during spontaneous contractions and pre-contracting (contracted-phase during spontaneous contractions /pre-contracting) conditions found in the cluster, and the “Total genes in cluster” refers to the total number of proteins within the STRING cluster; higher enrichment scores indicate stronger enrichment, and “Direction of enrichment” indicates the direction of enrichment (bottom = downregulated in IOL group, top = upregulated in IOL group; both ends = proteins both upregulated and downregulated); FDR is the false discovery rate, considered significant if ≤0.05.

|  |  |  |  |  |  |  |
| --- | --- | --- | --- | --- | --- | --- |
| **Significantly enriched cluster** | **Genes mapped** | **total genes in cluster** | **Enrichment score** | **Direction of enrichment** | **FDR** | **Method** |
| **Transcription factor AP-1 complex, and AP-1 transcription factor** | 3 | 6 | 4.57 | bottom | <0.001 | AFC |
| **Response of EIF2AK1 (HRI) to heme deficiency, and Transcription factor AP-1 complex** | 6 | 18 | 2.93 | bottom | <0.001 | AFC |
| **Mixed, incl. Peptide amidation, and TonB box, conserved site** | 5 | 6 | 2.79 | top | <0.001 | AFC |
| **Mixed, incl. Basic region leucin zipper, and Early growth response, N-terminal** | 8 | 32 | 2.68 | bottom | 0.002 | AFC |
| **Membrane attack complex** | 6 | 7 | 2.35 | top | <0.001 | AFC |
| **Mixed, incl. Inter-alpha-trypsin inhibitor heavy chain C-terminus, and Alpha-1-acid glycoprotein** | 9 | 10 | 1.97 | top | 0.002 | AFC |
| **Chemokine receptors bind chemokines** | 9 | 64 | 1.85 | both ends | 0.008 | AFC |
| **Mixed, incl. COVID-19, thrombosis and anticoagulation, and Inter-alpha-trypsin inhibitor heavy chain C-terminus** | 19 | 21 | 1.85 | top | <0.001 | KS |
| **Mixed, incl. COVID-19, thrombosis and anticoagulation, and Scavenging of heme from plasma** | 24 | 26 | 1.74 | top | <0.001 | KS |
| **COVID-19, thrombosis and anticoagulation, and Negative regulation of fibrinolysis** | 10 | 11 | 1.74 | top | 0.003 | AFC |
| **Formation of the cornified envelope, and Serpin, conserved site** | 19 | 105 | 1.52 | bottom | 0.002 | AFC |
| **Formation of the cornified envelope, and Autosomal recessive congenital ichthyosis** | 20 | 125 | 1.49 | bottom | 0.002 | AFC |
| **Complement cascade** | 25 | 40 | 1.44 | top | <0.001 | KS |
| **Complement and coagulation cascades, and Positive regulation of opsonization** | 74 | 115 | 1.42 | top | <0.001 | KS |
| **Complement cascade** | 30 | 50 | 1.33 | top | <0.001 | KS |
| **Complement and coagulation cascades, and Positive regulation of opsonization** | 69 | 109 | 1.32 | top | <0.001 | KS |
| **Hemostasis, and Dissolution of Fibrin Clot** | 35 | 50 | 1.26 | top | <0.001 | KS |
| **Hemostasis, and Dissolution of Fibrin Clot** | 36 | 55 | 1.22 | top | <0.001 | KS |
| **Complement and coagulation cascades, and Protein-lipid complex** | 95 | 161 | 1.20 | top | <0.001 | KS |
| **Complement and coagulation cascades, and Protein-lipid complex** | 96 | 166 | 1.19 | top | <0.001 | KS |
| **Mixed, incl. Complement and coagulation cascades, and Protein-lipid complex** | 98 | 172 | 1.16 | top | <0.001 | KS |
| **Initial triggering of complement, and Regulation of complement activation** | 17 | 31 | 1.15 | top | 0.005 | AFC |
| **Mixed, incl. Complement and coagulation cascades, and Protein-lipid complex** | 112 | 196 | 1.07 | top | <0.001 | KS |
| **Mixed, incl. Glutathione metabolism, and Detoxification of Reactive Oxygen Species** | 54 | 93 | 0.48 | bottom | 0.002 | KS |
| **Mixed, incl. Glutathione metabolism, and Detoxification of Reactive Oxygen Species** | 58 | 100 | 0.46 | bottom | 0.003 | KS |
| **Precatalytic spliceosome, and Renpenning syndrome** | 37 | 52 | 0.42 | top | 0.004 | KS |
| **Mixed, incl. Glutathione metabolism, and Detoxification of Reactive Oxygen Species** | 68 | 115 | 0.42 | bottom | 0.008 | KS |
| **Cytoplasmic ribosomal proteins** | 31 | 32 | 0.37 | top | 0.002 | KS |
| **Cytoplasmic ribosomal proteins** | 39 | 40 | 0.37 | top | <0.001 | KS |
| **Viral mRNA Translation** | 63 | 71 | 0.36 | top | <0.001 | KS |
| **Viral mRNA Translation** | 66 | 81 | 0.36 | top | <0.001 | KS |
| **Cytoplasmic ribosomal proteins** | 62 | 66 | 0.36 | top | <0.001 | KS |
| **Cytoplasmic ribosomal proteins** | 57 | 61 | 0.35 | top | <0.001 | KS |
| **Cytoplasmic ribosomal proteins** | 51 | 53 | 0.35 | top | <0.001 | KS |
| **Viral mRNA Translation** | 67 | 87 | 0.35 | top | <0.001 | KS |
| **Cytoplasmic ribosomal proteins** | 44 | 45 | 0.35 | top | <0.001 | KS |
| **Viral mRNA Translation** | 65 | 76 | 0.35 | top | <0.001 | KS |
| **Spliceosomal snRNP complex, and mRNA cis splicing, via spliceosome** | 46 | 65 | 0.33 | top | 0.002 | KS |
| **Viral mRNA Translation, and Sec61 translocon complex** | 77 | 108 | 0.32 | top | <0.001 | KS |
| **Eukaryotic Translation Elongation, and Sec61 translocon complex** | 81 | 116 | 0.31 | top | <0.001 | KS |
| **Eukaryotic Translation Elongation, and This family consists of several GAGE and XAGE proteins which are found exclusively in humans. The function of this family is unknown although they have been implicated in human cancers (PMID:11992404)** | 82 | 131 | 0.30 | top | <0.001 | KS |
| **Eukaryotic Translation Elongation, and This family consists of several GAGE and XAGE proteins which are found exclusively in humans. The function of this family is unknown although they have been implicated in human cancers (PMID:11992404)** | 88 | 150 | 0.27 | top | <0.001 | KS |
| **Eukaryotic Translation Elongation, and This family consists of several GAGE and XAGE proteins which are found exclusively in humans. The function of this family is unknown although they have been implicated in human cancers (PMID:11992404)** | 85 | 140 | 0.27 | top | <0.001 | KS |
| **Mixed, incl. Eukaryotic Translation Elongation, and This family consists of several GAGE and XAGE proteins which are found exclusively in humans. The function of this family is unknown although they have been implicated in human cancers (PMID:11992404)** | 95 | 163 | 0.25 | top | <0.001 | KS |
| **Mixed, incl. Eukaryotic Translation Elongation, and This family consists of several GAGE and XAGE proteins which are found exclusively in humans. The function of this family is unknown although they have been implicated in human cancers (PMID:11992404)** | 98 | 168 | 0.24 | top | <0.001 | KS |
|  |  |  |  |  |  |  |

**Supplementary Information 44:** Accession numbers and protein descriptions for total proteins where the comparison between the relaxed-phase during spontaneous contractions and pre-contracting conditions ratio (relaxed-phase during spontaneous contractions /pre-contracting) at least halved for all myometrial samples in the validation group, in order of mean relaxed-phase during spontaneous contractions/pre-contracting ratio (n=3).

| **Protein description** | **Gene** | **Accession** | **Mean REL/PRE** |
| --- | --- | --- | --- |
| **Acid-sensing ion channel 5** | ASIC5 | Q9NY37 | 0.174675 |
| **Putative heat shock protein HSP 90-beta 4** | HSP90AB4P | Q58FF6 | 0.179087 |
| **COL8A2 protein (Fragment)** | COL8A2 | Q4VAP9 | 0.190707 |
| **T-cell lymphoma invasion and metastasis 1 variant (Fragment)** |  | Q59GK8 | 0.191501 |
| **Alternative protein SV2A** | SV2A | L8E840 | 0.192258 |
| **Protein CXorf40B (Fragment)** | CXorf40B | S4R3G8 | 0.192549 |
| **Heavy chain of factor I (Fragment)** |  | Q6LAM1 | 0.198744 |
| **Leucine-rich repeat transmembrane neuronal protein 3** | LRRTM3 | Q86VH5 | 0.202634 |
| **Complement C2** | C2 | P06681 | 0.203823 |
| **MHC class I antigen (Fragment)** | HLA-C | J9PWW9 | 0.204 |
| **Alpha-2-HS-glycoprotein** | AHSG | P02765 | 0.204276 |
| **Isocitrate dehydrogenase [NAD] subunit alpha, mitochondrial (Fragment)** | IDH3A | H0YLI6 | 0.206203 |
| **ETS domain-containing protein Elk-3 (Fragment)** | ELK3 | F8VZQ0 | 0.210359 |
| **Uncharacterized protein** |  | B4E1Z4 | 0.215487 |
| **cDNA FLJ37293 fis, clone BRAMY2014813** |  | Q8N1Y0 | 0.215557 |
| **Afamin** | AFM | P43652 | 0.216285 |
| **Lymphocyte cytosolic protein 2 (Fragment)** | LCP2 | E5RKA2 | 0.220541 |
| **Ig kappa chain V-III region GOL OS=Homo sapiens PE=1 SV=1 - [KV307_HUMAN]** |  | P04206 | 0.221674 |
| **cDNA FLJ53950, highly similar to Angiotensinogen** |  | B4E1B3 | 0.224544 |
| **Serotransferrin (Fragment)** | TF | C9JVG0 | 0.226664 |
| **Gasdermin-B** | GSDML | B2CM73 | 0.229643 |
| **V4-1 protein (Fragment)** | V4-1 | Q5NV68 | 0.230223 |
| **Nuclear pore complex protein Nup98-Nup96 (Fragment)** | NUP98 | H0YDF4 | 0.233823 |
| **Complement factor I light chain** | CFI | G3XAM2 | 0.234673 |
| **cDNA FLJ50624** |  | B7Z5L2 | 0.236541 |
| **MRDS1 protein (Fragment)** | MRDS1 | Q8IZS4 | 0.237525 |
| **Transferrin variant (Fragment)** |  | Q53H26 | 0.239237 |
| **Alpha-1-antichymotrypsin** | SERPINA3 | P01011 | 0.241487 |
| **Alpha-1-acid glycoprotein 2** | ORM2 | P19652 | 0.241575 |
| **Ceruloplasmin (Fragment)** |  | Q1L857 | 0.242832 |
| **Myosin-reactive immunoglobulin light chain variable region (Fragment)** |  | Q9UL83 | 0.243847 |
| **cDNA, FLJ94361, highly similar to Homo sapiens serine (or cysteine) proteinase inhibitor, clade A(alpha-1 antiproteinase, antitrypsin), member 6 (SERPINA6), mRNA** |  | B2R9F2 | 0.244553 |
| **Sex hormone-binding globulin** | SHBG | I3L145 | 0.246362 |
| **Alpha-1-antitrypsin** | SERPINA1 | P01009 | 0.246759 |
| **Thyroxine-binding globulin** | SERPINA7 | P05543 | 0.247522 |
| **Serum albumin** | ALB | P02768 | 0.247628 |
| **Myosin-reactive immunoglobulin heavy chain variable region (Fragment) OS=Homo sapiens PE=2 SV=1 - [Q9UL88_HUMAN]** |  | Q9UL88 | 0.251172 |
| **N-deacetylase/N-sulfotransferase (Heparan glucosaminyl) 1 variant (Fragment)** |  | Q59GK2 | 0.252713 |
| **cDNA FLJ46572 fis, clone THYMU3041573** |  | Q6ZR80 | 0.258675 |
| **cDNA FLJ53075, highly similar to Kininogen-1** |  | B4DPP8 | 0.25953 |
| **Antithrombin-III** | SERPINC1 | P01008 | 0.262913 |
| **Peroxisomal proliferator-activated receptor A interacting complex 285** | PRIC285 | A7E2C9 | 0.267948 |
| **HSPC254 (Fragment)** |  | Q9P0C7 | 0.268763 |
| **Hemopexin** | HPX | P02790 | 0.269767 |
| **Apolipoprotein A-I** | APOA1 | P02647 | 0.275777 |
| **Torsin family 3, member A, isoform CRA_b** | TOR3A | A0A024R943 | 0.277476 |
| **Vitamin D-binding protein** | GC | P02774 | 0.277481 |
| **Alpha-1-acid glycoprotein 1** | ORM1 | P02763 | 0.277913 |
| **IGL@ protein** | IGL@ | Q6PIK1 | 0.278325 |
| **Ig kappa chain V-I region DEE** |  | P01597 | 0.278384 |
| **C-reactive protein(1-205)** | CRP | Q5VVP7 | 0.279079 |
| **Transthyretin** | TTR | P02766 | 0.27967 |
| **Ig heavy chain V-III region TUR** |  | P01779 | 0.279983 |
| **tRNA (guanine-N(7)-)-methyltransferase non-catalytic subunit WDR4** | WDR4 | P57081 | 0.282096 |
| **V3-4 protein (Fragment)** | V3-4 | Q5NV62 | 0.282735 |
| **Haptoglobin** | HP | P00738 | 0.285602 |
| **Rheumatoid factor RF-ET6 (Fragment)** |  | A2J1N5 | 0.287805 |
| **IgG H chain OS=Homo sapiens PE=2 SV=1 - [S6BGE0_HUMAN]** |  | S6BGE0 | 0.289817 |
| **Putative upstream-binding factor 1-like protein 6** | UBTFL6 | P0CB48 | 0.291095 |
| **Claudin-10** | CLDN10 | P78369 | 0.291845 |
| **60S ribosomal protein L10-like** | RPL10L | Q96L21 | 0.292394 |
| **Ig heavy chain variable region (Fragment)** |  | A0A068LKQ0 | 0.294428 |
| **cDNA, FLJ93914, highly similar to Homo sapiens histidine-rich glycoprotein (HRG), mRNA** |  | B2R8I2 | 0.295174 |
| **Ig kappa chain V-III region POM** |  | P01624 | 0.296392 |
| **Ig kappa chain V-I region Roy** |  | P01608 | 0.301331 |
| **Retinol binding protein 4, plasma, isoform CRA_b** | RBP4 | Q5VY30 | 0.303165 |
| **Zinc finger MYM-type protein 3** | ZMYM3 | A6NHN7 | 0.303994 |
| **Putative uncharacterized protein DKFZp686G11190** | DKFZp686G11190 | Q6MZQ6 | 0.306337 |
| **IgG L chain** |  | S6BAR0 | 0.306421 |
| **Myosin-reactive immunoglobulin heavy chain variable region (Fragment)** |  | Q9UL89 | 0.306845 |
| **NK1 transcription factor-related protein 2** | NKX1-2 | Q9UD57 | 0.310642 |
| **ATP synthase protein 8** | ATP8 | Q6RM58 | 0.313637 |
| **V1-22 protein (Fragment)** | V1-22 | Q5NV88 | 0.313656 |
| **Alpha-2-antiplasmin** | SERPINF2 | P08697 | 0.314162 |
| **Ig lambda chain V-II region BUR** |  | P01708 | 0.314365 |
| **Myosin-reactive immunoglobulin light chain variable region (Fragment)** |  | Q9UL70 | 0.314583 |
| **Ig gamma-2 chain C region** | IGHG2 | P01859 | 0.318381 |
| **Ig heavy chain variable region (Fragment)** |  | A0A068LKQ5 | 0.320759 |
| **Immunoglobulin heavy chain variant (Fragment)** |  | Q9NPP6 | 0.322146 |
| **Apolipoprotein A-II (Fragment)** | APOA2 | V9GYG9 | 0.328051 |
| **Heparin cofactor 2** | SERPIND1 | P05546 | 0.328895 |
| **Putative uncharacterized protein DKFZp686K18196 (Fragment)** | DKFZp686K18196 | Q6N092 | 0.329612 |
| **Ig kappa chain V-I region Wes** |  | P01611 | 0.33268 |
| **VH3 protein (Fragment)** | VH3 | Q9Y509 | 0.333364 |
| **cDNA FLJ14473 fis, clone MAMMA1001080, highly similar to Homo sapiens SNC73 protein (SNC73) mRNA** |  | Q96K68 | 0.333596 |
| **Coagulation factor XII-Mie** |  | Q8IZZ5 | 0.334685 |
| **Protein IGKV2D-24 (Fragment)** | IGKV2D-24 | A0A075B6R9 | 0.335101 |
| **Kininogen 1, isoform CRA_b** | KNG1 | B4E1C2 | 0.336223 |
| **Complement component C6** | C6 | P13671 | 0.338469 |
| **Putative uncharacterized protein DKFZp686K04218 (Fragment)** | DKFZp686K04218 | Q7Z379 | 0.339185 |
| **Complement C3** | C3 | P01024 | 0.339726 |
| **Haptoglobin-related protein** | HPR | P00739 | 0.3411 |
| **Myosin-reactive immunoglobulin light chain variable region (Fragment)** |  | Q9UL78 | 0.342398 |
| **Bone marrow proteoglycan** | PRG2 | P13727 | 0.347319 |
| **cDNA FLJ51742, highly similar to Inter-alpha-trypsin inhibitor heavy chain H4** |  | B7Z544 | 0.347931 |
| **DF protein** | DF | Q6FHW3 | 0.348498 |
| **Alpha-2-macroglobulin** | A2M | P01023 | 0.350212 |
| **Epididymis luminal protein 180 (Fragment)** | HEL180 | B6EDE2 | 0.350831 |
| **Ig kappa chain V-IV region STH (Fragment)** |  | P83593 | 0.350888 |
| **Complement factor H-related protein 2** | CFHR2 | V9GYE7 | 0.351515 |
| **Coagulation factor XI** | F11 | D6RB32 | 0.351786 |
| **Rearranged VH4-34 V gene segment (Fragment)** | VH4-34 | Q7Z3Y6 | 0.354457 |
| **cDNA FLJ54981, highly similar to Asparaginyl-tRNA synthetase, cytoplasmic (EC 6.1.1.22)** |  | B4DF91 | 0.357127 |
| **Protein IGKV3-11** | IGKV3-11 | A0A087WZW8 | 0.357979 |
| **Complement component C8 alpha chain** | C8A | P07357 | 0.358861 |
| **Cryocrystalglobulin CC1 heavy chain variable region (Fragment)** |  | B1N7B6 | 0.358917 |
| **Anti-H1N1 influenza HA kappa chain variable region (Fragment)** |  | G3GAU4 | 0.359899 |
| **IgG H chain** |  | S6BGD4 | 0.361408 |
| **Ig lambda-7 chain C region (Fragment)** | IGLC7 | A0A075B6L1 | 0.361505 |
| **Uncharacterized protein** |  | Q7Z2U7 | 0.36181 |
| **cDNA FLJ78071, highly similar to Human MHC class III complement component C6 mRNA** |  | A8K8Z4 | 0.363487 |
| **Ig kappa chain V-I region BAN** |  | P04430 | 0.364185 |
| **Zinc-alpha-2-glycoprotein** | AZGP1 | P25311 | 0.367259 |
| **Immunoglobulin J chain** | IGJ | P01591 | 0.369822 |
| **Complement C4A3 (Fragment)** | C4A | V9H0D6 | 0.370672 |
| **Complement component 9, isoform CRA_a** | C9 | A0A024R035 | 0.372345 |
| **Tetranectin** | CLEC3B | P05452 | 0.37671 |
| **Ig kappa chain V-I region Lay** |  | P01605 | 0.377028 |
| **Ig mu heavy chain disease protein** |  | P04220 | 0.378461 |
| **Ig mu chain C region** | IGHM | A0A087WYJ9 | 0.37923 |
| **Ig kappa chain C region** | IGKC | A0A087WYL9 | 0.379857 |
| **cDNA, FLJ95492, highly similar to Homo sapiens myosin regulatory light chain interacting protein(MIR), mRNA** |  | B2RBG2 | 0.383065 |
| **Protein AMBP** | AMBP | P02760 | 0.391841 |
| **Apolipoprotein L1 (Fragment)** | APOL1 | U5LKR8 | 0.394813 |
| **Molybdenum cofactor synthesis-step 1 protein B splice type I (Fragment)** | MOCS1 | Q9NP26 | 0.397583 |
| **cDNA, FLJ79457, highly similar to Insulin-like growth factor-binding proteincomplex acid labile chain** |  | B0AZL7 | 0.398735 |
| **Carboxypeptidase N catalytic chain** | CPN1 | P15169 | 0.399288 |
| **Probable Xaa-Pro aminopeptidase 3** | XPNPEP3 | Q9NQH7 | 0.401726 |
| **Transcription factor AP-2-epsilon** | TFAP2E | Q6VUC0 | 0.406294 |
| **Ig kappa chain V-I region EU** |  | P01598 | 0.406536 |
| **CD5 antigen-like** | CD5L | O43866 | 0.410149 |
| **Ig gamma-3 chain C region** | IGHG3 | A0A087WXL8 | 0.416541 |
| **Phosphatidylinositol 3,4,5-trisphosphate 5-phosphatase 1** | INPP5D | Q92835 | 0.417309 |
| **Single chain Fv (Fragment)** |  | Q9HCC1 | 0.423404 |
| **Calcium/calmodulin-dependent protein kinase type 1B** | PNCK | Q6P2M8 | 0.427963 |
| **cDNA FLJ78207, highly similar to Human complement protein component C7 mRNA** |  | A8K2T4 | 0.436489 |
| **Glutamate [NMDA] receptor subunit epsilon 2 variant (Fragment)** |  | Q59HA9 | 0.455341 |
| **Carboxypeptidase B2** | CPB2 | A0A087WSY5 | 0.490011 |

**Supplementary Information 45:** Accession numbers and protein descriptions for total proteins where the comparison between the relaxed-phase during spontaneous contractions and pre-contracting conditions ratio (relaxed-phase during spontaneous contractions/pre-contracting) ratio at least doubled for all samples in the validation group, in order of mean relaxed-phase during spontaneous contractions/pre-contracting ratio (n=3).

| **Protein description** | **Gene** | **Accession** | **Mean REL/PRE** |
| --- | --- | --- | --- |
| **Mitogen-activated protein kinase 11** | MAP3K11 | E9PID4 | 3.604 |
| **Tristetraprolin (Fragment)** | ZFP36 | M0QZ04 | 3.437 |
| **Transcription factor jun-B** | JUNB | P17275 | 4.243 |
| **C-C motif chemokine 2** | CCL2 | P13500 | 4.187 |
| **Cyclin-dependent kinase inhibitor 1** | CDKN1A | P38936 | 6.547 |

**Supplementary Information 46:** 25 most overrepresented total protein pathways according to Reactome analysis (ordered by ascending p-value) where the comparison between the relaxed-phase during spontaneous contractions and pre-contracting conditions ratio (relaxed-phase during spontaneous contractions condition/pre-contracting condition) in the validation sample at least halved (n=3). The entities found represent the number of entities found in the validation dataset that are present in the named pathway, divided by the total number of known entities in that particular pathway. The entities p-value is the statistical test for over-representation, as described, and the entities FDR corrects the p-value test for multiple testing. The reactions found represent the number of pathways identified in the overall pathway for which there is at least one entity from the study dataset found. The reactions ratio is the ratio of reactions in the specific pathway which contain entities from the study dataset, divided by the total number of reactions within the Reactome database which contain entities from the study dataset.

| **Pathway Name** | **Entities found** | **p-value for entities found** | **FDR for entities found** | **Reactions found** | **Reactions ratio** |
| --- | --- | --- | --- | --- | --- |
| **Scavenging of heme from plasma** | 20/99 | 1.11x10-16 | 6.66x10-15 | 12/12 | 9.61x10-4 |
| **Regulation of complement cascade** | 23/135 | 1.11x10-16 | 6.66x10-15 | 35/42 | 0.003 |
| **Complement cascade** | 24/146 | 1.11x10-16 | 6.66x10-15 | 57/71 | 0.006 |
| **Binding and uptake of ligands by scavenger receptors** | 20/129 | 2.22x10-16 | 9.99x10-15 | 17/33 | 0.003 |
| **Initial triggering of complement** | 17/111 | 4.75x10-14 | 1.71x10-12 | 14/21 | 0.002 |
| **Classical antibody-mediated complement activation** | 15/95 | 1.03x10-12 | 3.09x10-11 | 2/2 | 1.60x10-4 |
| **Creation of C4 and C2 activators** | 15/103 | 3.20x10-12 | 3.09x10-11 | 2/2 | 1.60x10-4 |
| **FCGR activation** | 14/101 | 3.32x10-11 | 7.31x10-10 | 6/6 | 4.8x10-4 |
| **CD22 mediated BCR regulation** | 12/70 | 8.26x10-11 | 1.65x10-9 | ¾ | 3.2x10-4 |
| **Role of phospholipids in phagocytosis** | 14/114 | 1.59x10-10 | 2.85x10-9 | 5/12 | 9.61x10-4 |
| **FCERI mediated Ca+2 mobilisation** | 14/117 | 2.21x10-10 | 3.54x10-9 | 5/11 | 8.81x10-4 |
| **FCERI mediated MAPK activation** | 14/119 | 2.75x10-10 | 4.12x10-9 | 9/20 | 0.002 |
| **Role of LAT2/NTAL/LAB on calcium mobilisation** | 13/102 | 4.73x10-10 | 6.62x10-9 | 2/7 | 5.61x10-4 |
| **Haemostasis** | 31/723 | 5.42x10-10 | 7.04x10-9 | 24/327 | 0.026 |
| **Antigen activates B cell receptor (BCR) leading to generation of second messengers** | 12/95 | 2.5x10-9 | 3x10-8 | 10/25 | 0.002 |
| **Regulation of actin dynamics for phagocytic cup formation** | 14/150 | 5.13x10-9 | 5.64x10-8 | 6/24 | 0.002 |
| **Fcgamma receptor (FCGR) dependent phagocytosis** | 14/175 | 3.44x10-8 | 3.44x10-7 | 17/42 | 0.003 |
| **Cell surface interactions at the vascular wall** | 16/246 | 5.87x10-8 | 5.87x10-7 | 3/64 | 0.005 |
| **FCERI mediated NF-kB activation** | 13/167 | 1.48x10-7 | 1.33x10-6 | 1/19 | 0.002 |
| **Fc epsilon receptor (FCERI) signaling** | 14/218 | 4.82x10-7 | 4.32x10-6 | 22/63 | 0.005 |
| **Platelet degranulation** | 11/128 | 5.40x10-7 | 4.32x10-6 | 3/11 | 8.81x10-4 |
| **Response to elevated platelet cytosolic Ca2+** | 11/133 | 7.82x10-7 | 6.26x10-6 | 3/14 | 0.001 |
| **Signaling by the B Cell Receptor (BCR)** | 12/176 | 1.79x10-6 | 1.25x10-5 | 13/43 | 0.003 |
| **Innate Immune System** | 33/1186 | 3.81x10-6 | 2.67x10-5 | 108/696 | 0.056 |
| **Post-translational protein phosphorylation** | 9/107 | 7.12x10-6 | 4.99x10-5 | 1/1 | 8.01x10-5 |

**Supplementary Information 47:** Ten most overrepresented pathways according to Reactome analysis (ordered by ascending p-value) where proteins in the comparison between the relaxed-phase during spontaneous contractions and pre-contracting conditions (relaxed-phase during spontaneous contractions condition/pre-contracting condition) in the validation samples at least doubled.

| **Pathway Name** | **Entities found** | **p-value for entities found** | **FDR for entities found** | **Reactions found** | **Reactions ratio** |
| --- | --- | --- | --- | --- | --- |
| **Interleukin-4 and Interleukin-13 signaling** | 3/111 | 9.22x10-6 | 7.37x10-4 | 3/46 | 0.004 |
| **Cytokine Signalling in Immune system** | 4/954 | 2.3x10-4 | 0.009 | 5/699 | 0.056 |
| **Signaling by Interleukins** | 3/456 | 6.1x10-4 | 0.016 | 4/490 | 0.039 |
| **Transcriptional activation of cycle inhibitor p21** | 1/4 | 0.002 | 0.028 | 1/5 | 4x10-4 |
| **Transcriptional activation of p53 responsive genes** | 1/4 | 0.002 | 0.028 | 1/5 | 4x10-4 |
| **TFAP2 (AP-2) family regulates transcription of cell cycle factors** | 1/5 | 0.002 | 0.028 | 1/5 | 4x10-4 |
| **RUNX3 regulates CDKNIA transcription** | 1/7 | 0.003 | 0.032 | 2/6 | 4.8x10-4 |
| **TP53 Regulates transcription of genes involved in G1 cell cycle arrest** | 1/14 | 0.006 | 0.032 | 3/17 | 0.001 |
| **AKT phosphorylates targets in the cytosol** | 1/14 | 0.006 | 0.032 | 1/9 | 7.21x10-4 |
| **FOXO-mediated transcription of cell cycle genes** | 1/17 | 0.007 | 0.032 | 1/22 | 0.002 |


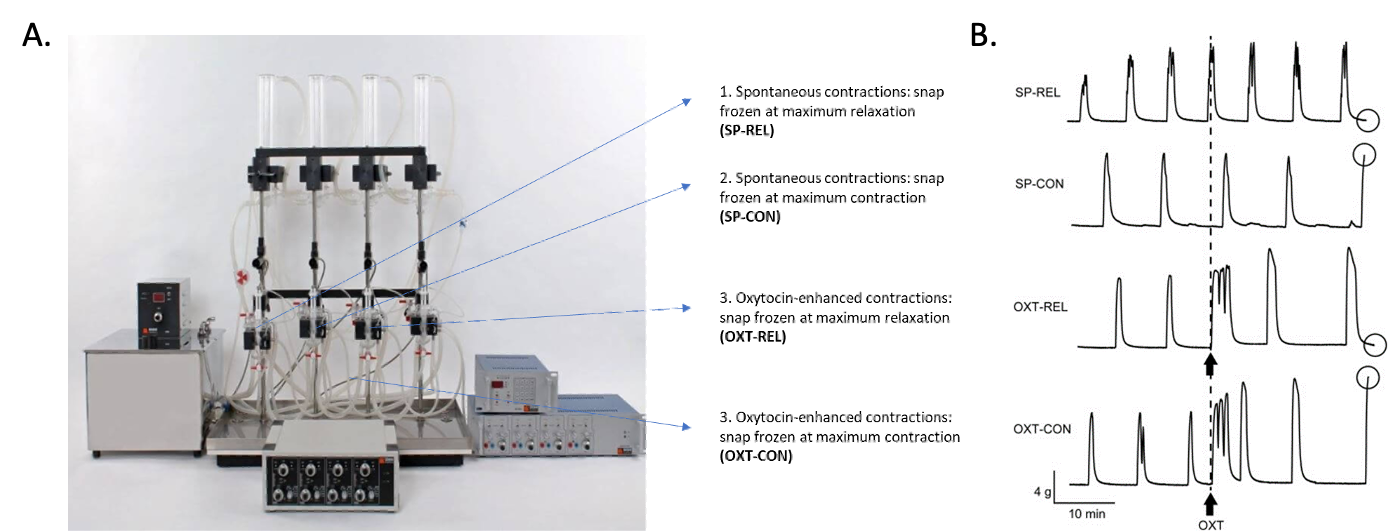
**Supplementary Information 48:** A. Myobath-II system with experimental conditions used in each of the four chambers used (World Precision Instruments); B. Example of electronic output of myometrial samples connected to force-transducers using Lab-Trax data acquisition system (World Precision Instruments) (figure adapted from Hudson & López-Bernal, 2012). Circles indicate when the samples were snap frozen, where: SP-REL = relaxed-phase during spontaneous contractions condition, where the sample was snap-frozen 20 seconds following the end of a phasic contraction; SP-CON = contracted-phase during spontaneous contractions condition, where the sample was snap-frozen at peak phasic contraction; OXT-REL = relaxed-phase during oxytocin-induced contractions, where the sample was snap-frozen 20 seconds following the end of a phasic contraction; and OXT-CON = contracted-phase during oxytocin-induced contractions, where the sample was snap-frozen 20 seconds following the end of a phasic contraction.

Supplementary Information 49: Flow diagram for methodology for phosphoproteomics analysis for failed IOL vs elCS study


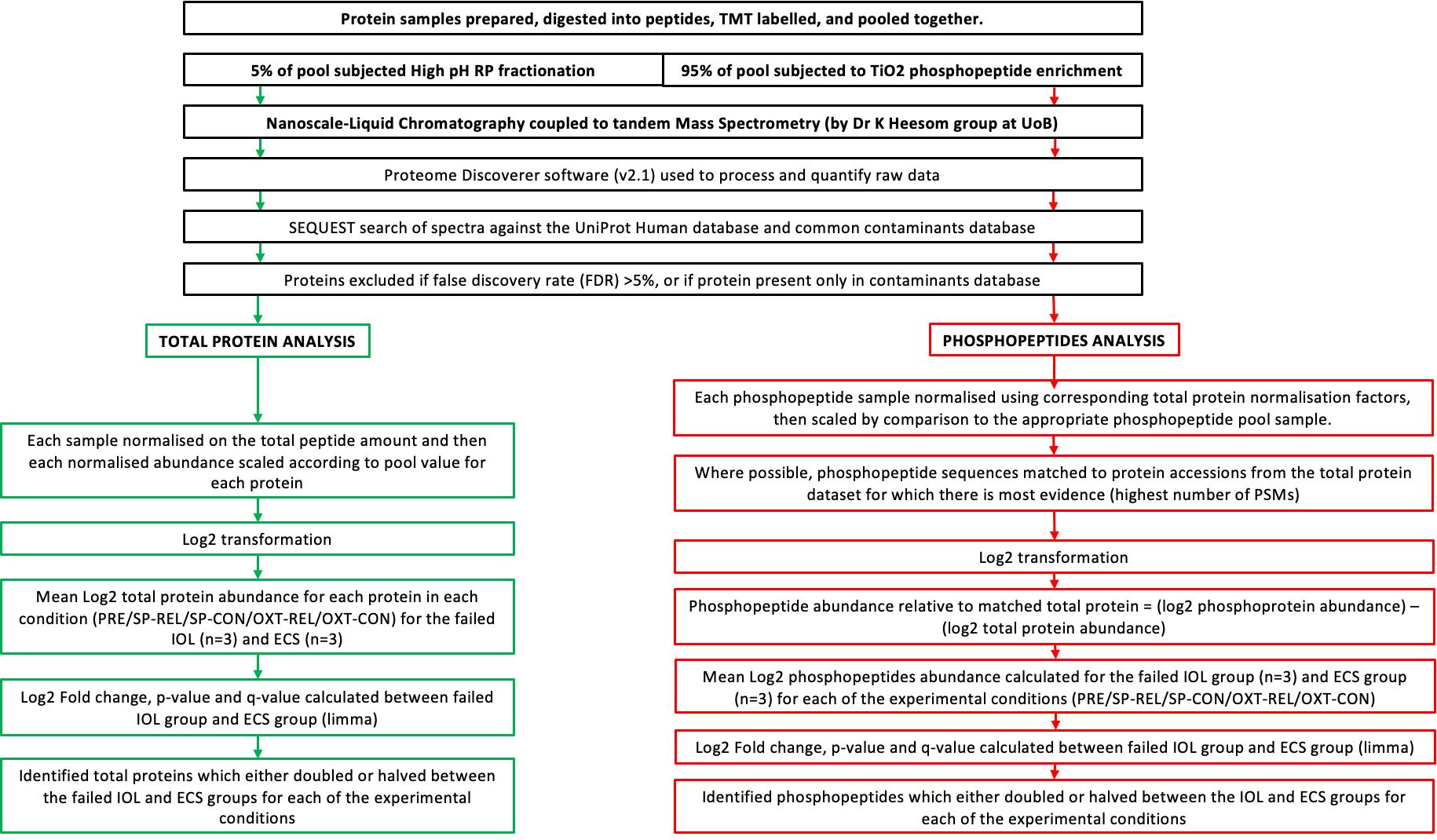


Supplementary Information 50: Flow diagram for methodology for analysis conducted for the validation study (SP-REL/PRE only) (PSM = Peptide Spectral Matches)


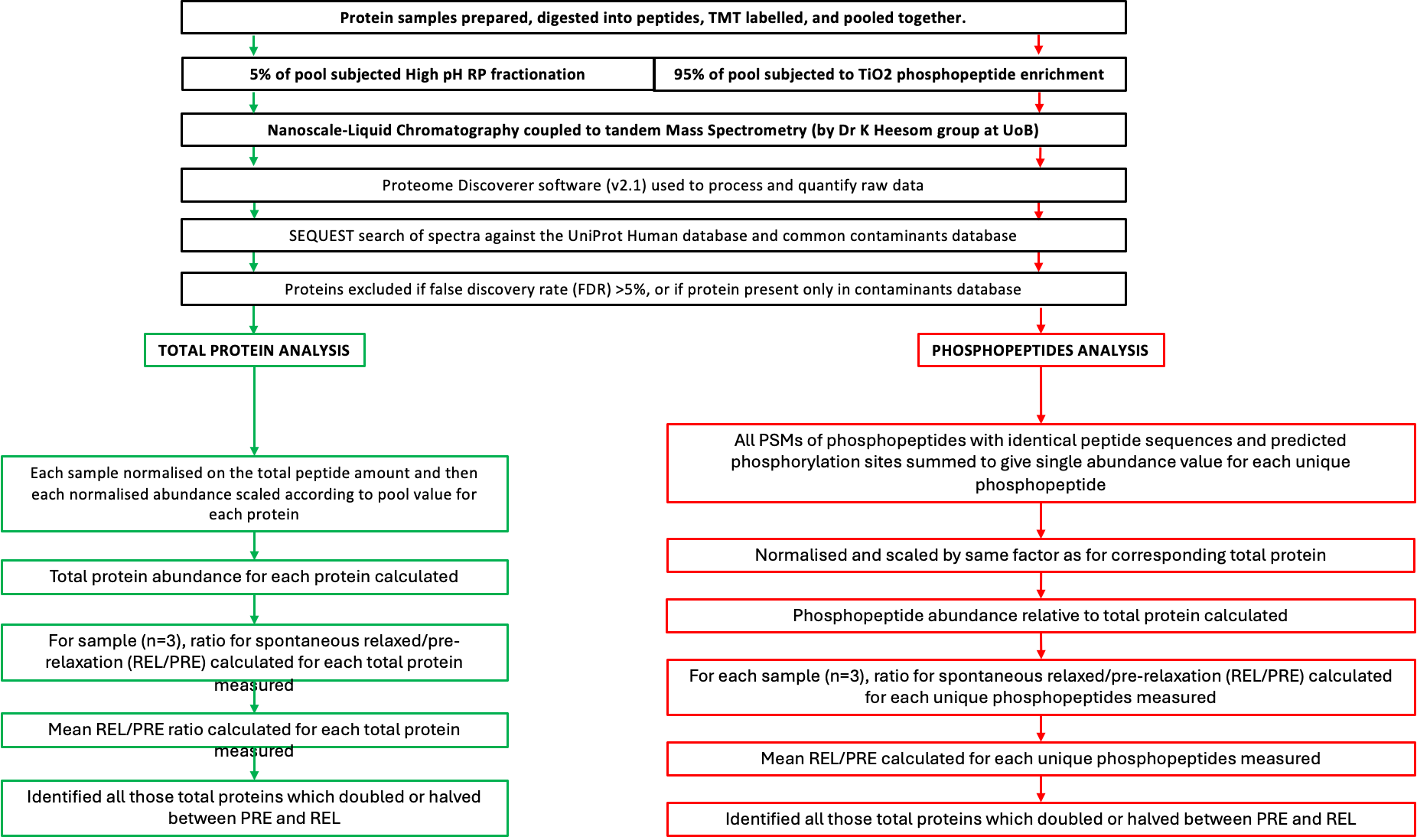

Supplement: Supplementary file 1 — Supplementary Material 1 [file 41598_2025_27605_MOESM1_ESM.docx]
